# Ru(II)-Based Multitopic Hosts for Fullerene Binding: Impact of the Anion in the Recognition Process

**DOI:** 10.1021/acs.inorgchem.4c04608

**Published:** 2025-01-30

**Authors:** Adriana Sacristán-Martín, Nerea Álvarez-Llorente, Alberto Diez-Varga, Héctor Barbero, Celedonio M. Álvarez

**Affiliations:** † GIR MIOMeT, IU CINQUIMA/Química Inorgánica, Facultad de Ciencias, 16782 Universidad de Valladolid, Valladolid E47011, Spain

## Abstract

The development of multitopic hosts for fullerene recognition based on nonplanar corannulene (C_20_H_10_) structures presents challenges, primarily due to the requirement for synergistic interactions with multiple units of this polycyclic aromatic hydrocarbon. Moreover, increasing the number of corannulene groups in a single chemical structure while avoiding the cost of increasing flexibility has been scarcely explored. Herein, we report the synthesis of a family of multitopic Ru­(II)-polypyridyl complexes bearing up to six units of corannulene arranged by pairs, offering a total of three molecular tweezers. All of them are fixed by the central atom and organized in an octahedral structure. Their fullerene recognition capabilities have been thoroughly demonstrated toward C_60_ and C_70_ showing that they can reasonably accommodate up to three fullerenes per host in a noncooperative manner. There are, however, some features that diverge from comparable hosts in the literature, such as the low value of several association constants. This behavior, supported by theoretical studies, is attributed to the presence of two noninnocent BAr_4_^F^ anions that interfere with the supramolecular binding through ion pair formation. These findings highlight the crucial role of selecting compatible ionic species in supramolecular host design as they can significantly influence the recognition process.

## Graphical abstract

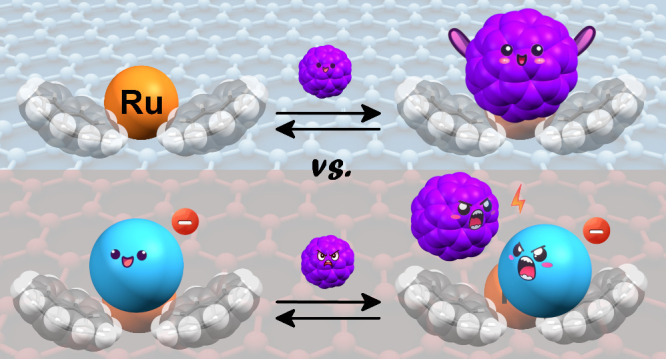

## Introduction

Curved polycyclic aromatic hydrocarbons (PAHs)^1,2^ are organic molecules characterized by an aromatic framework composed of multiple fused benzene rings, exclusively containing sp^2^ carbon atoms. Their unique geometry, deviating from planarity,^3,4^ grants them significant scientific interest. These nonplanar PAHs exhibit superior properties over planar analogs,^5,6^ such as enhanced solubility (better processing) and the ability to hierarchically self-assemble, making them ideal components of materials for applications in electronic and optoelectronic devices. Curvature arises from defects in the expected planar honeycomb-like structure of fused hexagons. The sole ortho fusion of benzene groups creates steric hindrance from only four rings with increasing repulsion as the number of aryls grows. This gives rise to helical structures in open arrangements.^7–9^ In closed structures, the presence of at least one ring with a different number of carbons (4, 5, 7 or 8, mainly)^10–12^ results in a geometry with two possible curvatures. For a number of carbons less than 6 (quadrannulene, corannulene), the geometry adopts a bowl-shaped structure (Gaussian positive curvature), whereas for a number of carbons over 6, such a geometry resembles a saddle structure (pleiadeannulene, [8]­circulene, with Gaussian negative curvature). Owing to their particular surface, they are capable of establishing strong supramolecular interactions with the convex outer surface of fullerenes,^13–19^ allowing, for instance, precise p–n junctions in active layers of optoelectronic materials from a bottom-up fabrication point of view.^20–22^

The most prominent member of the family is, however, corannulene ([5]­circulene), with a formula of C_20_H_10_. It is one of the most studied geodesic polyarenes thanks to its availability via regular chemical means^23,24^ or, more recently, by mechanochemical approaches.^25^ Structurally, corannulene consists of five hexagons fused around a central pentagon and is regarded as a hydrogen-terminated fragment of Buckminsterfullerene (C_60_). Its functionalization has been thoroughly studied to furnish new species with emergent properties (nonlinear emission, high electron affinity, electron transport) useful for potential applications in organic electronics and other technologies.^15,26,27^ The topology of corannulene, resembling a “cap” for C_60_, suggests excellent surface complementarity (concave–convex) that is expected to play a significant role in supramolecular binding. Consequently, various molecular tweezers incorporating two corannulene units linked by different spacer groups have been reported, demonstrating binding affinities influenced by factors such as (1) host preorganization (rigid and well-defined), (2) fullerene surface coverage (the higher, the better), and (3) synergy between the spacer group and the guest molecule (the spacer group recognizes the guest as well). Notable examples include Sygula’s buckycatchers family;^28–30^ Chen’s organic helicene;^31^ or our organic,^32–36^ organometallic,^37,100^ and inorganic^38^ pincers. The latter is especially relevant to this discussion (see below). It is also worth-mentioning that fullerene recognition can be remarkably enhanced when concealed within the cavity of molecular cages,^17,39–41^ which benefits from optimal guest surface coverage. In most cases, the stoichiometry of the resulting supramolecular adduct is 1:1, where a single molecular tweezer captures one fullerene molecule. This raises the intriguing question of whether it is feasible to design a multitopic fullerene receptor capable of recognizing multiple guests simultaneously (i.e., a multitopic receptor).

The search for curved molecular polyarenes-based multitopic hosts for fullerenes remains challenging nowadays. As of today, we can clearly answer the long-ago posed question by Sygula et al. (are three [corannulenes] better than two?)^42^ with a sound “no” or with a nuanced “possibly”, as the outcome is highly dependent on above-described factors. Our efforts toward creating multitopic corannulene-based hosts have highlighted the importance of preorganization in pincer-like structures;^32–38^ even minor modifications, such as the presence or absence of a methylene bridge,^37^ bipyridine conformational freedom,^38^ or mobility restrictions in sulfur-based biphenyls,^35^ can lead to absolute differences in recognition capabilities. However, when an additional recognition element, such as a porphyrin, is integrated alongside corannulene groups, the binding affinity for fullerenes is significantly enhanced, even if the overall preorganization is less optimal compared to systems without the assistance of an auxiliary motif.^43–45^ This is due to the strong synergistic effects among all recognition groups, which outweigh other energy-related penalties. Nonetheless, caution is warranted, as an excessively high association constant may induce negative allosteric effects, potentially hindering multitopic binding.^45^ In this context, Stuparu’s work has shown excellent results, with polymeric methacrylate-based materials incorporating pending corannulene groups demonstrating the ability to conceal up to 8 wt % of C_60_ or C_70_ in a micellar core.^46–48^ Although the exact arrangement of recognition motifs with the guest is not fully understood, given the nature of the receptor, it is suggested that up to three corannulene units can accommodate each carbon allotrope due to the adaptability conferred by the polymeric backbone.

As commented above, we have developed a Pt­(II)-based organometallic^37^ molecular tweezer (Figure 1a) bearing two corannulene ends grafted to the metal center by a rigid acetylide group with an enhanced affinity toward C_70_ with respect to C_60_. More recently, we reported a bidentate 2,2′-bipyridine (bpy) ligand functionalized at 4 and 4′ positions with (ethynyl)­corannulene that acts as a bistable molecular machine with reversible ON/OFF behavior upon coordination to a Cu­(I) center (Figure 1b).^38^ The operation principle was very simple and relied on the preference for the ligand to establish an *anti* conformation, keeping both corannulene groups far away from each other and thus preventing any fullerene recognition (OFF state). Copper grafting could fix the *syn* conformation, establishing a pincer-like structure where both corannulene units were at an appropriate distance. This permitted fullerene binding (ON state). The cycle can be repeated multiple times by removing the metal in situ. In light of these findings, we deemed the possibility of further exploiting this motif through coordination to other transition metals with accessible high coordination numbers. Ru­(II)-polypyridyl systems were identified as particularly promising.^49^ This large family of complexes has been extensively studied over the past four decades, resulting in a well-established body of chemistry. Their significant interest within the scientific community is largely attributed to their notable photophysical and electrochemical properties, which facilitate a wide range of applications in contemporary fields such as materials science, biomedicine, and (photo)­catalysis.^50–52^ Our design comprises a 4,4′-bis­(arylethynyl)-2,2′-bipyridine featuring either pyrene or corannulene aromatic termini coordinated to a Ru­(II) center (Figure 1c). The pyrene group was employed for comparative purposes, particularly with regard to its properties relevant to fullerene binding. We chose to incorporate an ethynyl spacer group since molecular tweezers with this feature typically exhibit superior performance compared to those with directly attached aryl groups. The versatility of the metal core allowed us to prepare complexes with one, two, and three molecular tweezers whose mobility is mainly restricted by the central scaffold offering the possibility to obtain a tritopic fullerene host as depicted in Figure 1d. In such an octahedral (CN = 6) complex, up to six corannulene groups, arranged by pairs through bpy spacers, are assembled so that they are expected to work independently due to the overall rigidity of the system. In other words, our priori assumption states that every single pincer would recognize fullerene without the cooperation of the other two (more details below). This is not necessarily a suboptimal design because, as mentioned above, the search for cooperativity might lead to undesired negative allosteric behavior.^45^

**Figure 1:**
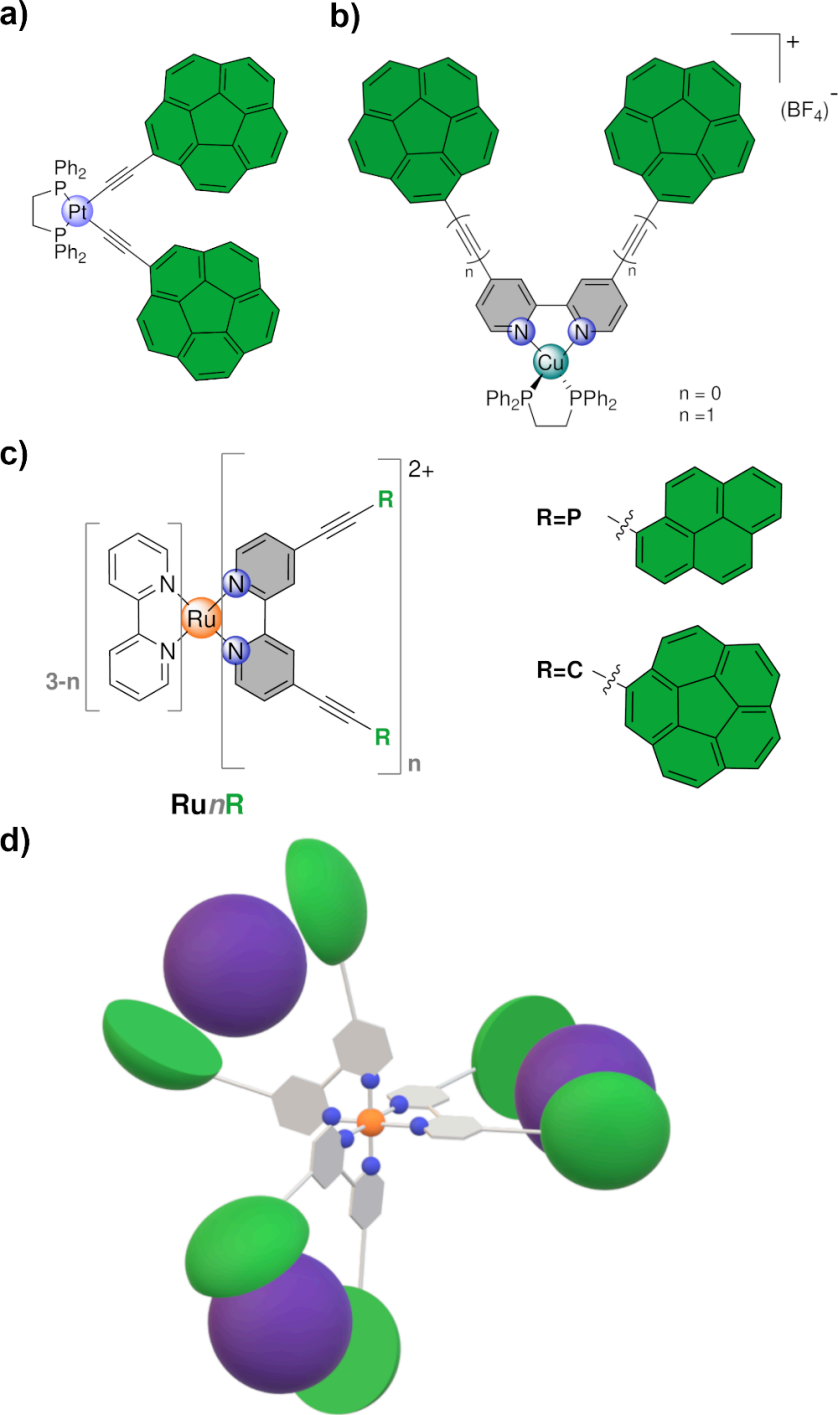
(a) A previously reported Pt­(II)-based organometallic host. (b) Cu­(I) complexes bearing 2,2′-bipyridine functionalized at positions 4 and 4′ with corannulene motifs acting as a receptor for fullerenes. (c) The family of Ru­(II)-bpy complexes reported in this work. (d) Cartoon depiction of the **(C_60_)_3_@Ru3C** supramolecular adduct (vide infra).

## Results and Discussion

### Synthesis of Target Complexes

Considering that the synthetic access to an already described substituted bpy ligand has been proven feasible,^38^ the most reasonable strategy comprises its sequential coordination to a suitable precursor such as the well-known dichloro­(1,5-cyclooctadiene)­ruthenium­(II) polymer^53,54^ or the pivotal complex [Ru­(DMSO)_4_Cl_2_],^55–57^ both established as excellent methods to achieve a high degree of functionalization around the metal center. None of these protocols succeeded, owing to a very low conversion despite the substantial excess of ligands used or the application of elevated temperatures (even under microwave irradiation). Apparently, these coordination reactions required a large energy input for activation, leading to ligand decomposition before achieving full conversion. We therefore focused our attention on an alternative method^58–61^ that consists of a final ligand functionalization after its coordination with a suitable bpy, as shown in Scheme 1. Thus, parent complex [RuCl_2_(COD)]_*n*_, prepared from Ru­(III) chloride,^62^ was split into two pathways. On one hand, complex **1**, a *cis*-dichlorido complex bearing two bpy ligands,^53^ was prepared (Scheme 1a) and functionalized with 4,4′-dibromo-2,2′-bipyridine to furnish cationic complex **Ru1Br**, which was subjected to a Sonogashira C–C cross coupling with the corresponding ethynyl precursor to give rise to intermediate complexes with hexafluorophosphate as the counterion. Due to their very poor solubility in common low-polarity solvents (see below in the host–guest chemistry studies), an anion exchange step was carried out with sodium tetrakis­(3,5-bis­(trifluoromethyl)­phenyl)­borate Na­(BAr_4_^F^) to furnish final expected compounds **Ru1P** and **Ru1C**. On the other hand, a distinct *cis*-dichlorido complex (**2**) was similarly prepared by using 4,4′-dibromo-2,2′-bipyridine as the ligand (see Scheme 1b). The synthetic sequence further diverges into two subpathways (Scheme 1c and d) resulting in the formation of complexes **Ru2Br** and R**u3Br**, analogous to compound **Ru1Br** differing primarily in the number of functionalizable ligands that are coordinated to Ru­(II). Final Sonogashira C–C cross coupling and subsequent anion exchange afforded desired complexes **Ru2P**, **Ru2C**, **Ru3P,** and **Ru3C**. Overall, yields were moderate to good across all cases, especially for the multiple coupling reactions, whose yields were on average 80% (see the experimental section for more details).

**Scheme 1:**
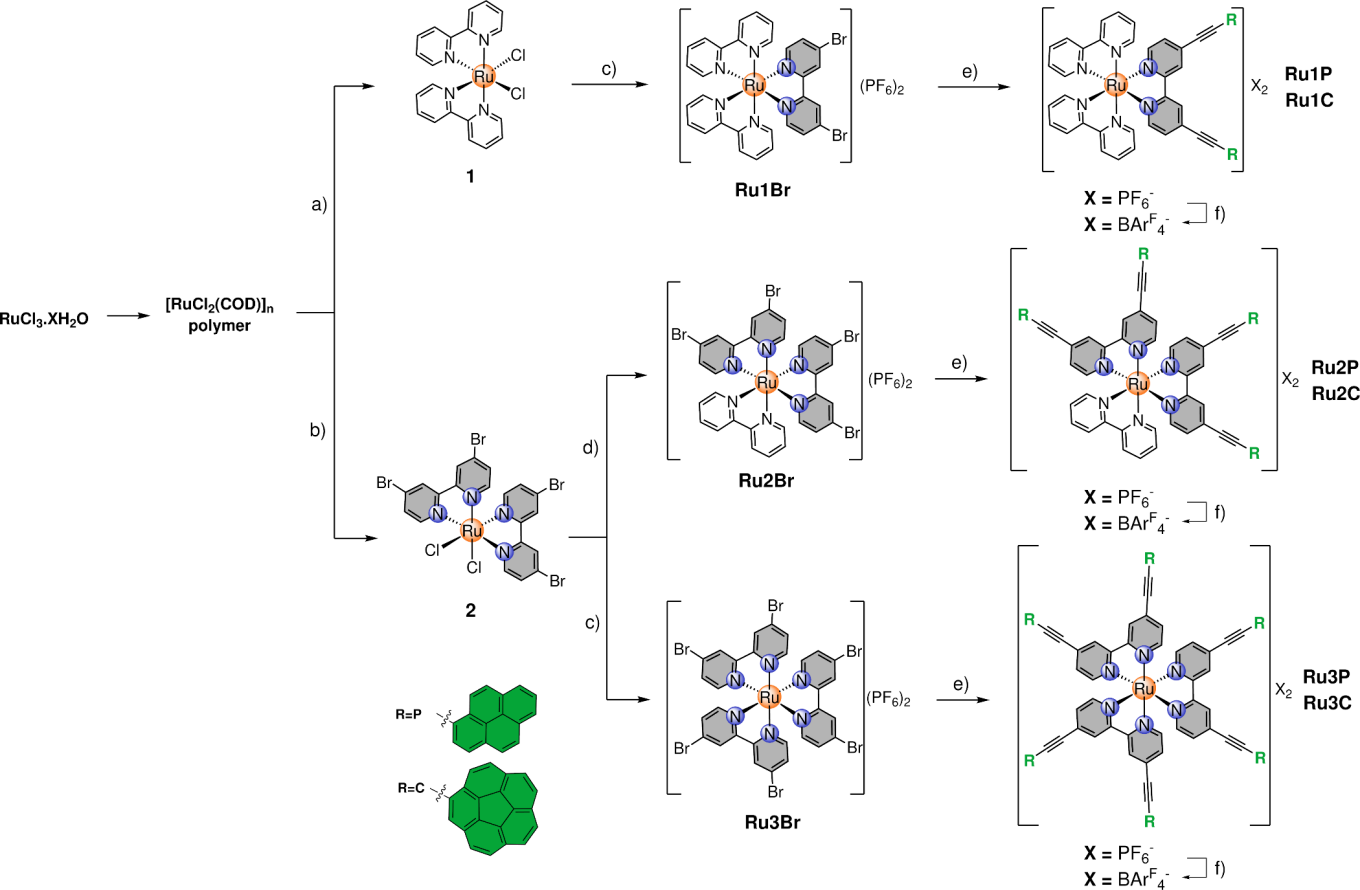
Preparation of the Whole Family of Ru­(II)-bpy Complexes Reported in This Work^a^

a Reagents and conditions: (a) 2,2′-bipyridine, dichlorobenzene, 180 °C. (b) 4,4′-dibromo-2,2′-bipyridine, dichlorobenzene, 180 °C. (c, *i*) 4,4′-dibromo-2,2′-bipyridine, EtOH, reflux; (*ii*) NH_4_PF_6_. (d, *i*) 2,2′-bipyridine, EtOH, reflux; (*ii*) NH_4_PF_6_. (e) PAH-acetylene, [PdCl_2_(dppf)], CuI, CH_3_CN/NEt_3_, THF, r.t. (f) Na­(BAr_4_^F^), CH_2_Cl_2_, r.t. Note: complexes **Ru1P** and **Ru1C** could also be prepared from precursor [RuCl_2_(DMSO)_4_], but with a much lower yield.

### Characterization of Ru­(II)-Polypyridyl Complexes

The entire collection of complexes was fully characterized in solution by spectroscopic methods, as well as by mass spectrometry.

With respect to NMR techniques, all aromatic protons resonate over a relatively broad spectral window between 9.0 and 7.4 ppm (Figure 2a,b,c). It is worth noting that the relatively simple ^1^H NMR spectrum of complex **Ru1C** (Figure 2a), whose corannulene groups are chemically equivalent, becomes significantly more intricate as the second corannulene-bearing bpy is coordinated owing to the introduction of chemical inequivalence, and therefore, signal splitting is observed (**Ru2C** in Figure 2b). Last, after grafting the third bpy derivative in **Ru3C**, the ^1^H NMR spectrum considerably simplifies due to the higher symmetry (D_3_ group) of the complex, as shown in Figure 2c. Typically, the two most deshielded signals in the NMR spectrum correspond to the protons at positions 3 and 3′ of the nitrogenated ligand, appearing as a singlet for substituted bpy and a doublet for unsubstituted bpy. These are followed by the signal of the proton nearest the substituted carbon attached to the ethynyl group in the aromatic hydrocarbon, which manifests as the only singlet in that moiety. Next, a doublet is observed for the second proton that is closest to the substituted carbon. At higher fields, the remaining signals arise from the aromatic hydrocarbon and the other bpy ligands with the number of peaks depending on the symmetry of the complex. A very similar behavior was observed for pyrene-derived series, and it was possible to assign all nuclei by 2D methods (refer to the Supporting Information file for further details).

**Figure 2:**
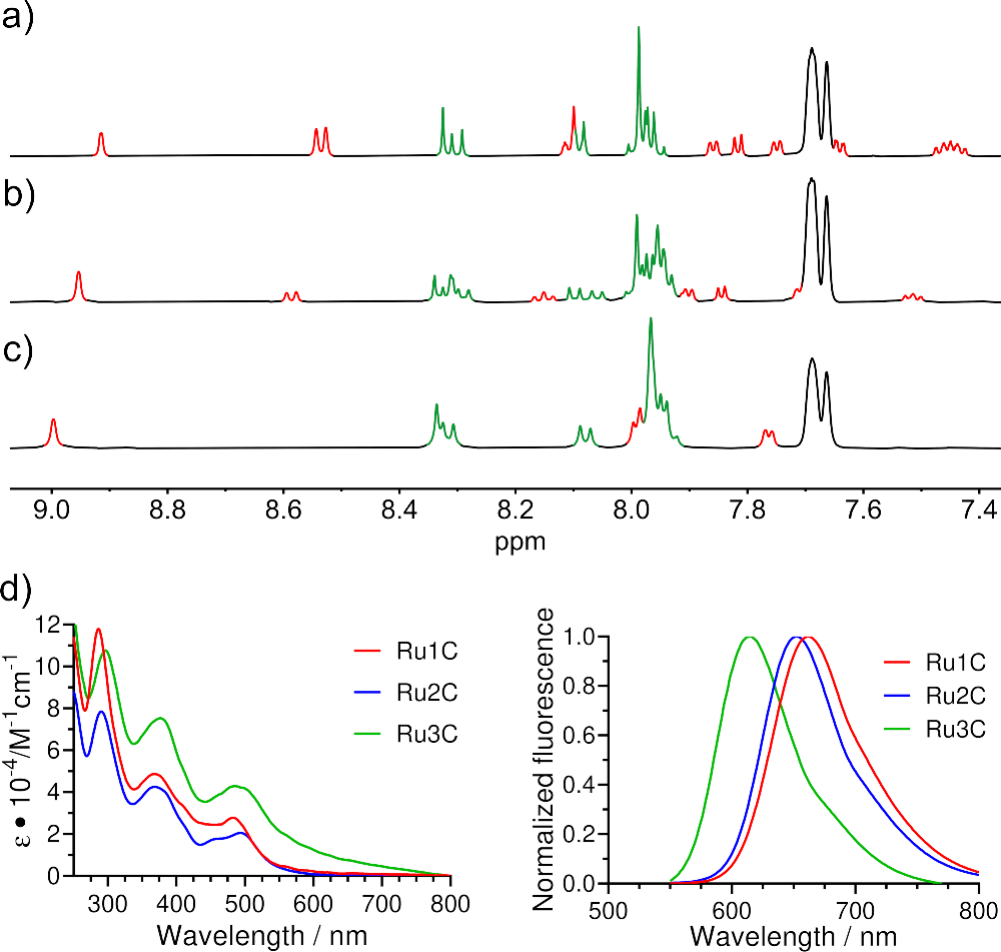
^1^H NMR (298 K, 500 MHz, acetonitrile-*d*_3_) spectra of complexes (a) **Ru1C**, (b) **Ru2C,** and (c) **Ru3C.** Peaks corresponding to bpy and corannulene hydrogens are highlighted in red and green, respectively. Black signals belong the to BAr_4_^F^ anion. (d) UV–vis absorption and normalized luminescence spectra of complexes **Ru1C** (λ_ex_ = 488 nm), **Ru2C** (λ_ex_ = 499 nm), and **Ru3C** (λ_ex_ = 487 nm) in acetonitrile at room temperature and a concentration of 1.0 × 10^–5^ M.

UV–vis absorption spectra show three distinct bands with a decreasing molar absorption coefficient as the wavelength increases in all Ru*n*C (*n* = 1, 2, and 3) complexes (Figure 2d). Typically,^58–61,63–65^ the band within the UV region (λ_abs_ = 285–294 nm) is attributed to intraligand π–π* transitions associated with bpy. Moving toward the visible region (λ_abs_ = 367–373 nm), a band corresponding to intraligand π–π* transitions of the C≡C–corannulene fragment can be observed. Within the visible portion of the spectrum (λ_abs_ = 485–493 nm), the expected combination of MLCT dπ­(Ru) → π*­(bpy) and MLCT dπ­(Ru) → π*­(C≡C–corannulene) transitions are shown. Ru*n*P complex series possesses similar features (see the Supporting Information).

Emission spectra of complexes Ru*n*C (Figure 2d) provide the expected broad unstructured band^58–61,63–66^ (λ_em_ = 615–662 nm) upon excitation of the MLCT state (λ_ex_ = 487–499 nm). The shorter wavelength for the emission of complex **Ru3C**, compared to those of **Ru1C** and **Ru2C**, suggests a more energetic excited state. Their decay lifetimes substantially increase (up to 4-fold) under deareated conditions, confirming the triplet nature of the emissive ^3^MLCT state and the phosphorescent character of the emission. Measured lifetimes (776–1556 ns) are in accordance with those reported for other RuL_3_^+^ trischelate complexes containing bidentate *N*-donor ligands^63^ (see the Supporting Information for more details).

Unfortunately, our efforts to obtain single crystals suitable for X-ray diffraction were unfruitful, and only intermediates were characterized with this technique (see the Supporting Information).

### Host–Guest Chemistry with Fullerenes

Given the relative location of both corannulene groups in the same bpy ligand and previous results in our group,^38^ it is reasonable to expect association constants in the range of 10^3^ to 10^5^ M^–1^ in toluene-*d*_8_. This solvent was chosen for comparison purposes with other systems previously published in the literature, given that the majority of association constants are reported in this medium. It provides good fullerene solubility^67,68^ and reasonable solubility of the complex. Therefore, NMR spectroscopy seems to be a feasible tool to appropriately determine them. It is worth mentioning that UV–vis absorption and emission experiments were tested but failed providing information about the supramolecular binding (see the Supporting Information for details). Indeed, the addition of aliquots of C_60_ or C_70_ to the hosts caused changes in several aromatic chemical shifts indicating a fast exchange regime and confirming supramolecular binding (Figure 3a).^69,70^ No changes were found for the Ru*n*P family, clearly indicating the need for positive Gaussian curvature as provided by the Ru*n*C series. As the number of cavities imposed by pairs of corannulenes increases from complex **Ru1C** through **Ru3C**, the binding stoichiometry might change accordingly. Thus, we systematically applied nonlinear regression fittings for 1:1, 1:2, and 1:3 stoichiometries (Figure 3b, see the Supporting Information for details). For stoichiometries beyond the binary 1:1 model, four interaction scenarios (“flavors”) were considered: full, additive, noncooperative, and statistical.^71,72^ Stepwise association constants are summarized in Table 1 and Table 2.

**Figure 3:**
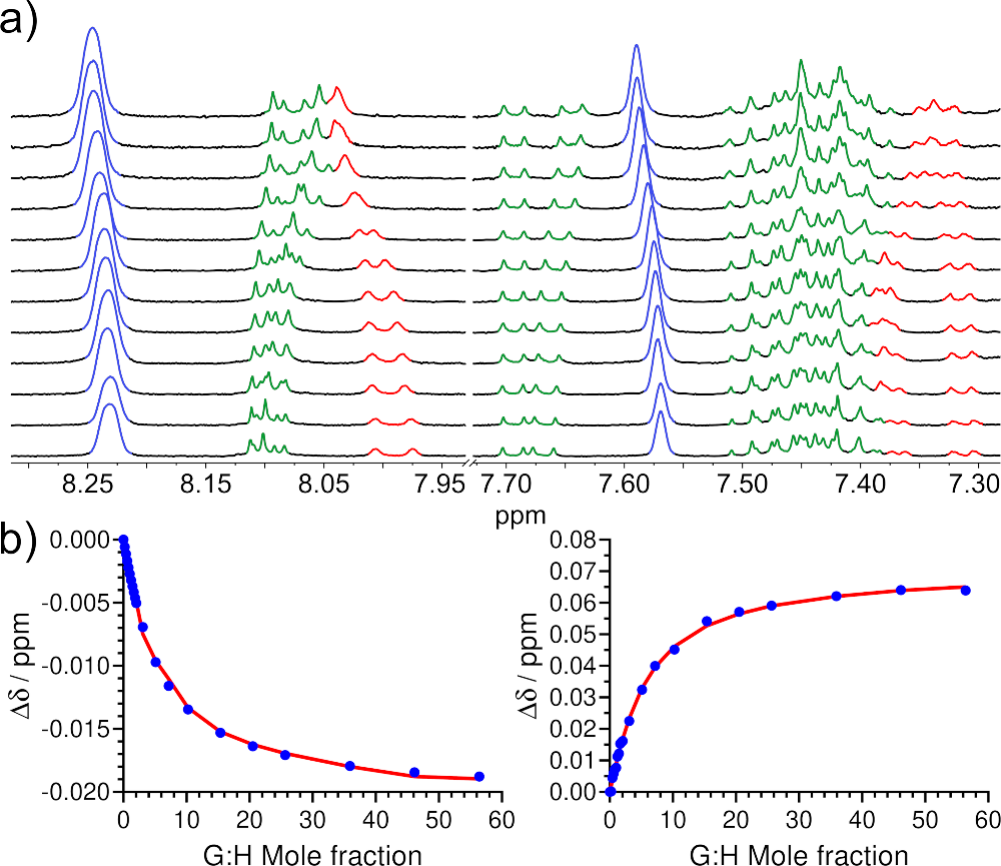
(a) Stacked ^1^H NMR (298 K, 500 MHz, toluene-*d*_*8*_) spectra of complex **Ru2C** with variable concentrations of C_70_. Chemical shifts of corannulene, bpy, and BAr_4_^F^ protons have been colored in green, red, and blue, respectively. (b) Variation of chemical shifts (Δδ) for two different signals versus G:H (guest mole fraction; blue points) along with a fitted binding isotherm obtained by nonlinear regression in a 1:2 noncooperative model (red line).

**Table 1:** Association Constants (in M^–1^) Obtained for Supramolecular Adducts between Synthesized Hosts **Ru1C**–**Ru3C**and C_60_ after Nonlinear Regressions with the Most Likely Binding Model^a^

complex	*K*_1_	*K*_2_	*K*_3_
**Ru1C**	(3.54 ± 0.19) × 10^2^		
**Ru2C** (noncoop)	(7.21 ± 0.15) × 10^2^	(1.80 ± 0.04) × 10^2^	
**Ru3C** (noncoop)	(5.97 ± 0.27) × 10^3^	(1.99 ± 0.09) × 10^3^	(6.63 ± 0.30) × 10^2^

a For host **Ru3C**, uncertainties were estimated with Monte Carlo simulations.^72,76^

**Table 2:** Association Constants (in M^–1^) Obtained for Supramolecular Adducts between Synthesized Hosts Ru1C–**Ru3 C** and C_70_ after Nonlinear Regressions with the Most Likely Binding Models^a^

complex	*K*_1_	*K*_2_	*K*_3_
**Ru1C**	(5.69 ± 0.08) × 10^2^		
**Ru2C**(full)	(1.98 ± 0.08) × 10^3^	(1.62 ± 0.08) × 10^3^	
**Ru2C**(noncoop)	(4.93 ± 0.05) × 10^3^	(1.23 ± 0.01) × 10^3^	
**Ru3C** (full)	(1.16 ± 0.05) × 10^9^	(2.76 ± 0.12) × 10^3^	(9.96 ± 0.60) × 10^2^
**Ru3C** (noncoop)	(1.31 ± 0.05) × 10^4^	(4.35 ± 0.18) × 10^3^	(1.45 ± 0.06) × 10^3^

a For host **Ru3C**, uncertainties were estimated with Monte Carlo simulations.^72,76^

Regarding C_60_ recognition, as expected, host **Ru1C** shows a preferred binding model of 1:1 stoichiometry, whereas for hosts **Ru2C** and **Ru3C**, 1:2 and 1:3 models with a noncooperative factor can reasonably describe the supramolecular association (Table 1). Covfit factors are higher than simpler models but not fully significant (2.32 in the best case).^72,73^ The noncooperative flavor means the binding of one fullerene does not alter the structure of the host and neither enhances nor hinders further bindings (refer to the Supporting Information for more details about this model). This outcome is plausible, as the rigid tweezers in an octahedral complex are unlikely to exert an effect on other binding sites within the same molecule. The most important finding is the relatively low values of the constants which are inferior to those of previous Cu­(I)-based molecular tweezers (1.15 × 10^3^ M^–1^ in CD_2_Cl_2_),^38^ with comparable values observed only for host **Ru3C**. They are also lower than other neutral hosts such as Chen’s corannulene rigid helicene (2.79 × 10^3^ M^–1^ in toluene)^31,74^ or our family of molecular tweezers of different natures (1.28 × 10^3^ to 4.70 × 10^3^ M^–1^ in toluene-*d*_8_)^32–37^ and clearly underperform when compared to Sygula’s buckycatchers (3.2 × 10^3^ in toluene-*d*_8_ to 1.0 × 10^4^ M^–1^ in chlorobenzene-*d*_5_).^28–30,75^

Regarding C_70_ recognition, the behavior of all complexes is essentially similar in terms of preferred stoichiometries as seen with C_60_ (Table 2). However, for hosts **Ru2C** (in 1:2 stoichiometry) and **Ru3C** (in 1:3 stoichiometry), no clear preference in flavor was found as both full and noncooperative models show comparable covfit factors. Complex **Ru2C** has similar association constants (both *K*_1_ and *K*_2_) between different flavors (on the same order of magnitude). On the other hand, complex **Ru3C** shows a very interesting behavior since (1) it shows the highest association constants of the entire family (log β = 10.9 for C_70_ vs 9.8 for C_60_ under the same noncooperative model) and (2) the *K*_1_ value is surprisingly high under the full flavor. This flavor makes no assumptions regarding the nature of the cavity or chemical shift correlation, thus allowing for potential cooperativity. The first finding can be explained based on previous reports where C_70_ usually binds stronger than C_60_ in this kind of host given its better complementarity and appropriate size to fit in the cavity.^28–33,35–38^ However, a simple explanation for the second finding cannot be stated with ease given that the cooperativity of the first binding step, calculated as 3*K*_2_/*K*_1_, returns a value of 7 × 10^–6^, indicating significant negative cooperativity (see the Supporting Information for more details). In other words, interactions in the supramolecular adduct **C_70_@Ru3C** are much stronger than those of **(C_70_)_2_@Ru3C** and **(C_70_)_3_@Ru3C**.^77^ This interpretation is supported by the slightly higher covfit factor for the full model compared to the noncooperative model (4.2 vs 3.98), indicating some significance.

A closer inspection of the ^1^H NMR spectra recorded during fullerene titrations revealed another relevant aspect initially overlooked: proton signals from the BAr_4_^F^ anion changed proportionally with all the other chemical shifts as fullerene aliquots were added (see blue peaks in Figure 3a). Since the Ru­(II) cation is expected to be the sole responsible component involved in host–guest chemistry with fullerenes, the anion is not expected to play any role. The BAr_4_^F^ anion was chosen due to the poor solubility of hexafluorophosphate salts in most solvents, under the assumption that it would remain “innocent” in the host–guest interaction studies. This effect prompted us to consider the anion to be involved in the adduct formation equilibria. Indeed, fitting its chemical shift changes to the same models returned constants very similar to those obtained by using corannulene and bpy protons. In fact, all models provided consistent results when including BAr_4_^F^ signals in the fitting process. Given that the bulky, negatively charged tetrahedral anion is unlikely to engage in strong attractive interactions with fullerenes, we initially hypothesized that two BAr_4_^F^ molecules were not fully solvated in toluene-*d*_*8*_, resulting in the formation of an ionic pair stabilized by strong electrostatic interactions. To test this hypothesis, we conducted DOSY experiments in a polar solvent such as CD_3_CN and found clearly distinct diffusion trends for the Ru­(II) cation and anions (Figure 4a). Conversely, when performing under the same conditions in toluene-*d*_8_, the cation and anions showed an identical diffusion coefficient (Figure 4b). This result clearly demonstrates the inability of toluene to solvate BAr_4_^F^ favoring the formation of an ion pair.^78–80^ Consequently, the presence of two bulky anions next to the cavities imposed by corannulene pairs in the molecular tweezers hinders the recognition, resulting in lower association constants. It remains unclear whether BAr_4_^F^ anions merely impose steric hindrance on the receptor or participate in additional interactions with corannulene moieties in solution. Furthermore, the addition of an excess of fullerene did not alter the diffusion of cations and anions in toluene-*d*_8_, suggesting that the ion pair persisted in the supramolecular adduct.

**Figure 4:**
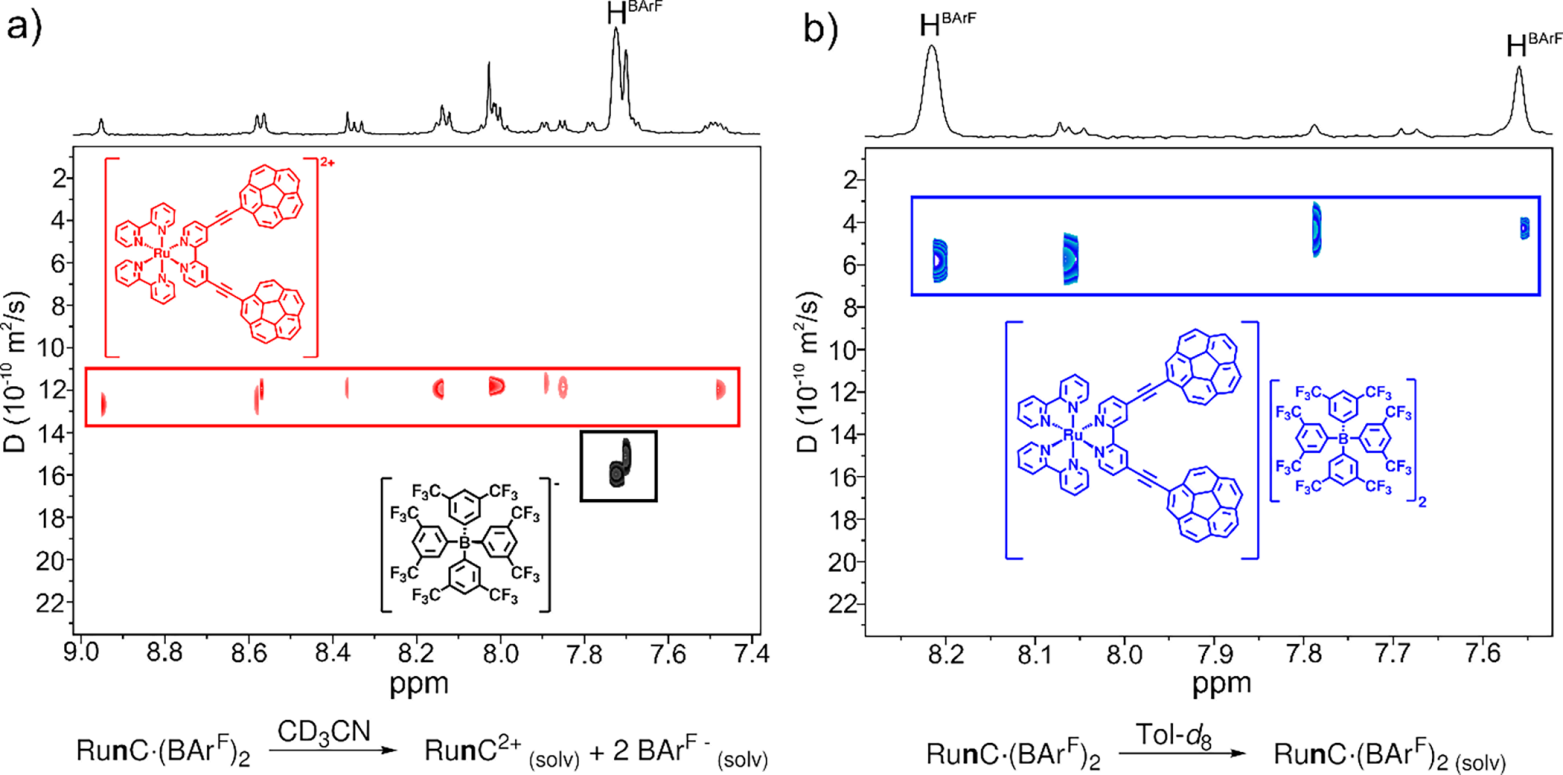
DOSY experiments (298 K, 500 MHz) for complex **Ru1C** (a) in CD_3_CN and (b) in toluene-*d*_8_. Equations depicting the presence of two solvated ions or an ion pair are described below each spectrum.

### Computational Studies

In order to shed light on the above-described effect in the supramolecular adducts in solution, we carried out density functional theory (DFT) studies at the PBE0-D3BJ/LANL2DZ//6-31G­(d,p)/PCM­(toluene)^81–86^ level of theory. An optimized structure of assembly (C_60_)_3_@Ru3C (being the host a dication) shows three well-defined molecular tweezers, formed by pairs of corannulene groups attached to the same bpy ligand, fixed by the central Ru­(II) atom with each tweezer embracing one fullerene molecule (Figure 5a). The structure also shows a negligible C–C≡C–C angle bending as a consequence of an excellent cavity size defined by the design. The octahedron around the central atom is slightly distorted due to additional weak interactions between nonadjacent groups (see below). Most of the fullerene surface is covered by the inner face of the corannulene subunits, guaranteeing strong dispersion interactions. These findings were also obtained for adducts (C_60_)_2_@**Ru2C** and**C_60_@Ru1C**(see the Supporting Information for more details). Noncovalent interaction (NCI) plots^87,88^ confirmed this by revealing extended weak (van der Waals) contacts between the outer surface of fullerene and the inner surface of corannulenes (Figure 5b). Counterpoise-corrected interaction energy^89,90^ between C_60_ and complex **Ru1C** was −35.84 kcal/mol, a value within the range of typical corannulene-substituted hosts.^91,92^ For higher hosts **Ru2C** and **Ru3C**, those energies rose up to −75.73 and −113.83 kcal/mol, which are ca. twice and three times the interaction energy for **C_60_@Ru1C**. These values indicate no improvement or decrease in binding abilities for a molecular tweezer upon recognition by any other tweezer within the same metal complex. The minor increase in interaction energy for hosts **Ru2C** and **Ru3C** with respect to **Ru1C** (approximately 2.1 kcal/mol) can be attributed to small interactions between nonadjacent corannulene/fullerene units (i.e., from different bpy ligands) as also revealed by NCI analysis (little isolated green regions in Figure 5b).

**Figure 5:**
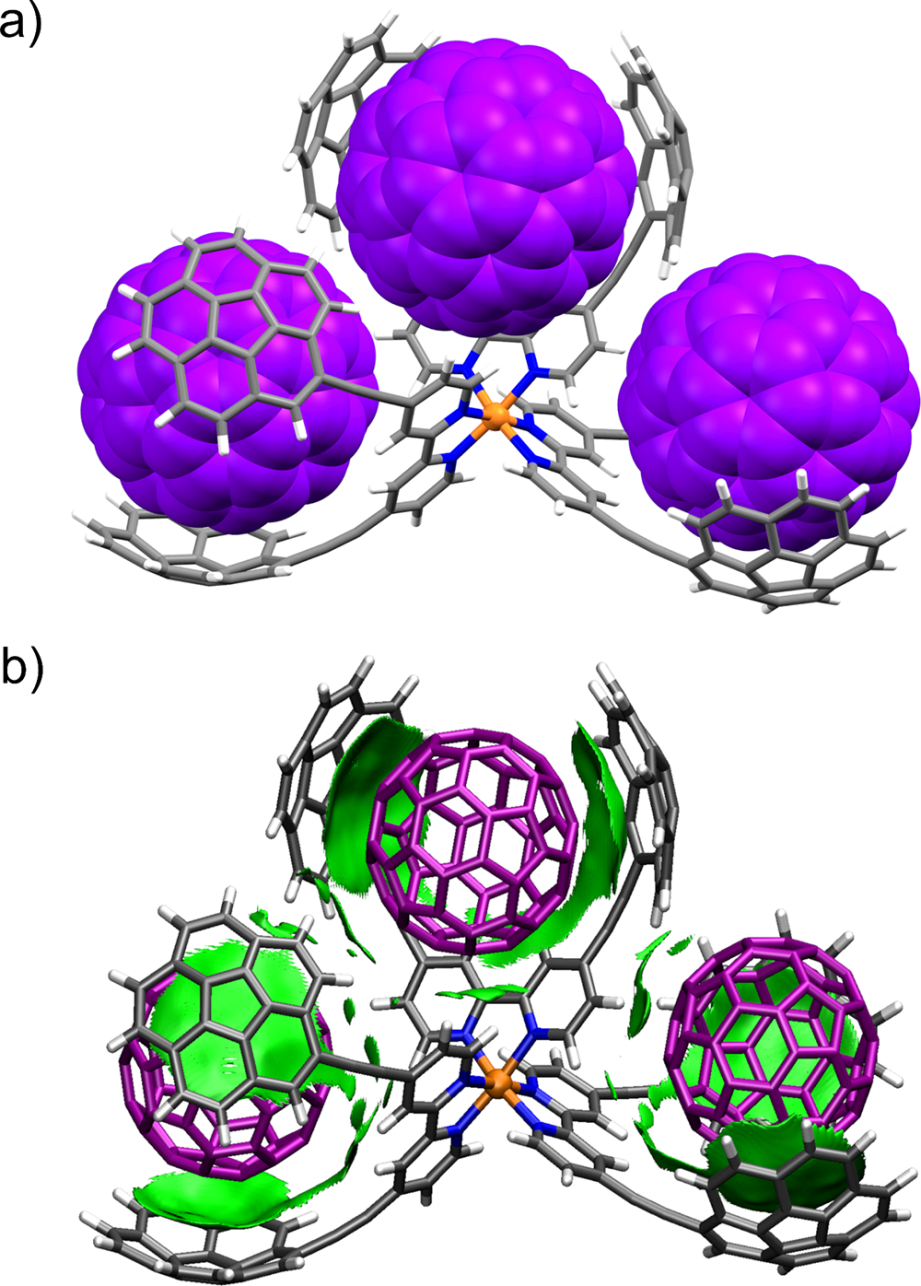
(a) DFT-optimized structure of the supramolecular adduct **(C_60_)_3_@Ru3C** at the PBE0-D3BJ/LANL2DZ//6-31G­(d,p)/PCM­(toluene) level of theory. Fullerene molecules are represented in the space-filling model. (b) Gradient isosurfaces (isovalue = 0.3 au) for the same assembly with densities within the interval −0.02 < ρ < 0 are exclusively shown to represent weak (vdW) interactions between the host and the guest molecules. Surfaces were colored in an RGB pattern with the following criteria: red indicates repulsion, green indicates weak attraction, and blue indicates strong attraction.

Theoretical calculations of the cationic complex and its interactions are not sufficient to account for the experimental behavior described above. The presence of two bulky BAr_4_^F^ anions could partially inhibit fullerene recognition by their mere presence next to the cation in the ion pair. However, if dispersion-based interactions between the anion and the relatively large surface of corannulene groups are strong enough, the supramolecular equilibrium changes and a new equilibrium arises, since tetrahedral BAr_4_^F^ competes for the cavity (Figure 6a). It is also worth noting that the radii of C_60_ and BAr_4_^F^ are similar (7.1 vs. 6.3 Å) and, consequently, the anion could fit in the cavity. In order to estimate the interaction strength between anion and cation, we carried out a systematic geometry optimization of different ion pairs for complex **Ru1C·(BAr_4_^F^)_2_** starting from the structure of a single crystal of parent complex [Ru­(bpy)_3_]·(BAr^F^)_2_. Anions were positioned in different locations around the central metal atom, and the system was allowed to relax until a minimum was found (see the Supporting Information for more details). The two most stable conformers are shown in Figure 6b, where the anions appear to interact attractively with both the inner and outer faces of the corannulene groups. NCI analysis reveals multiple weak contacts among these chemical species (Figure 6c). Overall, interaction energies between both BAr_4_^F^ anions and the Ru­(II) cation are highly negative (−190 kcal/mol on average), clearly indicating that the ion pair mainly interacts by strong electrostatic forces. Dissection of the contributions of each anion (i.e., the interaction of only one molecule of BAr_4_^F^ with the cation) revealed that an anion located near a corannulene group exhibited a slightly stronger interaction than one located elsewhere (estimated around 7 kcal/mol). This means that roughly 4% of the entire interaction energy between cation and anion is due to attractive dispersion forces established with the corannulene group. Albeit a relatively small contribution, this could be enough to allow the existence of a conformer where the anion is interacting with corannulene moieties in solution, thereby hindering the fullerene recognition process. Furthermore, the presence of three cavities in host **Ru3C** and only two BAr_4_^F^ anions could explain why the 1:3 (full) binding model yielded a high *K*_1_ at the expense of *K*_2_ and *K*_3_. The first binding event may proceed through a simple equilibrium, while the second and third bindings could involve competition with the two anions (Figure 6a). This scenario could also explain the observation of the extraordinarily negative cooperativity in the full model, as described above.

**Figure 6:**
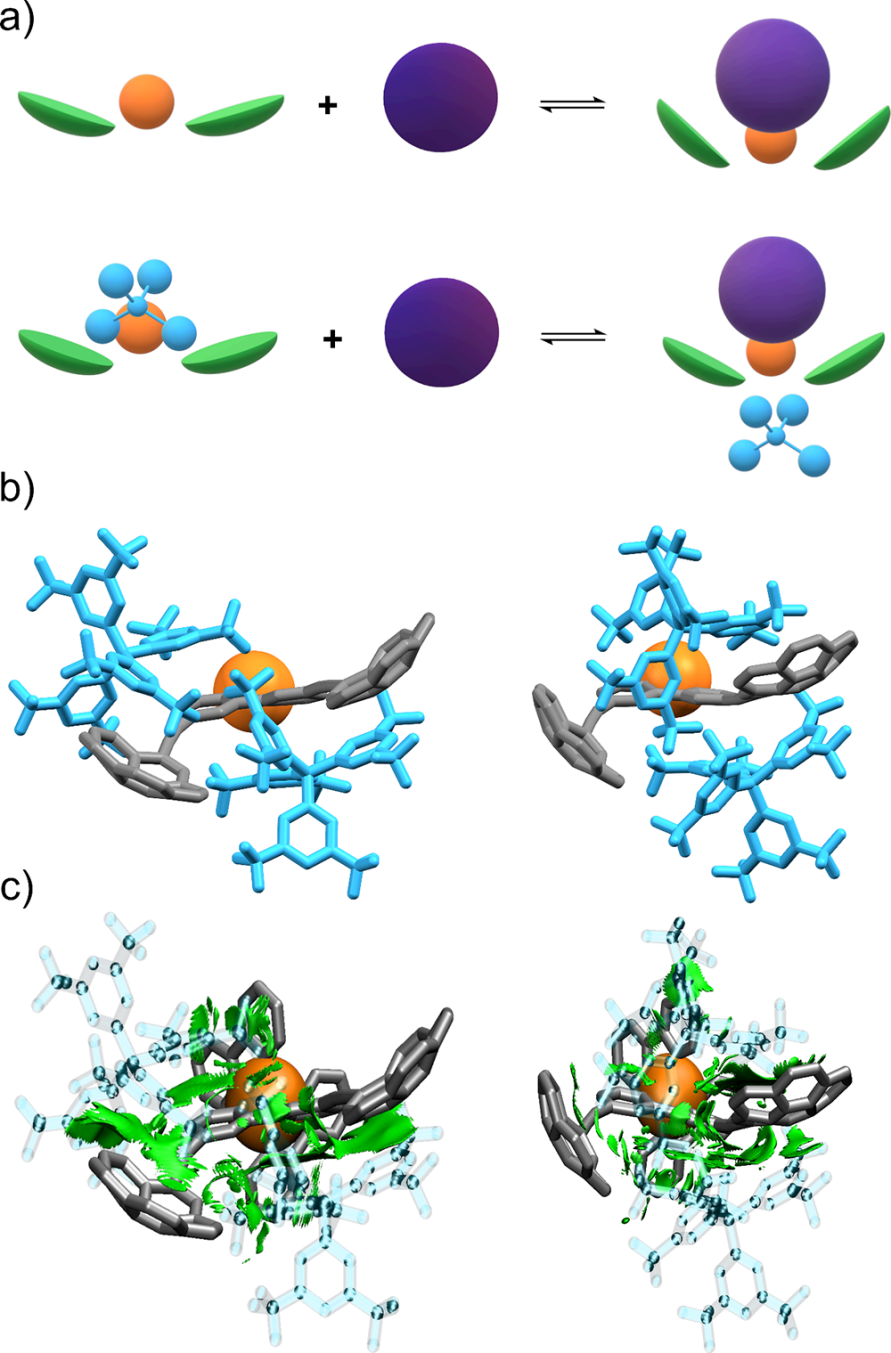
(a) Cartoon depiction of a simple supramolecular equilibrium between a single molecular tweezer bearing corannulene (green) and fullerene (purple), and a competitive equilibrium in the presence of BAr_4_^F^ counterion (blue). (b) Most stable DFT-optimized conformers of complex **Ru1C·(BAr^F^_4_)_2_.** (c) Gradient isosurfaces representing vdW interactions plotted with the same isovalue, cutoff, and RGB pattern as described in Figure 5. The Ru­(II) atom has been colored orange in all cases.

## Conclusions

A method to prepare a family of octahedral Ru­(II)–polypyridyl complexes bearing either planar (pyrene, **Ru1P–Ru3P**) and nonplanar (corannulene, **Ru1C–Ru3C**) aromatic groups have been developed. It relies on the successful synthesis of parent brominated compounds containing one, two, and three 4,4′-dibromo-2,2′-bipyridine ligands which were subjected to multiple Sonogashira C–C couplings with the corresponding arylethynyl ligand to furnish expected compounds. Another approach consisting of ligand prefunctionalization and further coordination to Ru­(II) failed due to a very poor conversion in the last step.

Resulting compounds exhibit fixed polycyclic aromatic hydrocarbons by pairs, thanks to the Ru­(II) metal center. They are also well-preorganized to establish a cavity for fullerene binding. Comprehensive supramolecular binding studies revealed that hosts **Ru1P–Ru3P** did not suffer any change upon guest (C_60_ or C_70_) addition in solution. Conversely, hosts **Ru1C–Ru3C** gave positive results in all cases, demonstrating the critical role of positive Gaussian curvature in facilitating concave–convex complementarity. We found that all cavities can accommodate guests C_60_ and C_70_, reaching the highest stoichiometries possible (up to 1:3) as their nonlinear regressions fitted reasonably well. This underscores the excellent contribution of the metal center by fixing all of the bpy ligands in the same molecule, offering ditopic and tritopic hosts for **Ru2C** and **Ru3C**, respectively. The most significant model corresponded to a noncooperative behavior, which suggests that there is no interaction among all cavities upon binding and, therefore, each molecular tweezer operates independently. However, the association constants were lower than anticipated yet remained comparable to those of a previously reported Cu­(I)-bpy complex.^38^ The most remarkable result was the lack of preference between two models when binding to C_70_ as both noncooperative and full flavors had the best statistical parameters. For the latter, it was found that the first stepwise constant was much higher than any other within the family (1.16 × 10^9^ vs. 1.31 × 10^4^ for the same K and host–guest couple but different flavor). All of these findings, with the assistance of computational studies, led us to consider the ability of the BAr_4_^F^ anion to hinder or partially inhibit fullerene binding in solution owing to the demonstrated existence of a strong ion pair in the solvent used for titration (toluene-*d*_*8*_). It has been ventured that the pair of anions could potentially interact with corannulene arms with a certain degree of strength, provoking a shift from a simple supramolecular equilibrium to competitive binding. If there is a mismatch between the number of cavities for supramolecular binding and anions (3 vs 2), the host–guest interaction might show distinct behavior between the first event and the other two.

## Supplementary Material



## Notes

Detailed experimental procedures, synthetic protocols, NMR, HRMS, UV–vis absorption and emission spectra, X-ray crystallographic data, association constants measurements, DOSY experiments, and computational calculation details (PDF)
