# Supplementary material for: Ru(II)-Based Multitopic Hosts for Fullerene Binding: Impact of the Anion in the Recognition Process
Source: Inorg Chem. 2025 Jan 30;64(5):2360–70. doi: 10.1021/acs.inorgchem.4c04608 (PMC12129259; doi:10.1021/acs.inorgchem.4c04608)
Supplement: Supplementary file 1 [file ic4c04608_si_001.pdf]

Ru(II)-based multitopic hosts for fullerene binding: impact of the anion in the recognition process

Adriana Sacristán-Martín,[a†] Nerea Álvarez-Llorente,[a†] Alberto Diez-Varga,[a] Héctor Barbero,[a*] Celedonio M. Álvarez[a*]

Table of contents

General Methods 2

Compound nomenclature 3

Synthetic procedures 4

NMR and HR-MS Spectra 15

UV/Vis Absorption and Fluorescence Studies 84

X-ray Crystallographic Tables 88

Association constants measurements 92

DOSY experiments 105

Computational Calculations Details 108

References 133

[a]GIR MIOMeT, IU CINQUIMA/Química Inorgánica, Facultad de Ciencias, Universidad de Valladolid, Valladolid, E47011, Spain. E-mail: hector.barbero@uva.es, celedonio.alvarez@uva.es
[†]A.S.-M. and N.A.-L. contributed equally to this work.

General Methods

Reagents were purchased from regular suppliers and used without further purification. 1-Bromocorannulene (Br-cora) was acquired from Synoi Chemicals (http://synoichemicals.uva.es/). Solvents were of analytical grade or spectrophotometric grade. They were either used as purchased or dried according to procedures described elsewhere.[1,2] Microwave reactions were carried out with an Anton Paar Monowave 300 Reactor using tightly capped flasks G10 (for volumes up to 10 mL) especially designed for the apparatus. All reactions under inert atmosphere (when needed) were performed with standard Schlenk techniques. They were also used as a preliminary step for degassing microwave flasks when inert atmosphere was necessary in microwave reactions. Column chromatography separations were carried out by using Silica gel 60 (particle size 0.040-0.063 mm; 230-400 mesh; Merck, Germany) as the stationary phase and TLCs were performed on precoated silica gel plates (0.25 mm thick, 60 F254, Merck, Germany) and observed under UV light. Purifications by centrifugation were performed in an Ortoalresa UNICEN centrifuge. NMR spectra were recorded on Agilent DD2 500 and Agilent MR 400 instruments. ^{1}H and $^{13}C\{^{1}H\}$ NMR chemical shifts are reported in parts per million (ppm) and are referenced to TMS, using residual solvent peak as an internal reference. A non-conventional numbering system has been used due to the complexity that arises from different symmetries. Coupling constants (J) are reported in hertz (Hz). Standard abbreviations used to indicate multiplicity: s = singlet, d = doublet, m = multiplet, dd = doublet of doublets. ^{1}H and ^{13}C assignments were performed by utilizing 2D NMR methods (gDQCOSY, ROESYAD, gHSQCAD, gHSQCAD-PS, gHMBCAD, band selective gHSQC, band selective gHMBC, gradient crisis HSQC, gradient crisis HMBC and c2HSQCse). DOSY experiments were performed using Bipolar Pulse Pair Stimulated Echo and ONESHOT sequences. High resolution mass spectra were recorded at mass spectrometry service of the Laboratory of Instrumental Techniques of the University of Valladolid (L.T.I., www.laboratoriotecnicasinstrumentales.es). A MALDI-TOF system (MALDI-TOF) Bruker Autoflex Speed (N_2 laser 337 nm, pulse energy 100 μJ, 1 ns), acceleration voltage 19 kV, reflector positive mode) was used. *Trans*-2-[3-(4-tert-butylphenyl)-2-methyl-2-propenylidene]malonitrile (DCTB) and 1,8-dihydroxy-9(10H)-anthracenone (dithranol) were used as matrixes. HRMS spectra were analyzed using Bruker DataAnalysis 4.1© (www.bruker.com). Steady state UV/Vis absorption spectroscopy was carried out on a Perkin Elmer Lambda 265 spectrophotometer, whereas emission spectroscopy was performed on a Cary Eclipse Fluorescence, using quartz cuvettes with a path length of 1.0 cm in acetonitrile as the solvent. Time-resolved fluorescence measurements were carried out using the single-photon counting technique with ns time resolution. A high repletion pulsed light source is used to excite the sample, and the photons emitted are processed using the TCC1 card in the computer. Fluorescence decays were obtained with the time-correlated single- photon counting (TCSPC) and MCP-PMT counter module (TCC2) of the FLS980 spectrometer (Edinburgh Instruments). The excitation source was a diode laser with excitation wavelengths 280 and 405 nm. EPLs-lasers produce picosecond duration pulses <1 ns at repetition rates up to 20 MHz (50 ns). The emission slit used was 4 nm. Fluorescence decays were analyzed with the method of nonlinear least squares iterative deconvolution and the quality of the fits was judged by the values of the reduced Chi-square (χ^2) and the autocorrelation function of the residuals using the FAST (Advanced Fluorescence Lifetime Analysis Software) program provided by the equipment. To measure the photoluminescence quantum yield (QY) the FLS980 fluorescence spectrometer is equipped with an integrating sphere. A rectangular 10 mm cuvette was used for the fluorescence measurements and spectrophotometric grade toluene as the solvent. All data were measured at 25 °C in the facilities of the Technological Scientific Park of the University of Burgos. Diffraction data were collected using an Oxford Diffraction Supernova diffractometer equipped with an Atlas CCD area detector and a four-circle kappa goniometer. For data collection, Mo or Cu micro-focused sources with multilayer optics were used. When necessary, crystals were mounted directly from solution using perfluorohydrocarbon oil to prevent atmospheric oxidation, hydrolysis and solvent loss. Data integration, scaling, and empirical absorption correction were performed using the CrysAlisPro software package. The structures were solved by direct methods and refined by full-matrix-least-squares against F^2 with SHELX in OLEX2. Non-hydrogen atoms were refined anisotropically, and hydrogen atoms were placed at idealized positions and refined using the riding model. Graphics were made using OLEX2 and MERCURY.

Compound nomenclature

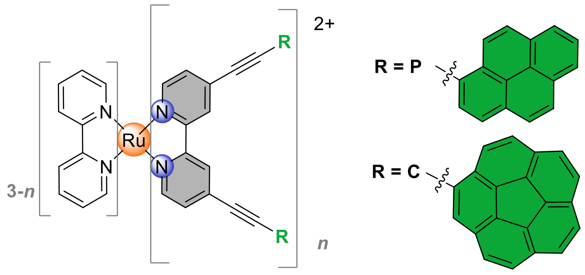

Ru*n*R

n/R	Pyrene		Corannulene	
1	Ru1P·PF_6	Ru1P·BAr^F_4	Ru1C·PF_6	Ru1C·BAr^F_4
2	Ru2P·PF_6	Ru2P·BAr^F_4	Ru2C·PF_6	Ru2C·BAr^F_4
3	Ru3P·PF_6	Ru3P·BAr^F_4	Ru3C·PF_6	Ru3C·BAr^F_4

Table S 1. Nomenclature of the different compounds prepared in this work. PF_6 and BAr^F_4 complexes are distinguished in this section due to their different properties and characterization. However, in the main paper only BAr^F_4 complexes are discussed, otherwise noted.

Synthetic procedures

Synthesis overview

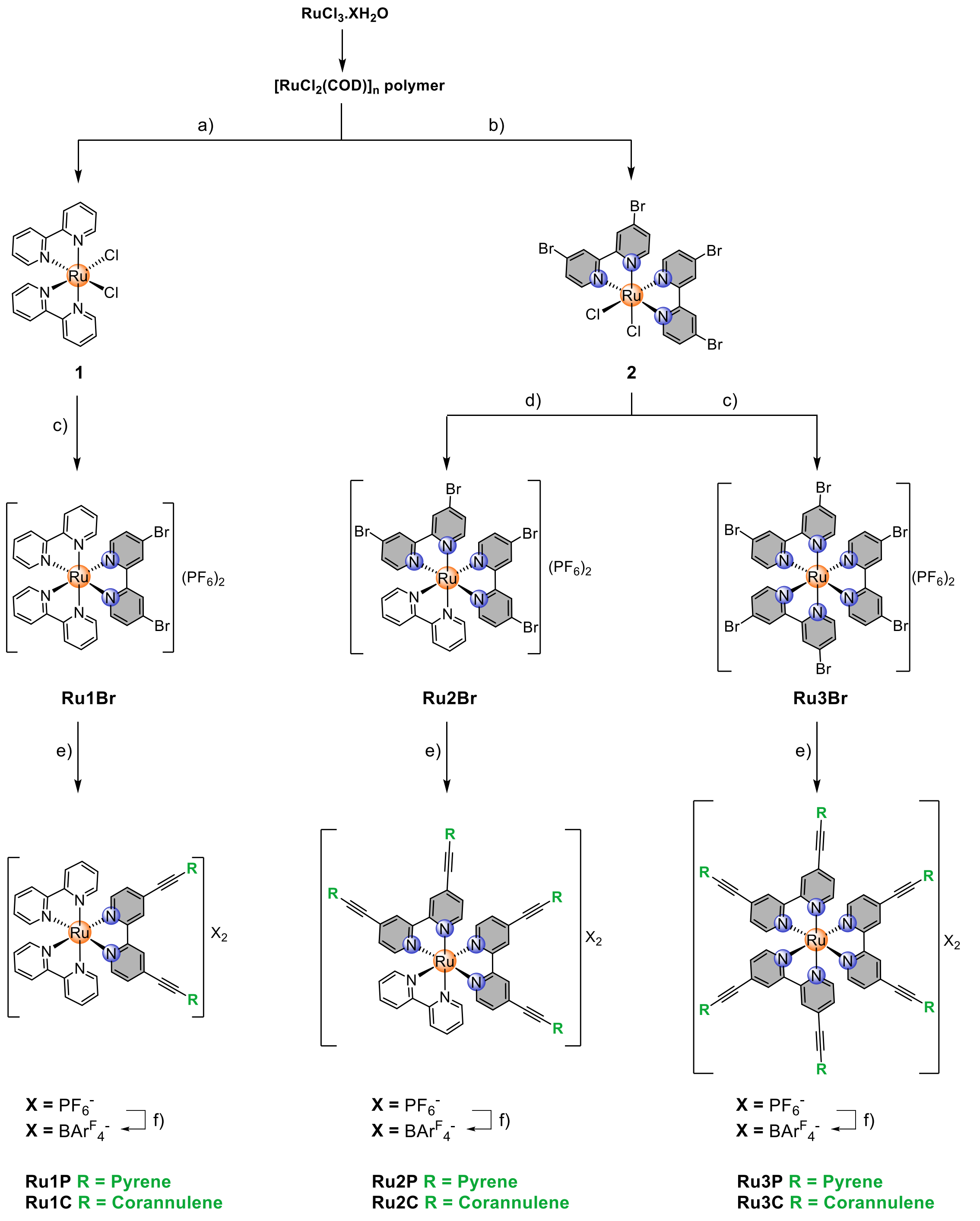

Scheme S 1. Synthesis routes to achieve final ruthenium complexes functionalized with polyaromatic hydrocarbons. Reagents and conditions: a) 2,2'-bipyridine, dichlorobenzene, 180 °C. b) 4-4'dibromo-2,2'-bipyridine, dichlorobenzene, 180 °C. c) i) 4-4'dibromo-2,2'-bipyridine, EtOH, reflux, ii) NH_4PF_6. d) 2,2'-bipyridine, EtOH, reflux, NH_4PF_6. e) PAH-acetylene, $[PdCl_2(dppf)]$, CuI, CH_3CN/NEt_3, THF, r.t. f) $Na(BAr^F{}_4)$, CH_2Cl_2, r.t.

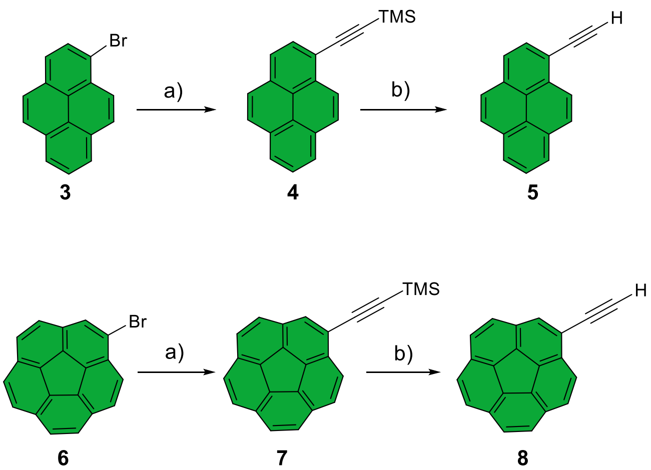

Scheme S 2. Synthesis of PAHs. Reagents and Conditions: a) trimethylsilyl acetylene, [$PdCl_2$(dppf)], CuCl, NEt_3, MW, 85 °C. b) KF·$2H_2O$, MeOH, THF, MW 50 °C.

[Ru(COD)Cl_2]$_n$ was prepared according to the reported procedure.[3] Compounds **4** and **7** were prepared according to the literature.[4]

<u>Trimethylsilylethynyl arene deprotection</u>

Trimethylsilylethynyl arene (0.15 mmol), KF·$2H_2O$ (18 mg, 0.19 mmol) were added to a microwave flask. The mixture was dissolved in a sealed microwave flask in THF/MeOH (2 mL / 2 mL) and irradiated in a microwave reaction at 50 °C for 30 min with stirring at 600 rpm. After finishing, the solvent was removed under vacuum and the resulting crude was dissolved in CH_2Cl_2 and washed with H_2O (3 x 10 mL). The organic layer was dried with anhydrous $MgSO_4$, filtered and the solvent removed in a rotary evaporator (33 mg, quantitative yield, 41 mg, quantitative yield for **5** and **8** respectively). The spectral data were in agreement with those reported in the literature.[5,6]

<u>*cis-[Ru(bpy)$_2$Cl$_2$]*</u>[7]

[$RuCl_2$(COD)]$_n$[3] (0.28 g, 1.0 mmol) and 2,2'-bipyridine (0.31 g, 2.0 mmol) were added to a Schlenk under nitrogen atmosphere. 20 mL of degassed *o*-dichlorobenzene was added to the solution and the mixture was heated at 180 °C under nitrogen atmosphere. After 2 h, it was cooled to room temperature and diethyl ether (40 mL) was added forming a dark precipitate. The solid was filtered and washed with diethyl ether. The resulting solid was used without further purification (0.42 g, 86%). The spectral data was in agreement with those reported in the literature.[8,9] ^{1}H NMR (500 MHz, Acetonitrile-*d*$_3$) δ 10.13 – 10.05 (m, 2H), 8.37 (dd, *J* = 8.0, 1.3 Hz, 2H), 8.24 – 8.19 (m, 2H), 7.99 (td, *J* = 7.8, 1.5 Hz, 2H), 7.68 (ddd, *J* = 7.4, 5.7, 1.3 Hz, 2H), 7.64 (d, *J* = 5.8 Hz, 2H), 7.60 (td, *J* = 7.8, 1.4 Hz, 2H), 6.99 (ddd, *J* = 7.2, 5.7, 1.4 Hz, 2H). HRMS (MALDI): m/z = 483.9778 [M^+] (calculated 483.9789 for $C_{20}H_{16}Cl_2N_4Ru$).

<u>*cis-[Ru(Br-bpy)$_2$Cl$_2$]*</u>

[$RuCl_2$(COD)]$_n$[3] (0.28 g, 1.0 mmol) and 4,4'-dibromo-2,2'-bipyridine (0.63 g, 2.0 mmol) were added to a Schlenk under nitrogen atmosphere. 20 mL of degassed *o*-dichlorobenzene was added to the solution and the mixture was heated at 180 °C under nitrogen atmosphere. After 2 h, it was cooled to room temperature and diethyl ether (40 mL) was added forming a dark precipitate. The solid was filtered and washed with diethyl ether. The resulting solid was used without further purification (0.76 g, 95%). ^{1}H NMR (500 MHz, Acetonitrile-*d*$_3$) δ 9.86 (d, *J* = 5.8 Hz, 2H), 8.61 (d, *J* = 2.0 Hz, 2H), 8.45 (d, *J* = 2.3 Hz, 2H), 7.87 (dd, *J* = 5.8, 2.3 Hz, 2H), 7.50 (d, *J* = 6.5 Hz, 2H), 7.21 (dd, *J* = 6.5, 2.0 Hz, 2H). HRMS (MALDI): m/z = 799.6179 [M^+] (calculated 799.6171 for $C_{20}H_{12}Br_4Cl_2N_4Ru$).

General method for [Ru(4,4'-dibromo-2,2'-bipyridine)$_n$ (2,2'-bipyridine)$_{3-n}$] $(PF_6)_2$ (n=1,2,3) preparation[7,10]

cis-[Ru(4,4'-dibromo-2,2'-bipyridine)(2,2'-bipyridine)$_2$] $(PF_6)_2$ (n=1) **Ru1Br·PF6**

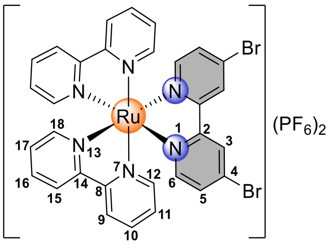

A solution of *cis*-[Ru(bipy)$_2$Cl$_2$] (0.24 g, 0.5 mmol) and 4,4'-dibromo-2,2'-bipyridine (0.16 g, 0.5 mmol) in EtOH (15 mL) was refluxed under nitrogen atmosphere for 24 h. The mixture was filtered through celite and then cooled down to room temperature. NH_4PF_6 (0.20 g, 1.2 mmol) was added producing the precipitation of a red solid. The dark red precipitate was filtered under reduced pressure, washed with ethanol (3 x 10 mL) and dried to give the pure compound **Ru1Br·PF6** as a red powder (0.46 g, 92%). ^{1}H NMR (500 MHz, Acetonitrile-*d*$_3$) δ 8.73 (d, *J* = 1.9 Hz, 2H, H^3), 8.49 (d, *J* = 7.7 Hz, 2H, H^9), 8.48 (d, *J* = 7.8 Hz, 2H, H^{15}) 8.07 (td, *J* = 7.7, 1.4 Hz, 2H, H^{10}), 8.05 (td, *J* = 7.8, 1.4 Hz, 2H, H^{16}) 7.77 – 7.74 (m, 2H, H^{12}), 7.69 – 7.64 (m, 2H, H^{18}), 7.59 (dd, *J* = 6.1, 1.9 Hz, 2H, H^5), 7.55 (d, *J* = 6.1 Hz, 2H, H^6), 7.43 – 7.40 (m, 2H, H^{11}), 7.40 - 7.36 (m, 2H, H^{17}). ^{13}C{^{1}H} NMR (101 MHz, Acetonitrile-*d*$_3$) δ 158.0 (C^2), 157.90 (C^8 or C^{14}), 157.86 (C^8 or C^{14}), 153.4 (C^6), 152.9 (C^{12}), 152.7 (C^{18}), 139.07 (C^{10} or C^{16}), 139.05 (C^{10} or C^{16}), 134.9 (C^4), 132.0 (C^5), 129.3 (C^3), 128.7 (C^{11} and C^{17}), 125.3 (C^9 and C^{15}). ^{19}F NMR (376 MHz, Acetonitrile-*d*$_3$) δ -73.0 (d, *J* = 706.0 Hz, PF_6^-). HRMS (MALDI): m/z = 872.8929 [Ru1Br + PF_6]$^+$ (calculated 872.8943 for $C_{30}H_{22}Br_2F_6N_6PRu$).

*cis- [Ru(*4,4'-dibromo-2,2'-bipyridine*)$_2$(2,2'-bipyridine)$_1$] $(PF_6)_2$ (n=2)* **Ru2Br·PF6**

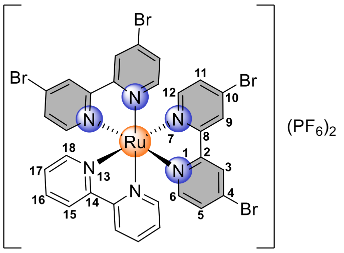

A solution of *cis*-[Ru(4,4'-dibromo-2,2'-bipyridine)$_2$Cl$_2$] (0.32 g, 0.4 mmol) and 2,2'-bipyridine (62 mg, 0.4 mmol) in EtOH (5 mL) was refluxed under nitrogen atmosphere for 24 h. The mixture was filtered through celite and then cooled down to room temperature. NH_4PF_6 (0.14 g, 0.84 mmol) was added producing the precipitation of a red solid. The dark red precipitate was filtered under reduced pressure, washed with ethanol (3 x 10 mL) and dried to give the pure compound **Ru2Br·PF6** as a red powder (0.31 g, 65%). ^{1}H NMR (500 MHz, Acetonitrile-*d*$_3$) δ 8.76 – 8.74 (m, 4H, H^3 and H^9), 8.50 (d, *J* = 8.2 Hz, 2H, H^{15}), 8.09 (td, *J* = 8.2, 1.5 Hz, 2H, H^{16}), 7.73 (d, *J* = 5.6 fHz, 2H, H^{18}), 7.64 (dd, *J* = 6.1, 2.0 Hz, 2H, H^{11}), 7.62 – 7.57 (m, 4H, H^5, H^{12}), 7.51 (d, *J* = 6.1 Hz, 2H, H^6), 7.48 – 7.37 (m, 2H, H^{17}). ^{13}C{^{1}H} NMR (101 MHz, Acetonitrile-*d*$_3$) δ 156.92 (C^2), 156.88 (C^8), 156.8 (C^{14}), 152.6 (C^{12}), 152.4 (C^6), 152.0 (C^{18}), 138.3 (C^{16}), 134.23 (C^4 or C^{10}), 134.19 (C^4 or C^{10}), 131.08 (C^5 or C^{11}), 131.05 (C^5 or C^{11}), 128.3 (C^3 and C^9), 127.7 (C^{17}), 124.4 (C^{15}). ^{19}F NMR (376 MHz, Acetonitrile-*d*$_3$) δ -73.0 (d, *J* = 705.9 Hz, PF_6^-). HRMS (MALDI): m/z = 1026.7107 [Ru2Br + PF_6]$^+$ (calculated 1026.7155 for $C_{30}H_{22}Br_4F_6N_6PRu$).

*cis-[Ru (*4,4'-dibromo-2,2'-bipyridine*)$_3$] $(PF_6)_2$ (n=3)* **Ru3Br·PF6**

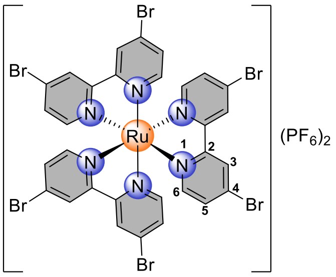

A solution of [Ru(4,4'-dibromo-2,2'-bipyridine)$_2$Cl$_2$] (0.3 g, 0.4 mmol) and 4,4'-dibromo-2,2'-bipyridine (0.12 g, 0.4 mmol) in EtOH (15 mL) was refluxed under nitrogen atmosphere for 24 h. The mixture was filtered through celite and then cooled down to room temperature. NH_4PF_6 (0.14 g, 0.84 mmol) was added producing the precipitation of

an orange solid. The orange precipitate was filtered under reduced pressure, washed with ethanol (3 x 10 mL) and dried to give the pure compound **Ru3Br·PF$_6$** as an orange powder (0.33 g, 62%). ^{1}H NMR (500 MHz, Acetonitrile-*d*$_3$) δ 8.74 (d, *J* = 2.0 Hz, 6H, H^3), 7.63 (dd, *J* = 6.1, 2.0 Hz, 6H, H^5), 7.55 (d, *J* = 6.1 Hz, 6H H^6). ^{13}C{^{1}H} NMR (101 MHz, Acetonitrile-*d*$_3$) δ 156.8 (C^2), 152.6 (C^6), 134.5 (C^4), 131.1 (C^5), 128.4 (C^3).^{19}F NMR (376 MHz, Acetonitrile-*d*$_3$) δ -73.0 (d, *J* = 706.3 Hz, PF_6^-). HRMS (MALDI): m/z = 1181.5332 [Ru3Br + PF$_6$]$^+$ (calculated 1181.5379 for $C_{30}H_{18}Br_6F_6N_6PRu$).

Sonogashira C-C coupling:[11]

Ru1P·PF$_6$

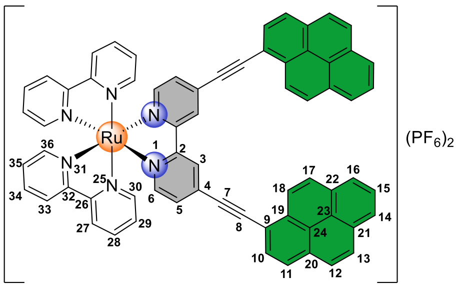

Ru1Br·PF$_6$ (70 mg, 0.07 mmol), [PdCl$_2$(dppf)] (6.8 mg, 6.5 µmol), and CuI (6 mg 20 µmol) were added to a Schlenk flask under nitrogen atmosphere. The Schlenk was purged with nitrogen several times. A mixture of CH_3CN/NEt_3 (2.2 mL /1.1 mL) was added and degassed again. After 10 min of stirring, a solution of **5** (47 mg, 0.21 mmol) in 4.0 mL of anhydrous THF was added dropwise to the reddish solution. The color of the solution shifted to darker red. The mixture was stirred for 1 hour under nitrogen atmosphere. Then, 10 mL of hexane were added to the mixture giving a red precipitate. The mixture was centrifuged, the resulting solid was washed with hexane (3 x 10 mL) and dried in the oven at 80 °C giving the expected compound as a dark red solid (46 mg, 50%). ^{1}H NMR (500 MHz, Acetonitrile-*d*$_3$) δ 8.99 (d, *J* = 1.7 Hz, 2H, H^3), 8.81 (d, *J* = 9.1 Hz, 2H, H^{18}), 8.55 (d, *J* = 8.2 Hz, 4H, H^{27} and H^{33}), 8.43 – 8.41 (m, 4H, H^{16} and H^{17}), 8.39 (d, *J* = 7.7 Hz, 2H, H^{14}), 8.37 (d, *J* = 8.0 Hz, 2H, H^{10}), 8.34 (d, *J* = 8.0 Hz, 2H, H^{11}), 8.30 (d, *J* = 9.0 Hz, 2H, H^{13}), 8.23 (d, *J* = 9.0 Hz, 2H, H^{12}), 8.17 (t, *J* = 7.7 Hz, 2H, H^{15}), 8.14 – 8.08 (m, 4H, H^{34} and H^{28}), 7.90 (d, *J* = 5.5 Hz, 2H, H^{30}), 7.83 (d, *J* = 5.9 Hz, 2H, H^6), 7.77 (d, *J* = 5.5 Hz, 2H, H^{36}), 7.69 (dd, *J* = 5.9, 1.7 Hz, 2H, H^5), 7.51 – 7.47 (m, 2H, H^{29}), 7.47 – 7.43 (m, 2H, H^{35}). ^{13}C{^{1}H} NMR (101 MHz, Acetonitrile-*d*$_3$) δ 157.1 (C^2), 157.0 (C^{26}), 156.9 (C^{32}), 151.9 (C^{30}), 151.8 (C^6), 151.7 (C^{36}), 138.10 (C^{28}), 138.07 (C^{34}),132.8 (C^{20}), 132.6 (C^4), 132.5 (C^{19}), 131.2 (C^{21}), 130.9 (C^{22}), 130.4 (C^{10}), 129.5 (C^{17}), 129.4 (C^{13}), 128.7 (C^5), 127.7 (C^{29} and C^{35}), 127.3 (C^{12}), 127.0 (C^{15}), 126.6 (C^{14}), 126.5 (C^{16}), 126.3 (C^3), 125.0 (C^{11}), 124.8 (C^{18}), 124.4 (C^{27} and C^{33}), 124.2 (C^{24}), 123.7 (C^{23}-in), 115.1 (C^9), 97.2 (C^8), 91.3 (C^7). ^{19}F NMR (376 MHz, Acetonitrile-*d*$_3$) δ -73.0 (d, *J* = 706.4 Hz, PF_6^-). HRMS (MALDI): m/z = 1163.2057 [Ru1P + PF$_6$]$^+$ (calculated 1163.2012 for $C_{66}H_{40}F_6N_6PRu$).

Ru2P·PF$_6$

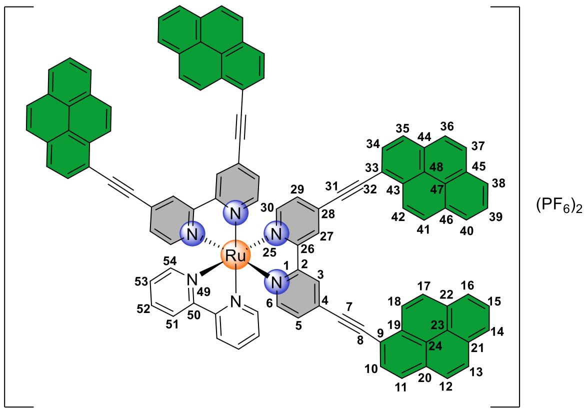

Ru2Br·PF$_6$ (70 mg, 0.06 mmol), [PdCl$_2$(dppf)] (4.3 mg, 6 µmol), and CuI (3.4 mg 18 µmol) were added to a Schlenk flask under nitrogen atmosphere. The Schlenk was purged with nitrogen several times. A mixture of CH_3CN/NEt_3 (2.0 mL /1.1 mL) was added and degassed again. After 10 min of stirring, a solution of **5** (67 mg, 0.3 mmol) in 5 mL of anhydrous THF was added dropwise to the reddish solution shifting in color to darker red. It was stirred for 1 hour under nitrogen atmosphere. Then, 10 mL of hexane were added to the mixture giving an orange precipitate. The mixture was centrifuged, the resulting solid was washed with hexane (3 x 10 mL) and dried in the oven at 80 °C giving the expected compound as an orange solid (69 mg, 65%). ^{1}H NMR (500 MHz, Acetonitrile-*d*$_3$) δ 9.04 (s, 4H, H^3, H^{27}), 8.84 (d, *J* = 9.0 Hz, 2H, H^{18}), 8.82 (d, *J* = 8.7 Hz, 2H, H^{42}), 8.62 (d, *J* = 8.4 Hz, 2H, H^{51}), 8.45 (d, *J* =

9.0 Hz, 2H, H^{17} or H^{41}), 8.43 – 8.33 (m, 18H, H^{16}, H^{17} or H^{41}, H^{14}, H^{40}, H^{10}, H^{34}, H^{38}, H^{11}, H^{35}), 8.31 (d, *J* = 9.0 Hz, 2H, H^{13}), 8.29 (d, *J* = 8.9 Hz, 2H, H^{37}), 8.24 (d, *J* = 9.0 Hz, 2H, H^{12}), 8.22 (d, *J* = 8.9 Hz, 2H, H^{36}), 8.20 – 8.13 (m, 6H, H^{15}, H^{52}, H^{39}), 8.01 (d, *J* = 5.8 Hz, 2H, H^{30}), 7.96 (d, *J* = 6.8 Hz, 2H, H^{54}), 7.88 (d, *J* = 5.8 Hz, 2H, H^{6}), 7.78 (dd, *J* = 5.8, 1.3 Hz, 2H, H^{29}), 7.75 (dd, *J* = 5.8, 1.5 Hz, 2H, H^{5}), 7.55 (t, *J* = 6.8 Hz, 2H, H^{53}). ^{13}C{^{1}H} NMR (126 MHz, Acetonitrile-*d*$_3$) δ 158.00 (C^{26}), 157.95 (C^{2}), 157.8 (C^{50}), 153.0 (C^{30}), 152.9 (C^{54}), 152.8 (C^{6}), 139.3 (C^{52}), 133.84 (C^{20} or C^{44}), 133.81 (C^{20} or C^{44}), 133.77 (C^{4} or C^{28}), 133.75 (C^{4} or C^{28}), 133.49 (C^{19} or C^{43}), 133.48 (C^{19} or C^{43}), 132.2 (C^{21} or C^{45}), 132.1 (C^{21} or C^{45}), 131.88 (C^{22} or C^{46}), 131.86 (C^{22} or C^{46}), 131.37 (C^{10} or C^{34}), 131.36 (C^{10} or C^{34}), 130.49 (C^{17} or C^{41}), 130.47 (C^{17} or C^{41}), 130.43 (C^{13} or C^{37}), 130.41 (C^{13} or C^{37}), 129.8 (C^{5} and C^{29}), 128.9 (C^{53}), 128.22 (C^{12} or C^{36}), 128.20 (C^{12} or C^{36}), 127.99 (C^{15} or C^{39}), 127.96 (C^{15} or C^{39}), 127.59 (C^{14} or C^{38}), 127.55 (C^{14} or C^{38}), 127.51 (C^{16} or C^{40}), 127.48 (C^{16} or C^{40}), 127.4 (C^{3} and C^{27}), 126.00 (C^{11} or C^{35}), 125.97 (C^{11} or C^{35}), 125.83 (C^{18} or C^{42}), 125.81 (C^{18} or C^{42}), 125.5 (C^{51}), 125.14 (C^{24} or C^{48}), 125.11 (C^{24} or C^{48}), 124.8 (C^{23} or C^{47}), 124.7 (C^{23} or C^{47}), 116.1 (C^{9} and C^{33}), 98.3 (C^{8} and C^{32}), 92.3 (C^{7} and C^{31}). ^{19}F NMR (470 MHz, Acetonitrile-*d*$_3$) δ -73.0 (d, *J* = 705.7 Hz, PF_6^-). HRMS (MALDI): m/z = 1611.3270 [Ru2P + PF_6]$^+$ (calculated 1611.3274 for $C_{102}H_{56}F_6N_6PRu$).

Ru3P·PF$_6$

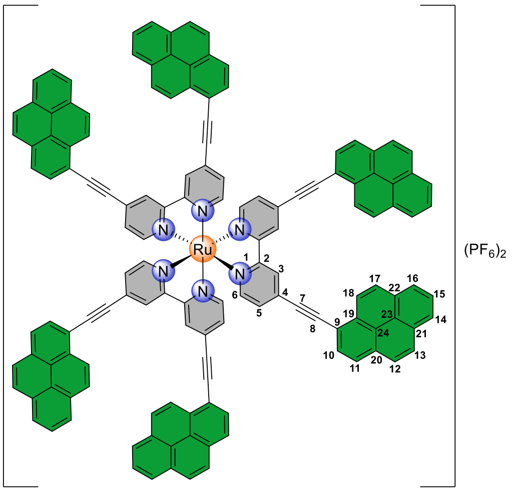

Ru3Br·PF$_6$ (70 mg, 0.05 mmol), [PdCl$_2$(dppf)] (3.8 mg, 5 µmol), and CuI (3.0 mg 16 µmol) were added to a Schlenk under nitrogen atmosphere. The Schlenk was purged with nitrogen several times. A mixture of CH_3CN/NEt_3 (1.8 mL /1.0 mL) was added and degassed again. After 10 min of stirring, a solution of **5** (84 mg, 0.4 mmol) in 5 mL of anhydrous THF was added dropwise to the reddish solution shifting in color to darker red. It was stirred for 1 h under nitrogen atmosphere. Then, 10 mL of hexane were added to the mixture giving an orange precipitate. The mixture was centrifuged, the resulting solid was washed with hexane (3 x 10 mL) and dried in the oven at 80 °C giving the expected compound as an orange solid (95 mg, 86%). ^{1}H NMR (500 MHz, DMSO-*d*$_6$) δ 9.59 (s, 6H, H^{3}), 8.78 (d, *J* = 9.2 Hz, 6H, H^{18}), 8.49 (d, *J* = 9.2 Hz, 6H, H^{17}), 8.47 (d, *J* = 7.7 Hz, 6H, H^{16}), 8.45 (d, *J* = 7.7 Hz, 6H, H^{14}), 8.43 (s, 12H, H^{10} and H^{11}), 8.37 (d, *J* = 8.9 Hz, 6H, H^{13}), 8.30 (d, *J* = 8.9 Hz, 6H, H^{12}), 8.19 (t, *J* = 7.7 Hz, 6H, H^{15}), 8.16 (d, *J* = 6.0 Hz, 6H, H^{6}), 8.01 (d, *J* = 6.0 Hz, 6H, H^{5}). ^{13}C{^{1}H} NMR (126 MHz, DMSO-*d*$_6$) δ 156.7 (C^{2}), 151.8 (C^{6}), 132.2 (C^{19}), 132.1 (C^{4}), 131.8 (C^{22}), 130.7 (C^{20}), 130.4 (C^{21}), 130.3 (C^{10}), 129.6 (C^{17}), 129.44 (C^{5}), 129.41 (C^{13}), 127.3 (C^{12}), 127.1 (C^{15}), 126.8 (C^{3}), 126.7 (C^{14}), 126.6 (C^{16}), 125.2 (C^{11}), 124.7 (C^{18}), 123.6 (C^{23}), 123.2 (C^{24}), 114.7 (C^{9}), 96.8 (C^{8}), 91.9 (C^{7}). ^{19}F NMR (470 MHz, DMSO-*d*$_6$) δ -70.2 (d, *J* = 711.5 Hz) (PF_6^-). HRMS (MALDI): m/z = 2059.4516 [Ru3P + PF_6]$^+$ (calculated 2059.4536 for $C_{138}H_{72}F_6N_6PRu$).

Ru1C·PF$_6$

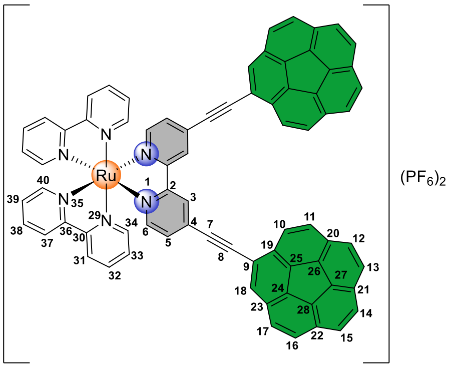

Ru1Br·PF$_6$ (20 mg, 0.02 mmol), [PdCl$_2$(dppf)] (1.4 mg, 2 µmol), and CuI (1.1 mg, 6 µmol) were added to a Schlenk flask under nitrogen atmosphere. The Schlenk was purged with nitrogen several times. A mixture of CH_3CN/NEt_3 (500 µL /200 µL) was added and degassed again. After 10 min of stirring, a solution of **8** (16 mg, 0.06 mmol) in 1 mL of anhydrous THF was added dropwise to the reddish solution shifting in color to darker red. It was stirred for 1 h under nitrogen atmosphere. Then, 5 mL of hexane were added to the mixture giving an orange precipitate. The mixture was centrifuged, the resulting solid was washed with hexane (3 x 5 mL) and dried in the oven at 80 ° C, giving the expected compound as an orange solid (27 mg, 95%). ^{1}H NMR (500 MHz, Acetonitrile-d_3) δ 8.95 (d, J = 1.5 Hz, 2H, H^{3}), 8.55 (d, J = 8.1 Hz, 4H, H^{31} and H^{37}), 8.33 (s, 2H, H^{18}), 8.31 (d, J = 8.8 Hz, 2H, H^{10}), 8.13 – 8.08 (m, 6H, H^{32}, H^{38} and H^{11}), 8.01 – 7.93 (m, 12H, H^{12}, H^{13}, H^{14}, H^{15}, H^{16} and H^{17}), 7.86 (d, J = 5.6 Hz, 2H, H^{34}), 7.82 (d, J = 5.8 Hz, 2H, Hz^{6}), 7.75 (d, J = 5.6 Hz, 2H, H^{40}), 7.64 (dd, J = 5.8, 1.5 Hz, 2H, H^{5}), 7.49 – 7.42 (m, 4H, H^{33} and H^{39}). ^{13}C{^{1}H} NMR (126 MHz, Acetonitrile-d_3) δ 158.0 (C^{2}), 157.9 (C^{30}), 157.8 (C^{36}), 152.9 (C^{34}), 152.8 (C^{6}), 152.6 (C^{40}), 139.1 (C^{32} or C^{38}), 139.0 (C^{32} or C^{38}), 136.8 (C^{27} or C^{28}), 136.6 (C^{24}), 136.3 (C^{26}), 136.1 (C^{27} or C^{28}), 135.7 (C^{25}), 134.4 (C^{18}), 133.2 (C^{4}), 132.9 (C^{21}, C^{22} or C^{23}), 132.5 (C^{20}), 132.3 (C^{21}, C^{22} or C^{23}), 131.4 (C^{19}), 131.0 (C^{21}, C^{22} or C^{23}), 129.9 (C^{5}), 129.5 (C^{11}), 129.2 (C^{Hcor}), 129.1 (C^{Hcor}), 129.0 (C^{Hcor}), 128.7 (C^{33} and C^{39}), 128.6 (C^{Hcor}), 128.5 (C^{Hcor}), 128.0 (C^{17}), 127.4 (C^{3}), 126.6 (C^{10}), 125.4 (C^{31} and C^{37}), 120.0 (C^{9}), 96.9 (C^{8}), 90.1 (C^{7}). ^{19}F NMR (470 MHz, Acetonitrile-d_3) δ -72.97 (d, J = 706.2 Hz) (PF_6^-). HRMS (MALDI): m/z = 1259.1997 [Ru1C + PF_6]$^+$ (calculated 1259.2014 for $C_{74}H_{40}F_6N_6PRu$).

Ru2C·PF$_6$

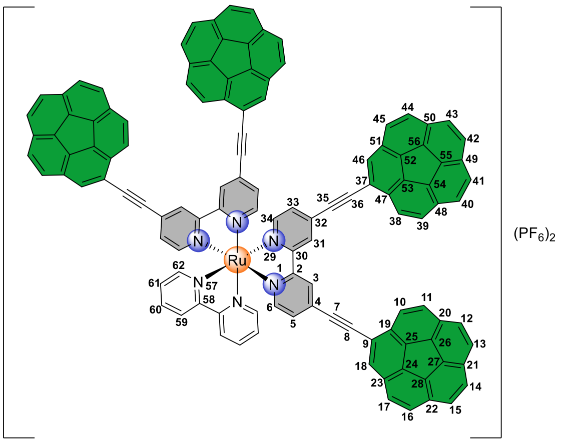

Ru2Br·PF$_6$ (20 mg, 0.02 mmol), [PdCl$_2$(dppf)] (1.4 mg, 2 µmol), and CuI (1.1 mg, 6 µmol) were added to a Schlenk flask under nitrogen atmosphere. The Schlenk was purged with nitrogen several times. A mixture of CH_3CN/NEt_3 (500 µL /200 µL) was added and degassed again. After 10 min of stirring, a solution of **8** (23 mg, 0.09 mmol) in 2 mL of anhydrous THF was added dropwise to the reddish solution shifting in color to darker red. It was stirred for 1 h under nitrogen atmosphere. Then, 5 mL of hexane were added to the mixture giving a red precipitate. The mixture was centrifuged, the resulting solid was washed with hexane (3 x 5 mL) and dried in the oven at 80 ° C, giving the expected compound as a red solid (31 mg, 93%). ^{1}H NMR (500 MHz, DMSO-d_6) δ 9.45 (s, 4H, H^{3} and H^{31}), 8.94 (d, J = 8.6 Hz, 2H, H^{59}), 8.45 (s, 2H, H^{18} or H^{46}), 8.44 (s, 2H, H^{18} or H^{46}), 8.33 – 8.29 (m, 4H, H^{10} and H^{38}), 8.27 (d, J = 8.6 Hz, 2H, H^{60}), 8.18 (d, J = 8.9 Hz, 2H, H^{11} or H^{39}), 8.16 (d, J = 8.8 Hz, 2H, H^{11} or H^{39}), 8.08 – 8.02 (m, 26H, H^{12}, H^{13}, H^{14}, H^{15}, H^{16}, H^{17}, H^{40}, H^{41}, H^{42}, H^{43}, H^{44}, H^{45} and H^{34}), 7.97 (d, J = 4.7 Hz, 2H, H^{62}), 7.89 – 7.86 (m, 6H, H^{5}, H^{6} and H^{33}), 7.67 – 7.62 (m, 2H, H^{61}). ^{13}C{^{1}H} NMR (126 MHz, DMSO-d_6) δ 156.8 (C^{2}, C^{30}, C^{58}), 151.8 (C^{34}-in), 151.7 (C^{62}-in), 151.0 (C^{6}-in), 138.5 (C^{60}-in), 135.4 (2C_q^{cor}), 135.1 (C^{24} and C^{52}), 134.9 (C^{26} and C^{54}), 134.7 (2C_q^{cor}), 134.3 (C^{25} and C^{53}), 133.3 (C^{18} and C^{46}), 131.7 (2C_q^{cor}), 131.4 (2C_q^{cor}), 131.3 (C^{20} and C^{48}), 131.1 (C^{4} and C^{32}), 130.0 (C^{19} and C^{47}), 129.9 (2C_q^{cor}), 129.5 (C^{5} and C^{33}), 128.8 (C^{11} and C^{39}), 128.5 (2C^{Hcor}), 128.4 (2C^{Hcor}), 128.3 (2C^{Hcor}), 128.2 (C^{61}), 127.9 (2C^{Hcor}), 127.7 (C^{12} and C^{40}), 127.3 (C^{17} and C^{45}), 126.8 (C^{3} and C^{31}), 125.6 (C^{10} and C^{38}), 124.6 (C^{59}), 118.6 (C^{9} and C^{37}), 95.5 (C^{8} and C^{36}), 89.8 (C^{7} and C^{35}). ^{19}F NMR (470 MHz, DMSO-d_6) δ -70.16 (d, J = 711.2 Hz) (PF_6^-). HRMS (MALDI): m/z = 1803.325 [Ru2C + PF_6]$^+$ (calculated 1803.3279 for $C_{118}H_{56}F_6N_6PRu$).

Ru3C·PF₆

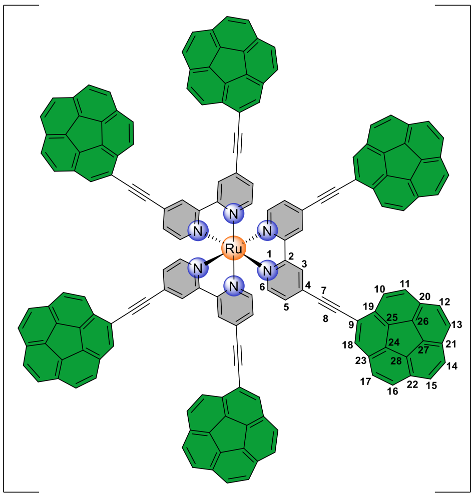

Ru3Br·PF₆ (20 mg, 0.02 mmol), [PdCl$_2$(dppf)] (1.4 mg, 2 μmol), and CuI (1.1 mg, 6 μmol) were added to a Schlenk flask under nitrogen atmosphere. The Schlenk was purged with nitrogen several times. A mixture of CH_3CN/NEt_3 (500 μL /200 μL) was added and degassed again. After 10 min of stirring, a solution of **8** (44 mg, 0.16 mmol) in 3 mL of anhydrous THF was added dropwise to the reddish solution shifting in color to darker red. It was stirred for 1 h under nitrogen atmosphere. Then, 5 mL of hexane were added to the mixture giving a red precipitate. The mixture was centrifuged, the resulting solid was washed with hexane (3 x 5 mL) and dried in the oven at 80 °C, giving the expected compound as a red solid (43 mg, 87%). ^{1}H NMR (500 MHz, DMSO-d_6) δ 9.50 (s, 6H, H^3), 8.46 (s, 6H, H^{18}), 8.33 (d, J = 8.8 Hz, 6H, H^{10}), 8.18 (d, J = 8.8 Hz, 6H, H^{11}), 8.09 (d, J = 6.0 Hz, 6H, H^6), 8.07 – 7.98 (m, 36H, H^{12}, H^{13}, H^{14}, H^{15}, H^{16} and H^{17}), 7.92 (dd, J = 6.0, 1.7 Hz, 6H, H^5). ^{13}C{^{1}H} NMR (126 MHz, DMSO-d_6) δ 156.6 (C^2), 151.8 (C^6), 135.4 (C_q^{cor}), 135.1 (C^{24}), 135.0 (C^{26}), 134.7 (C_q^{cor}), 134.3 (C^{25}), 133.4 (C^{18}), 131.74 (C^4), 131.69 (C_q^{cor}), 131.3 (C^{20}), 131.1 (C_q^{cor}), 130.1 (C^{19}), 129.9 (C_q^{cor}), 129.6 (C^5-in), 128.8 (C^{11}), 128.5 (C^{Hcor}), 128.4 (C^{Hcor}), 128.3 (C^{Hcor}), 127.9 (C^{Hcor}), 127.7 (C^{12}), 127.3 (C^{17}), 126.9 (C^3), 125.7 (C^{10}), 118.6 (C^9), 95.6 (C^8), 89.9 (C^7). ^{19}F NMR (470 MHz, DMSO-d_6) δ -70.17 (d, J = 710.8 Hz) (PF_6^-). HRMS (MALDI): m/z = 2347.4551 [Ru3C + PF$_6$]$^+$ (calculated 2347.4542 for $C_{162}H_{72}F_6N_6PRu$).

General method for anion exchange

The corresponding Ru(II) bipyridyl complex and Na(BAr^F$_4$) were dissolved in CH_2Cl_2 (5 mL) and stirred at r.t. for 15 min. Then, the crude was washed with H_2O (2 x 5 mL), the organic layer separated, dried with anhydrous $MgSO_4$, filtered and concentrated at the rotary evaporator. To the resulting solution, *n*-hexane was added, giving an orange or red precipitate that was isolated by centrifugation and dried under vacuum.

Ru(bpy)₃·BAr^{F}₄[12]

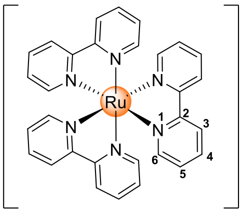

General method was followed using *tris*(2,2'-bipyridine)ruthenium(II) hexafluorophosphate (50 mg, 0.06 mmol) and Na(BAr^F$_4$) (103 mg, 0.12 mmol). **Ru(bpy)₃· BAr^{F}₄** was isolated as an orange solid (120 mg, 90%). ^{1}H NMR (500 MHz, Acetonitrile-d_3) δ 8.49 (d, J = 8.0 Hz, 6H, H^3), 8.05 (td, J = 8.0, 1.5 Hz, 6H, H^4), 7.73 – 7.71 (m, 6H, H^6), 7.70 – 7.64 (m, 48H, H^{BArF4}), 7.40 – 7.36 (m, 6H, H^5). ^{13}C{^{1}H} NMR (101 MHz, Acetonitrile-d_3) δ 163.3 (C^{BArF4}), 161.8 (C^{BArF4}), 157.9 (C^2), 152.6 (C^6), 138.8 (C^4), 135.6 (C^{BArF4}), 128.5 (C^5), 126.8 (C^{BArF4}), 125.2 (C^3), 124.1 (C^{BArF4}). ^{19}F NMR (471 MHz, Acetonitrile-d_3) δ -63.26 (s, BAr^F$_4$). UV-Vis (MeCN) λ nm (ε × 10^{-4} M^{-1} cm^{-1}) 284 (8.59), 448 (1.50). HRMS (MALDI): m/z = 1433.1752 [Ru(bpy)$_3$ + BAr^F$_4$]$^+$ (calculated 1433.1773 for $C_{62}H_{36}BF_{24}N_6Ru$).

Ru1P·BAr^F$_4$

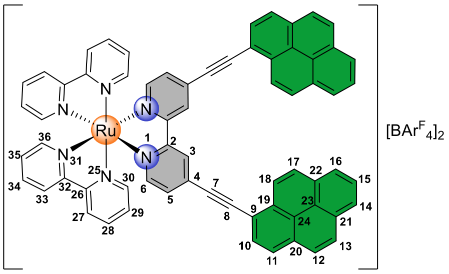

General method was followed using **Ru1P·PF$_6$** (20 mg, 0.02 mmol) and Na(BAr^F$_4$) (34 mg, 0.04 mmol). **Ru1P·BAr^F$_4$** was isolated as an orange solid (40 mg, 95%). ^{1}H NMR (500 MHz, Acetonitrile-*d*$_3$) δ 8.98 (d, *J* = 1.8 Hz, 2H, H^{3}), 8.81 (d, *J* = 9.2 Hz, 2H, H^{18}), 8.55 (d, *J* = 8.1 Hz, 4H, H^{27} and H^{33}), 8.44 – 8.40 (m, 4H, H^{17} and H^{16}), 8.39 (d, *J* = 7.6 Hz, 2H, H^{14}), 8.36 (d, *J* = 7.9 Hz, 2H, H^{10}), 8.33 (d, *J* = 7.9 Hz, 2H, H^{11}), 8.30 (d, *J* = 8.8 Hz, 2H, H^{13}), 8.22 (d, *J* = 8.8 Hz, 2H, H^{12}), 8.17 (t, *J* = 7.6 Hz, 2H, H^{15}), 8.14 – 8.08 (m, 4H, H^{34} and H^{28}), 7.90 (d, *J* = 5.9 Hz, 2H, H^{30}), 7.83 (d, *J* = 5.9 Hz, 2H, H^{6}), 7.77 (d, *J* = 5.3 Hz, 2H, H^{36}), 7.72 – 7.64 (m, 26H, H^{5} and H^{BArF4}), 7.48 (dd, *J* = 7.6, 5.9 Hz, 2H, H^{29}), 7.45 (ddd, *J* = 7.5, 5.3, 1.4 Hz, 2H, H^{35}). ^{13}C{^{1}H} NMR (126 MHz, Acetonitrile-*d*$_3$) δ 163.2 (C^{BArF4}), 162.8 (C^{BArF4}), 162.4 (C^{BArF4}), 162.0 (C^{BArF4}), 158.0 (C^{2}), 157.91 (C^{26}), 157.85 (C^{32}), 152.9 (C^{30}), 152.8 (C^{6}), 152.7 (C^{36}), 139.1 (C^{28}), 139.0 (C^{34}), 135.6 (C^{BArF4}), 133.7 (C^{20}), 133.6 (C^{4}), 133.5 (C^{19}), 132.2 (C^{21}), 131.9 (C^{22}), 131.3 (C^{10}), 130.5 (C^{17}), 130.4 (C^{13}), 130.29 (C^{BArF4}), 130.27 (C^{BArF4}), 130.25 (C^{BArF4}), 130.2 (C^{BArF4}), 130.04 (C^{BArF4}), 130.02 (C^{BArF4}), 130.00 (C^{BArF4}), 129.97 (C^{BArF4}), 129.79 (C^{BArF4}), 129.77 (C^{BArF4}), 129.75 (C^{BArF4}), 129.72 (C^{BArF4}), 129.68 (C^{5}), 129.52 (C^{BArF4}), 129.49 (C^{BArF4}), 129.47 (C^{BArF4}), 128.71 (C^{29}), 128.68 (C^{35}), 128.67 (C^{BArF4}), 128.2 (C^{12}), 128.0 (C^{15}), 127.6 (C^{14}), 127.5 (C^{16}), 127.3 (C^{3}), 126.5 (C^{BArF4}), 126.0 (C^{11}), 125.8 (C^{18}), 125.3 (C^{27} and C^{33}), 125.1 (C^{24}), 124.7 (C^{23}), 124.3 (C^{BArF4}), 122.2 (C^{BArF4}), 118.6 (C^{BArF4}), 116.1 (C^{9}), 98.1 (C^{8}), 92.2 (C^{7}). ^{19}F NMR (376 MHz, Acetonitrile-*d*$_3$) δ -63.26 (s, BAr^F$_4$). UV-Vis (MeCN) λ nm (ε × 10^{-4} M^{-1} cm^{-1}) 281 (12.0), 384 (5.99), 412 (6.81), 487 (3.83). HRMS (MALDI): m/z = 1881.3010 [Ru1P + BAr^F$_4$]$^+$ (calculated 1881.3037 for $C_{98}H_{52}BF_{24}N_6Ru$).

Ru2P·BAr^F$_4$

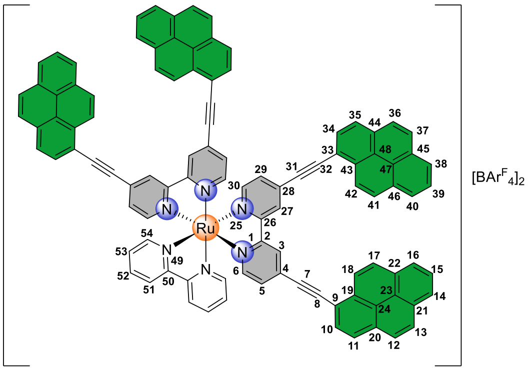

General method was followed using **Ru2P·PF$_6$** (20 mg, 0.01 mmol) and Na(BAr^F$_4$) (17 mg, 0.02 mmol). **Ru2P·BAr^F$_4$** was isolated as a red solid (33 mg, 92%). ^{1}H NMR (500 MHz, Acetonitrile-*d*$_3$) δ 9.03 (s, 4H, H^{3} and H^{27}), 8.85 – 8.78 (m, 4H, H^{18} and H^{42}), 8.61 (d, *J* = 8.1 Hz, 2H, H^{51}), 8.46 – 8.29 (m, 22H, H^{10}, H^{11}, H^{13} or H^{37}, H^{14}, H^{16}, H^{17}, H^{34}, H^{35}, H^{38}, H^{40}, H^{41}), 8.28 (d, *J* = 8.7 Hz, 2H, H^{13} or H^{37}), 8.23 (d, *J* = 8.7 Hz, 2H, H^{12} or H^{36}), 8.20 (d, *J* = 9.0 Hz, 2H, H^{12} or H^{36}), 8.19 – 8.12 (m, 6H, H^{15}, H^{39} and H^{52}), 8.01 (d, *J* = 6.0 Hz, 2H, H^{30}), 7.96 (d, *J* = 6.0 Hz, 2H, H^{54}), 7.88 (d, *J* = 6.0 Hz, 2H, H^{6}), 7.78 (dd, *J* = 6.0, 1.8 Hz, 2H, H^{29}), 7.74 (dd, *J* = 6.0, 1.7 Hz, 2H, H^{5}), 7.70 – 7.64 (m, 24H, H^{BArF4}), 7.55 (t, *J* = 6.0 Hz, 2H, H^{53}). ^{13}C{^{1}H} NMR (101 MHz, Acetonitrile-*d*$_3$) δ 163.3 (C^{BArF}), 162.8 (C^{BArF4}), 162.3 (C^{BArF4}), 161.8 (C^{BArF4}), 158.0 (C^{26}), 157.9 (C^{2}), 157.8 (C^{50}), 153.0 (C^{30}), 152.9 (C^{54}), 152.8 (C^{6}), 139.3 (C^{52}), 135.6 (C^{BArF4}), 133.9 (C^{4} or C^{28}), 133.83 (C^{4} or C^{28}), 133.78 (C^{20} or C^{44}), 133.75 (C^{20} or C^{44}), 133.50 (C^{19} or C^{43}), 133.47 (C^{19} or C^{43}), 132.17 (C^{21} or C^{45}), 132.15 (C^{21} or C^{45}), 131.88 (C^{22} or C^{46}), 131.85 (C^{22} or C^{46}), 131.4 (C^{10} or C^{34}), 131.3 (C^{10} or C^{34}), 130.48 (C^{17} or C^{41}), 130.46 (C^{17} or C^{41}), 130.44 (C^{13} or C^{37}), 130.41 (C^{13} or C^{37}), 130.08 (C^{BArF4}), 130.05 (C^{BArF4}), 130.0 (C^{BArF4}), 129.84 (C^{5} and C^{29}), 129.76 (C^{BArF4}), 129.74 (C^{BArF4}), 129.71 (C^{BArF4}), 129.68 (C^{BArF4}), 128.9 (C^{53}), 128.22 (C^{12} or C^{36}), 128.19 (C^{12} or C^{36}), 127.99 (C^{15} or C^{39}), 127.96 (C^{15} or C^{39}), 127.58 (C^{14} or C^{38}), 127.55 (C^{14} or C^{38}), 127.50 (C^{16} or C^{40}), 127.48 (C^{16} or C^{40}), 127.4 (C^{3} and C^{27}), 126.8 (C^{BArF4}), 125.99 (C^{11} or C^{35}), 125.97 (C^{11} or C^{35}), 125.8 (C^{18} and C^{42}), 125.5 (C^{51}), 125.14 (C^{24} or C^{48}), 125.11 (C^{24} or C^{48}), 124.8 (C^{23} or C^{47}), 124.7 (C^{23} or C^{47}), 124.1 (C^{BArF4}), 121.4 (C^{BArF4}), 118.68 (C^{BArF4}), 118.65 (C^{BArF4}), 118.6 (C^{BArF4}), 116.1 (C^{9} or C^{33}), 116.0 (C^{9} or C^{33}), 98.3 (C^{8} and C^{32}), 92.29 (C^{7} or C^{31}), 92.28 (C^{7} or C^{31}). ^{19}F NMR (376

MHz, Acetonitrile-d_3) δ -63.26 (s, $BAr^F{}_4$). UV-Vis (MeCN) λ nm (ε × 10^{-4} M^{-1} cm^{-1}) 280 (13.0), 385 (9.22), 412 (10.2), 498 (5.40). HRMS (MALDI): m/z = 2329.4282 [Ru2P + $BAr^F{}_4$]$^+$ (calculated 2329.4300 for $C_{134}H_{68}BF_{24}N_6Ru$).

Ru3P·$BAr^F{}_4$

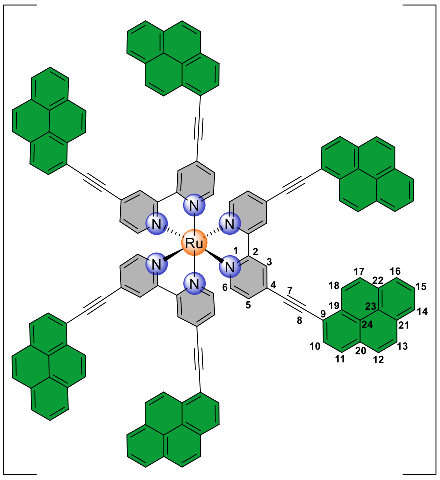

$[BAr^F{}_4]_2$

General method was followed using **Ru3P·PF_6** (20 mg, 0.01 mmol) and Na($BAr^F{}_4$) (17 mg, 0.02 mmol). **Ru3P·$BAr^F{}_4$** was isolated as a red solid (30 mg, 91%). ^{1}H NMR (500 MHz, Acetonitrile-d_3) δ 9.10 (d, J = 1.5 Hz, 6H, H^3), 8.86 (d, J = 9.2 Hz, 6H, H^{18}), 8.45 (d, J = 9.2 Hz, 6H, H^{17}), 8.42 (d, J = 7.7 Hz, 6H, H^{16}), 8.41 (d, J = 8.1 Hz, 6H, H^{10}), 8.39 (d, J = 7.7 Hz, 6H, H^{14}), 8.35 (d, J = 8.1 Hz, 6H, H^{11}), 8.30 (d, J = 9.0 Hz, 6H, H^{13}), 8.23 (d, J = 9.0 Hz, 6H, H^{12}), 8.17 (t, J = 7.7 Hz, 6H, H^{15}), 8.07 (d, J = 5.9 Hz, 6H, H^6), 7.84 (dd, J = 5.9, 1.5 Hz, 6H, H^5), 7.71 – 7.64 (m, 24H, H^{BArF4}). ^{13}C{^{1}H} NMR (126 MHz, Acetonitrile-d_3) δ 163.2 (C^{BArF4}), 162.8 (C^{BArF4}), 162.4 (C^{BArF4}), 162.0 (C^{BArF4}), 157.9 (C^2), 153.0 (C^6), 135.6 (C^{BArF4}), 134.1 (C^4), 133.8 (C^{20}), 133.5 (C^{19}), 132.2 (C^{21}), 131.9 (C^{22}), 131.4 (C^{10}), 130.5 (C^{17}), 130.4 (C^{13}), 130.03 (C^{BArF4}), 130.01 (C^{BArF4}), 129.99 (C^5), 129.8 (C^{BArF4}), 129.74 (C^{BArF4}), 129.71 (C^{BArF4}), 128.7 (C^{BArF4}), 128.2 (C^{12}), 128.0 (C^{15}), 127.6 (C^{14}), 127.49 (C^{16}), 127.48 (C^3), 126.5 (C^{BArF4}), 126.0 (C^{11}), 125.8 (C^{18}), 125.1 (C^{24}), 124.7 (C^{23}), 124.3 (C^{BArF4}), 122.2 (C^{BArF4}), 118.7 (C^{BArF4}), 118.64 (C^{BArF4}), 118.61 (C^{BArF4}), 116.0 (C^9), 98.5 (C^8), 92.3 (C^7). ^{19}F NMR (376 MHz, Acetonitrile-d_3) δ -63.26 (s, $BAr^F{}_4$). UV-Vis (MeCN) λ nm (ε × 10^{-4} M^{-1} cm^{-1}) 279 (16.8), 305 (14.7), 385 (14.8), 411 (ε = 16.0), 491 (ε = 9.28). HRMS (MALDI): m/z = 2777.5581 [Ru3P + $BAr^F{}_4$]$^+$ (calculated 2777.5562 for $C_{170}H_{84}BF_{24}N_6Ru$).

Ru1C·$BAr^F{}_4$

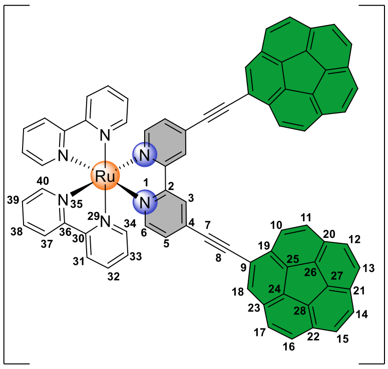

$[BAr^F{}_4]_2$

General method was followed using **Ru1C·PF_6** (20 mg, 0.01 mmol) and Na($BAr^F{}_4$) (17 mg, 0.02 mmol). **Ru1C·$BAr^F{}_4$** was isolated as an orange solid (38 mg, 95%). ^{1}H NMR (500 MHz, Acetonitrile-d_3) δ 8.91 (d, J = 1.1 Hz, 2H, H^3), 8.54 (d, J = 8.2 Hz, 4H, H^{31} and H^{37}), 8.33 (s, 2H, H^{18}), 8.30 (d, J = 8.8 Hz, 2H, H^{10}), 8.12 – 8.07 (m, 6H, H^{11}, H^{32} and H^{38}), 8.03 – 7.90 (m, 12H, H^{12}, H^{13}, H^{14}, H^{15}, H^{16} and H^{17}), 7.86 (d, J = 5.3 Hz, 2H, H^{34}), 7.82 (d, J = 5.9 Hz, 2H, H^6), 7.75 (d, J = 5.2 Hz, 2H, H^{40}), 7.73 – 7.65 (m, 24H, H^{BArF4}), 7.64 (dd, J = 5.9, 1.7 Hz, 2H, H^5), 7.52 – 7.41 (m, 4H, H^{33} and H^{39}). ^{13}C{^{1}H} NMR (101 MHz, Acetonitrile-d_3) δ 163.3 (C^{BArF4}), 162.8 (C^{BArF4}), 162.3 (C^{BArF4}), 161.8 (C^{BArF4}), 158.0 (C^2), 157.9 (C^{30} or C^{36}), 157.8 (C^{30} or C^{36}), 152.9 (C^{34}), 152.8 (C^6), 152.6 (C^{40}), 139.1 (C^{32} or C^{38}), 139.0 (C^{32} or C^{38}), 136.8 (C^{27} or C^{28}), 136.6 (C^{24}), 136.4 (C^{26}), 136.1 (C^{27} or C^{28}), 135.8 (C^{25}), 135.6 (C^{BArF4}), 134.4 (C^{18}), 133.2 (C^4), 132.9 (C^{22}), 132.5 (C^{20}), 132.3 (C^{21} or C^{23}), 131.4 (C^{19}), 131.0 (C^{21} or C^{23}), 129.9 (C^5), 129.5 (C^{11}), 129.3 (C^{Hcor}), 129.14 (C^{Hcor}), 129.05 (C^{Hcor}), 128.7 (C^{33} and C^{39}), 128.6 (C^{Hcor}), 128.5 (C^{12}), 128.0 (C^{17}), 127.4 (C^3), 126.8 (C^{BArF4}), 126.5 (C^{10}), 125.3 (C^{31} and C^{37}), 124.1 (C^{BArF4}), 120.0 (C^9), 118.68 (C^{BArF4}), 118.65 (C^{BArF4}), 118.6 (C^{BArF4}), 96.9 (C^8), 90.1 (C^7). ^{19}F NMR (470 MHz, Acetonitrile-d_3) δ -63.25 (s, $BAr^F{}_4$). UV-Vis (MeCN)

λ nm (ε × 10^{-4} M^{-1} cm^{-1}) 285 (1.27), 367 (4.78), 485 (2.68). HRMS (MALDI): m/z = 1977.3064 [Ru1C + BAr^F_4]$^+$ (calculated 1977.3040 for $C_{106}H_{52}BF_{24}N_6Ru$).

Ru2C·BAr$^F_4$

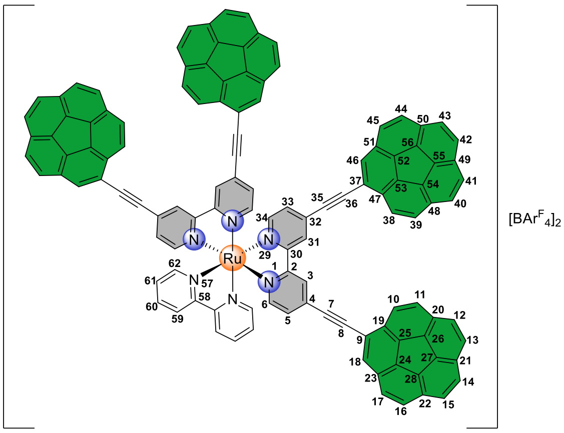

General method was followed using **Ru2C·PF$_6$** (20 mg, 0.01 mmol) and Na(BAr^F_4) (17 mg, 0.02 mmol). **Ru2C·BAr$^F_4$** was isolated as an orange solid (34 mg, 97%). ^{1}H NMR (500 MHz, Acetonitrile-d_3) δ 8.95 (s, 4H, H^3 and H^{31}), 8.59 (d, J = 8.5 Hz, 2H, H^{59}), 8.34 (s, 2H, H^{18} or H^{46}), 8.32 (d, J = 8.8 Hz, 2H, H^{10} or H^{38}), 8.31 (s, 2H, H^{18} or H^{46}), 8.29 (d, J = 8.8 Hz, 2H, H^{10} or H^{38}), 8.15 (t, J = 7.9 Hz, 2H, H^{60}), 8.10 (d, J = 8.8 Hz, 2H, H^{11} or H^{39}), 8.06 (d, J = 8.8 Hz, 2H, H^{11} or H^{39}), 8.02 – 7.91 (m, 26H, H^6 or H^{34}, H^{12}, H^{13}, H^{14}, H^{15}, H^{16}, H^{17}, H^{40}, H^{41}, H^{42}, H^{43}, H^{44} and H^{45}), 7.90 (d, J = 5.6 Hz, 2H, H^{62}), 7.84 (d, J = 5.7 Hz, 2H, H^6 or H^{34}), 7.73 – 7.64 (m, 28H, H^5, H^{33} and H^{BArF4}), 7.54 – 7.49 (m, 2H, H^{61}). ^{13}C{^{1}H} NMR (126 MHz, Acetonitrile-d_3) δ 163.2 (C^{BArF4}), 162.8 (C^{BArF4}), 162.4 (C^{BArF4}), 162.0 (C^{BArF4}), 157.92 (C^{30}), 157.86 (C^2), 157.8 (C^{58}), 153.0 (C^{34}), 152.9 (C^{62}), 152.8 (C^6), 139.3 (C^{60}), 136.8 (C_q^{cor}), 136.7 (C_q^{cor}), 136.63 (C^{24} or C^{52}), 136.59 (C^{24} or C^{52}), 136.34 (C^{26} or C^{54}), 136.30 (C^{26} or C^{54}), 136.13 (C_q^{cor}), 136.08 (C_q^{cor}), 135.74 (C^{25} or C^{53}), 135.70 (C^{25} or C^{53}), 135.6 (C^{BArF4}), 134.5 (C^{18} or C^{46}), 134.4 (C^{18} or C^{46}), 133.52 (C^4 or C^{32}), 133.50 (C^4 or C^{32}), 132.91 (C_q^{cor}), 132.86 (C_q^{cor}), 132.50 (C^{20} or C^{48}), 132.45 (C^{20} or C^{48}), 132.30 (C_q^{cor}), 132.25 (C_q^{cor}), 131.4 (C^{19} or C^{47}), 131.3 (C^{19} or C^{47}), 131.00 (C_q^{cor}), 130.96 (C_q^{cor}), 130.28 (C^{BArF4}), 130.26 (C^{BArF4}), 130.24 (C^{BArF4}), 130.22 (C^{BArF4}), 130.0 (C^5 and C^{39}), 129.78 (C^{BArF4}), 129.76 (C^{BArF4}), 129.73 (C^{BArF4}), 129.71 (C^{BArF4}), 129.52 (C^{11} or C^{39}), 129.48 (C^{11} or C^{39}), 129.3 (C^{Hcor}), 129.2 (C^{Hcor}), 129.14 (C^{Hcor}), 129.09 (C^{Hcor}), 129.04 (C^{Hcor}), 129.00 (C^{Hcor}), 128.8 (C^{61}), 128.7 (C^{BArF4}), 128.61 (C^{Hcor}), 128.56 (C^{Hcor}), 128.5 (C^{12} or C^{40}), 128.4 (C^{12} or C^{40}), 128.0 (C^{17} or C^{45}), 127.9 (C^{17} or C^{45}), 127.5 (C^3 and C^{31}), 126.5 (C^{10} and C^{38}), 126.4 (C^{BArF4}), 125.4 (C^{59}), 124.3 (C^{BArF4}), 122.2 (C^{BArF4}), 119.98 (C^9 or C^{37}), 119.96 (C^9 or C^{37}), 118.7 (C^{BArF4}), 118.64 (C^{BArF4}), 118.61 (C^{BArF4}), 97.19 (C^8 or C^{36}), 97.16 (C^8 or C^{36}), 90.1 (C^7 and C^{35}). ^{19}F NMR (470 MHz, Acetonitrile-d_3) δ -63.26 (s, BAr^F_4). UV-Vis (MeCN) λ nm (ε × 10^{-4} M^{-1} cm^{-1}) 290 (1.65), 368 (8.49), 494 (4.00). HRMS (MALDI): m/z = 2521.4331 [Ru2C + BAr^F_4]$^+$ (calculated 2521.4305 for $C_{150}H_{68}BF_{24}N_6Ru$).

Ru3C·BAr$^F_4$

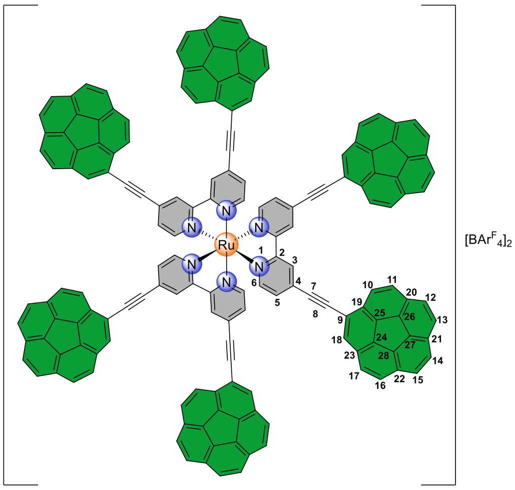

General method was followed using **Ru3C·PF$_6$** (20 mg, 0.01 mmol) and Na(BAr^F_4) (17 mg, 0.02 mmol). **Ru3C·BAr$^F_4$** was isolated as a red solid (30 mg, 94%). ^{1}H NMR (500 MHz, Acetonitrile-d_3) δ 9.00 (s, 6H, H^3), 8.34 (s, 6H, H^{18}), 8.32 (d, J = 8.8 Hz, 6H, H^{10}), 8.08 (d, J = 8.8 Hz, 6H, H^{11}), 7.99 (d, J = 5.9 Hz, 6H, H^6), 7.99 – 7.92

(m, 36H, H^{12}, H^{13}, H^{14}, H^{15}, H^{16} and H^{17}), 7.76 (d, *J* = 5.9 Hz, 6H, H^{5}), 7.72 – 7.64 (m, 24H, H^{BArF4}). $^{13}C\{^{1}H\}$ NMR (101 MHz, Acetonitrile-d_3) δ 163.3 (C^{BArF4}), 162.8 (C^{BArF4}), 162.3 (C^{BArF4}), 161.8 (C^{BArF4}), 157.8 (C^{2}), 153.0 (C^{6}), 136.8 (C_q^{cor}), 136.6 (C^{24}), 136.3 (C^{26}), 136.1 (C_q^{cor}), 135.7 (C^{25}), 135.6 (C^{BArF4}), 134.5 (C^{18}), 133.8 (C^{4}), 132.9 (C_q^{cor}), 132.5 (C^{20}), 132.3 (C_q^{cor}), 131.4 (C^{19}), 131.0 (C_q^{cor}), 130.1 (C^{5}), 129.5 (C^{11}), 129.2 (C^{Hcor}), 129.1 (C^{Hcor}), 129.0 (C^{Hcor}), 128.6 (C^{Hcor}), 128.4 (C^{12}), 127.9 (C^{17}), 127.6 (C^{3}), 126.8 (C^{BArF4}), 126.5 (C^{10}), 124.1 (C^{BArF4}), 119.9 (C^{9}), 97.4 (C^{8}), 90.1 (C^{7}). ^{19}F NMR (376 MHz, Acetonitrile-d_3) δ -63.26 (s, BAr^{F}_4). UV-Vis (MeCN) λ nm (ε × 10^{-4} M^{-1} cm^{-1}) 294 (16.0), 370 (10.2), 489 (5.42). HRMS (MALDI): m/z = 3065.5582 $[Ru3C + BAr^{F}_4]^+$ (calculated 3065.5568 for $C_{194}H_{84}BF_{24}N_6Ru$).

NMR and HR-MS Spectra

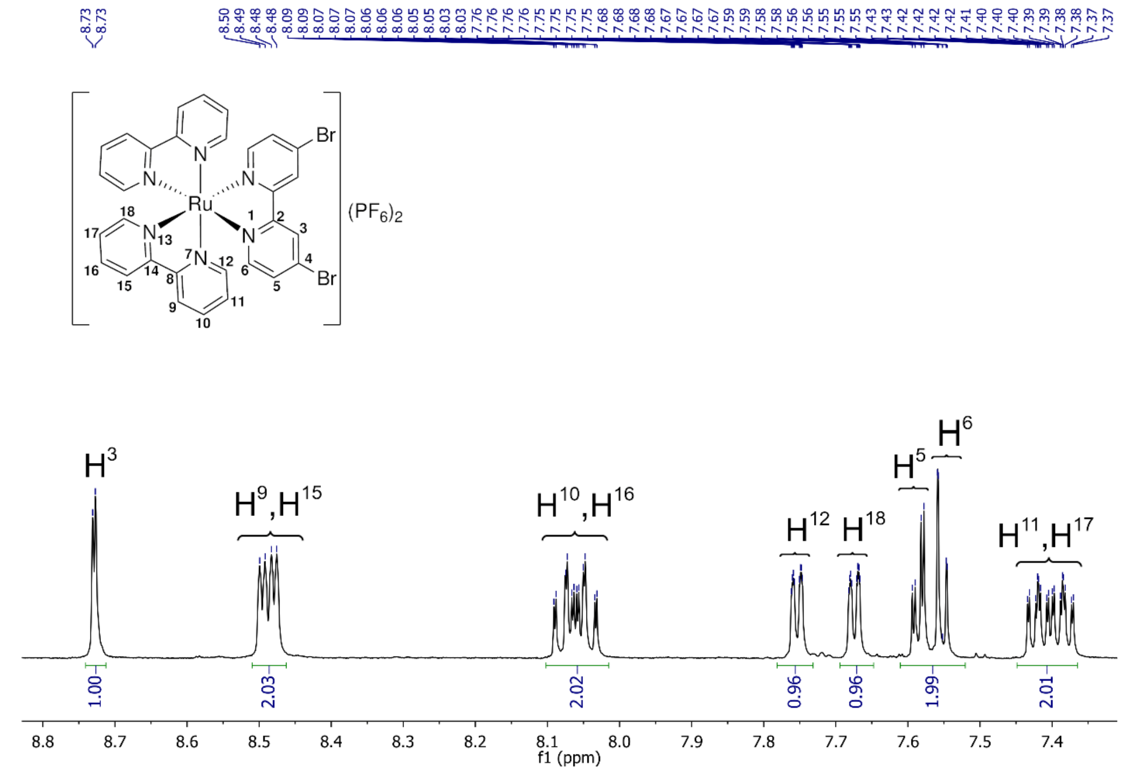

Figure S 1. ^{1}H-NMR (298 K, 500 MHz, Acetonitrile-d_3) spectrum of **Ru1Br·PF_6**.

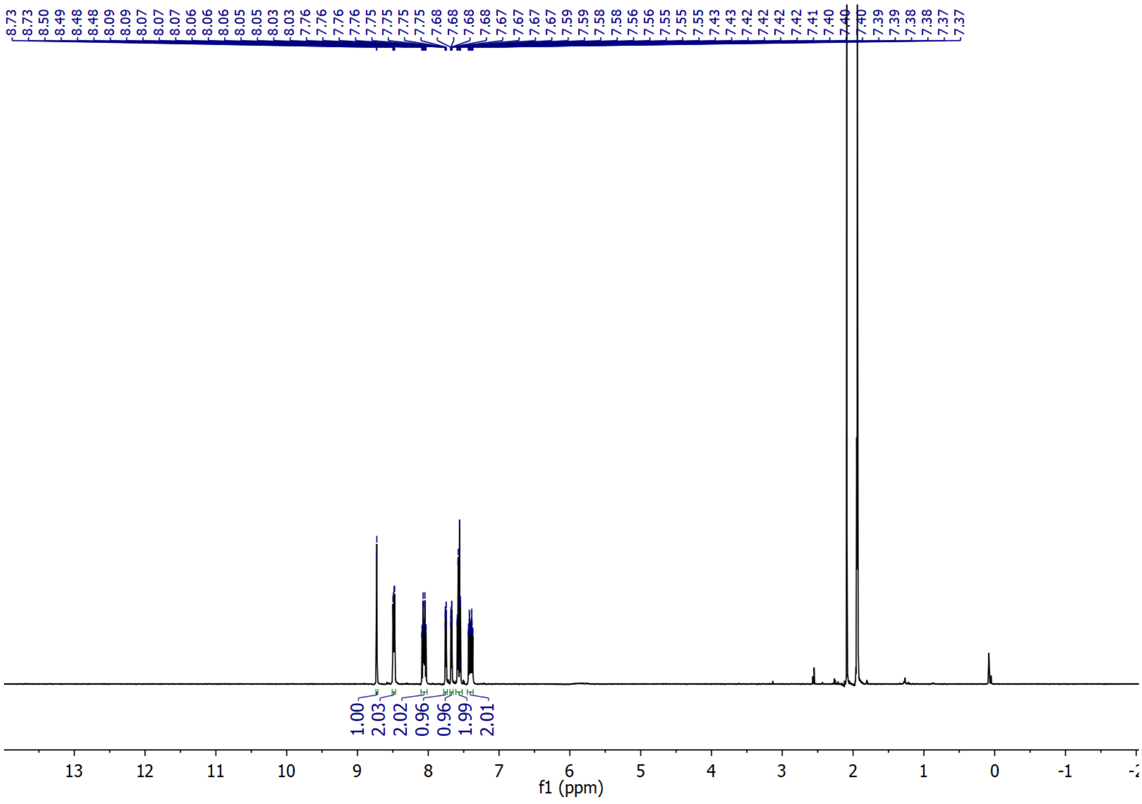

Figure S 2. Full ^{1}H-NMR (298 K, 500 MHz, Acetonitrile-d_3) spectrum of **Ru1Br·PF_6**.

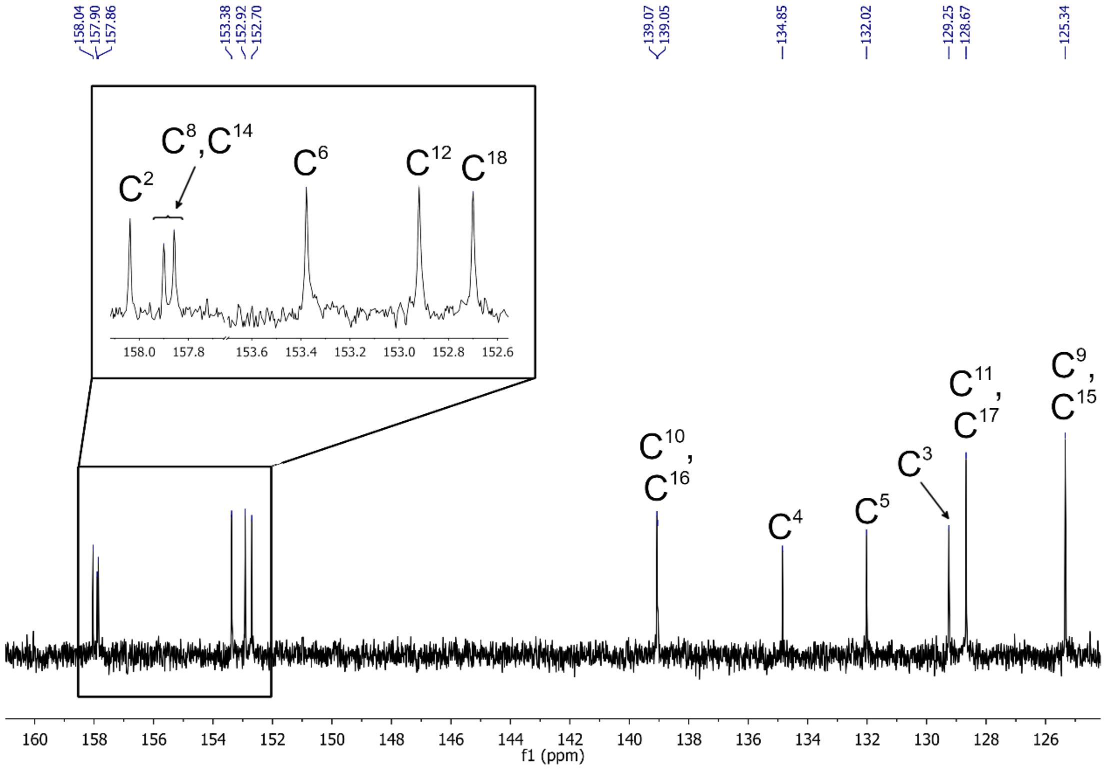

Figure S 3. $^{13}C\{^1H\}$-NMR (298 K, 101 MHz, Acetonitrile-d_3) spectrum of **Ru1Br·PF$_6$**.

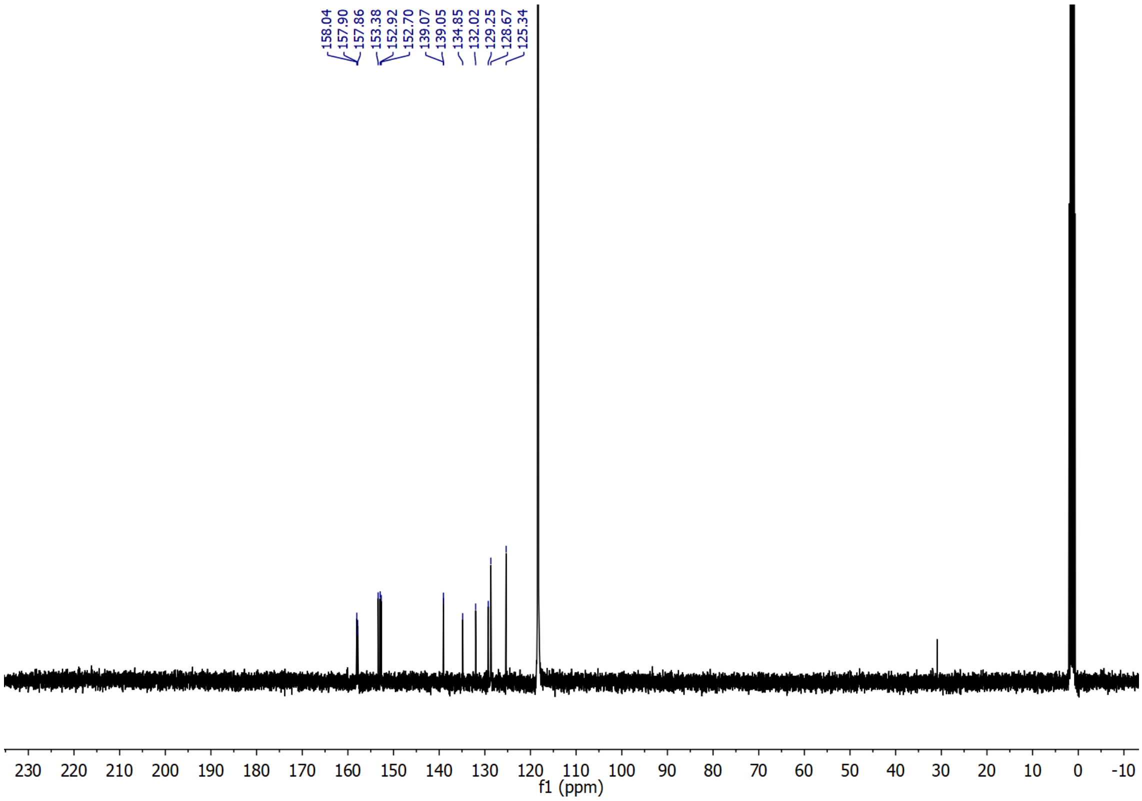

Figure S 4. Full $^{13}C\{^1H\}$-NMR (298 K, 101 MHz, Acetonitrile-d_3) spectrum of **Ru1Br·PF$_6$**.

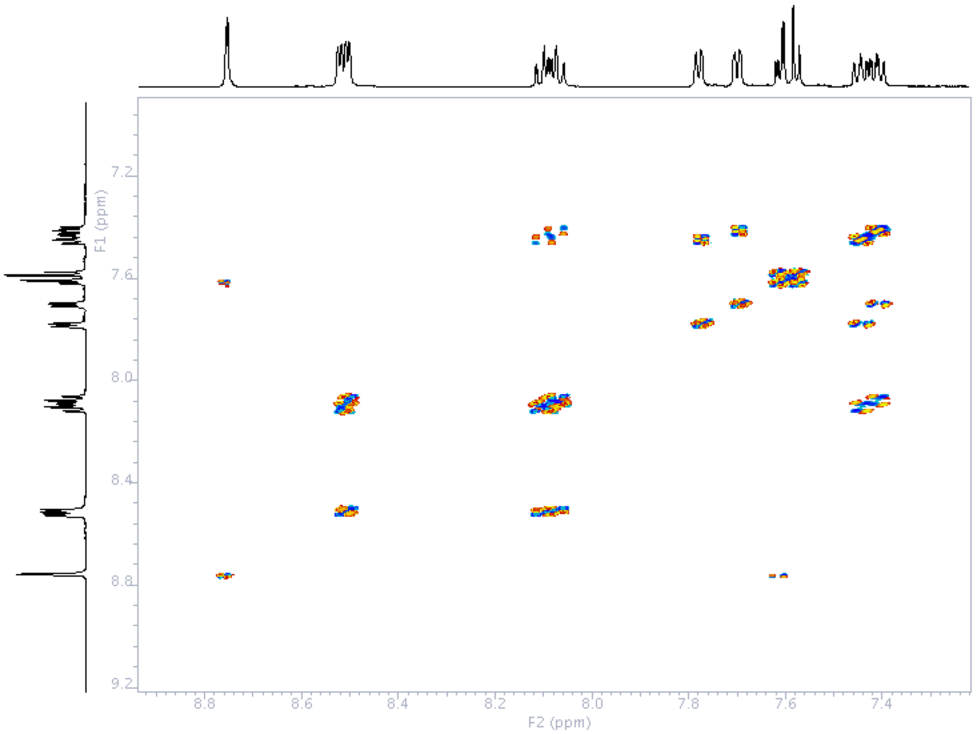

Figure S 5. ^{1}H-^{1}H gDQCOSY (298 K, 500 MHz, Acetonitrile-*d*$_3$) spectrum of compound **Ru1Br·PF$_6$**.

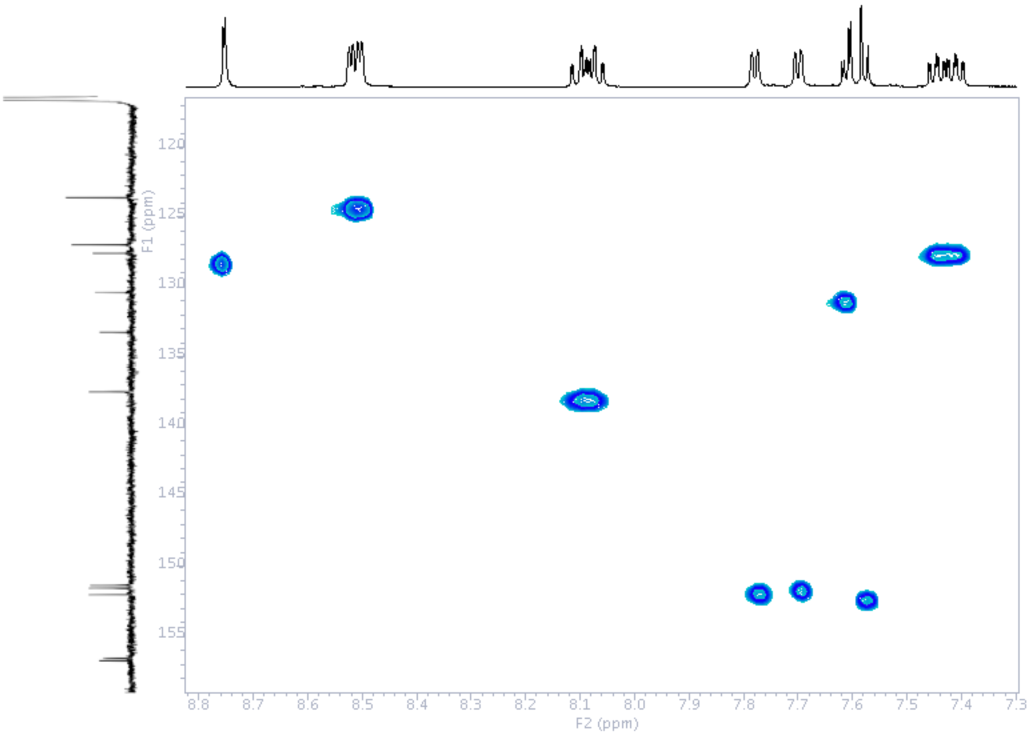

Figure S 6. ^{1}H-^{13}C gc2HSQC (298 K, 500 MHz, Acetonitrile-*d*$_3$) spectrum of compound **Ru1Br·PF$_6$**.

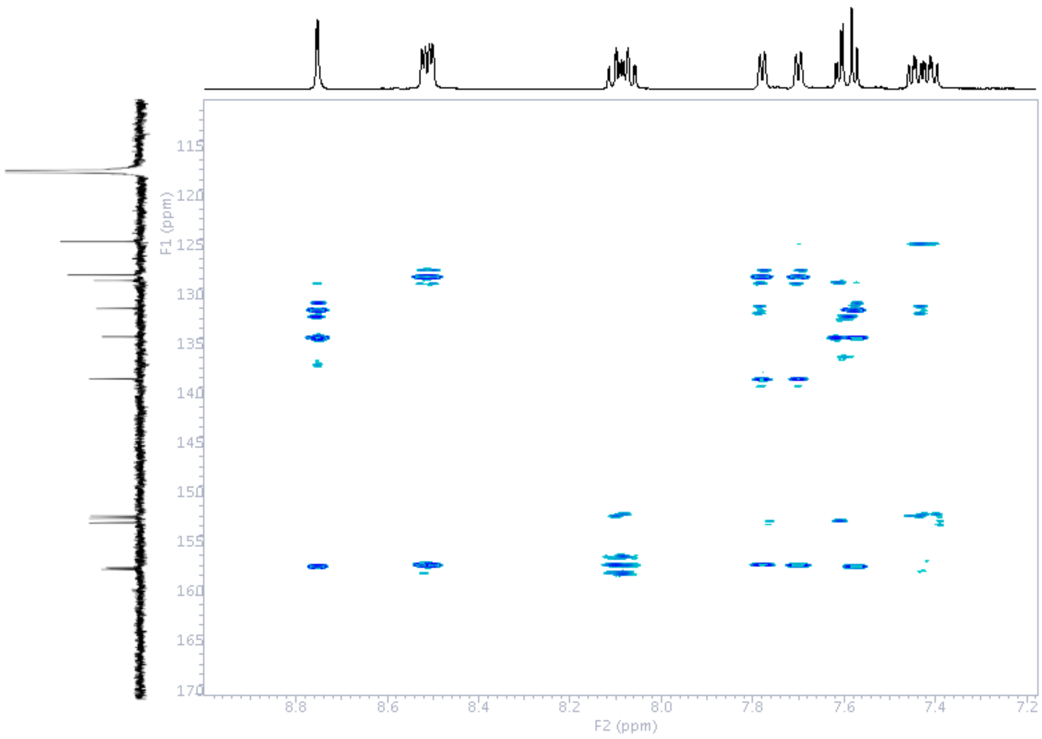

Figure S 7. ^{1}H-^{13}C bsgHMBC (298 K, 500 MHz, Acetonitrile-*d*$_3$) spectrum of compound **Ru1Br·PF$_6$**.

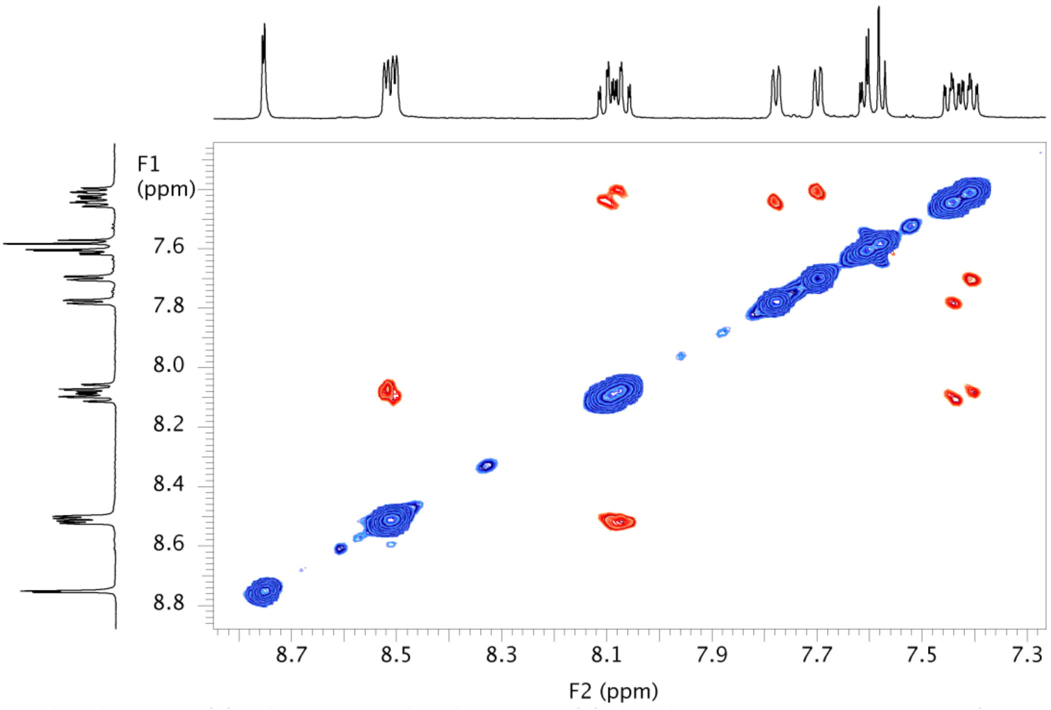

Figure S 8. ^{1}H-^{1}H ROESYAD (298 K, 500 MHz, Acetonitrile-*d*$_3$) spectrum of compound **Ru1Br·PF$_6$**.

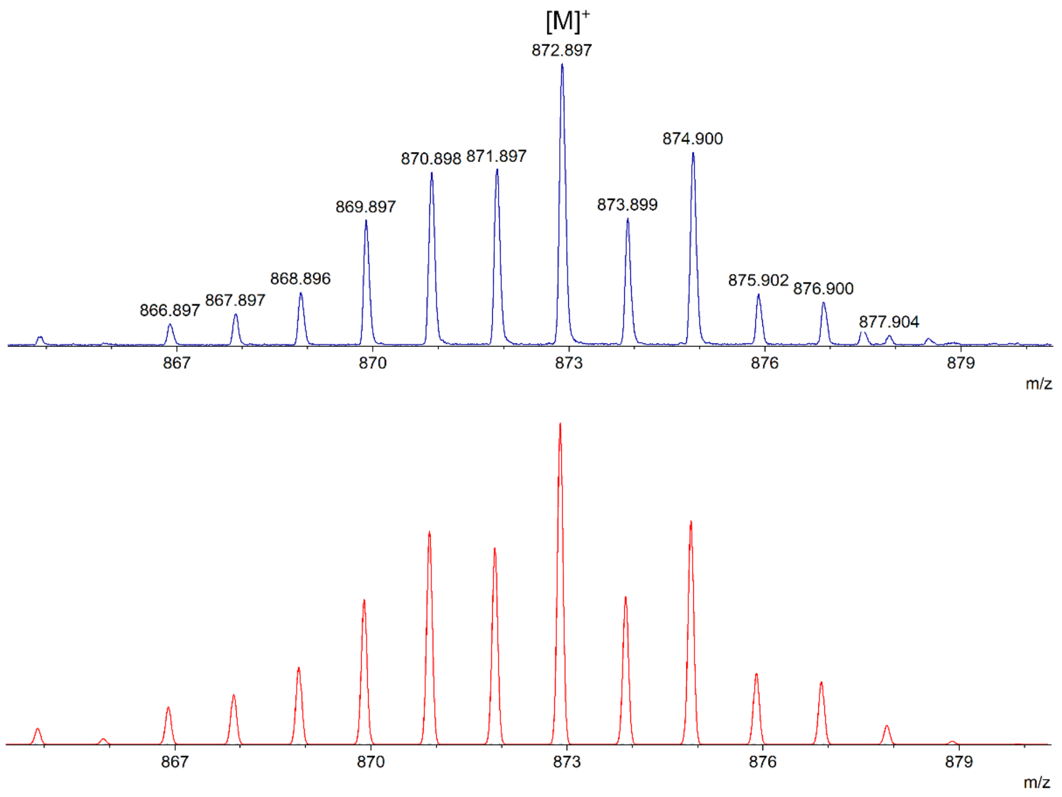

Figure S 9. HRMS (MALDI) of **Ru1Br·PF$_6$**, [Ru1Br + PF$_6$]$^+$**.** Calculated (red), measured (blue).

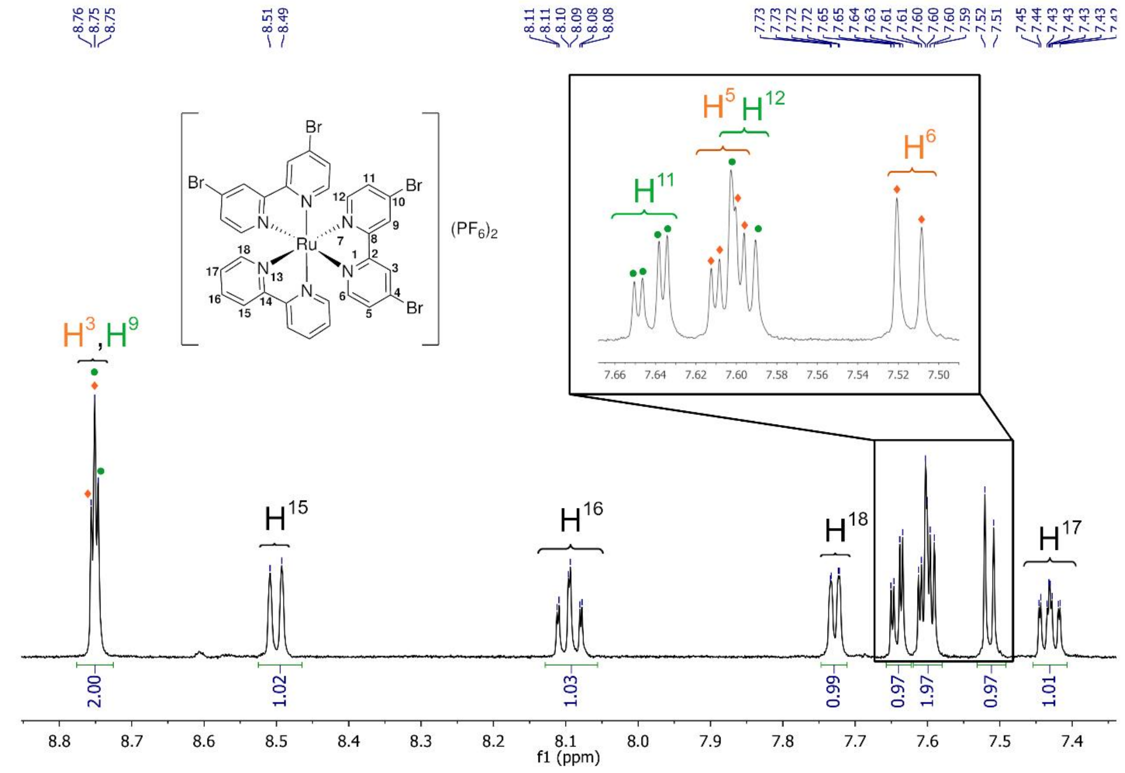

Figure S 10. ^{1}H-NMR (298 K, 500 MHz, Acetonitrile-d_3) spectrum of **Ru2Br·PF$_6$**.

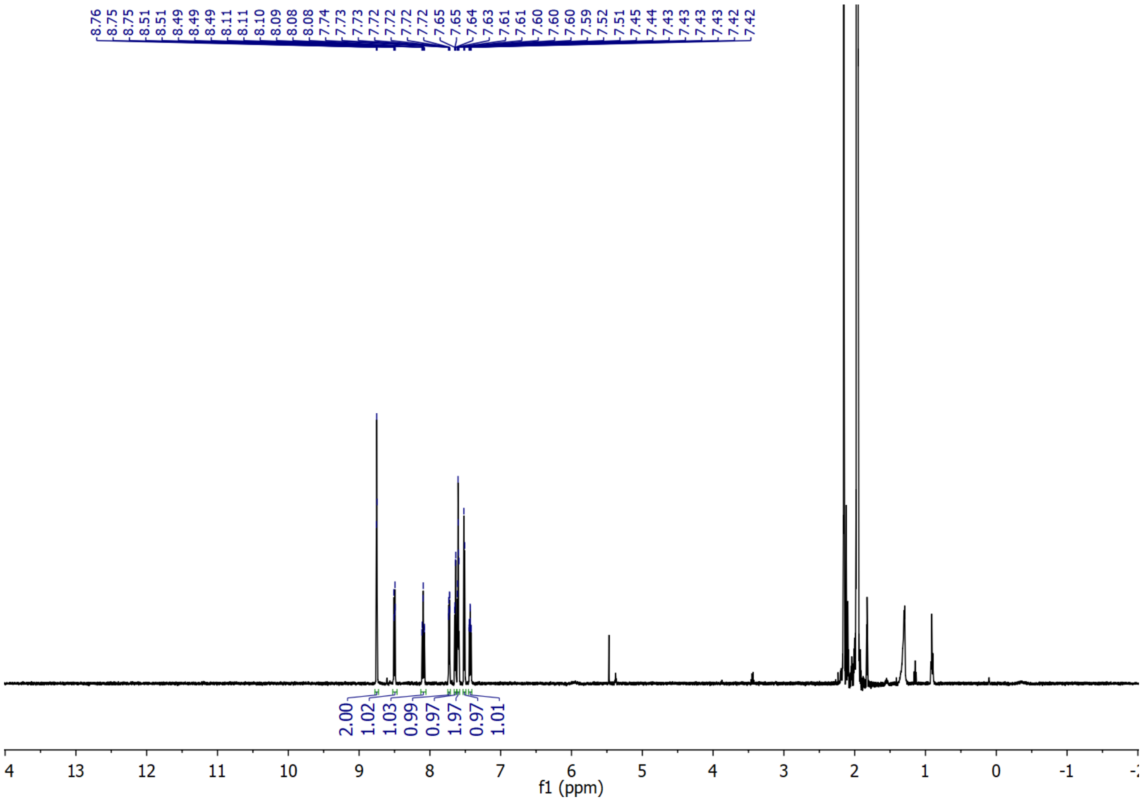

Figure S 11. Full ^{1}H-NMR (298 K, 500 MHz, Acetonitrile-d_3) spectrum of **Ru2Br·PF$_6$**.

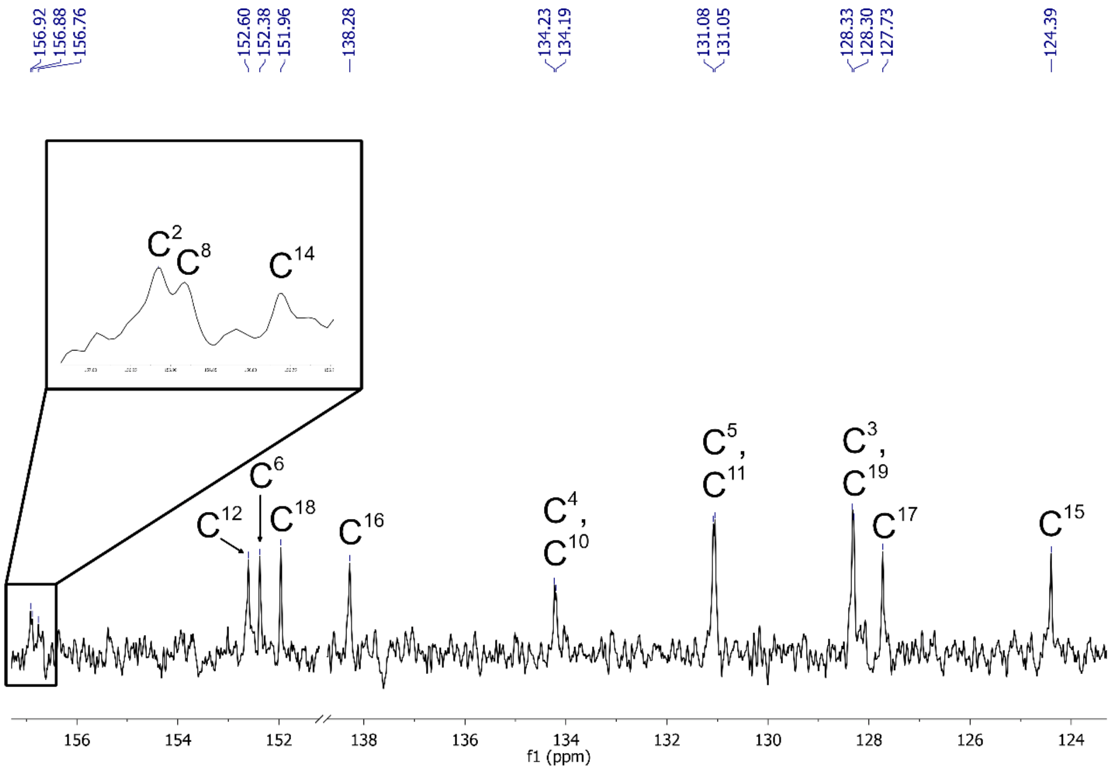

Figure S 12. $^{13}C\{^{1}H\}$-NMR (298 K, 101 MHz, Acetonitrile-d_3) spectrum of **Ru2Br·PF₆**.

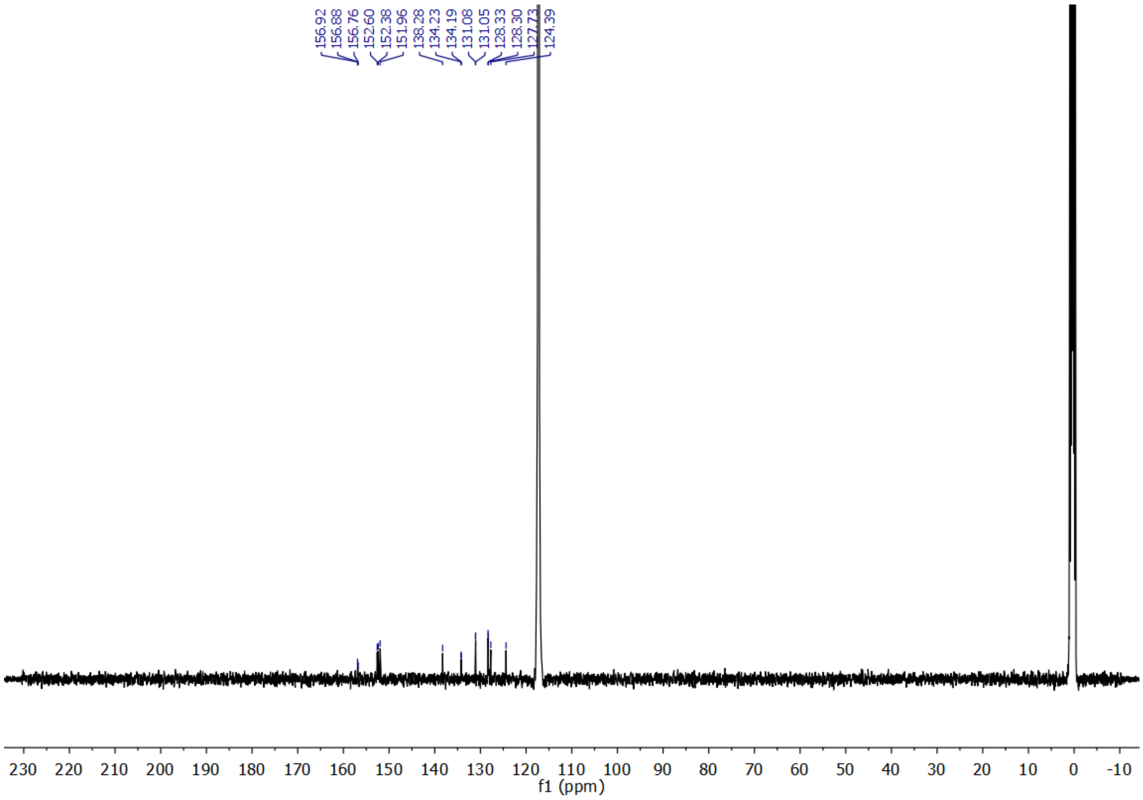

Figure S 13. Full $^{13}C\{^{1}H\}$-NMR (298 K, 101 MHz, Acetonitrile-d_3) spectrum of **Ru2Br·PF₆**.

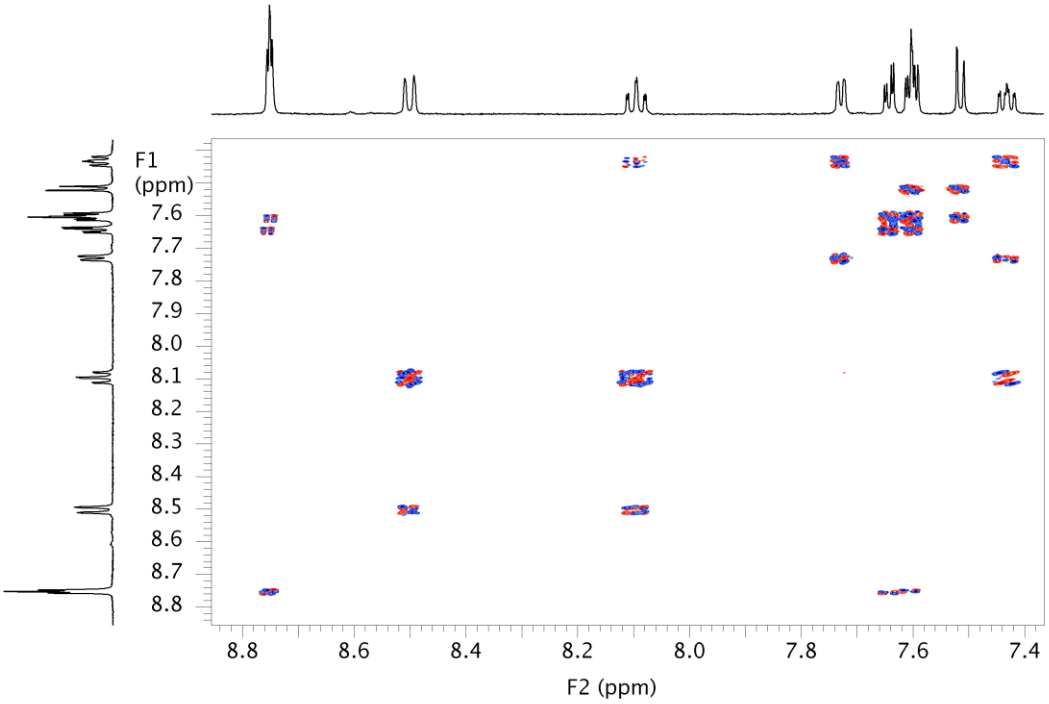

Figure S 14. ^{1}H-^{1}H gDQCOSY (298 K, 500 MHz, Acetonitrile-d_3) spectrum of compound **Ru2Br·PF$_6$**.

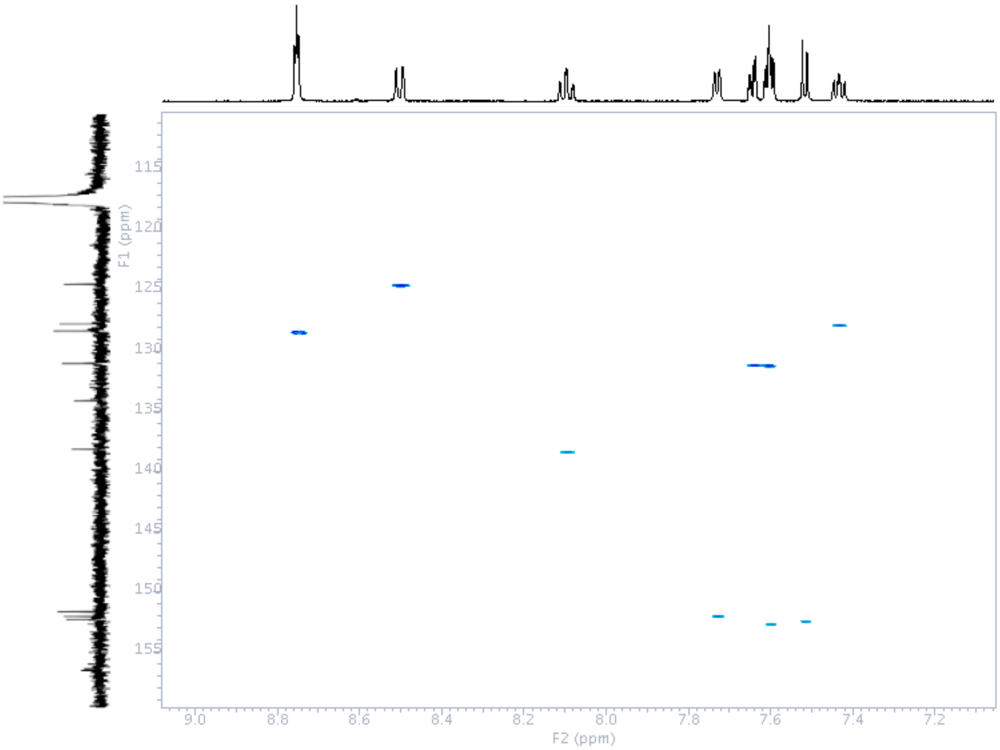

Figure S 15. ^{1}H-^{13}C bsgHSQC (298 K, 500 MHz, Acetonitrile-d_3) spectrum of compound **Ru2Br·PF$_6$**.

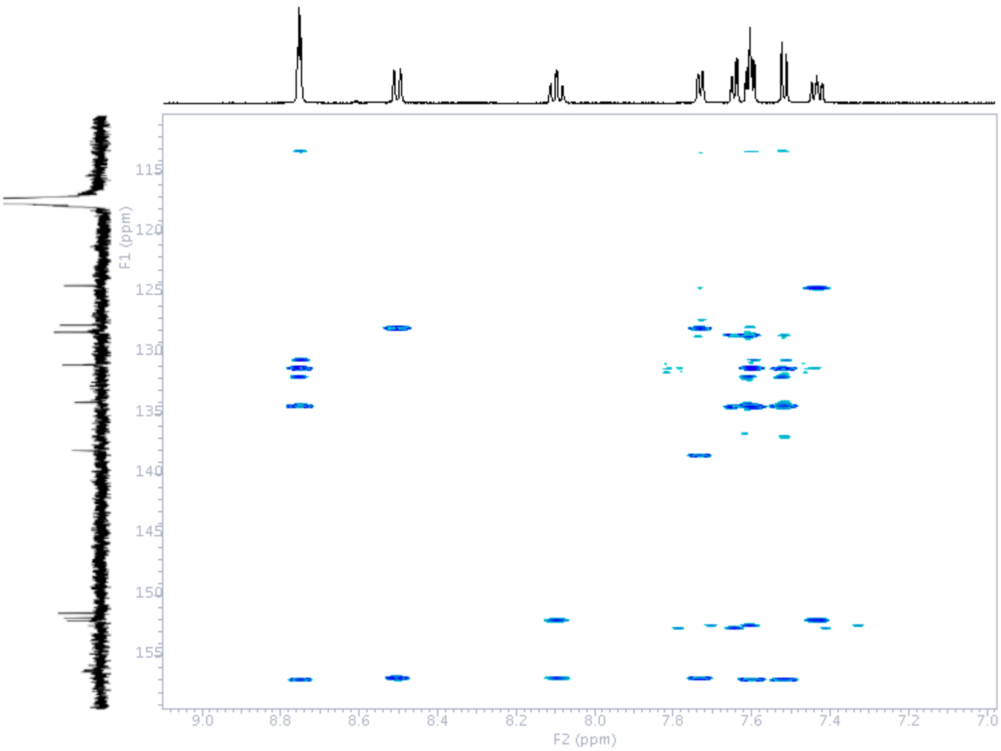

Figure S 16. ^{1}H-^{13}C bsgHMBC (298 K, 500 MHz, Acetonitrile-*d*$_3$) spectrum of compound **Ru2Br·PF$_6$**.

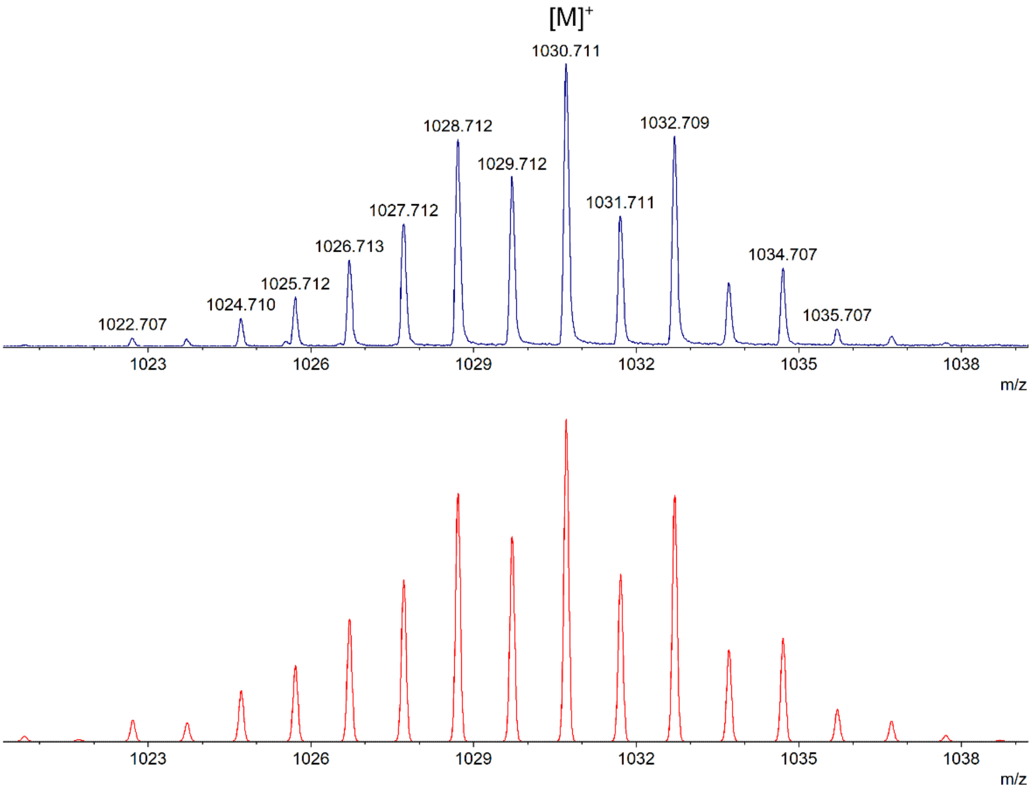

Figure S 17. HRMS (MALDI) of **Ru2Br·PF$_6$**, [Ru2Br + PF$_6$]$^+$**.** Calculated (red), measured (blue).

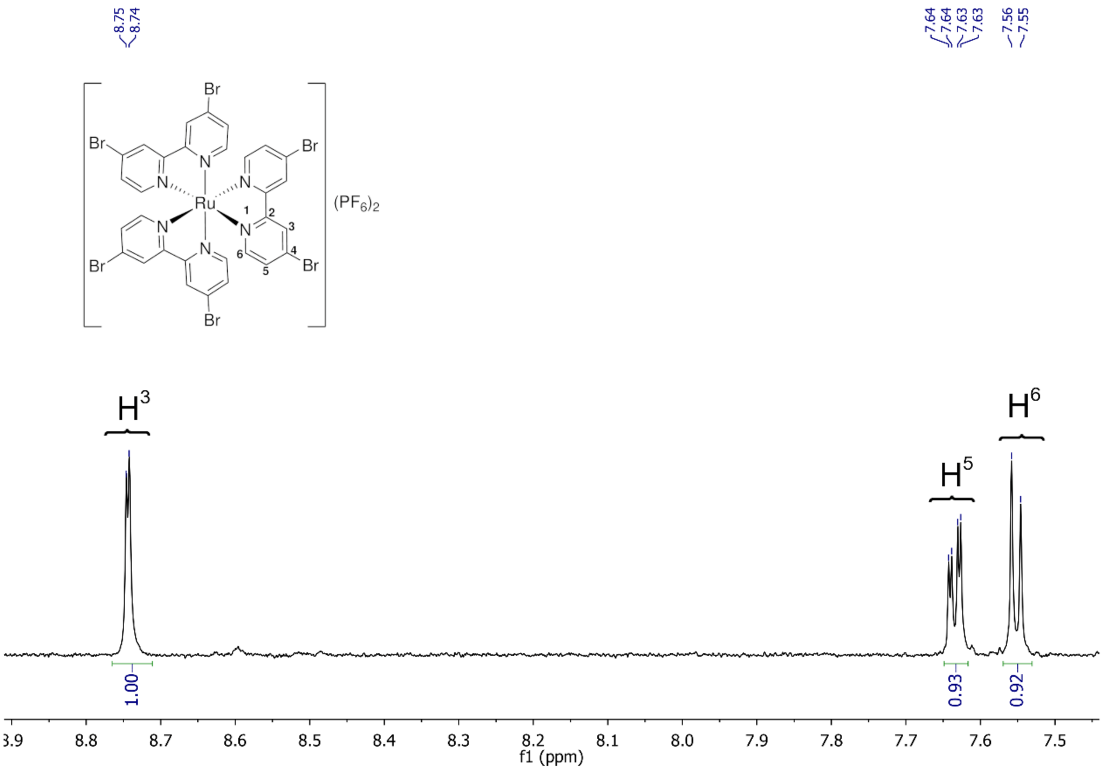

Figure S 18. ^{1}H-NMR (298 K, 500 MHz, Acetonitrile-d_3) spectrum of **Ru3Br·PF$_6$**.

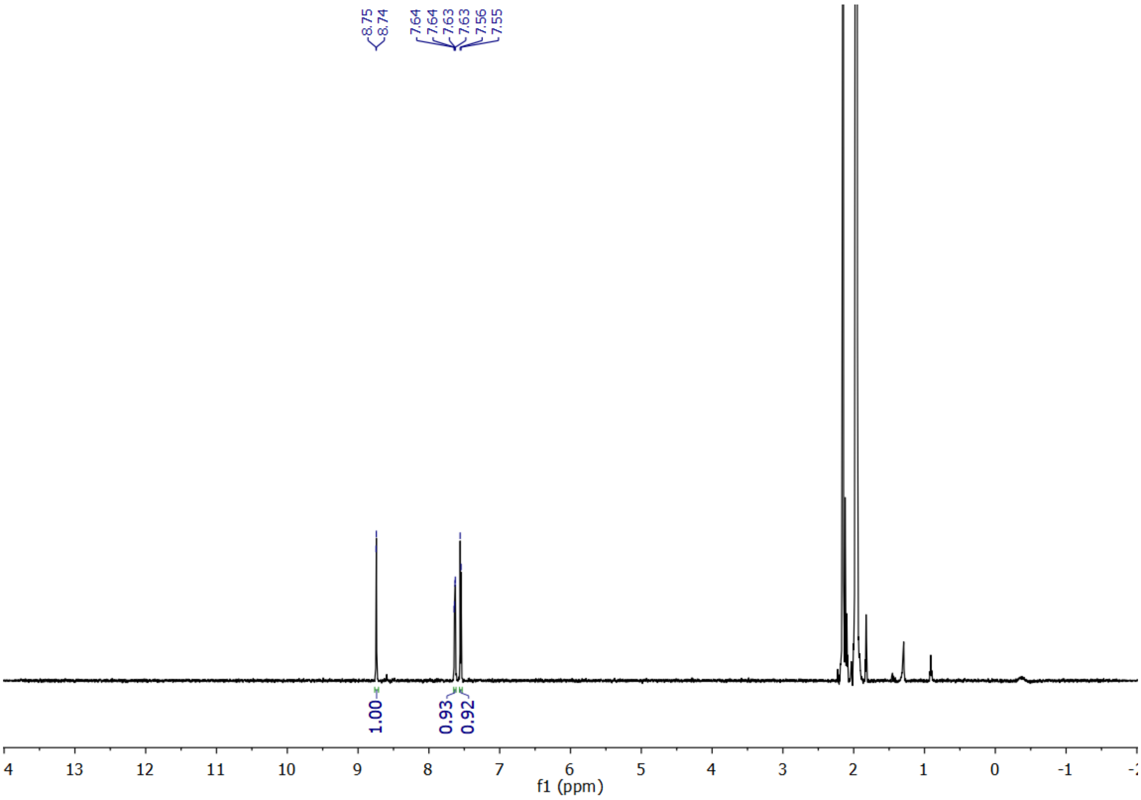

Figure S 19. Full ^{1}H-NMR (298 K, 500 MHz, Acetonitrile-d_3) spectrum of **Ru3Br·PF$_6$**.

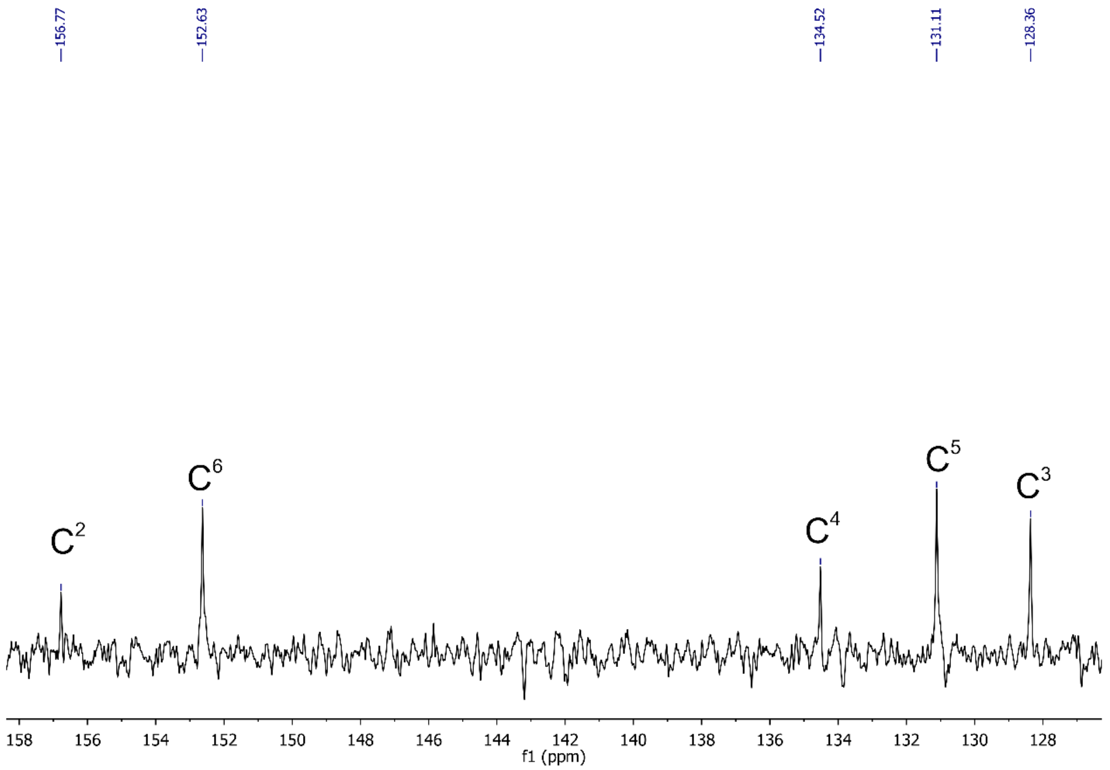

Figure S 20. $^{13}C\{^{1}H\}$-NMR (298 K, 101 MHz, Acetonitrile-d_3) spectrum of **Ru3Br·PF₆**.

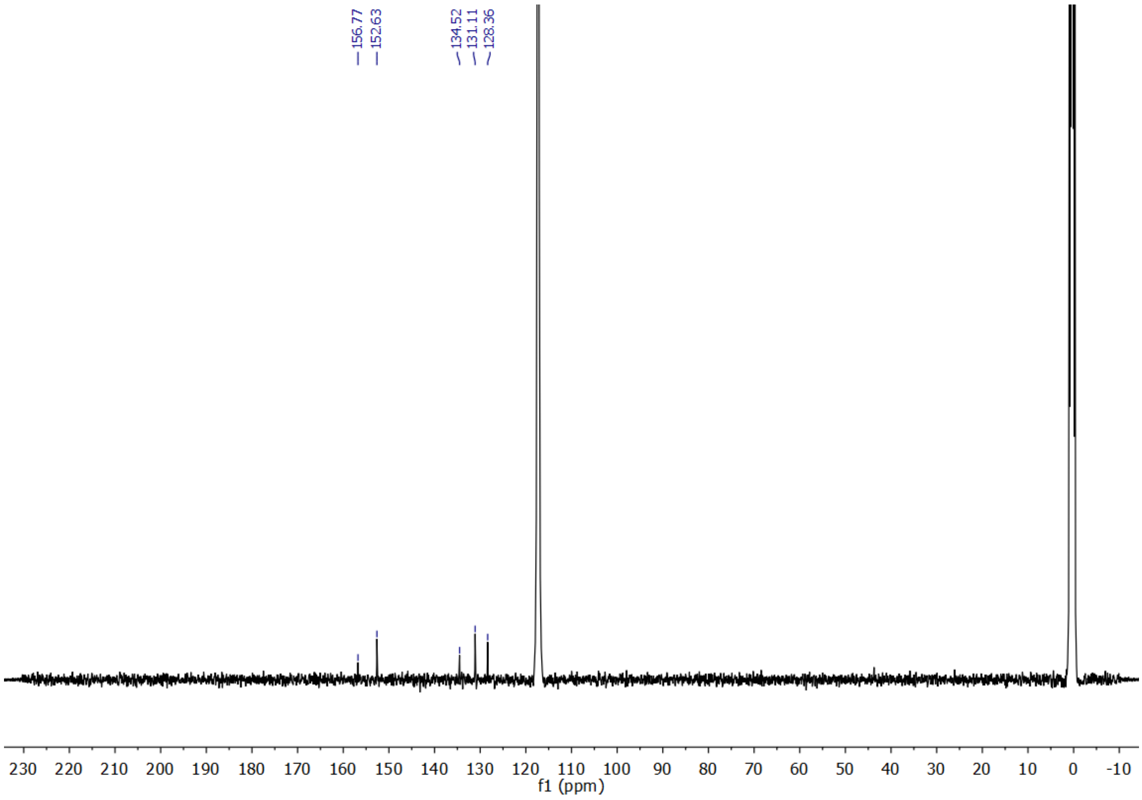

Figure S 21. Full $^{13}C\{^{1}H\}$-NMR (298 K, 101 MHz, Acetonitrile-d_3) spectrum of **Ru3Br·PF₆**.

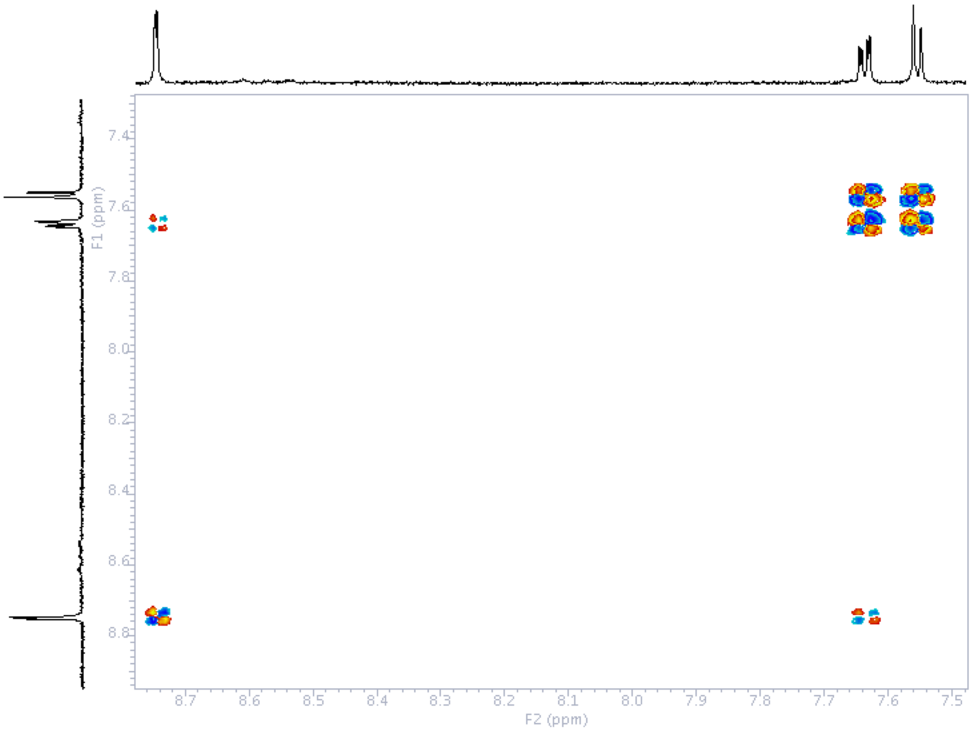

Figure S 22. ^{1}H-^{1}H gDQCOSY (298 K, 500 MHz, Acetonitrile-d_3) spectrum of compound **Ru3Br·PF$_6$**.

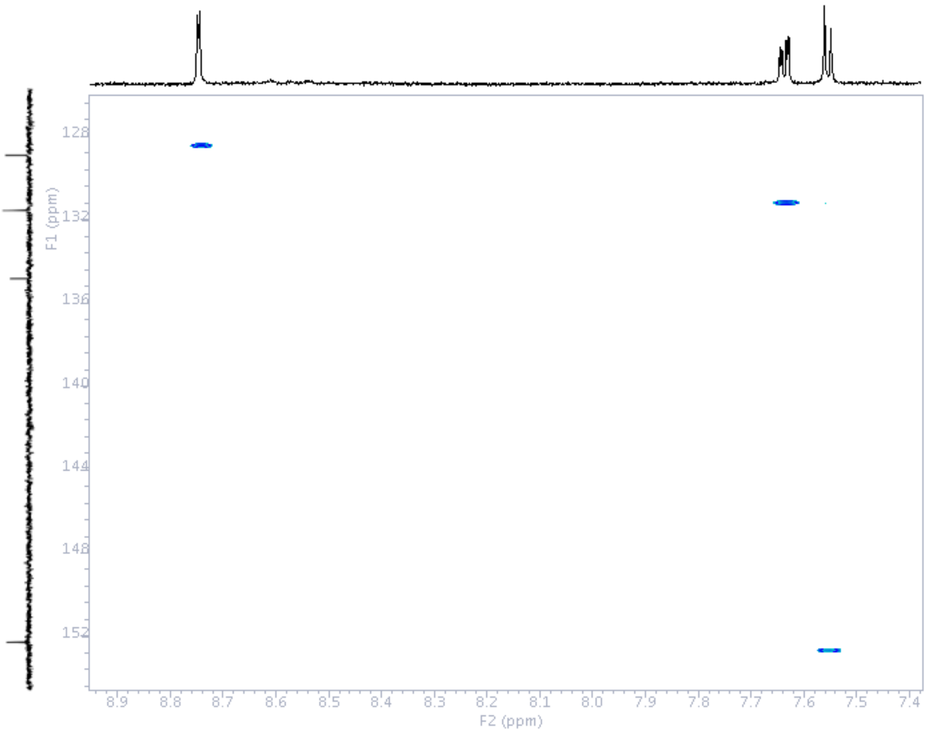

Figure S 23. ^{1}H-^{13}C bsgHSQC (298 K, 500 MHz, Acetonitrile-d_3) spectrum of compound **Ru3Br·PF$_6$**.

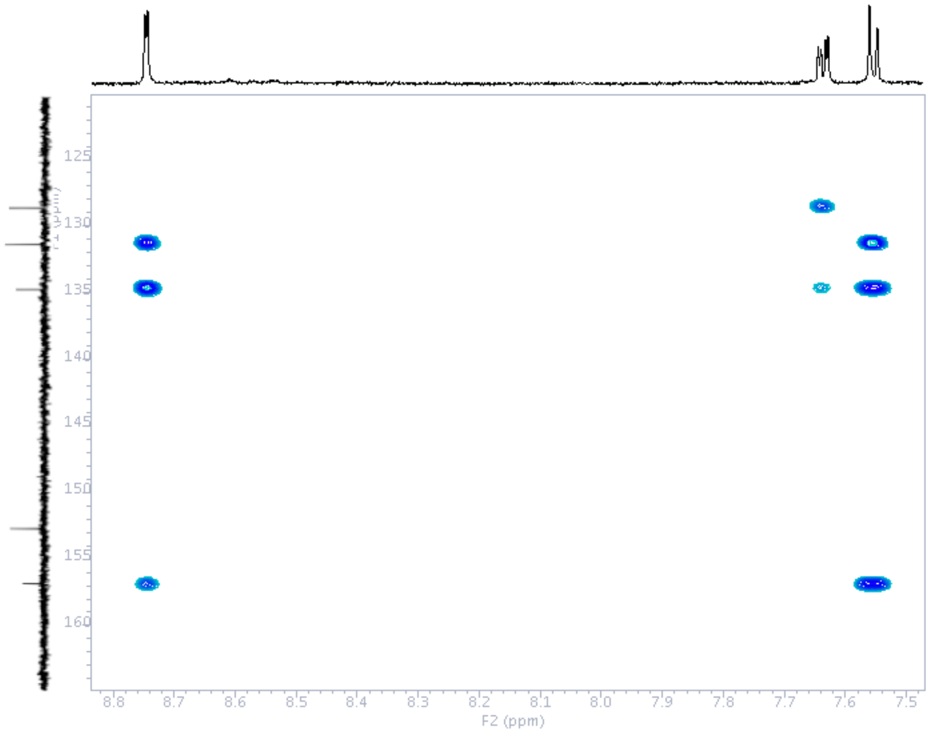

Figure S 24. ^{1}H-^{13}C gc2HMBC (298 K, 500 MHz, Acetonitrile-*d*$_3$) spectrum of compound **Ru3Br·PF$_6$**.

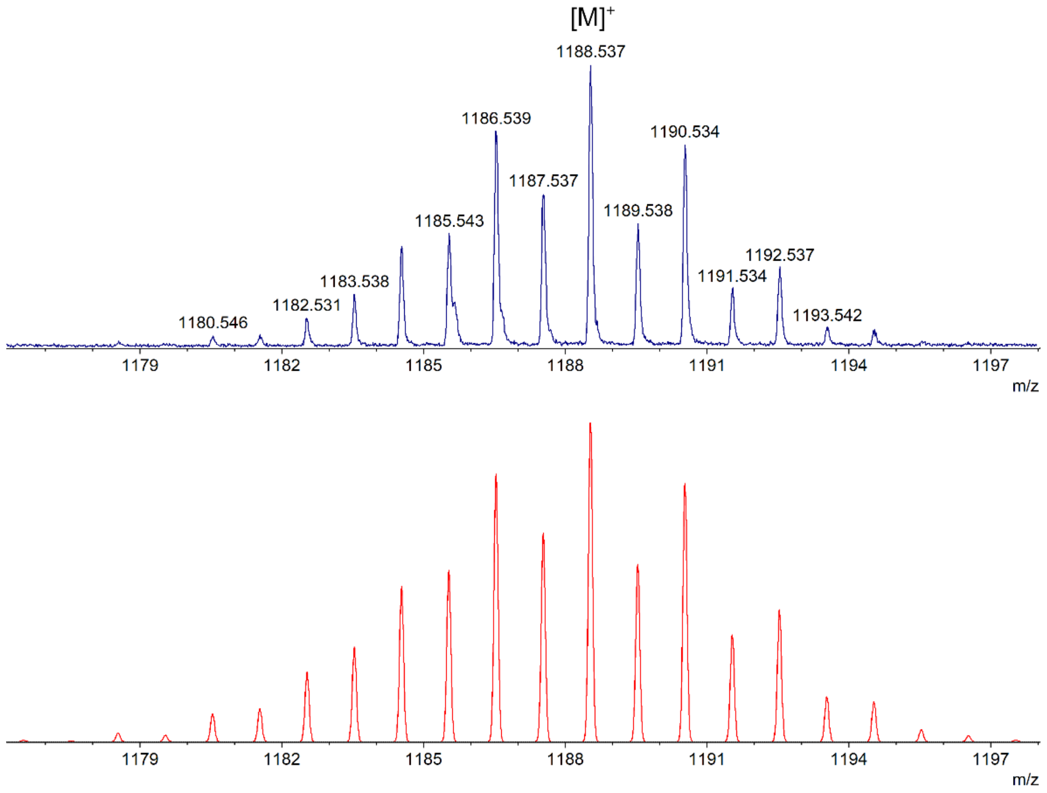

Figure S 25. HRMS (MALDI) of **Ru3Br·PF$_6$**, [Ru3Br + PF$_6$]$^+$**.** Calculated (red), measured (blue).

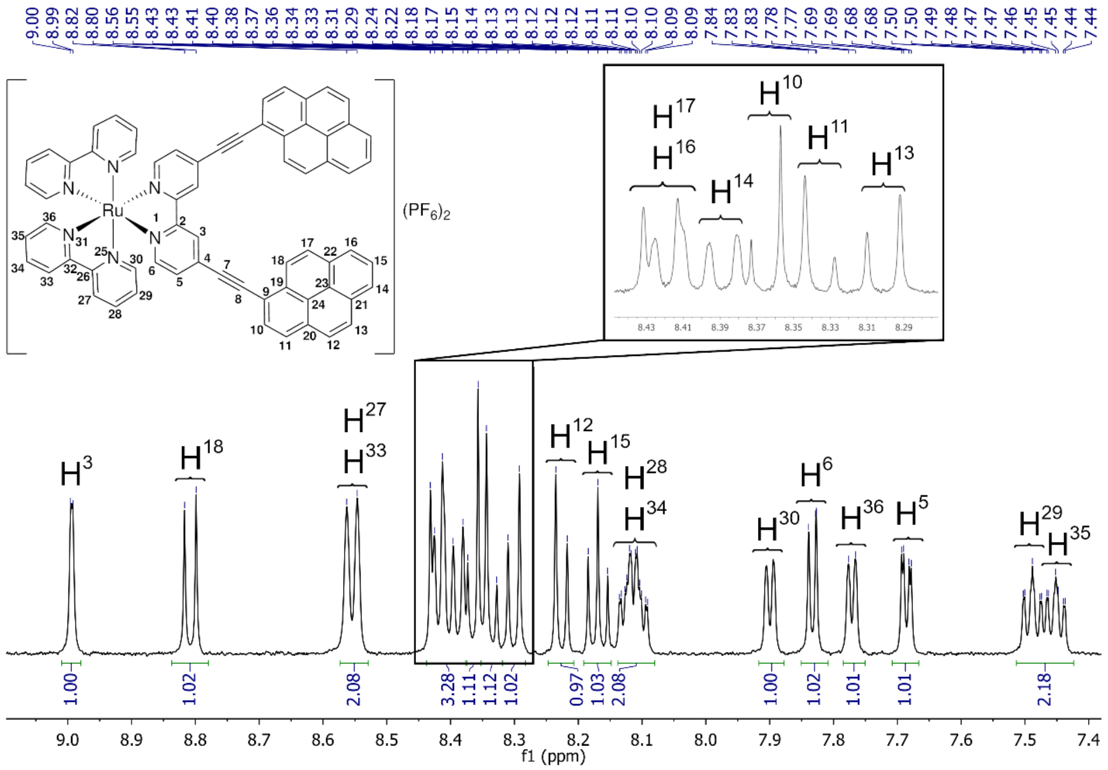

Figure S 26.^{1}H-NMR (298 K, 500 MHz, Acetonitrile-d_3) spectrum of **Ru1P·PF₆.**

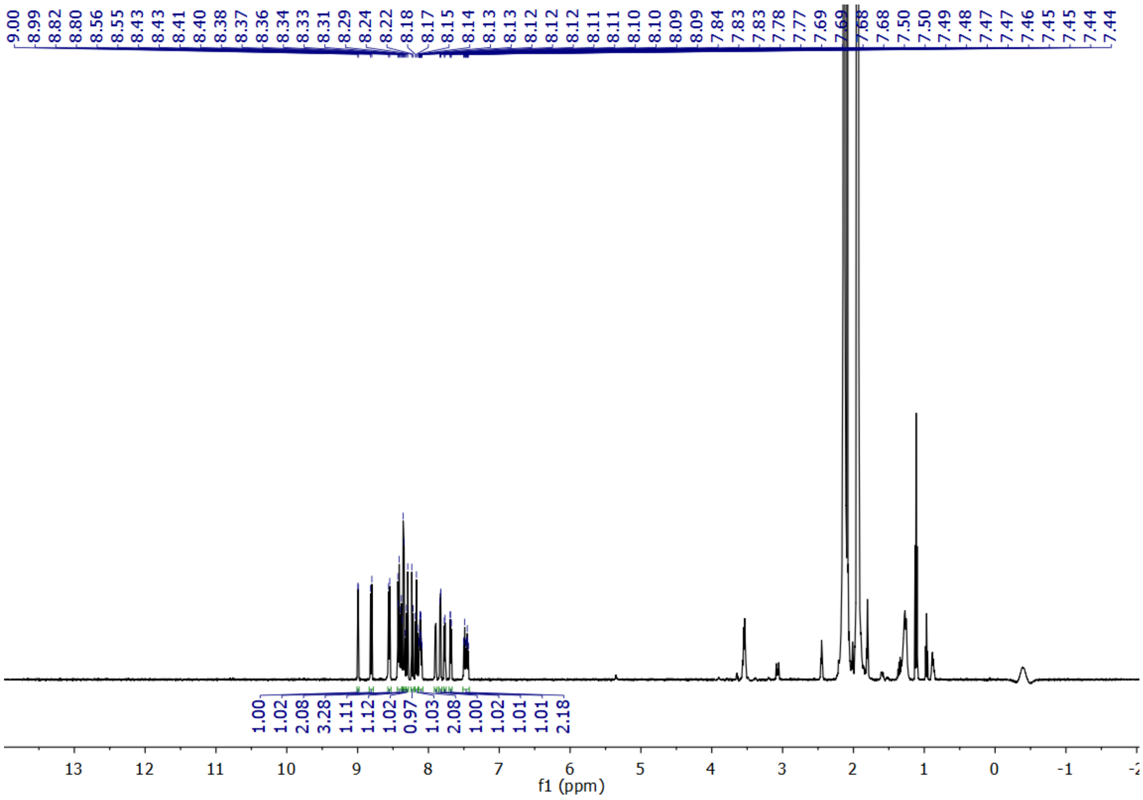

Figure S 27. Full ^{1}H-NMR (298 K, 500 MHz, Acetonitrile-d_3) spectrum of **Ru1P·PF₆.**

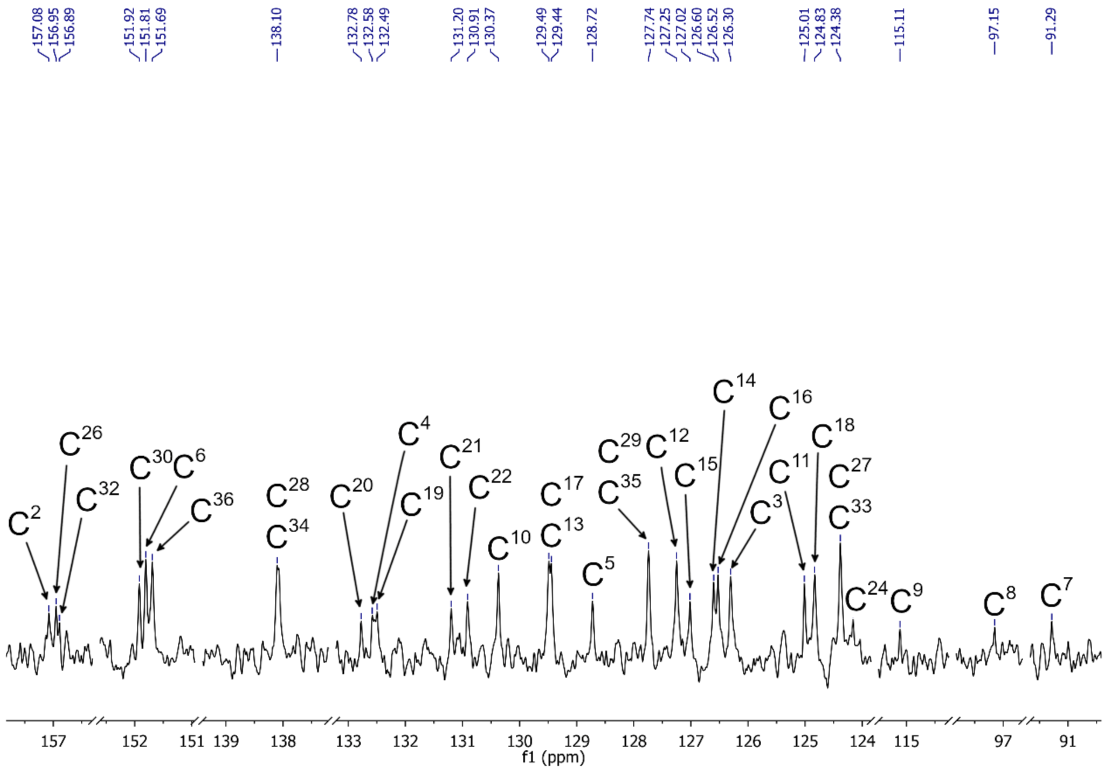

Figure S 28. $^{13}C\{^{1}H\}$-NMR (298 K, 101 MHz, Acetonitrile-d_3) spectrum of **Ru1P·PF₆**.

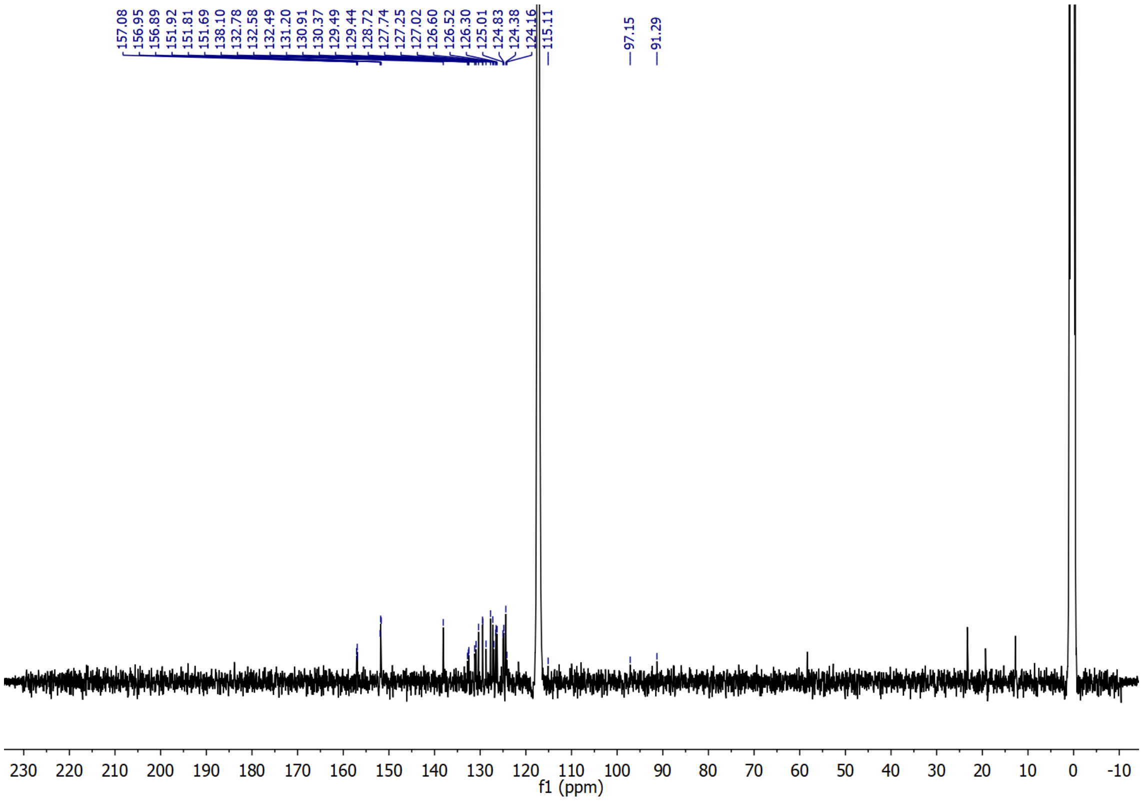

Figure S 29. Full $^{13}C\{^{1}H\}$-NMR (298 K, 101 MHz, Acetonitrile-d_3) spectrum of **Ru1P·PF₆**.

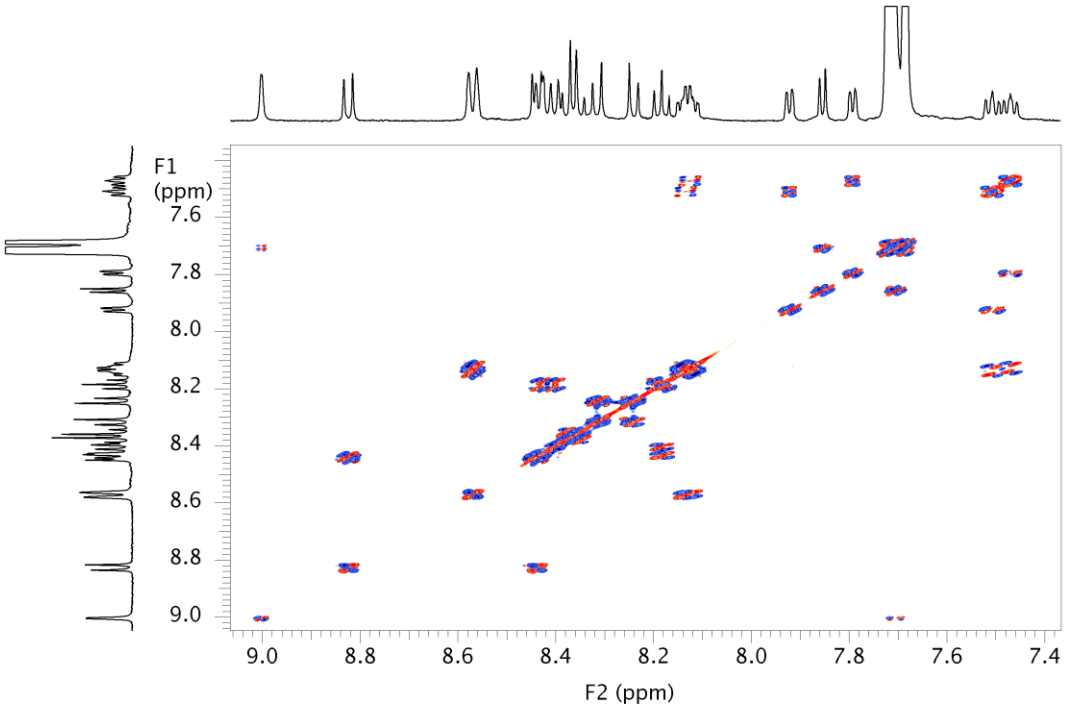

Figure S 30. ^{1}H-^{1}H gDQCOSY (298 K, 500 MHz, Acetonitrile-*d*$_3$) spectrum of compound **Ru1P·PF$_6$**.

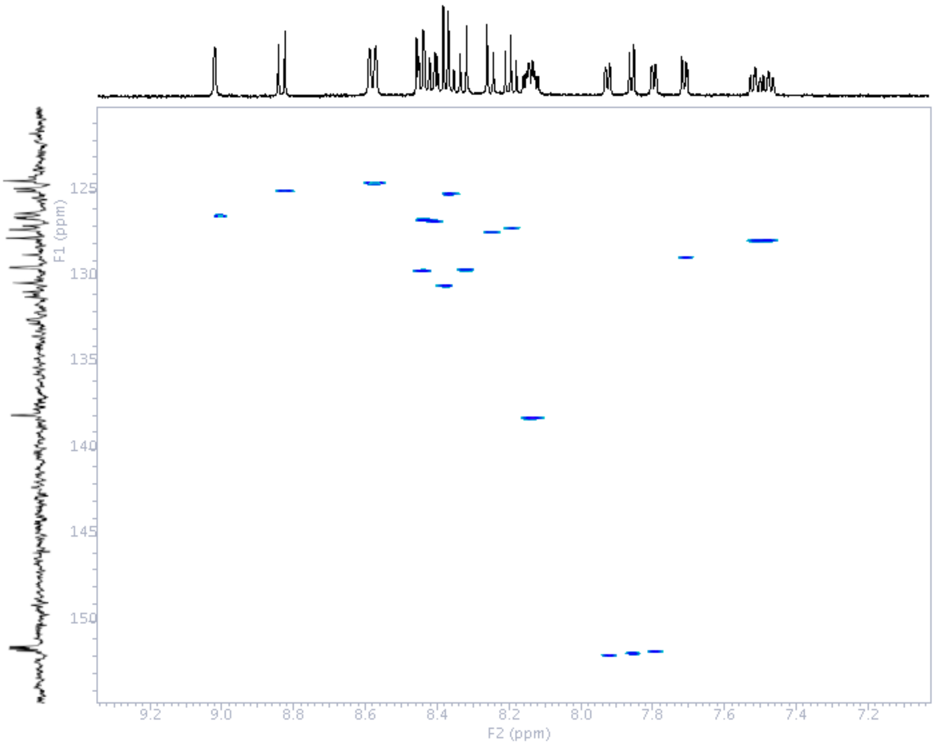

Figure S 31. ^{1}H-^{13}C bsgHSQC (298 K, 500 MHz, Acetonitrile-*d*$_3$) spectrum of compound **Ru1P·PF$_6$**.

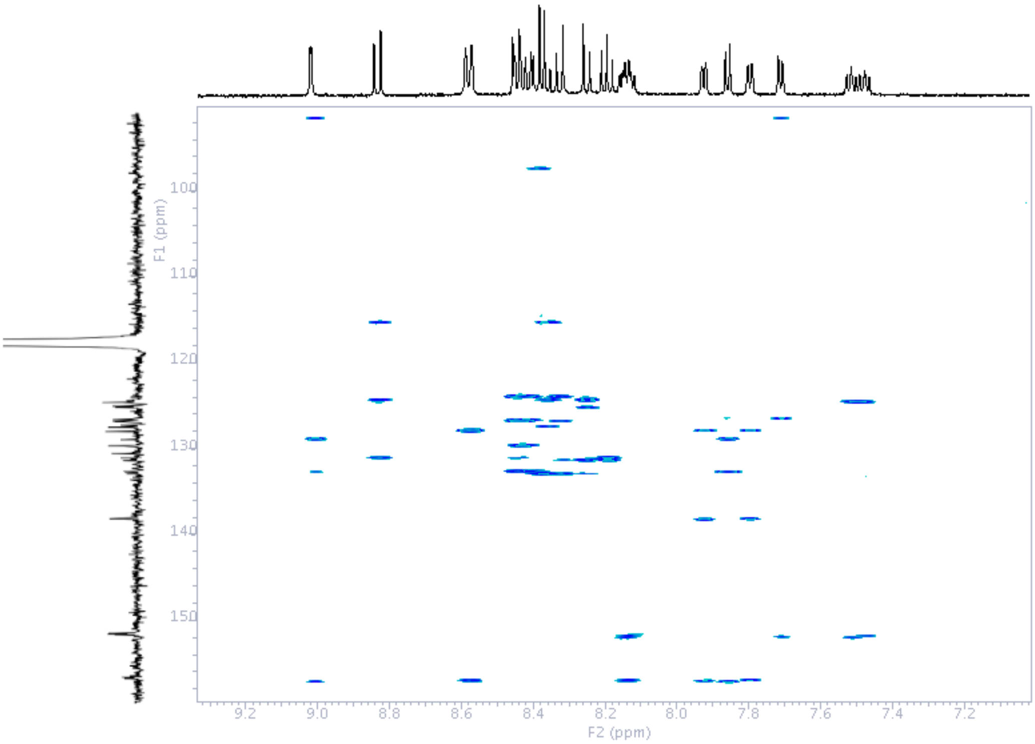

Figure S 32. ^{1}H-^{13}C bsgHMBC (298 K, 500 MHz, Acetonitrile-d_3) spectrum of compound **Ru1P·PF6**.

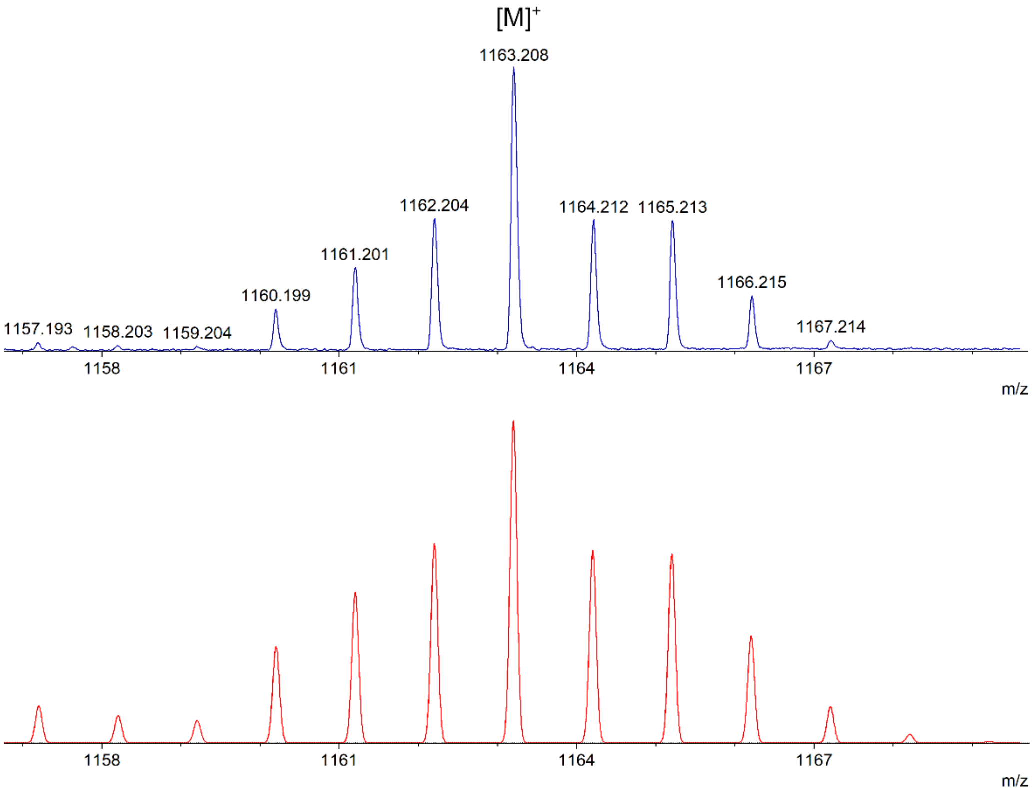

Figure S 33. HRMS (MALDI) of **Ru1P·PF6**, $[Ru1P + PF_6]^+$. Calculated (red), measured (blue).

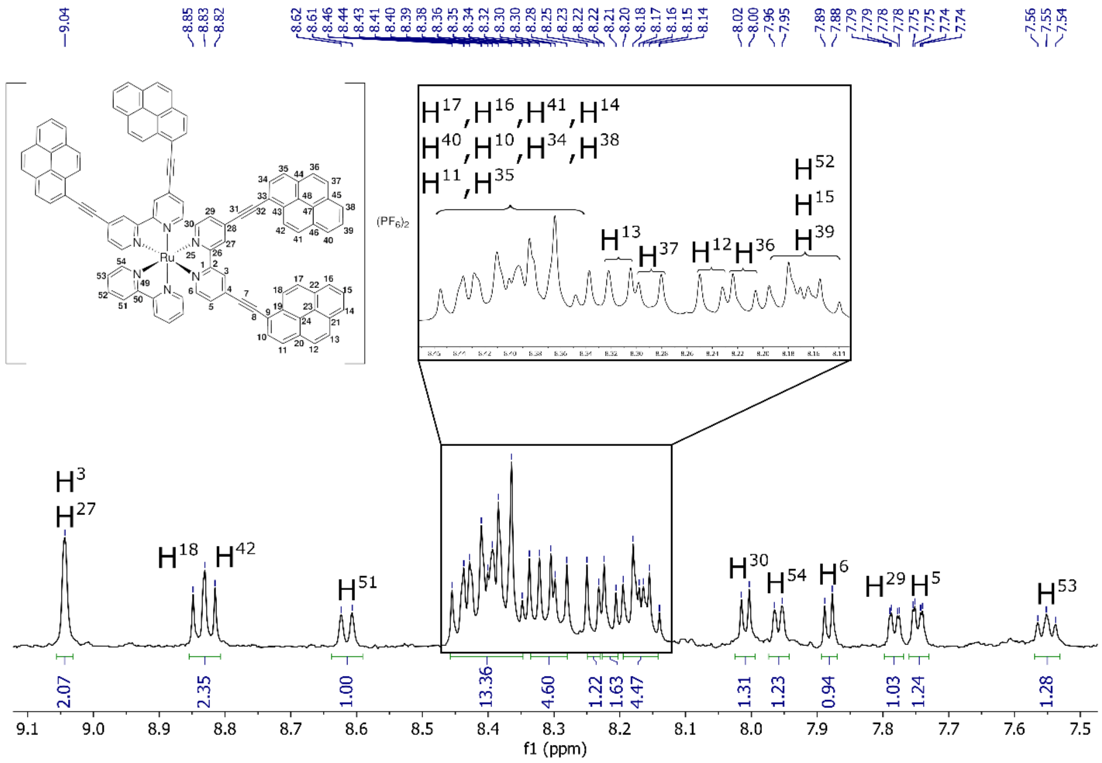

Figure S 34. ^{1}H NMR (298 K, 500 MHz, Acetonitrile-d_3) spectrum of **Ru2P·PF$_6$**.

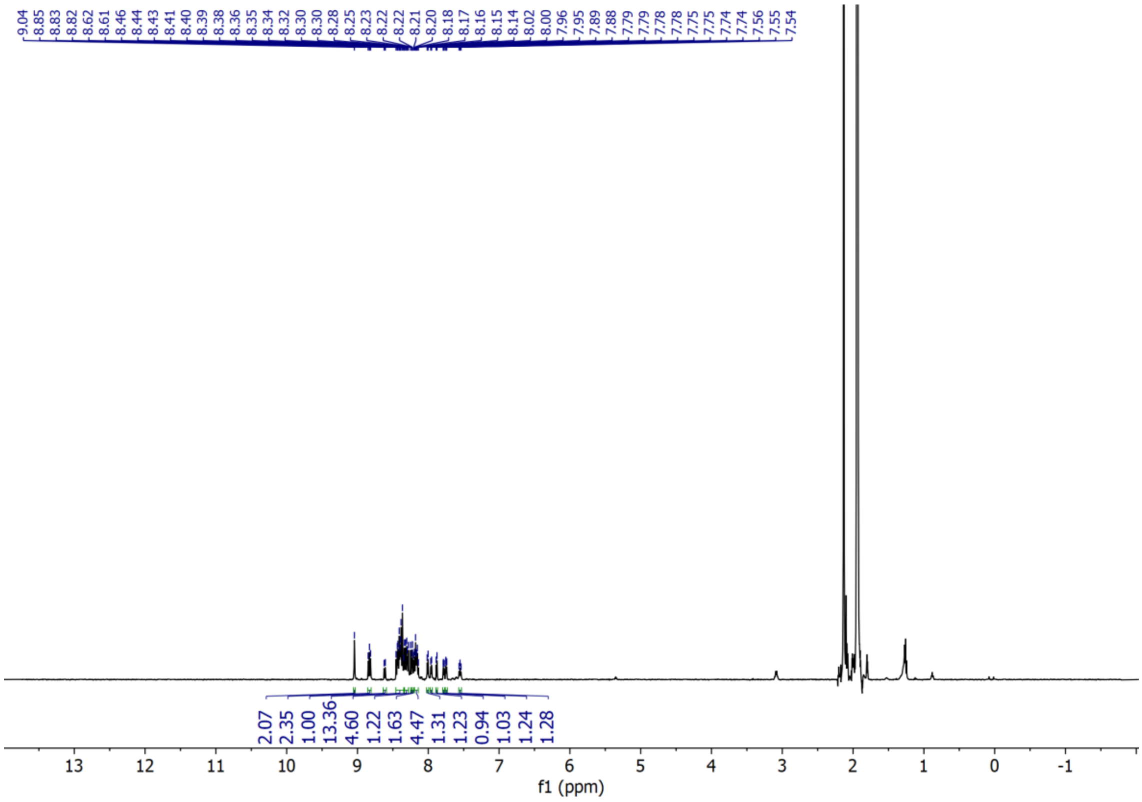

Figure S 35. Full ^{1}H-NMR (298 K, 500 MHz, Acetonitrile-d_3) spectrum of **Ru2P·PF$_6$**.

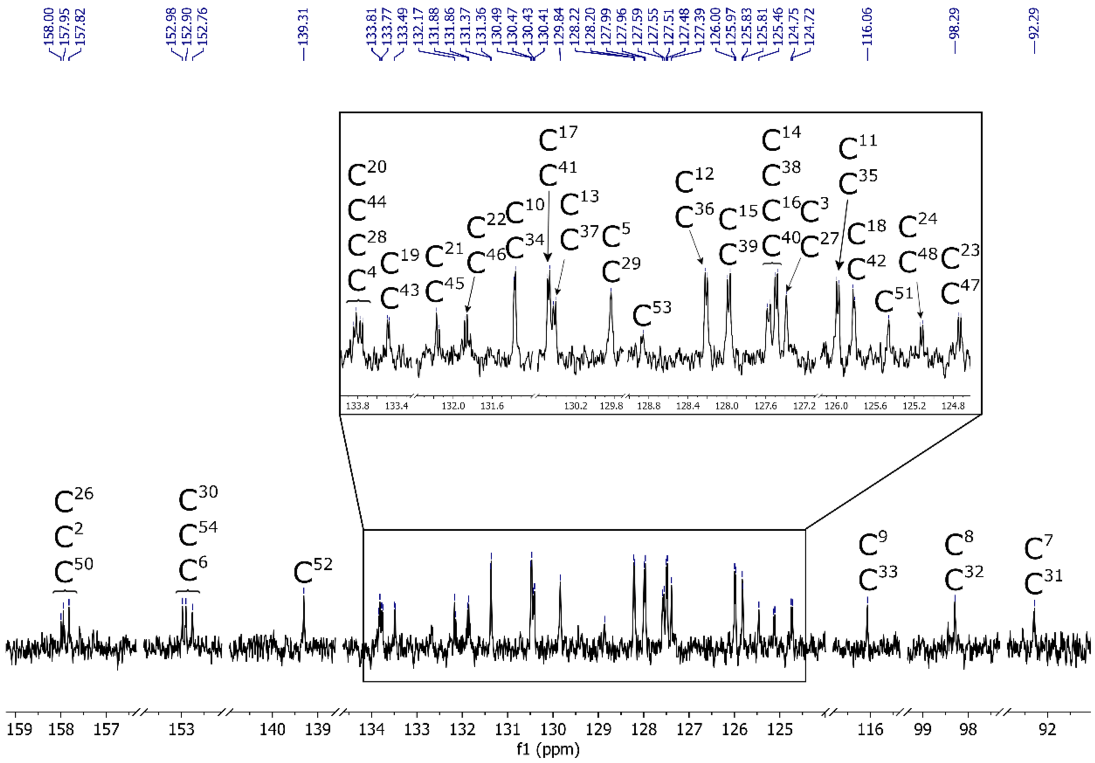

Figure S 36. $^{13}C\{^{1}H\}$ NMR (298 K, 126 MHz, Acetonitrile-d_3) spectrum of **Ru2P·PF$_6$**.

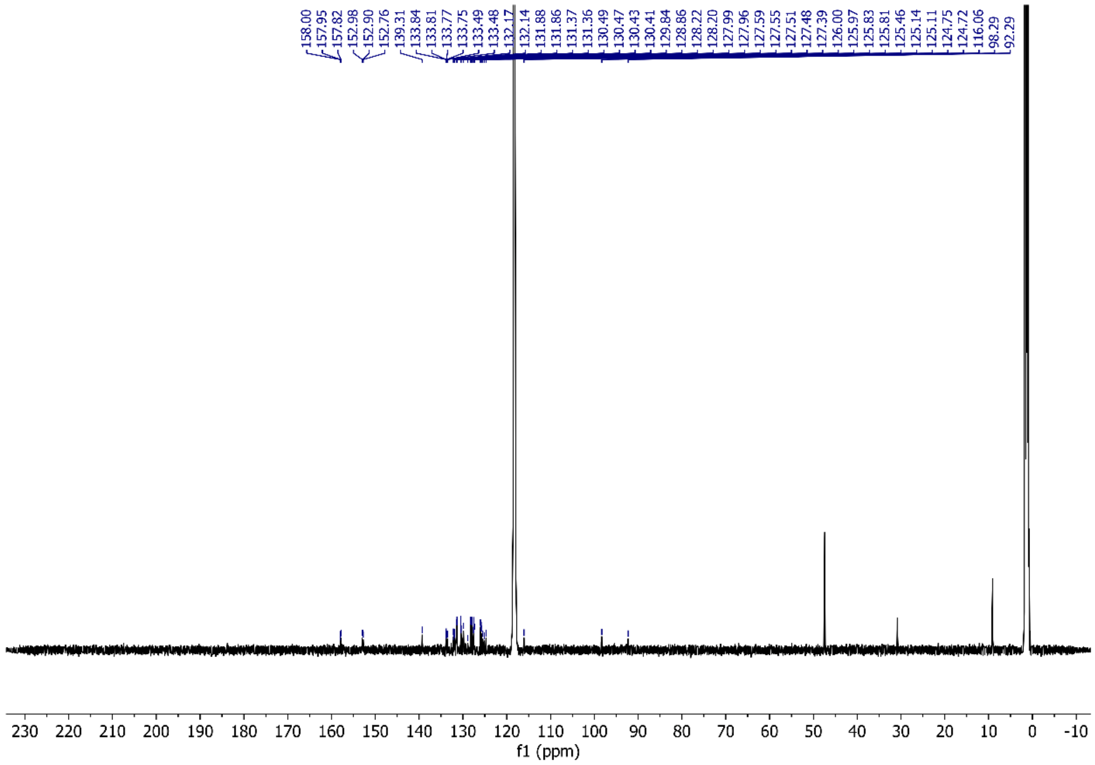

Figure S 37. Full $^{13}C\{^{1}H\}$-NMR (298 K, 126 MHz, Acetonitrile-d_3) spectrum of **Ru2P·PF$_6$**.

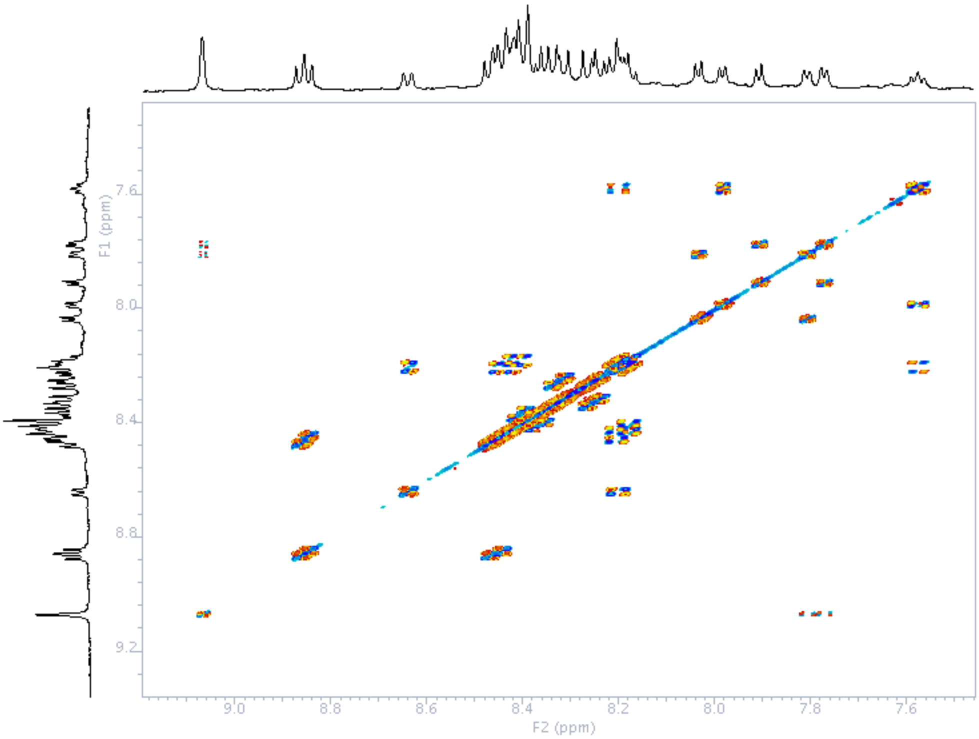

Figure S 38. ^{1}H-^{1}H gDQCOSY (298 K, 500 MHz, Acetonitrile-d_3) spectrum of compound **Ru2P·PF$_6$**.

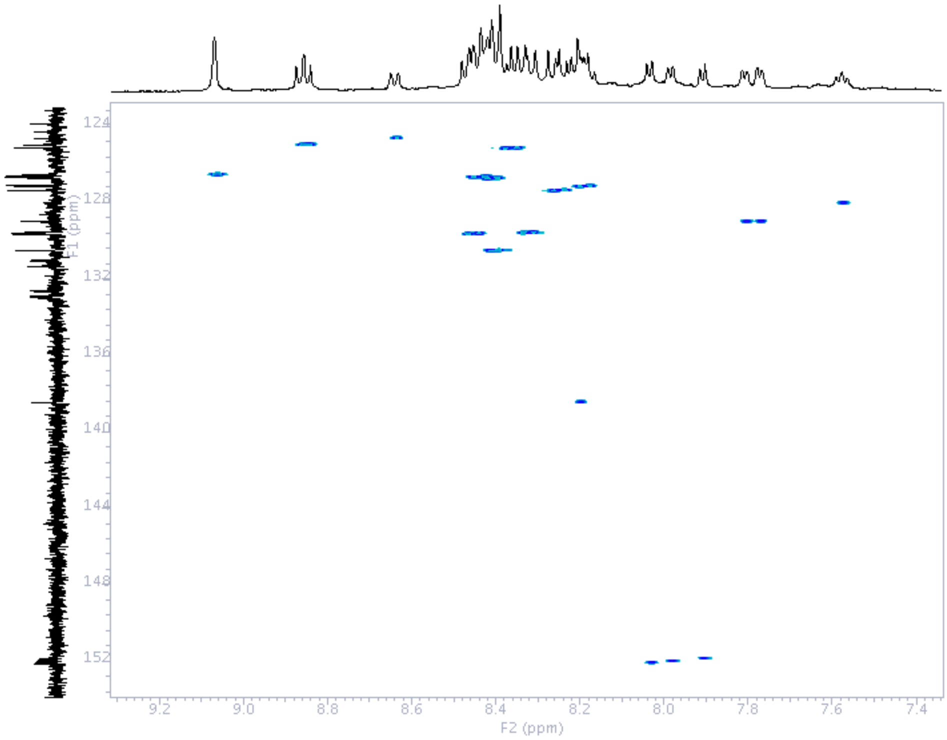

Figure S 39. ^{1}H-^{13}C gHSQCAD_PS (298 K, 500 MHz, Acetonitrile-d_3) spectrum of compound **Ru2P·PF$_6$**.

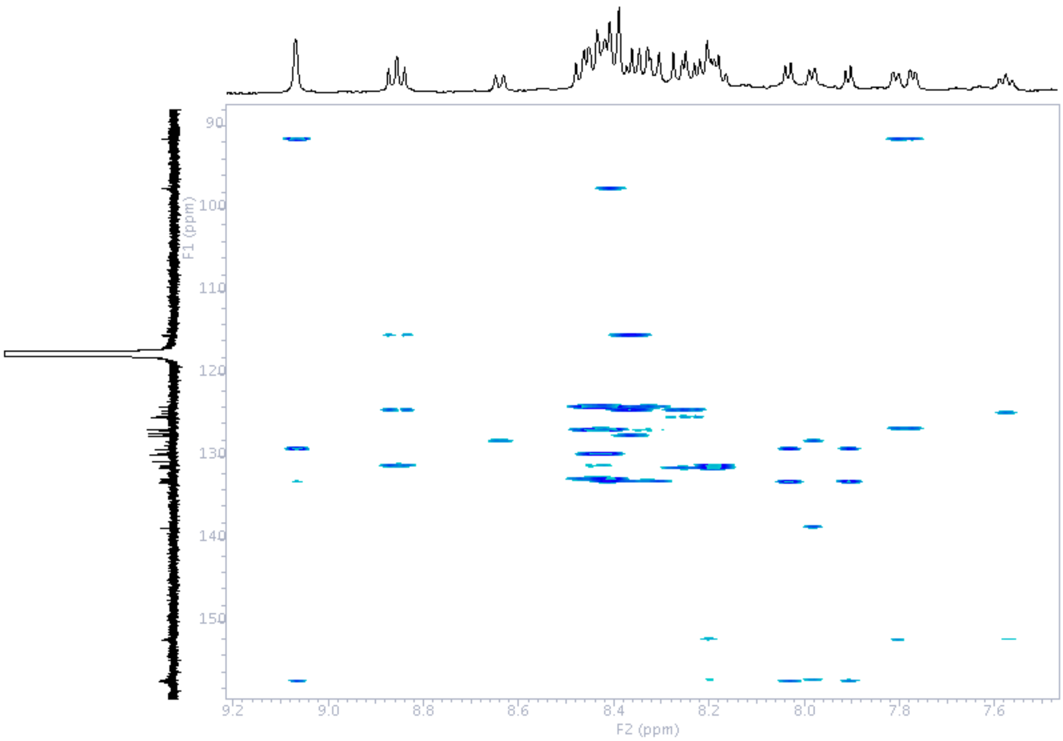

Figure S 40. ^{1}H-^{13}C bsgHMBC (298 K, 500 MHz, Acetonitrile-*d*$_3$) spectrum of compound **Ru2P·PF$_6$**.

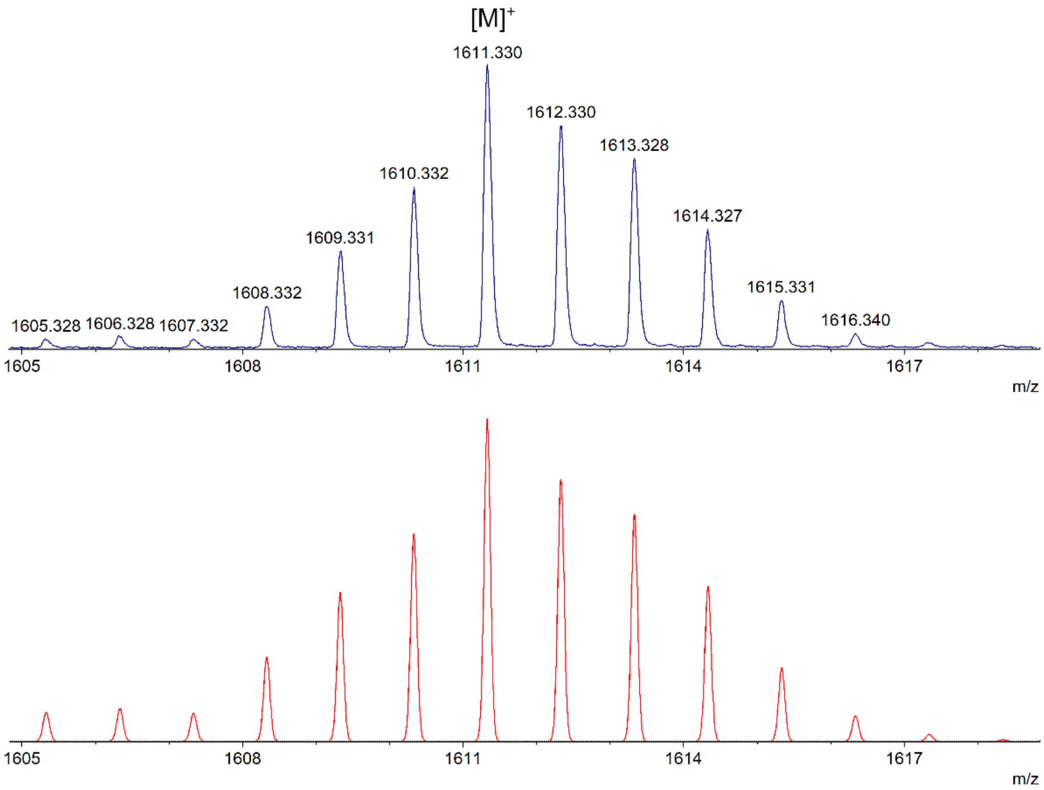

Figure S 41.HRMS (MALDI) of **Ru2P·PF$_6$**, $[Ru2P + PF_6]^+$. Calculated (red), measured (blue).

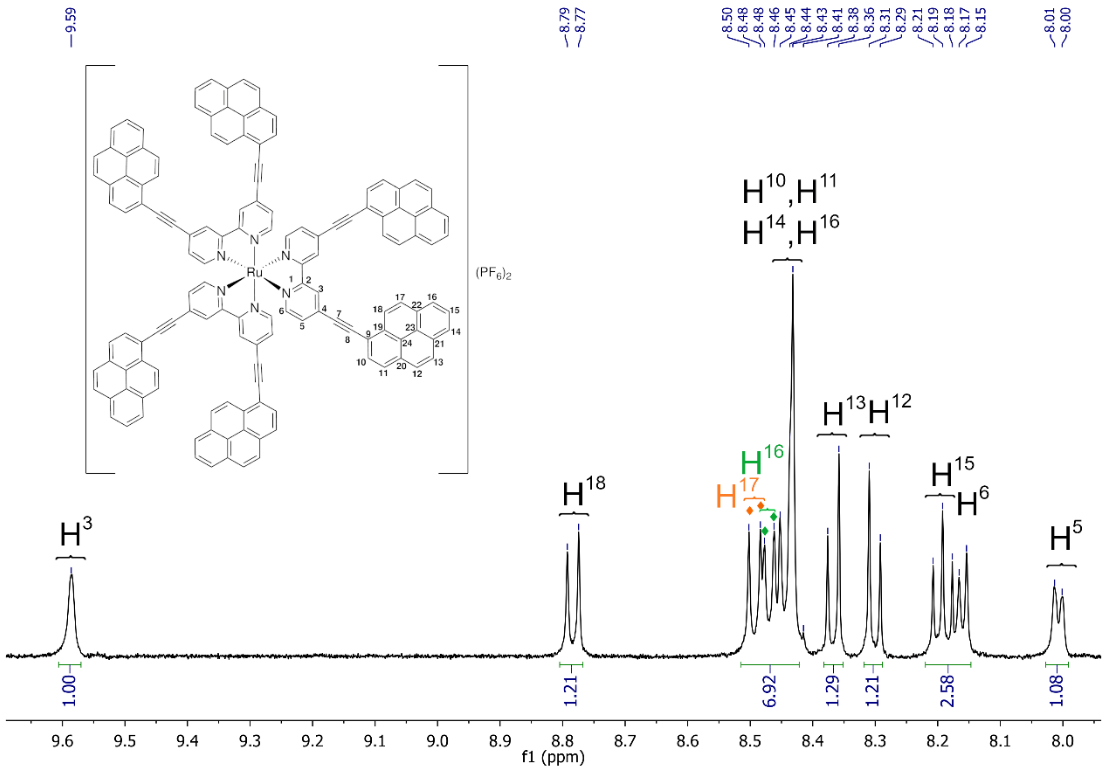

Figure S 42. ^{1}H-NMR (298 K, 500 MHz, DMSO-d_6) spectrum of **Ru3P·PF6**.

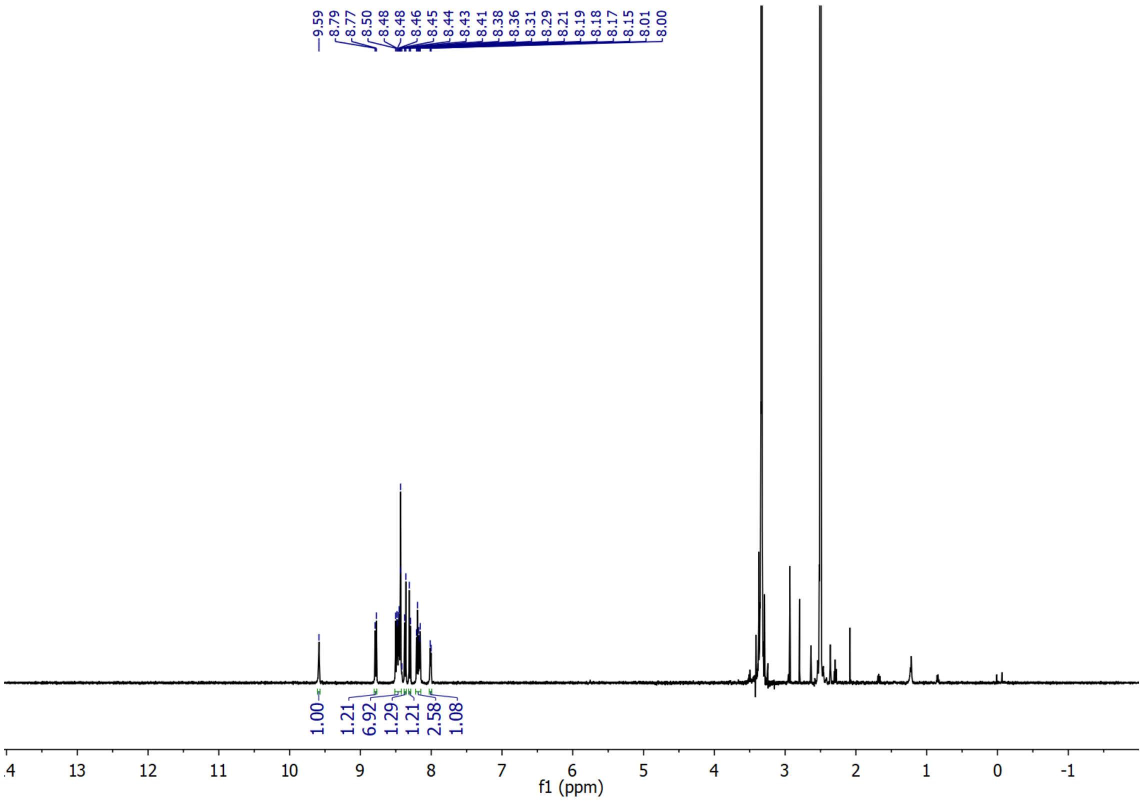

Figure S 43. Full ^{1}H-NMR (298 K, 500 MHz, DMSO-d_6) spectrum of **Ru3P·PF6**.

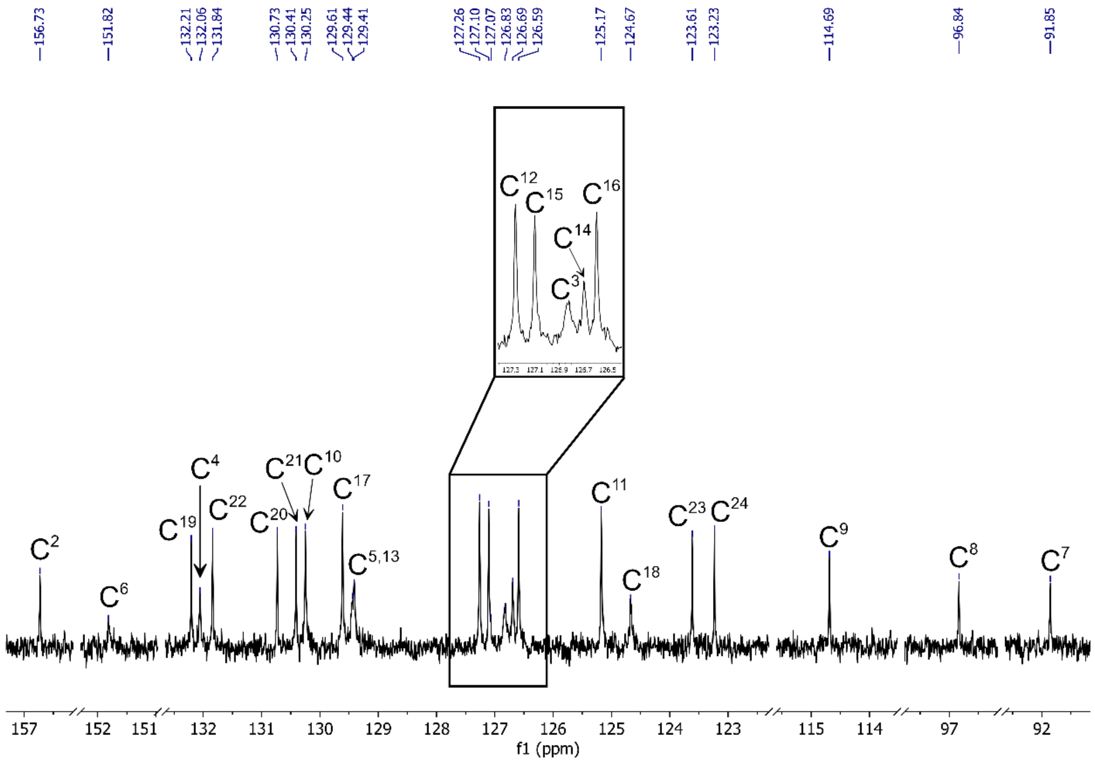

Figure S 44. $^{13}C\{^1H\}$-NMR (298 K, 126 MHz, DMSO-d_6) spectrum of **Ru3P·PF$_6$**.

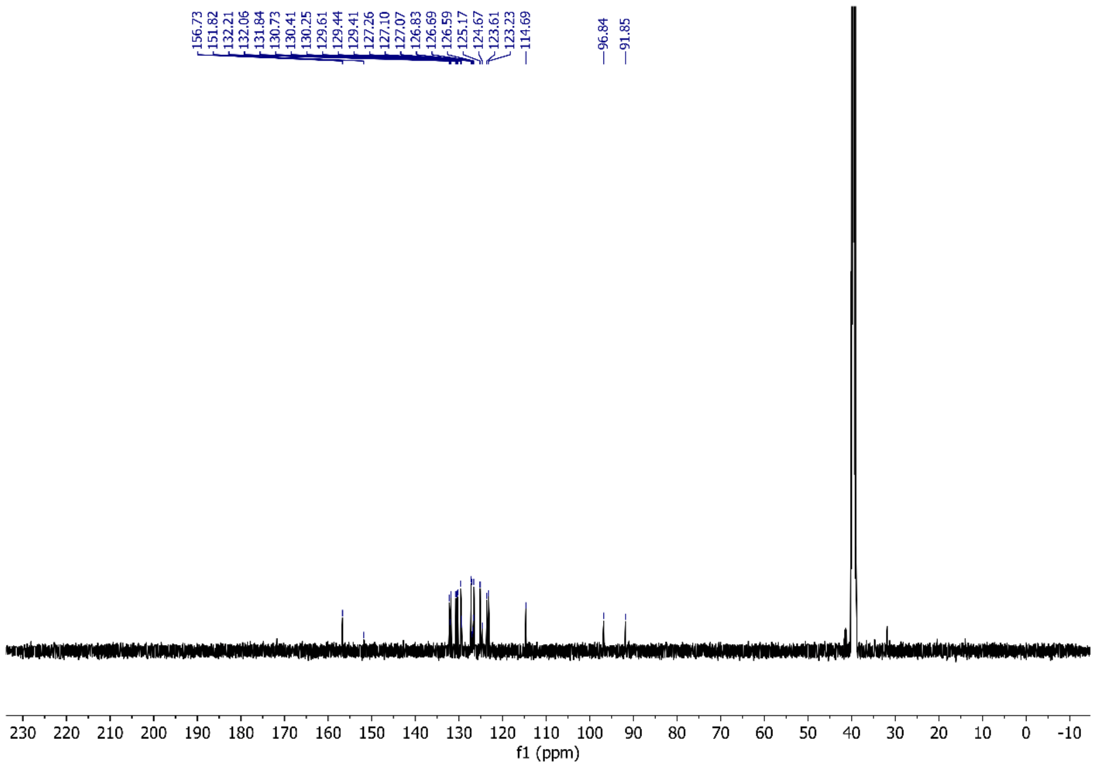

Figure S 45. Full $^{13}C\{^1H\}$-NMR (298 K, 126 MHz, DMSO-d_6) spectrum of **Ru3P·PF$_6$**.

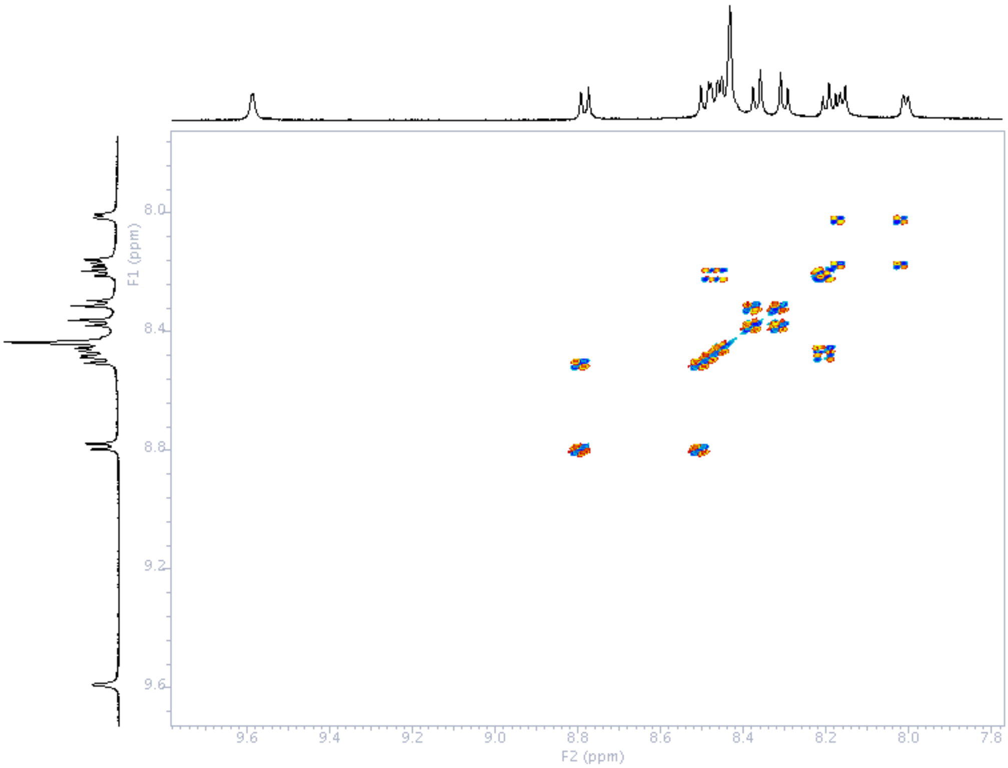

Figure S 46. ^{1}H-^{1}H gDQCOSY (298 K, 500 MHz, DMSO-d_6) spectrum of compound **Ru3P·PF$_6$**.

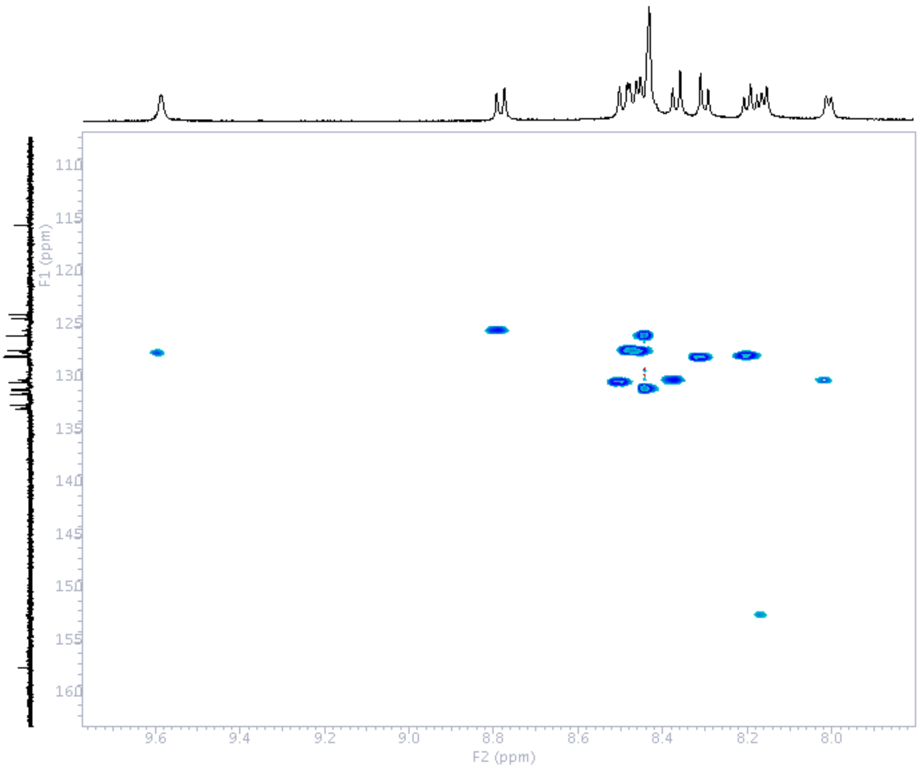

Figure S 47. ^{1}H-^{13}C gc2HSQC (298 K, 500 MHz, DMSO-d_6) spectrum of compound **Ru3P·PF$_6$**.

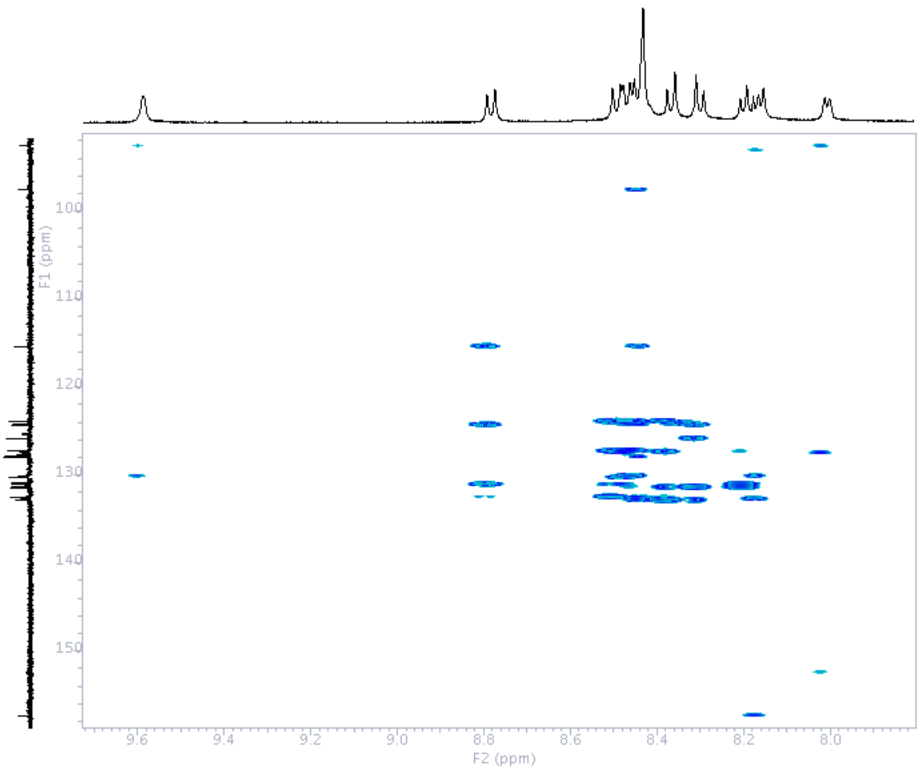

Figure S 48. ^{1}H-^{13}C bsgHMBC (298 K, 500 MHz, DMSO-d_6) spectrum of compound **Ru3P·PF$_6$**.

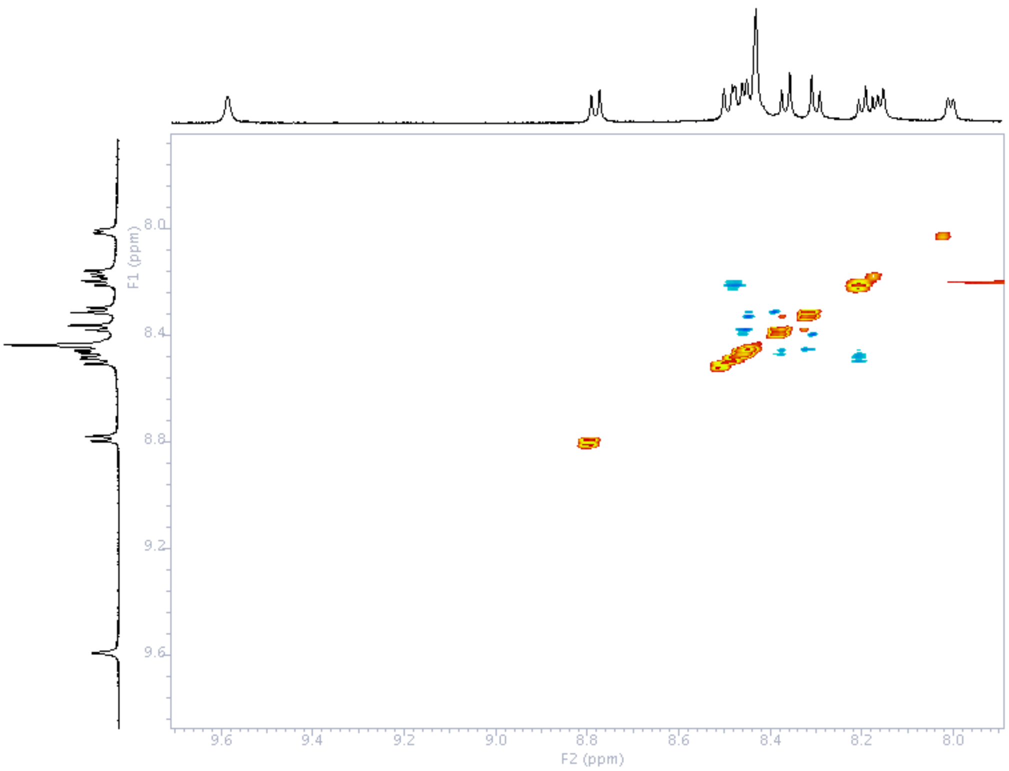

Figure S 49. ^{1}H-^{1}H ROESYAD (298 K, 500 MHz, DMSO-d_6) spectrum of compound **Ru3P·PF$_6$**.

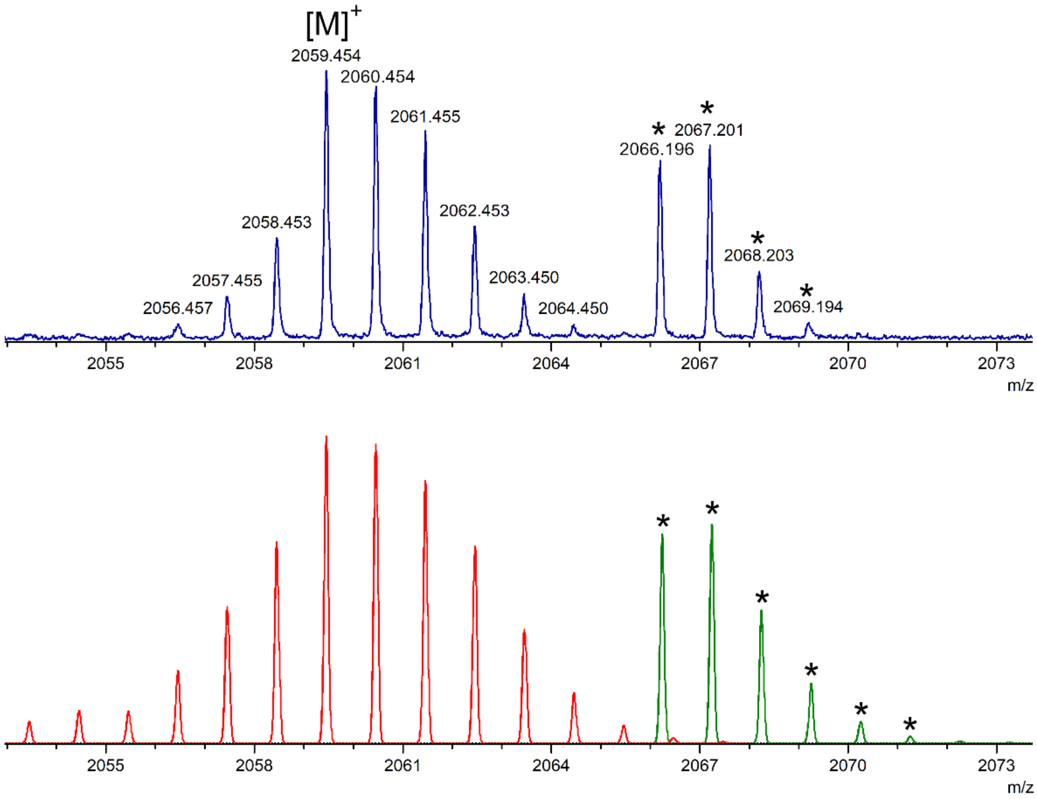

Figure S 50. HRMS (MALDI) of **Ru3P·PF₆**, [Ru3P + PF₆]⁺. Calculated (red), measured (blue). *Signals correspond to calibration standard (PEG, in green for calculated spectrum).

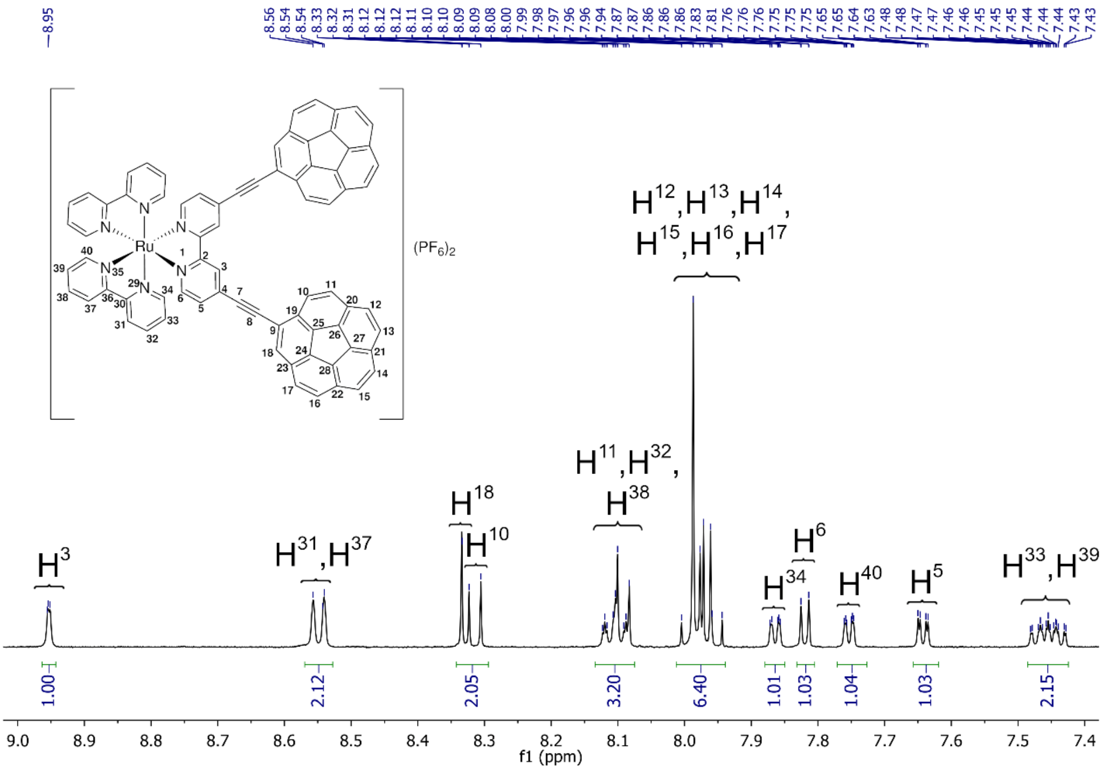

Figure S 51. ¹H-NMR (298 K, 500 MHz, Acetonitrile-*d*₃) spectrum of **Ru1C·PF₆**.

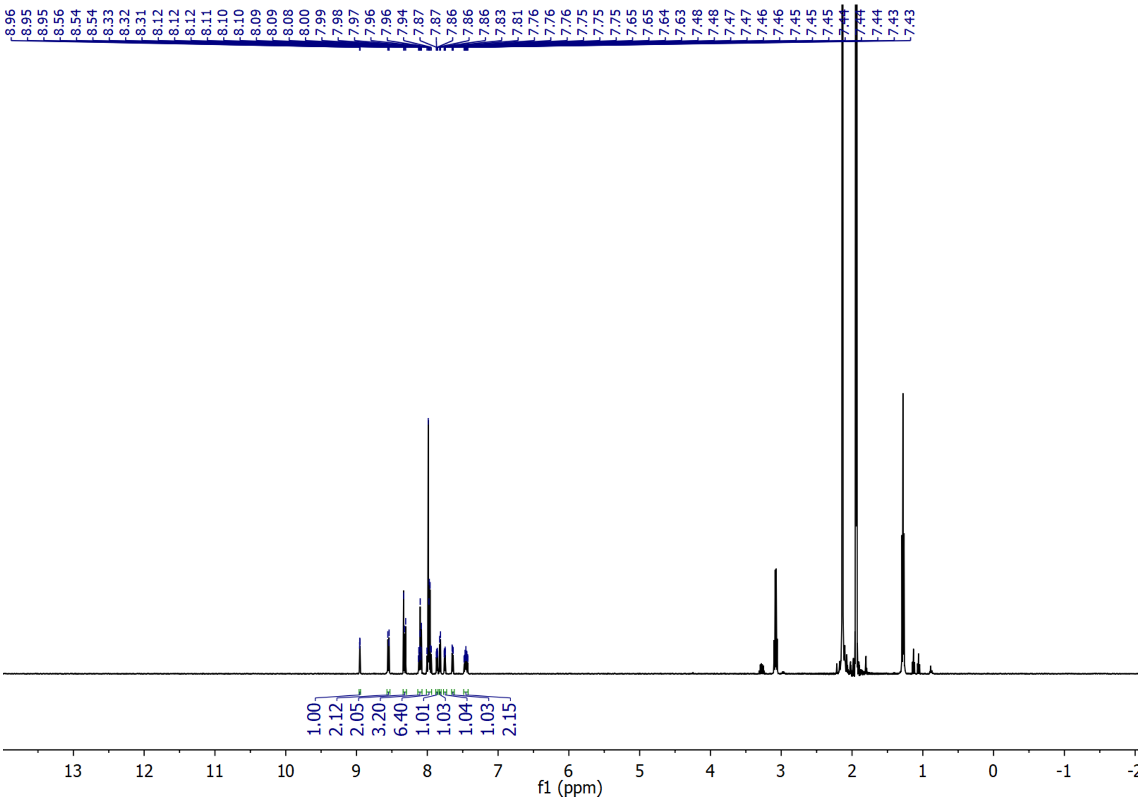

Figure S 52. Full ^{1}H-NMR (298 K, 500 MHz, Acetonitrile-d_3) spectrum of **Ru1C·PF$_6$**.

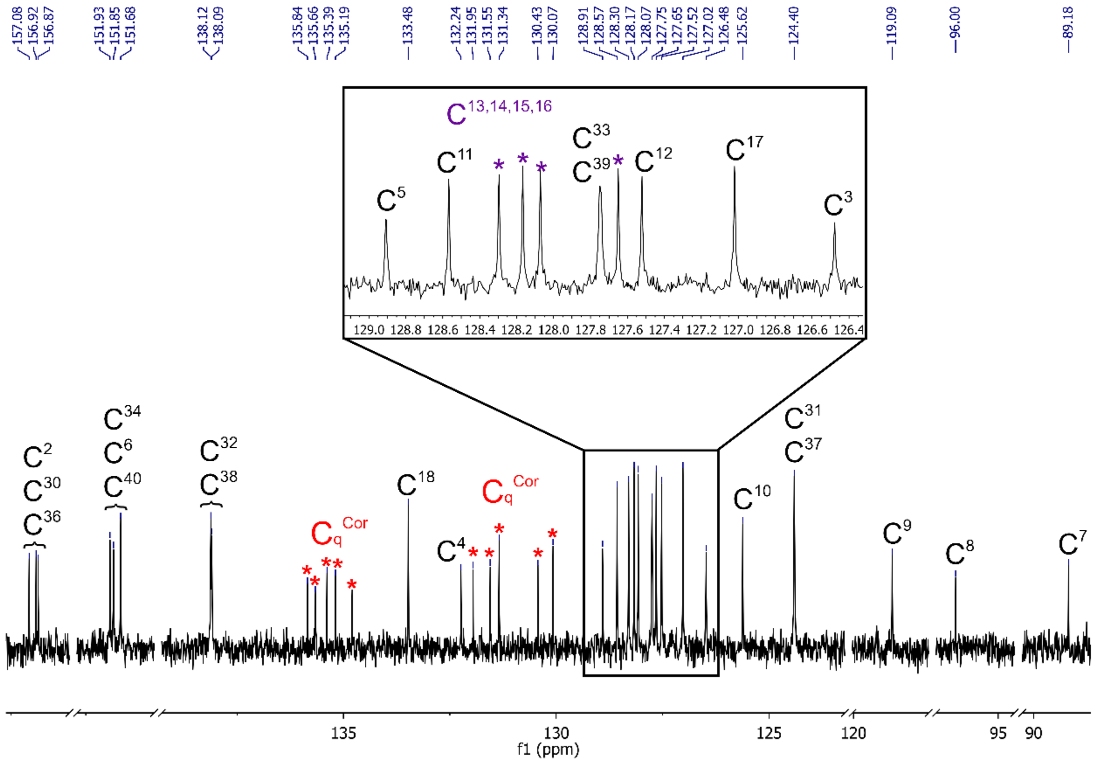

Figure S 53. $^{13}C\{^{1}H\}$-NMR (298 K, 126 MHz, Acetonitrile-d_3) spectrum of **Ru1C·PF$_6$**.

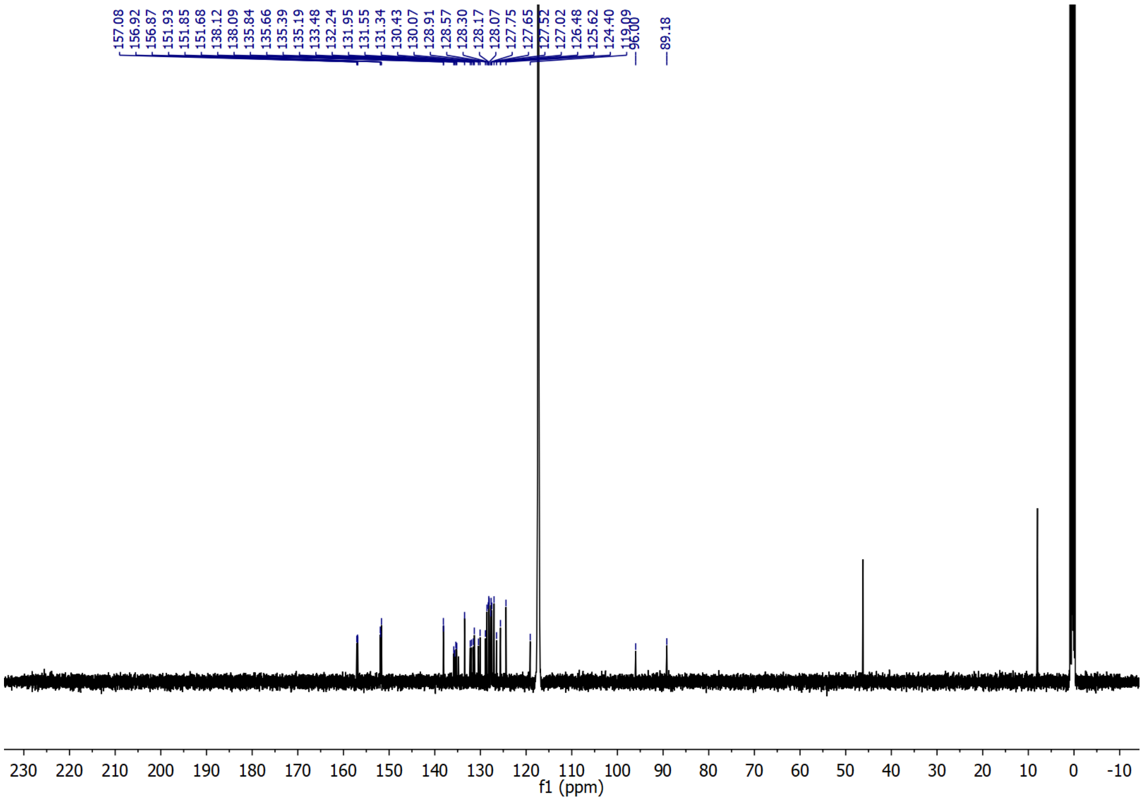

Figure S 54. Full $^{13}C\{^{1}H\}$-NMR (298 K, 126 MHz, Acetonitrile-d_3) spectrum of **Ru1C·PF$_6$.**

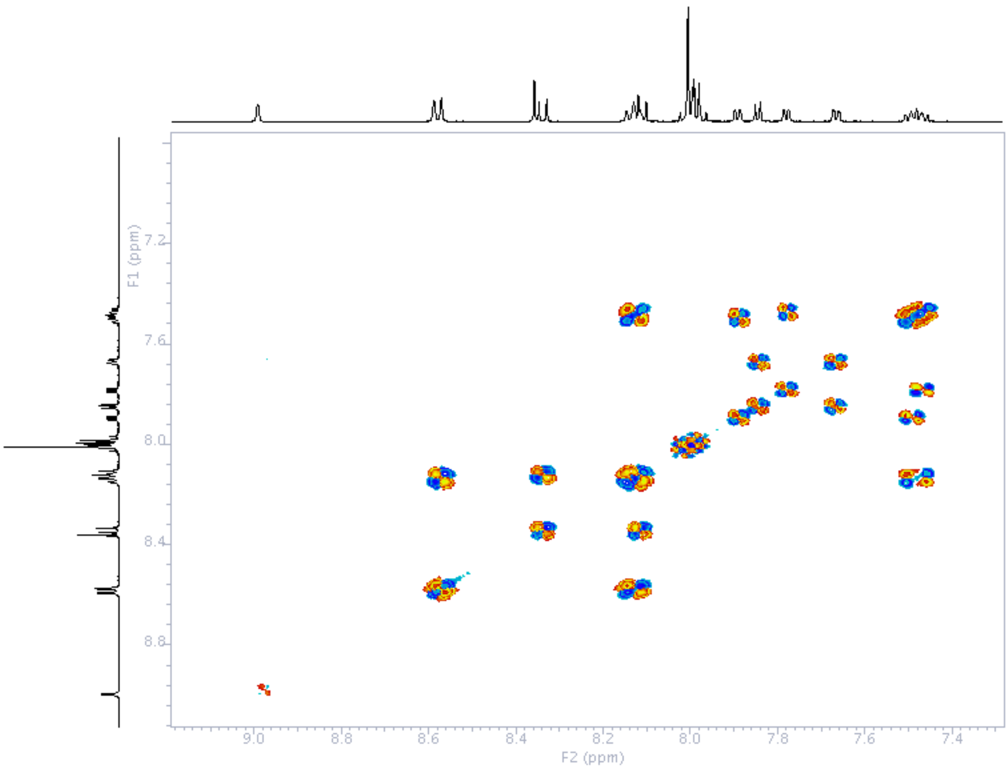

Figure S 55. ^{1}H-^{1}H gDQCOSY (298 K, 500 MHz, Acetonitrile-d_3) spectrum of compound **Ru1C·PF$_6$**.

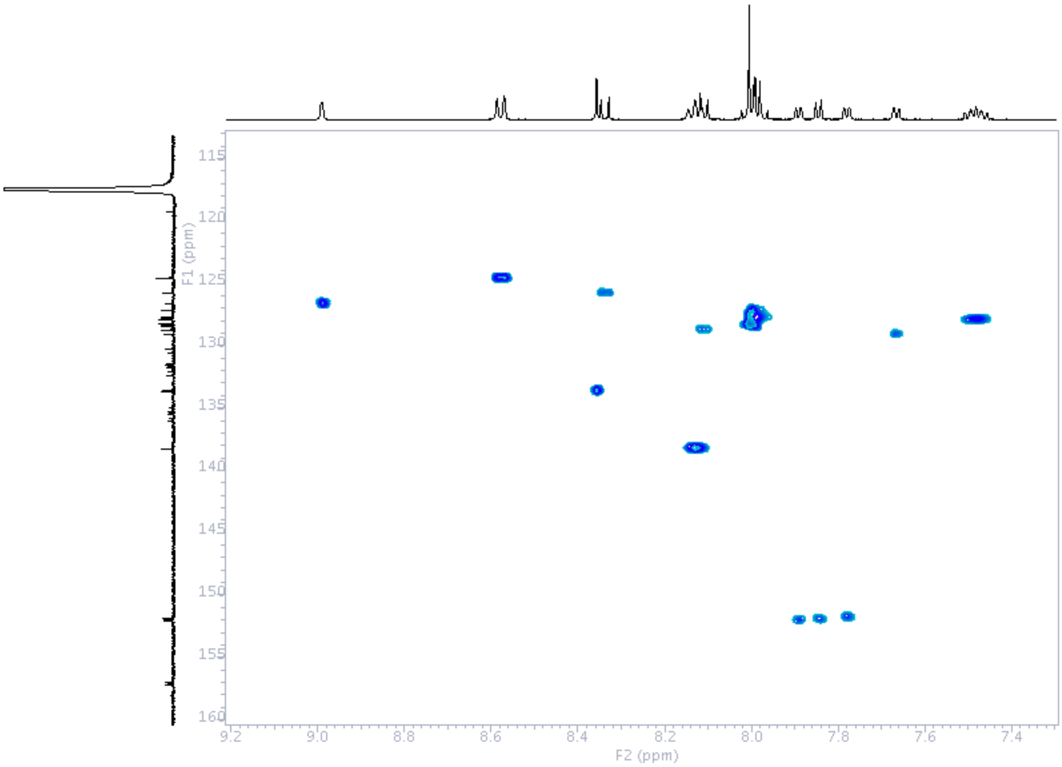

Figure S 56. ^{1}H-^{13}C gHSQCAD (298 K, 500 MHz, Acetonitrile-d_3) spectrum of compound **Ru1C·PF$_6$**.

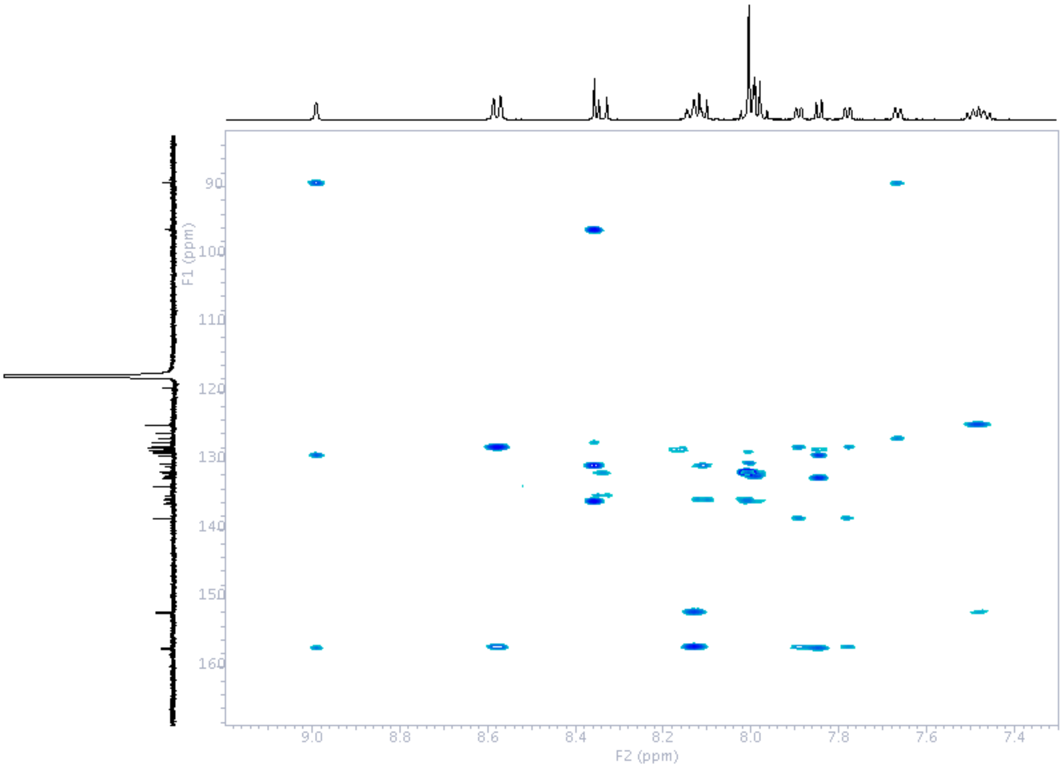

Figure S 57. ^{1}H-^{13}C gHMBCAD (298 K, 500 MHz, Acetonitrile-d_3) spectrum of compound **Ru1C·PF$_6$**.

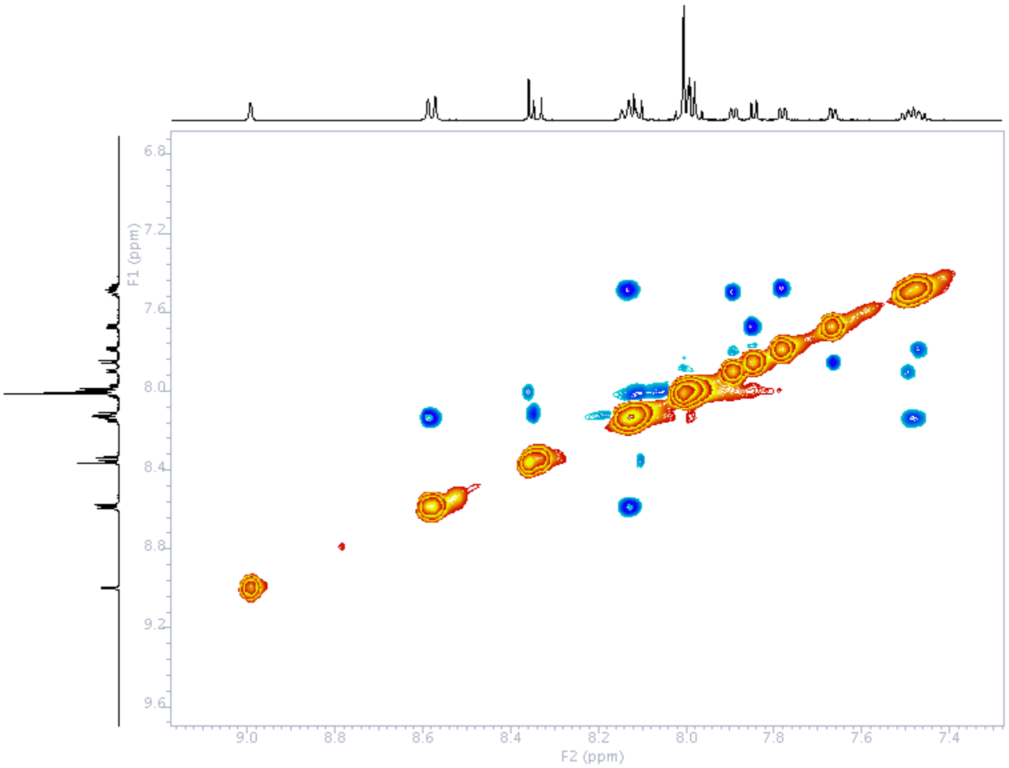

Figure S 58. ^{1}H-^{1}H ROESYAD (298 K, 500 MHz, Acetonitrile-d_3) spectrum of compound **Ru1C·PF$_6$**.

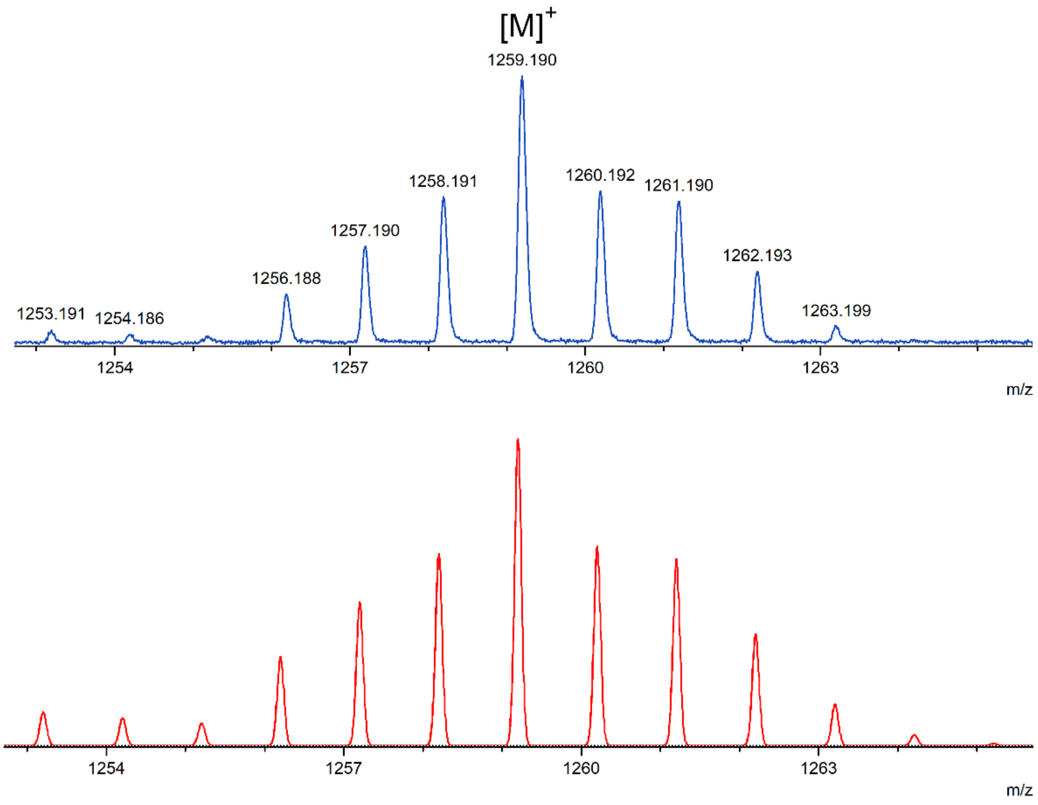

Figure S 59. HRMS (MALDI) of **Ru1C·PF$_6$**, [Ru1C + PF_6]$^+$. Calculated (red), measured (blue).

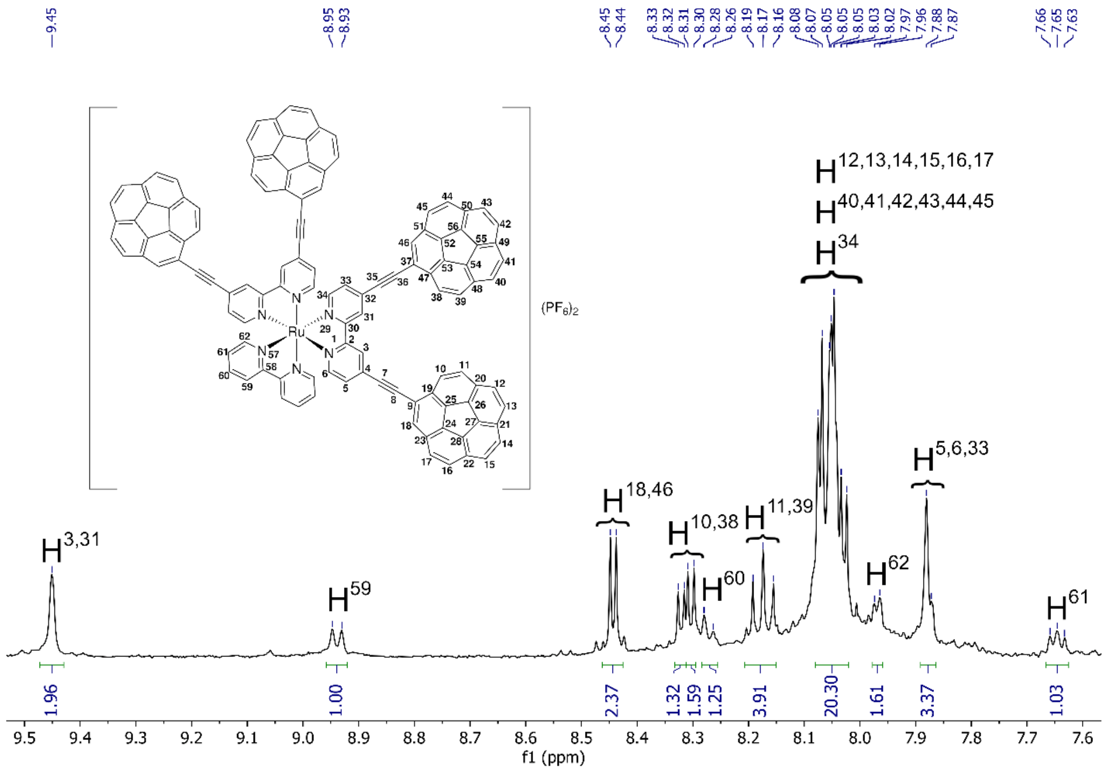

Figure S 60. ^{1}H-NMR (298 K, 500 MHz, DMSO-d_6) spectrum of **Ru2C·PF$_6$**.

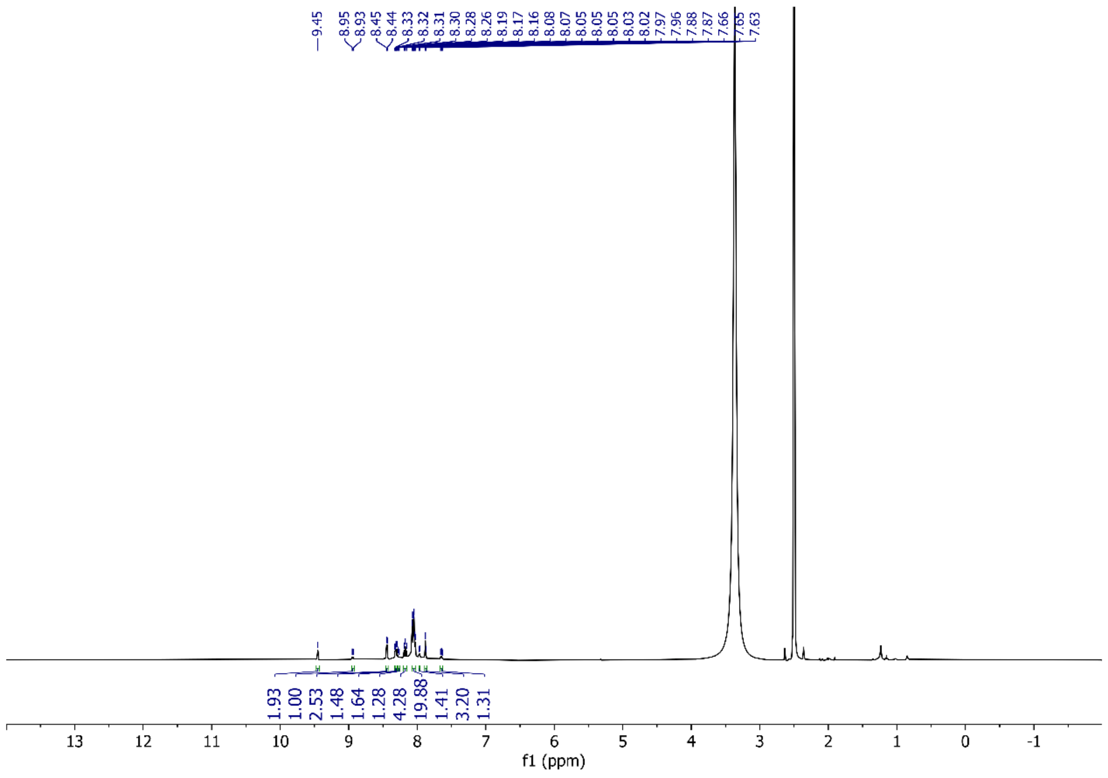

Figure S 61. Full ^{1}H-NMR (298 K, 500 MHz, DMSO-d_6) spectrum of **Ru2C·PF$_6$**.

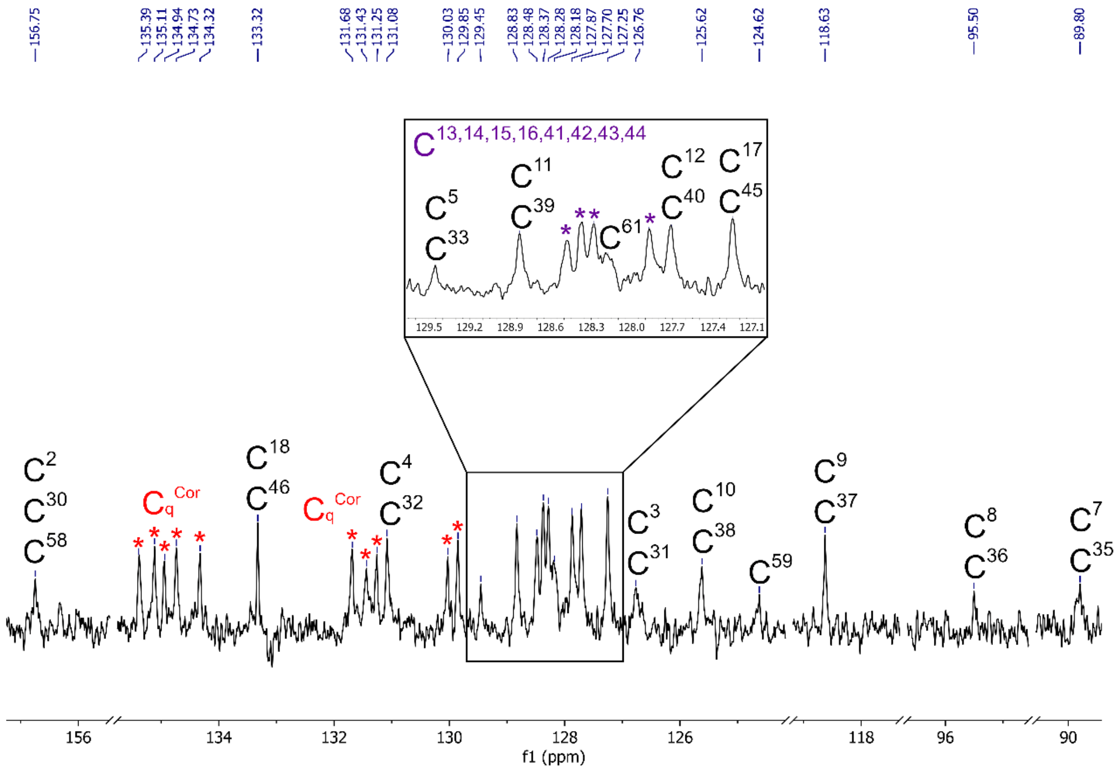

Figure S 62. $^{13}C\{^{1}H\}$-NMR (298 K, 126 MHz, DMSO-d_6) spectrum of **Ru2C·PF$_6$.**

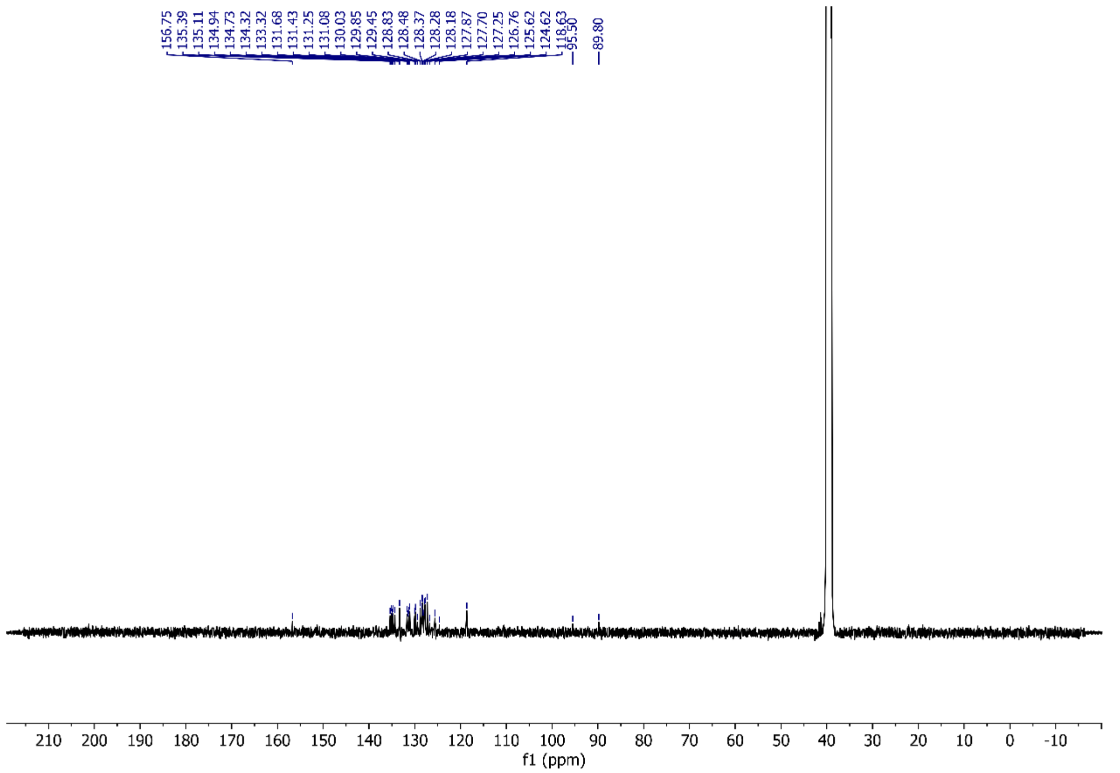

Figure S 63. Full $^{13}C\{^{1}H\}$-NMR (298 K, 126 MHz, DMSO-d_6) spectrum of **Ru2C·PF$_6$.**

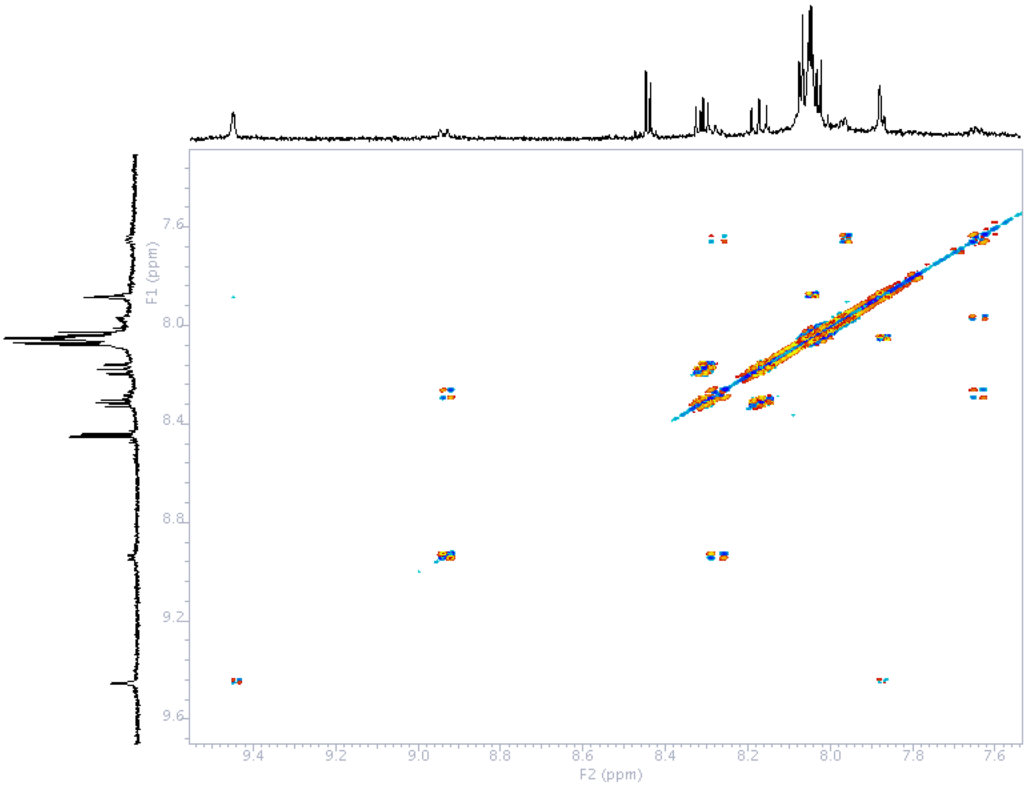

Figure S 64. ^1H-^1H gDQCOSY (298 K, 500 MHz, DMSO-d_6) spectrum of compound **Ru2C·PF$_6$**.

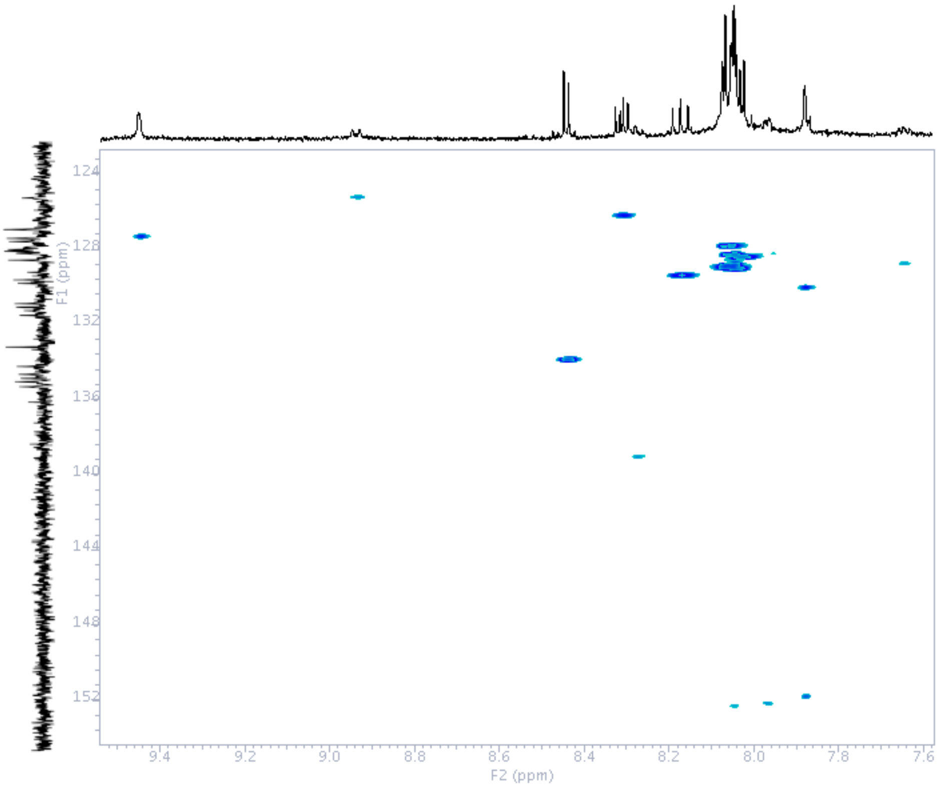

Figure S 65. ^1H-^{13}C c2HSQCse (298 K, 500 MHz, DMSO-d_6) spectrum of compound **Ru2C·PF$_6$**.

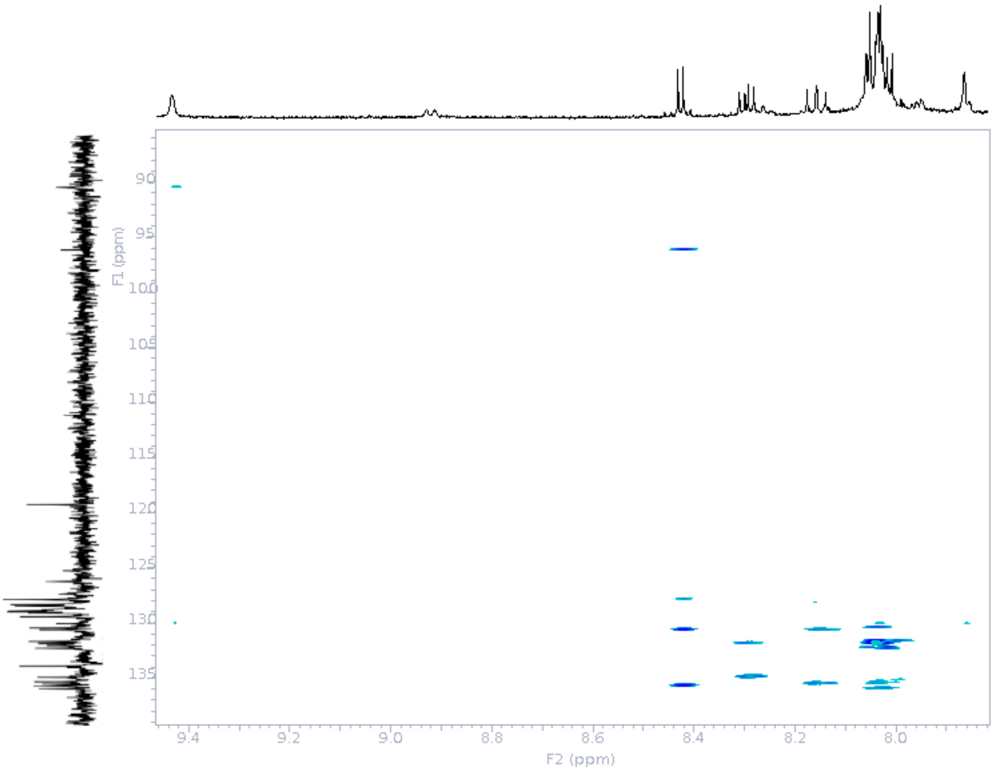

Figure S 66. ^{1}H-^{13}C gc2HMBC (298 K, 500 MHz, DMSO-d_6) spectrum of compound **Ru2C·PF₆**.

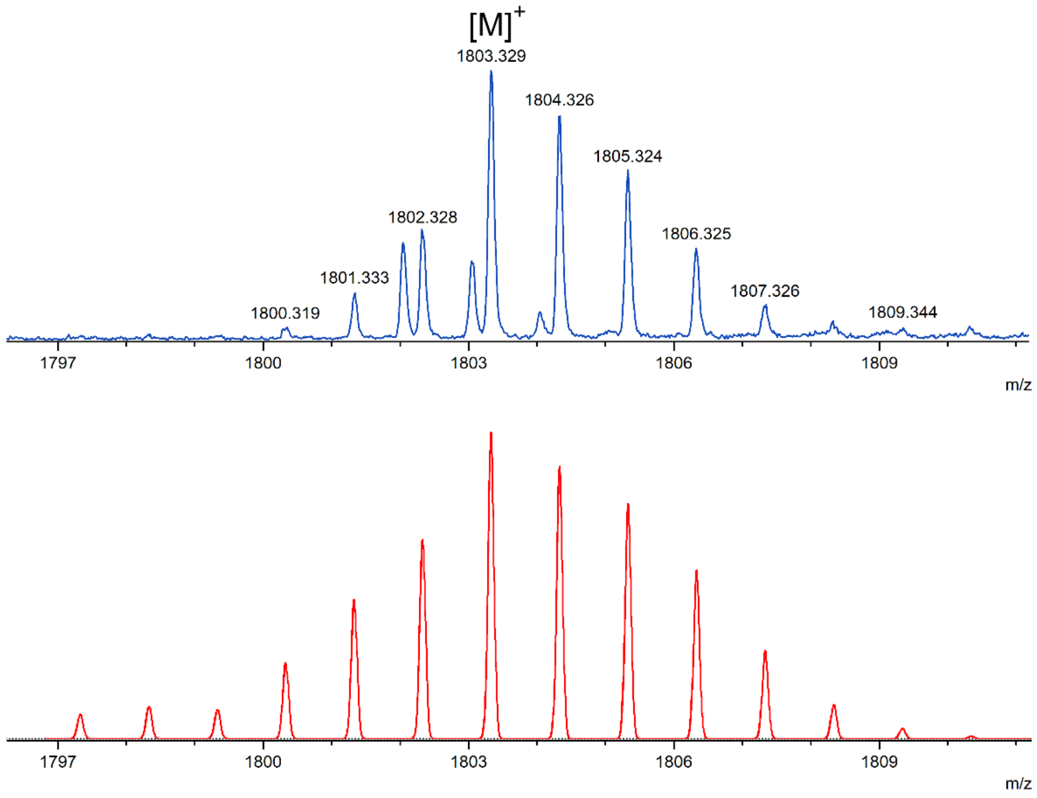

Figure S 67. HRMS (MALDI) of **Ru2C·PF₆**, [Ru2C + PF_6]$^+$**.** Calculated (red), measured (blue).

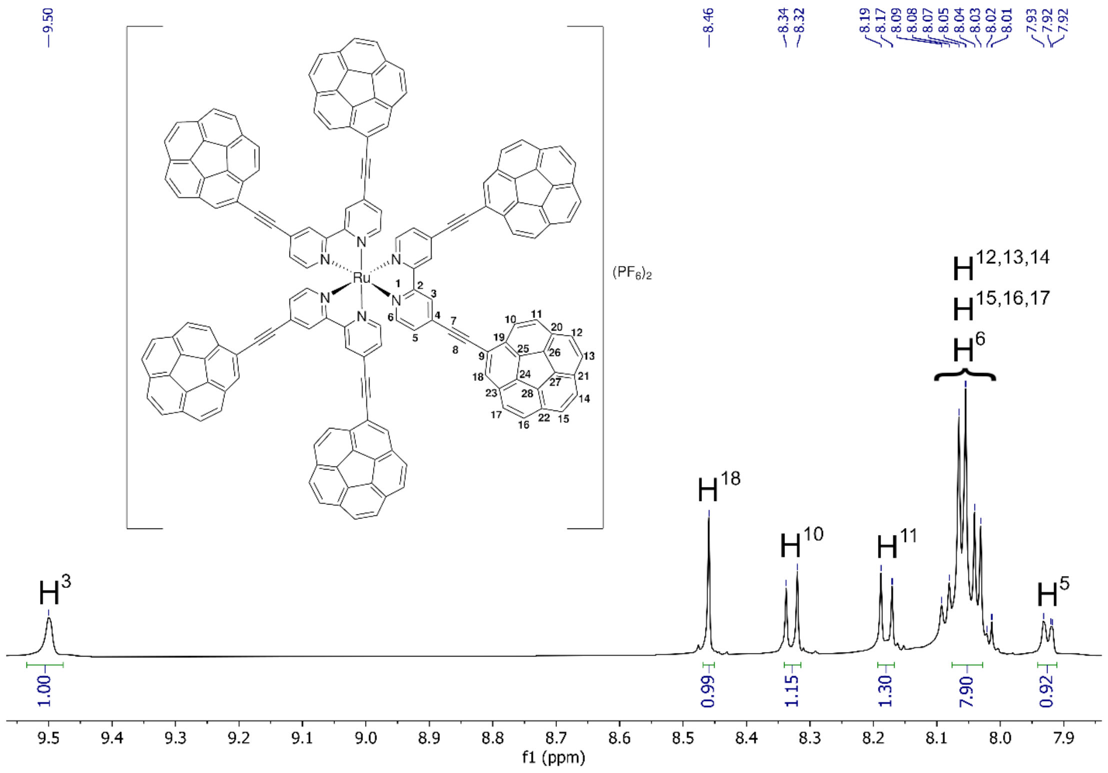

Figure S 68. ^{1}H-NMR (298 K, 500 MHz, DMSO-d_6) spectrum of **Ru3C·PF6**.

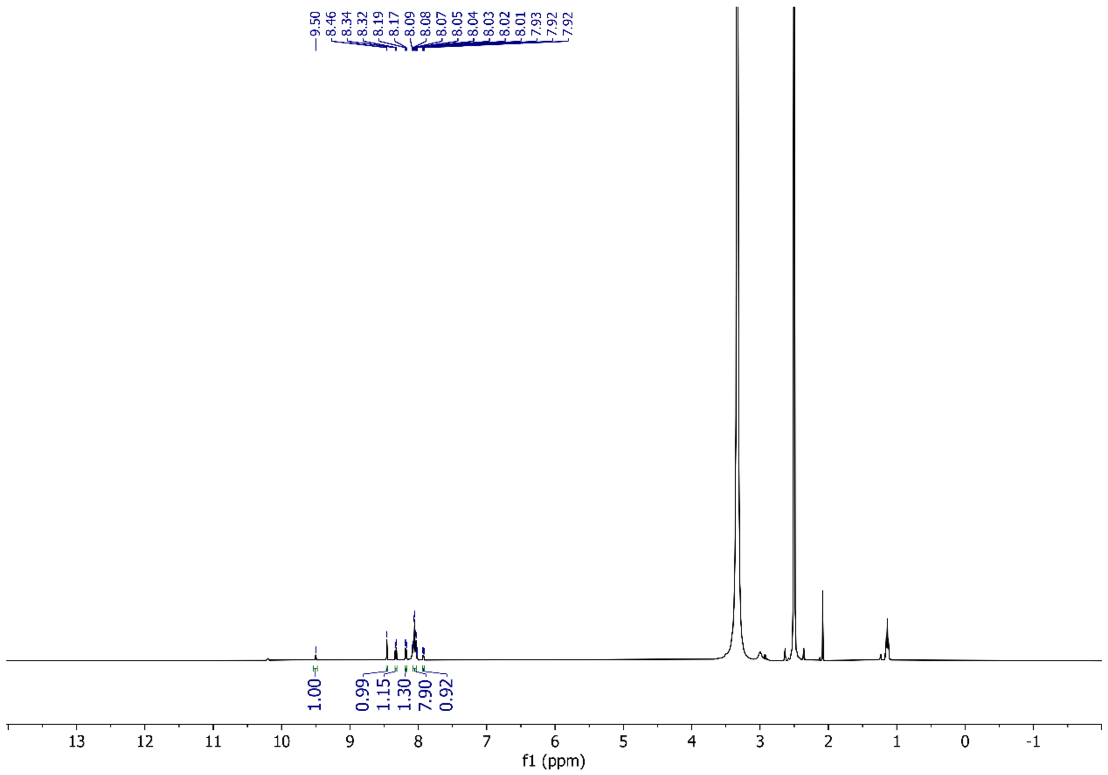

Figure S 69. Full ^{1}H-NMR (298 K, 500 MHz, DMSO-d_6) spectrum of **Ru3C·PF6**.

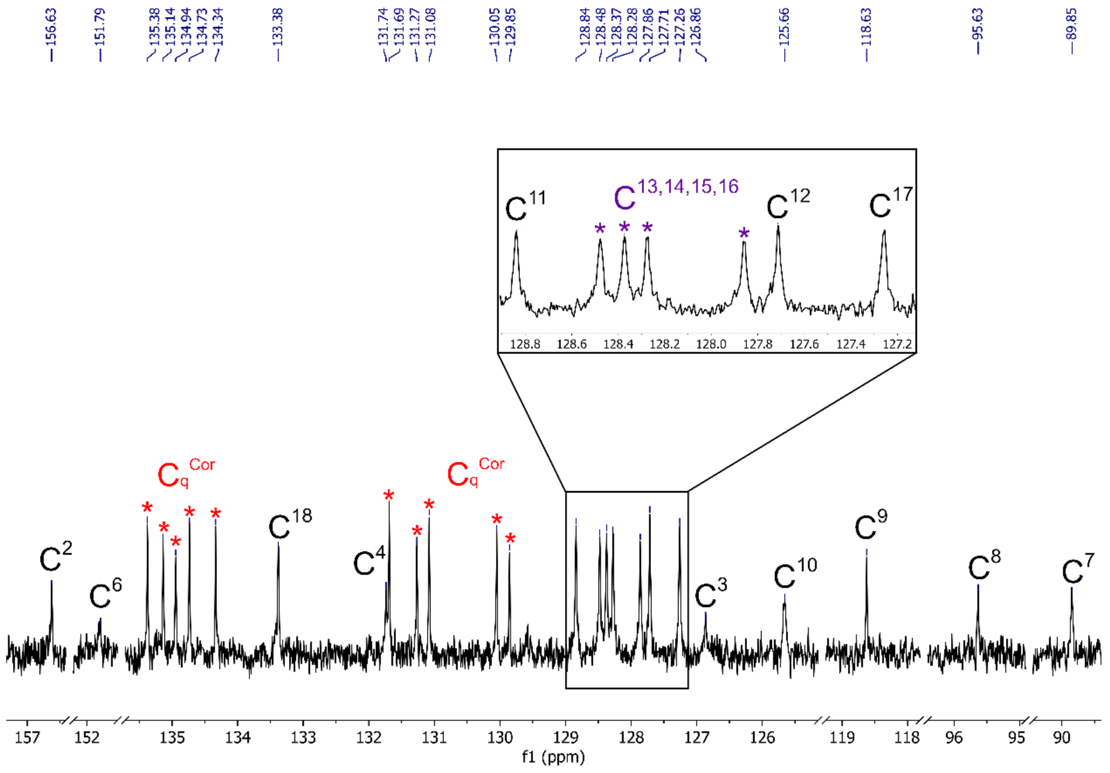

Figure S 70. $^{13}C\{^{1}H\}$-NMR (298 K, 126 MHz, DMSO-d_6) spectrum of **Ru3C·PF₆.**

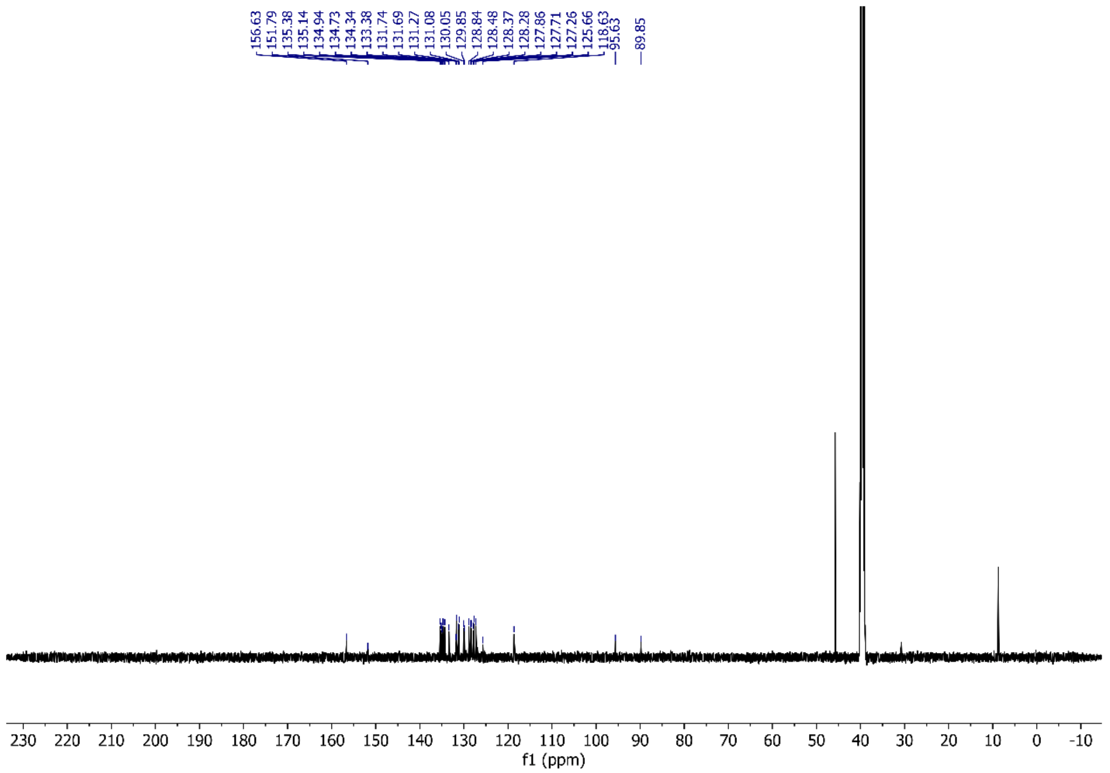

Figure S 71. Full $^{13}C\{^{1}H\}$-NMR (298 K, 126 MHz, DMSO-d_6) spectrum of **Ru3C·PF₆.**

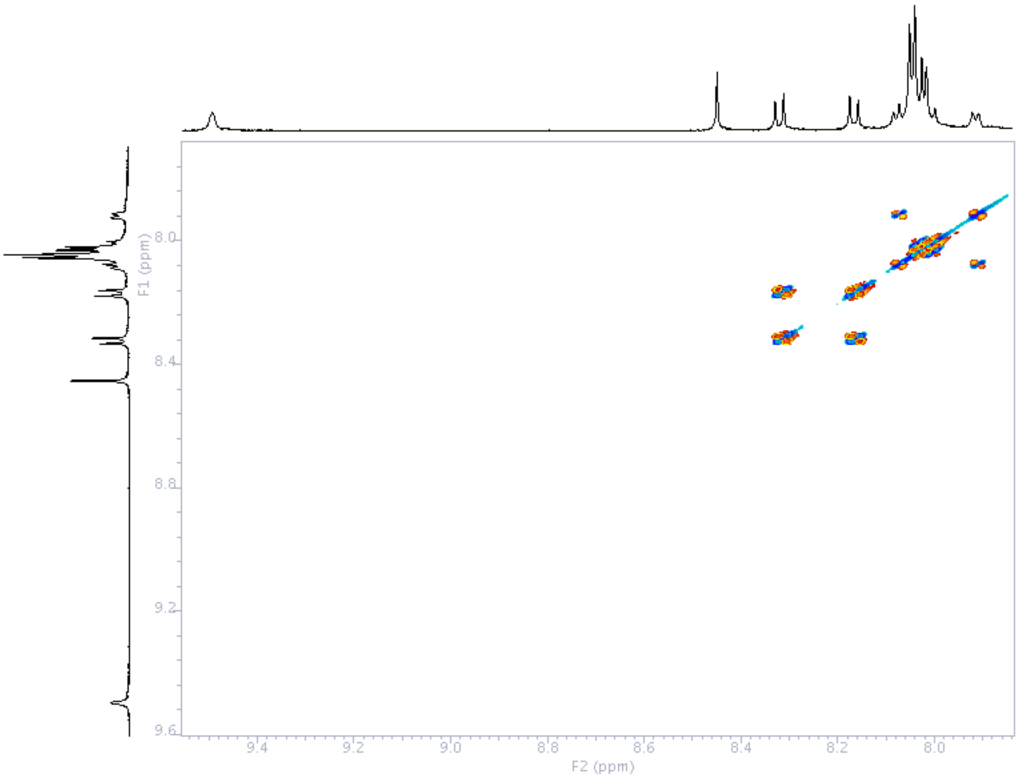

Figure S 72. ^{1}H-^{1}H gDQCOSY (298 K, 500 MHz, DMSO-d_6) spectrum of compound **Ru3C·PF$_6$**.

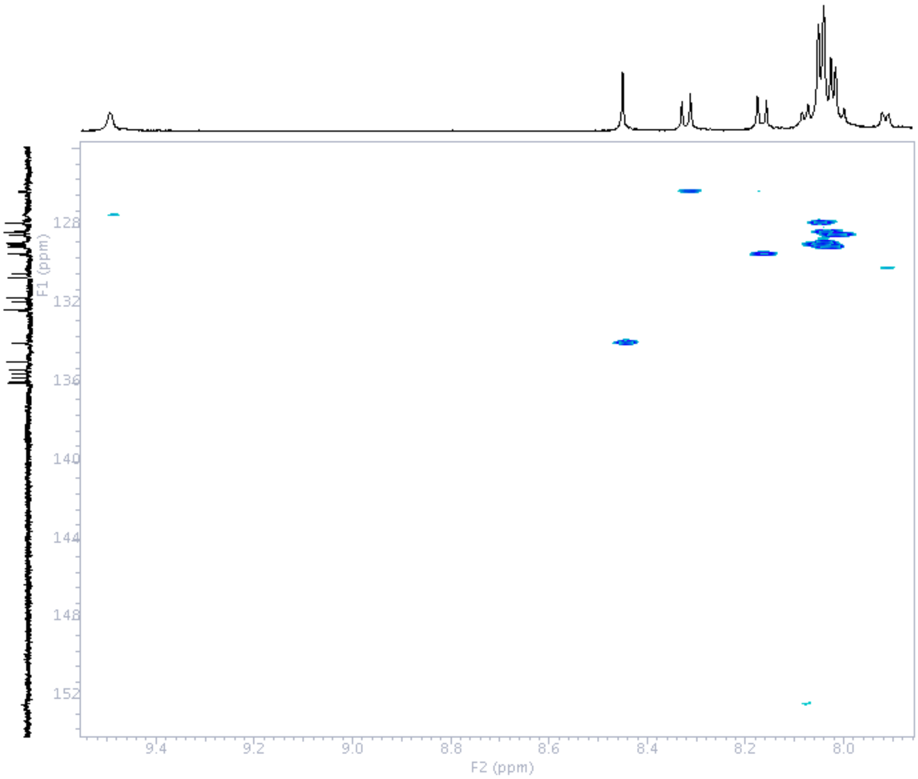

Figure S 73. ^{1}H-^{13}C gc2HSQC (298 K, 500 MHz, DMSO-d_6) spectrum of compound **Ru3C·PF$_6$**.

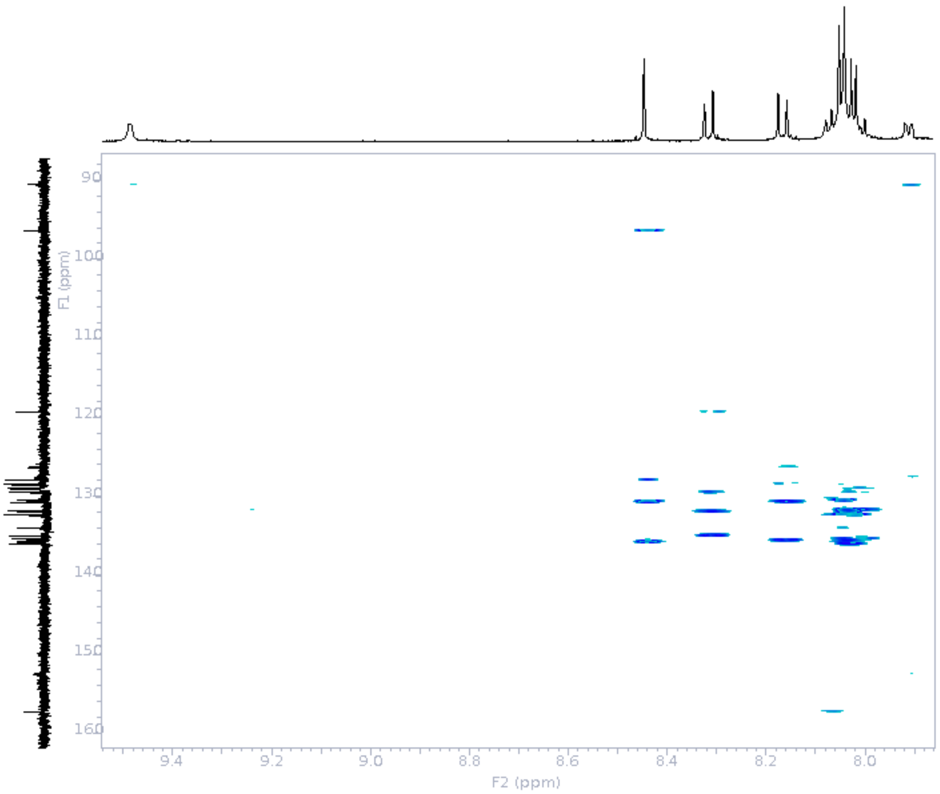

Figure S 74. ^{1}H-^{13}C gc2HMBC (298 K, 500 MHz, DMSO-d_6) spectrum of compound **Ru3C·PF6**.

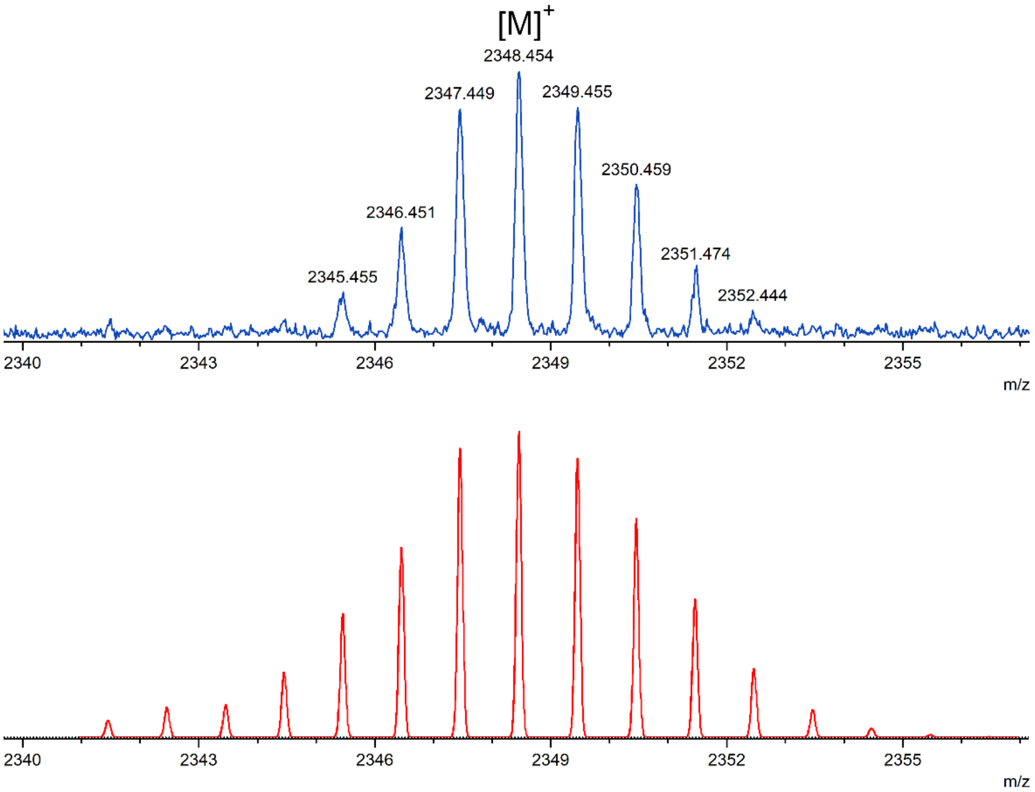

Figure S 75. HRMS (MALDI) of **Ru3C·PF6**, $[Ru3C + PF_6]^{+}$**.** Calculated (red), measured (blue).

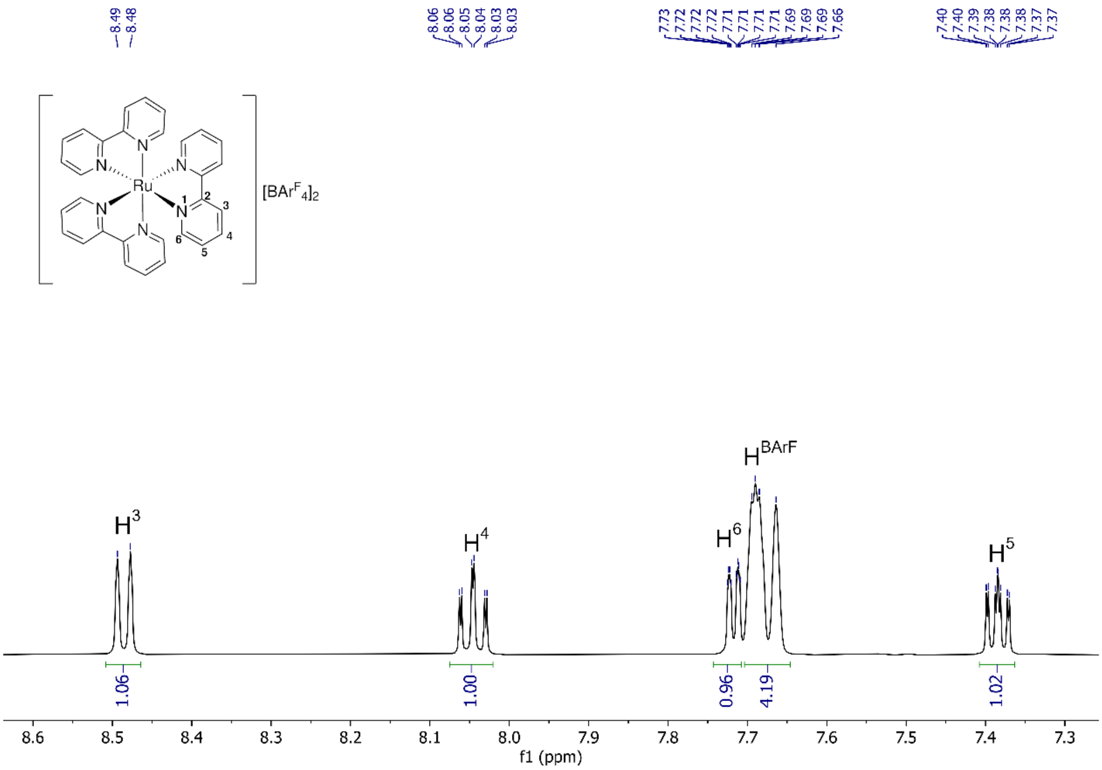

Figure S 76. ^{1}H-NMR (298 K, 500 MHz, Acetonitrile-d_3) spectrum of **Ru(bpy)$_3$·BAr^F$_4$**.

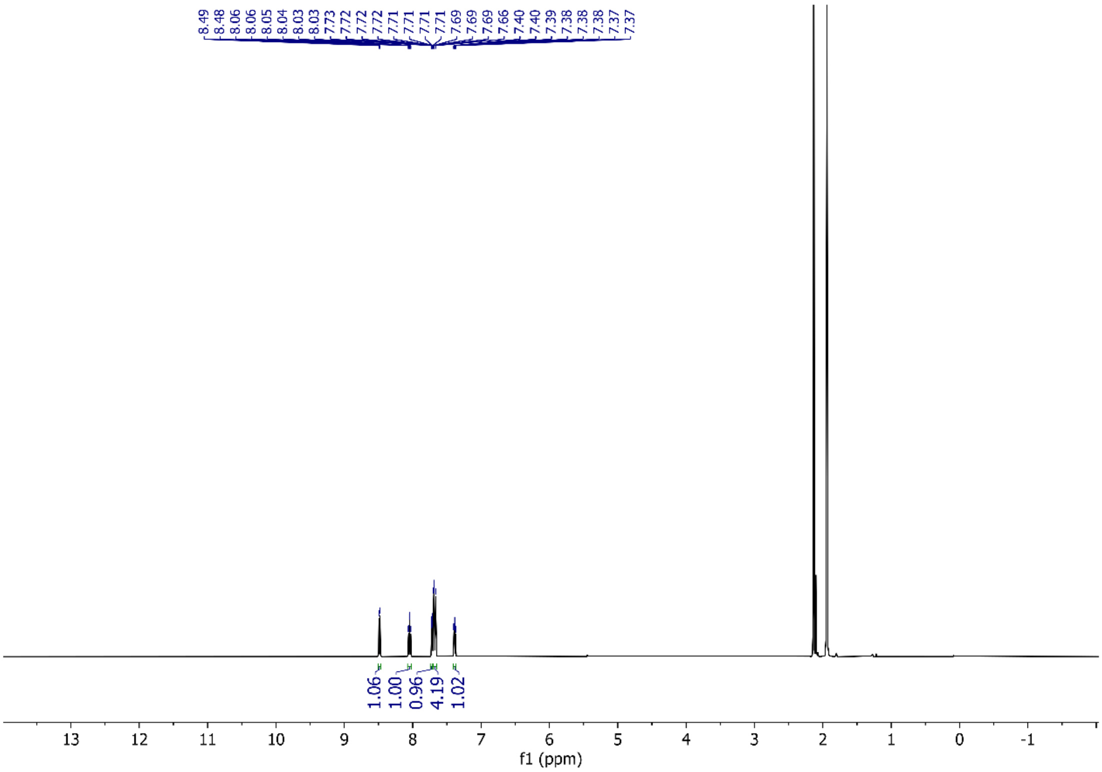

Figure S 77. Full ^{1}H-NMR (298 K, 500 MHz, Acetonitrile-d_3) spectrum of **Ru(bpy)$_3$·BAr^F$_4$**.

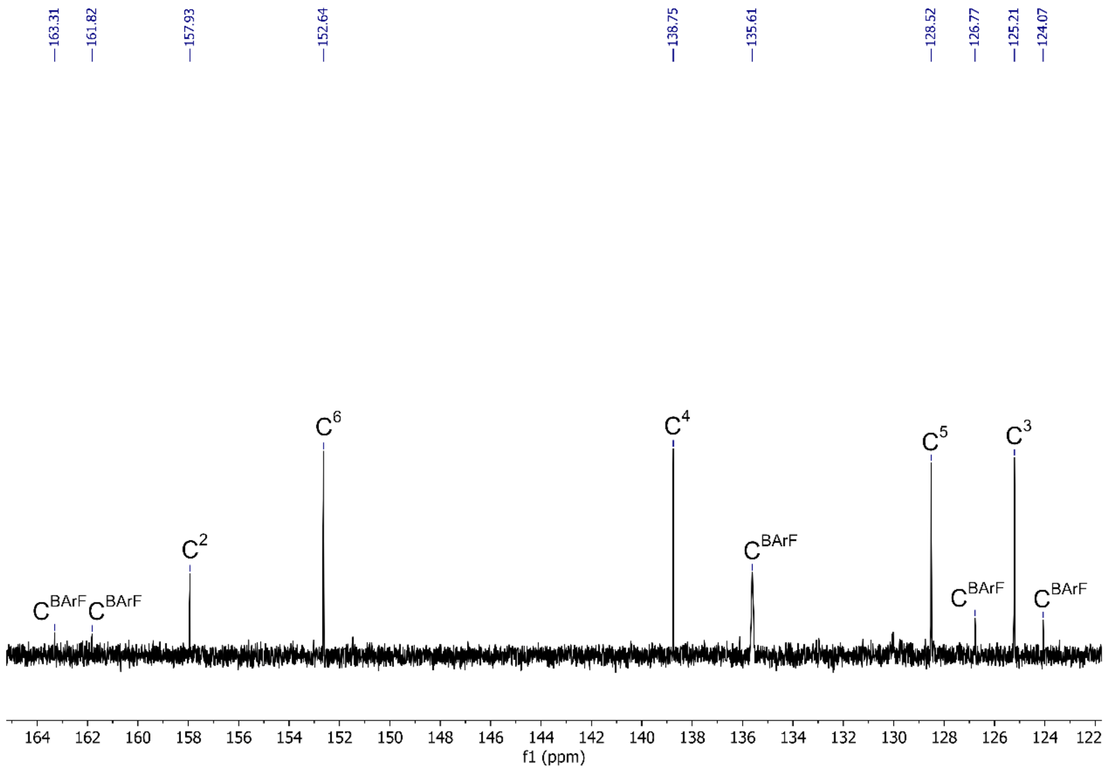

Figure S 78. $^{13}C\{^{1}H\}$ NMR (298 K, 101 MHz, Acetonitrile-d_3) spectrum of **Ru(bpy)$_3$·BAr^F$_4$**.

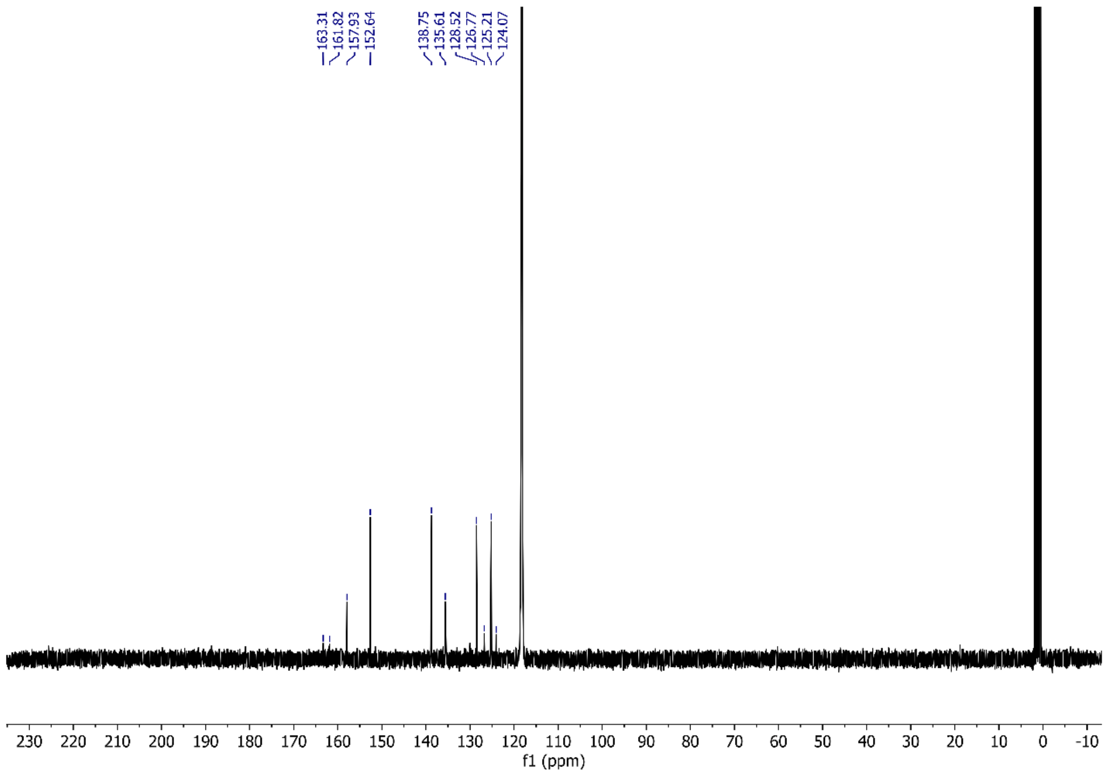

Figure S 79. Full $^{13}C\{^{1}H\}$ NMR (298 K, 101 MHz, Acetonitrile-d_3) spectrum of **Ru(bpy)$_3$·BAr^F$_4$**.

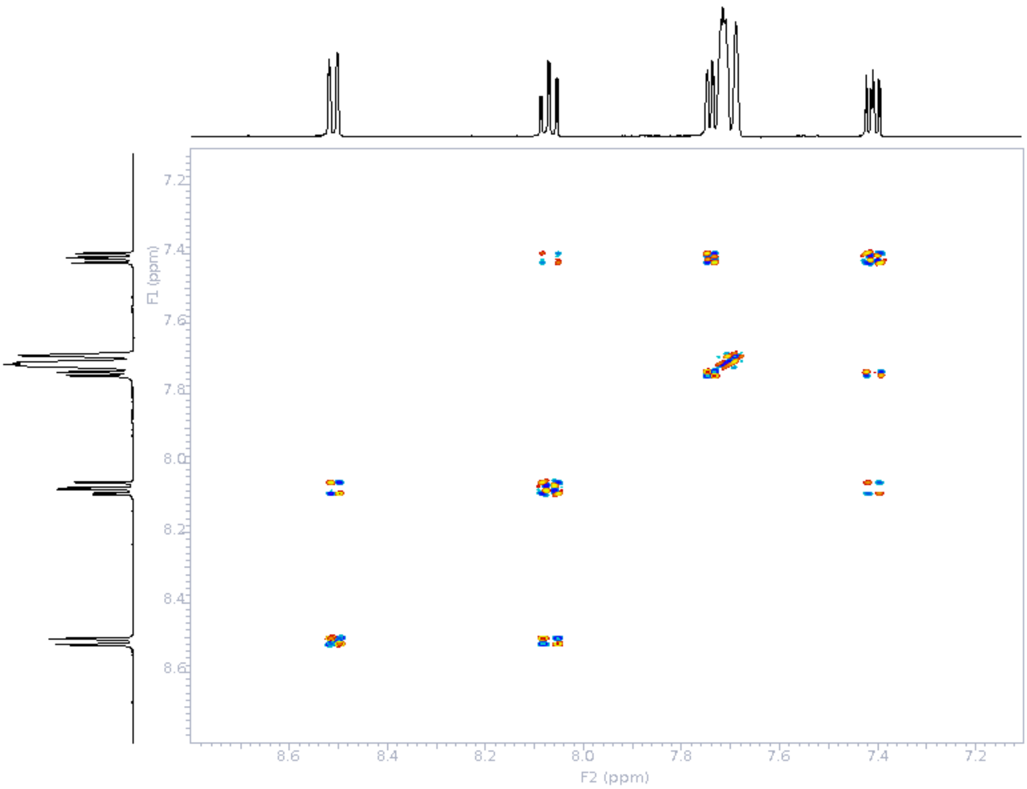

Figure S 80. ^{1}H-^{1}H gDQCOSY (298 K, 500 MHz, Acetonitrile-*d*$_3$) spectrum of compound **Ru(bpy)$_3$·BAr$^F{}_4$**.

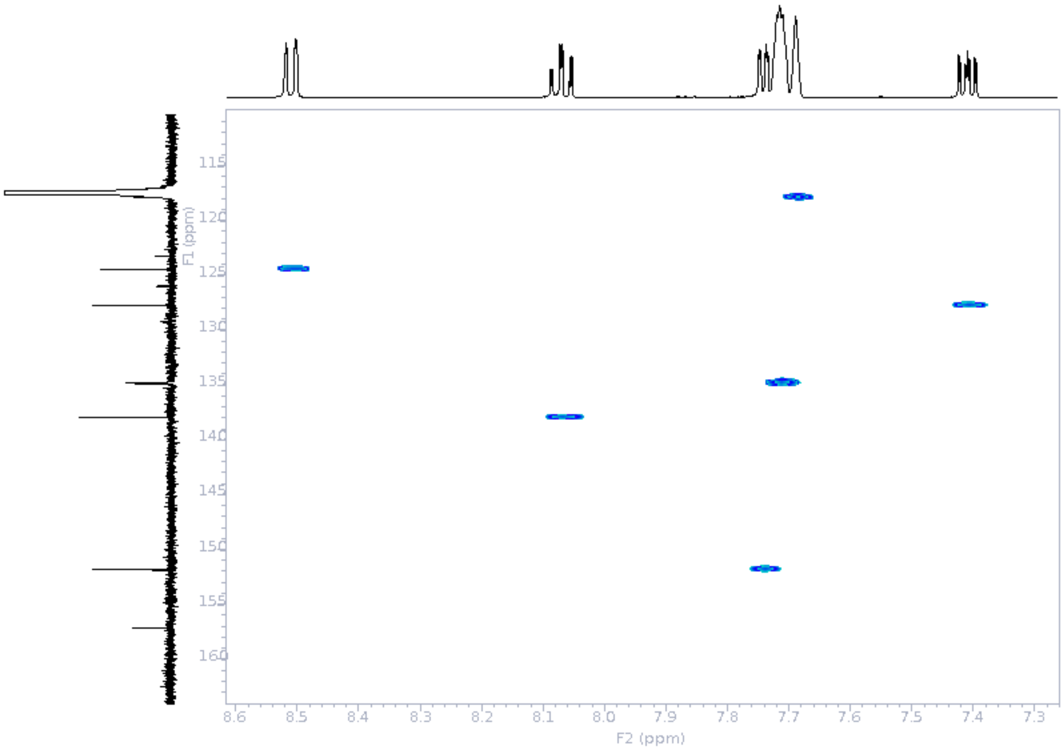

Figure S 81. ^{1}H-^{13}C gc2HSQC (298 K, 500 MHz, Acetonitrile-*d*$_3$) spectrum of compound **Ru(bpy)$_3$·BAr$^F{}_4$**.

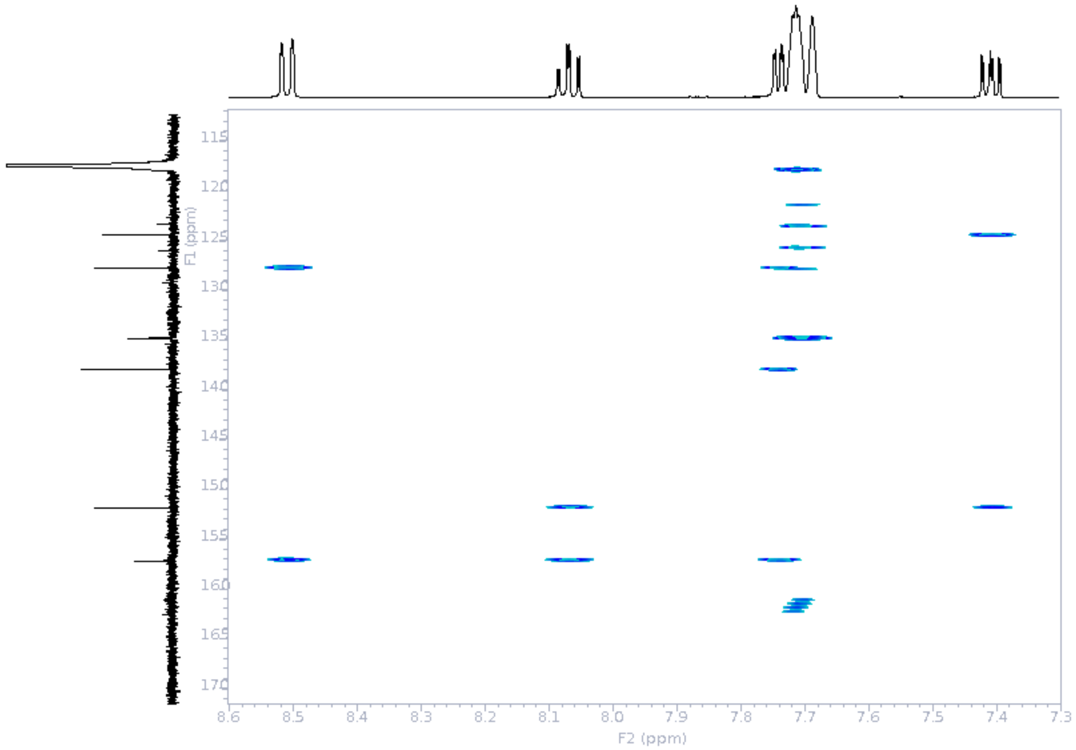

Figure S 82. ^{1}H-^{13}C gc2HMBC (298 K, 500 MHz, Acetonitrile-*d*$_3$) spectrum of compound **Ru(bpy)$_3$·BAr^F$_4$**.

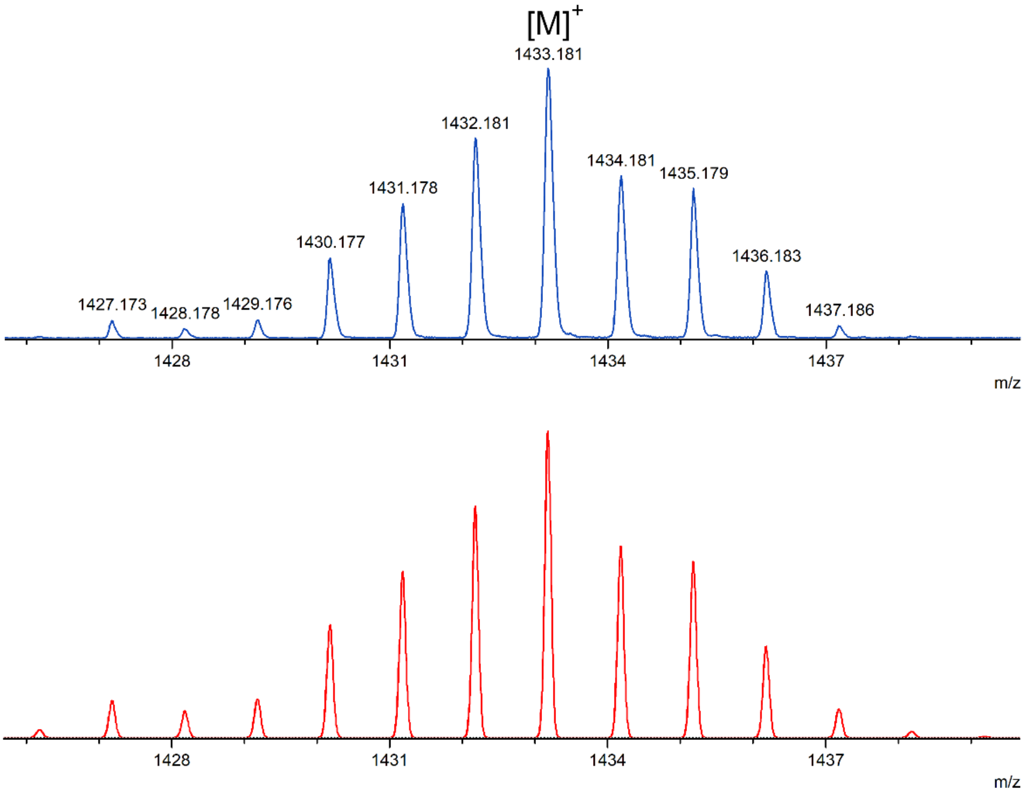

Figure S 83. HRMS (MALDI) of **Ru(bpy)$_3$·BAr^F$_4$**, [Ru(bpy)$_3$ + BAr^F$_4$]$^+$. Calculated (red), measured (blue).

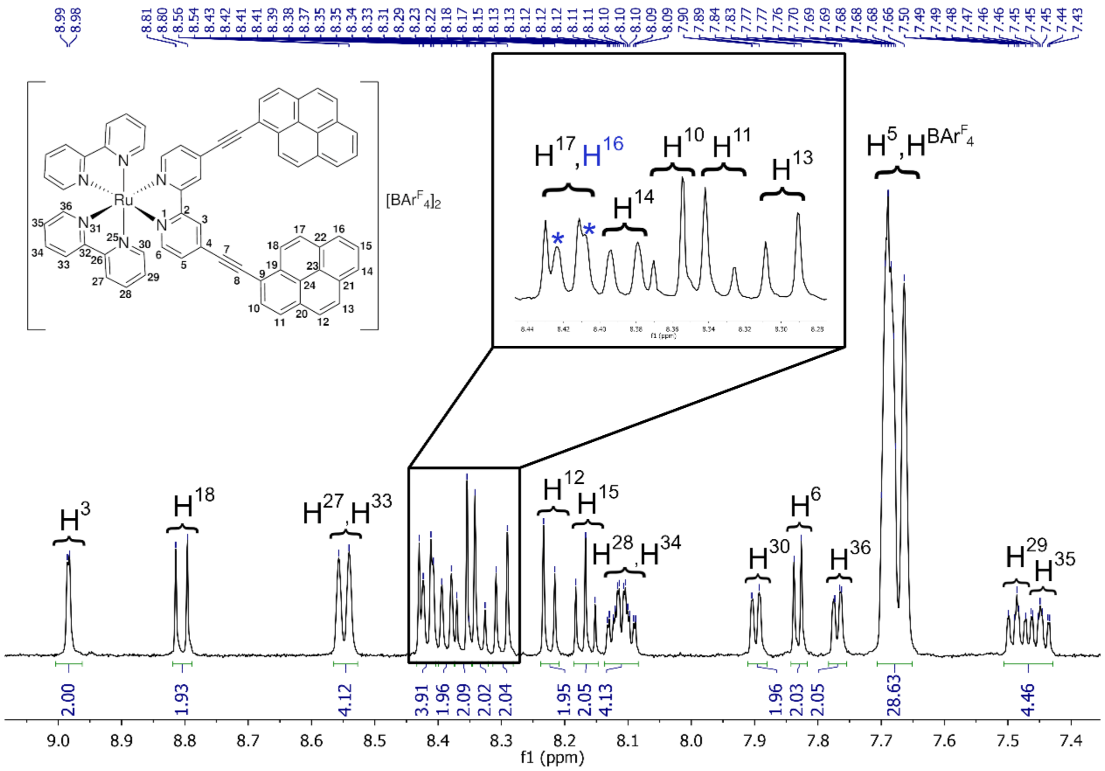

Figure S 84. ^{1}H-NMR (298 K, 500 MHz, Acetonitrile-d_3) spectrum of **Ru1P·BAr^F$_4$.**

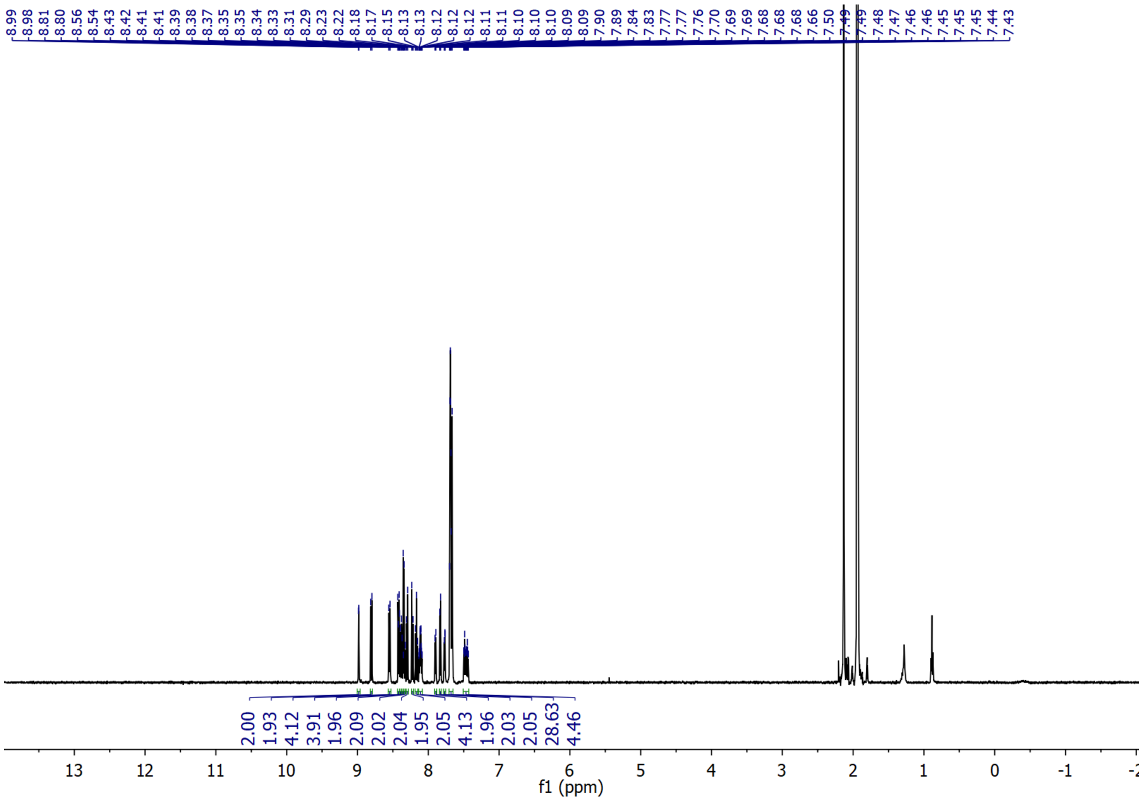

Figure S 85. Full ^{1}H-NMR (298 K, 500 MHz, Acetonitrile-d_3) spectrum of **Ru1P·BAr^F$_4$**.

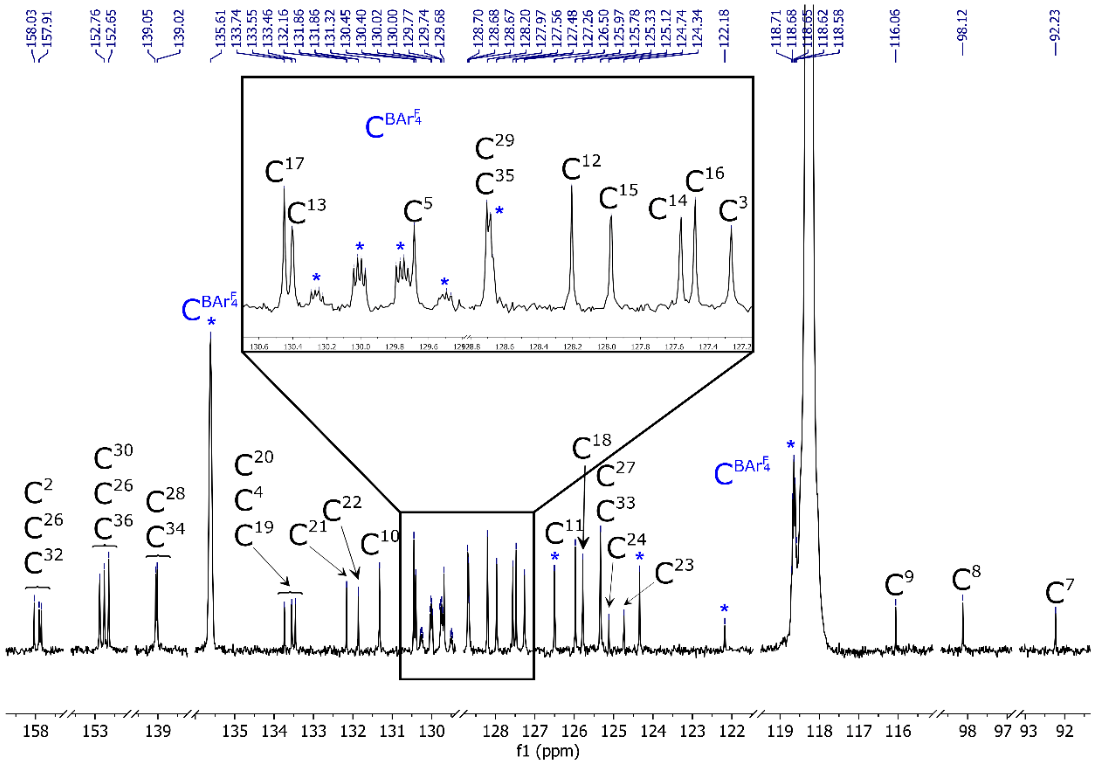

Figure S 86. $^{13}C\{^1H\}$ NMR (298 K, 126 MHz, Acetonitrile-d_3) spectrum of **Ru1P·BArF4**.

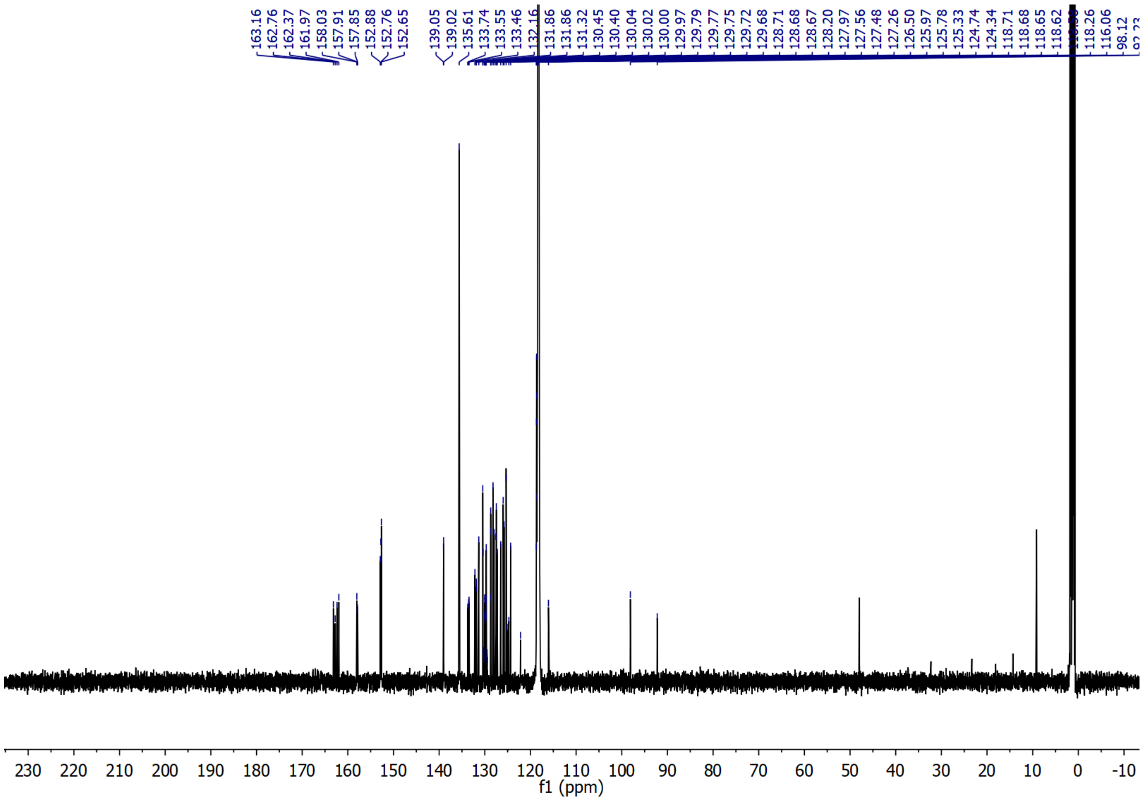

Figure S 87. Full $^{13}C\{^1H\}$-NMR (298 K, 126 MHz, Acetonitrile-d_3) spectrum of **Ru1P·BArF4**.

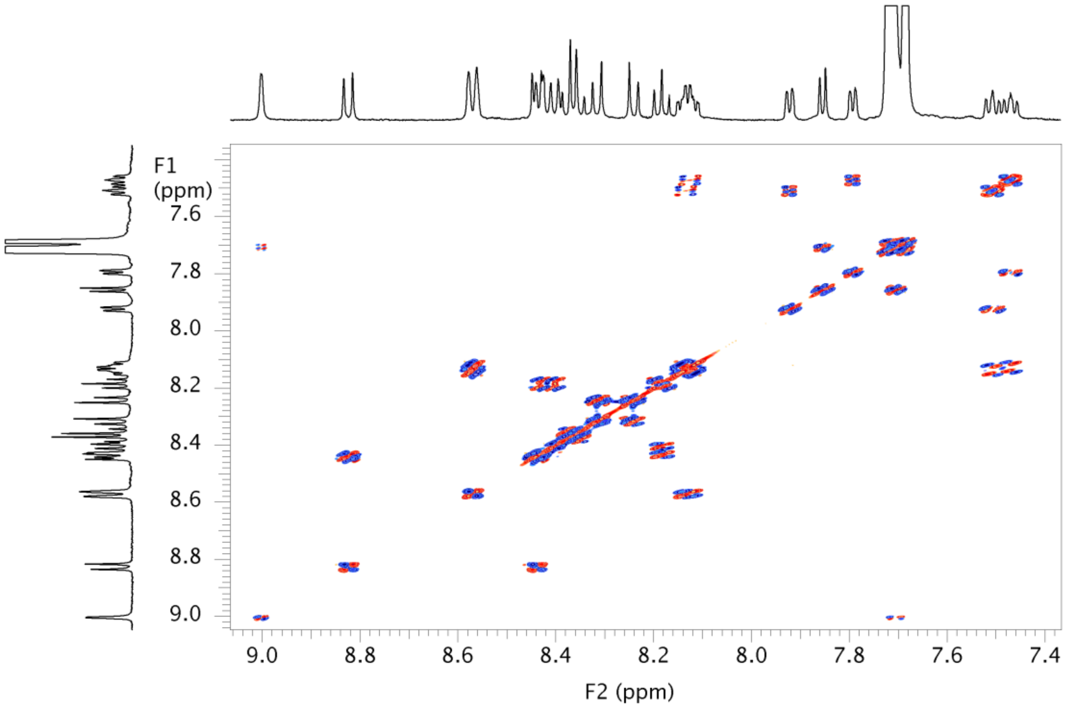

Figure S 88. ^{1}H-^{1}H gDQCOSY (298 K, 500 MHz, Acetonitrile-d_3) spectrum of compound **Ru1P·BAr^{F}$_4$**.

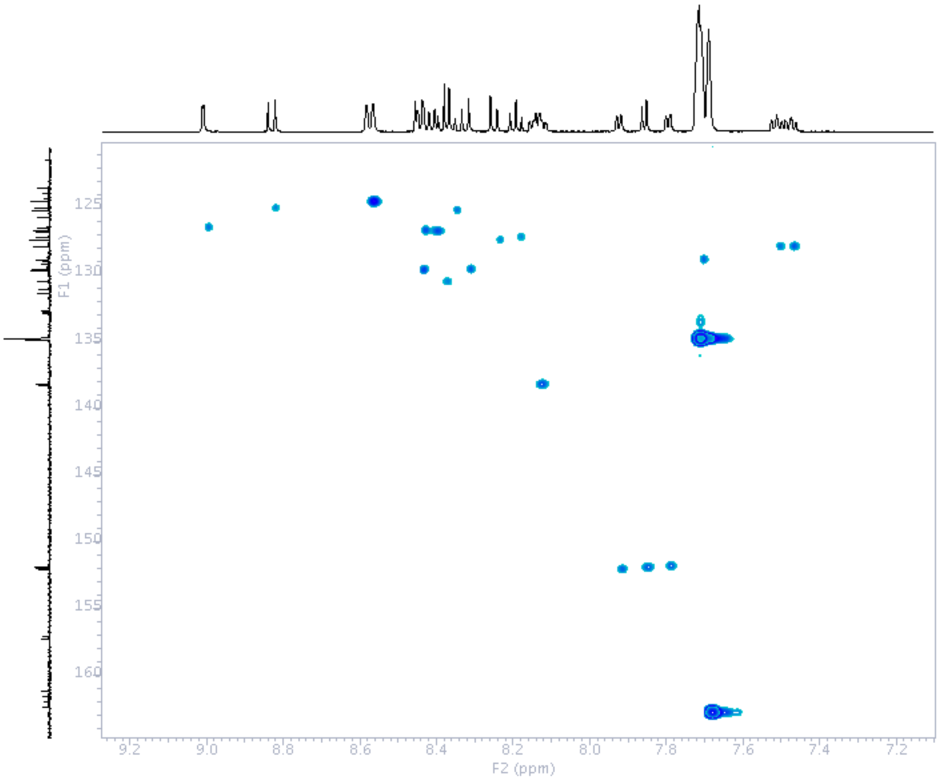

Figure S 89. ^{1}H-^{13}C gHSQCAD-PS (298 K, 500 MHz, Acetonitrile-d_3) spectrum of compound **Ru1P·BAr^{F}$_4$**.

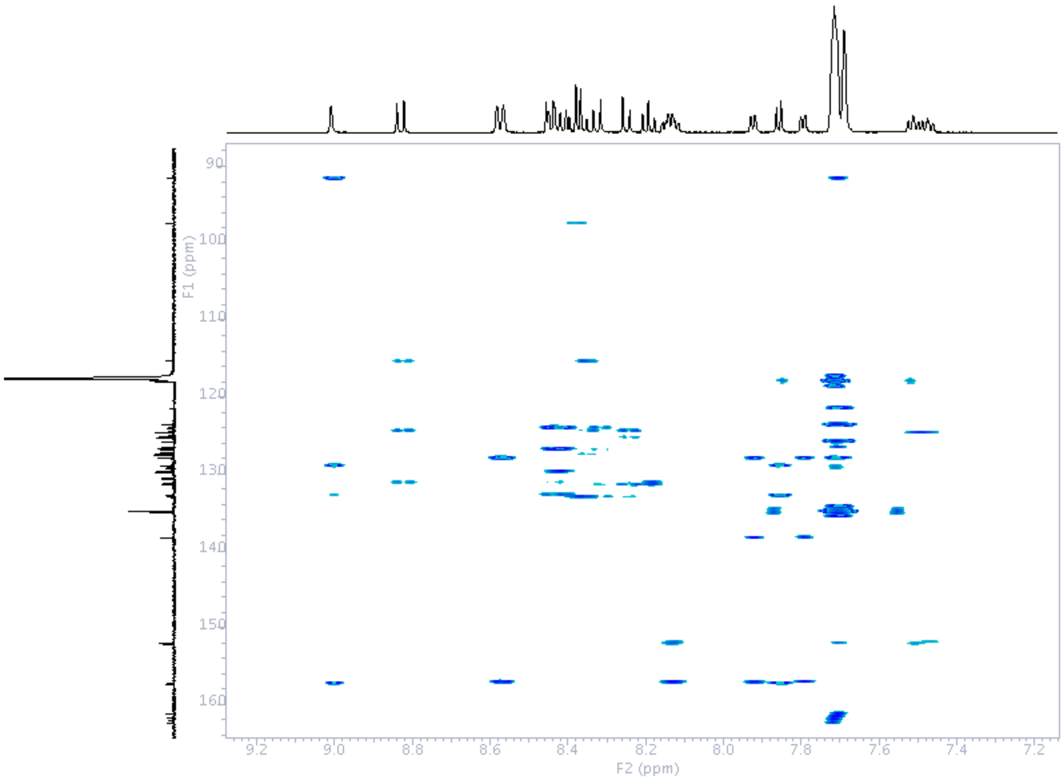

Figure S 90. ^{1}H-^{13}C bsgHMBC (298 K, 500 MHz, Acetonitrile-d_3) spectrum of compound **Ru1P·BAr^F$_4$**.

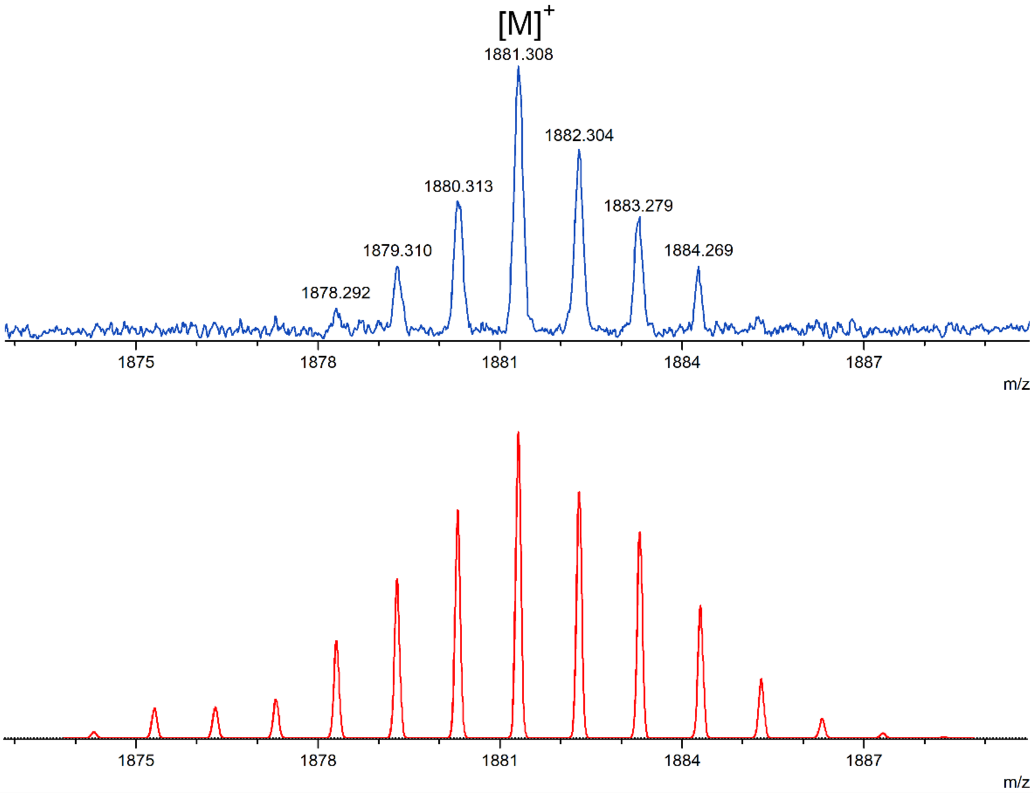

Figure S 91. HRMS (MALDI) of **Ru1P·BAr^F$_4$**, [Ru1P + BAr^F$_4$]$^+$**.** Calculated (red), measured (blue).

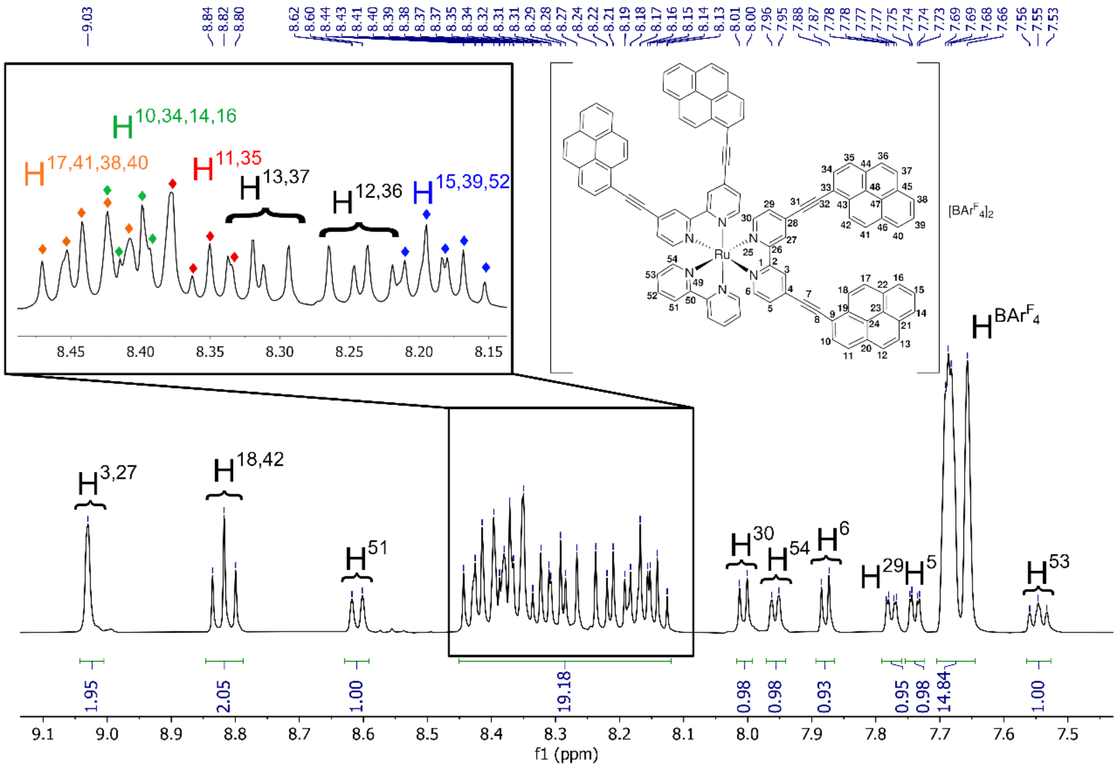

Figure S 92. ^{1}H-NMR (298 K, 500 MHz, Acetonitrile-*d*$_3$) spectrum of **Ru2P·BAr^F$_4$**.

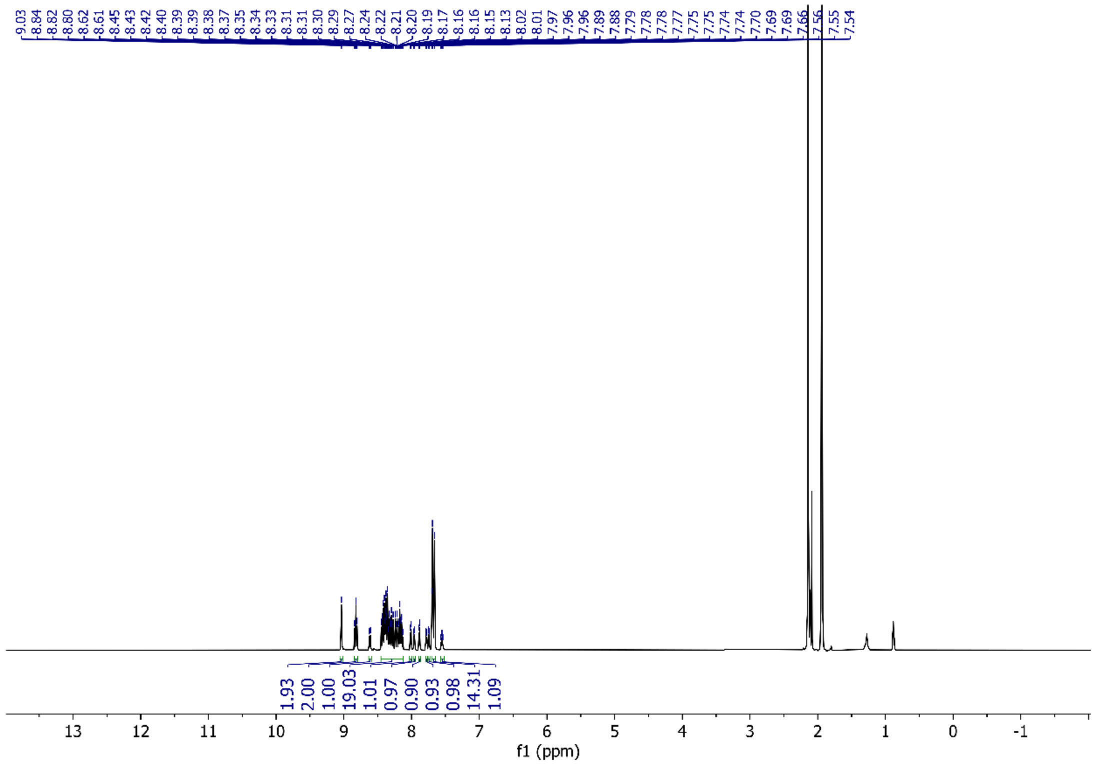

Figure S 93. Full ^{1}H-NMR (298 K, 500 MHz, Acetonitrile-*d*$_3$) spectrum of **Ru2P·BAr^F$_4$**.

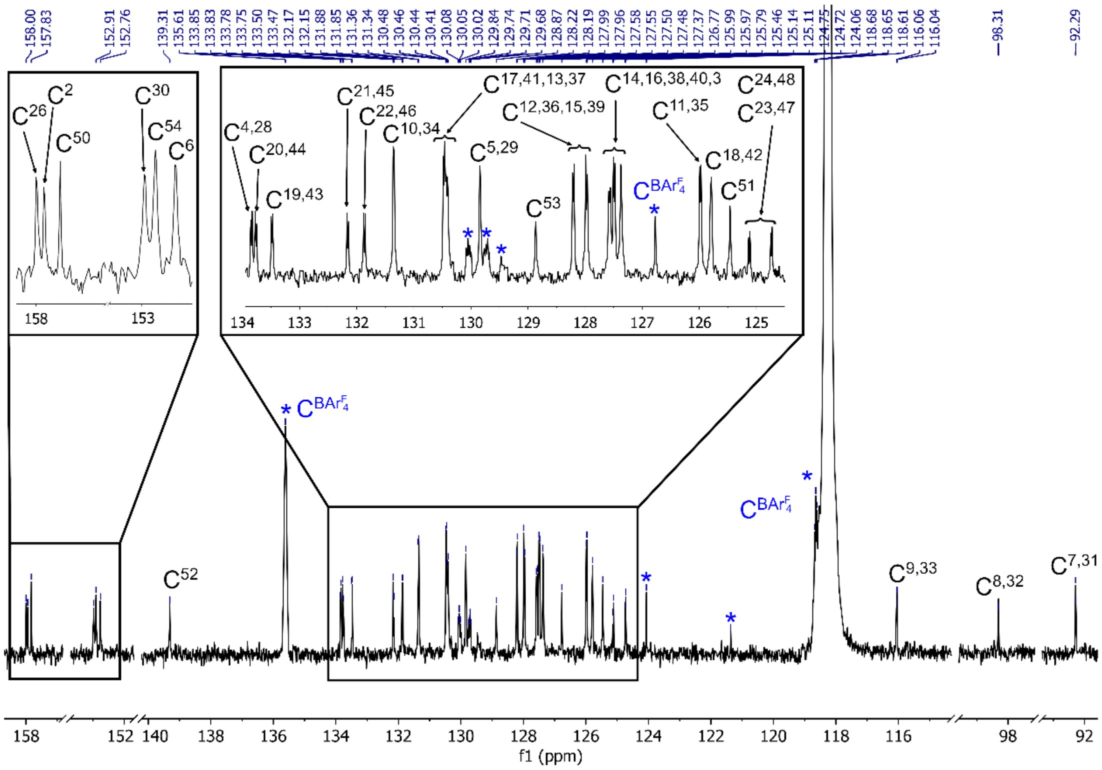

Figure S 94. $^{13}C\{^1H\}$-NMR (298 K, 101 MHz, Acetonitrile-d_3) spectrum of **Ru2P·BAr^F$_4$**.

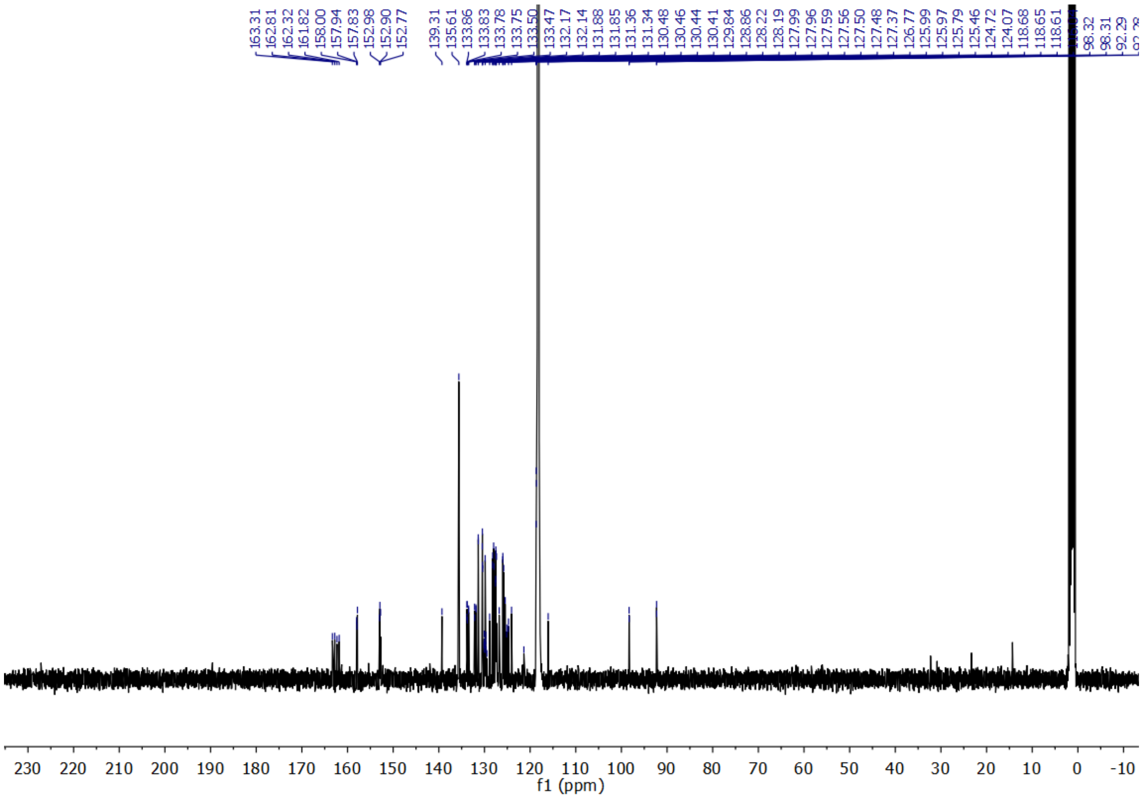

Figure S 95. Full $^{13}C\{^1H\}$-NMR (298 K, 101 MHz, Acetonitrile-d_3) spectrum of **Ru2P·BAr^F$_4$**.

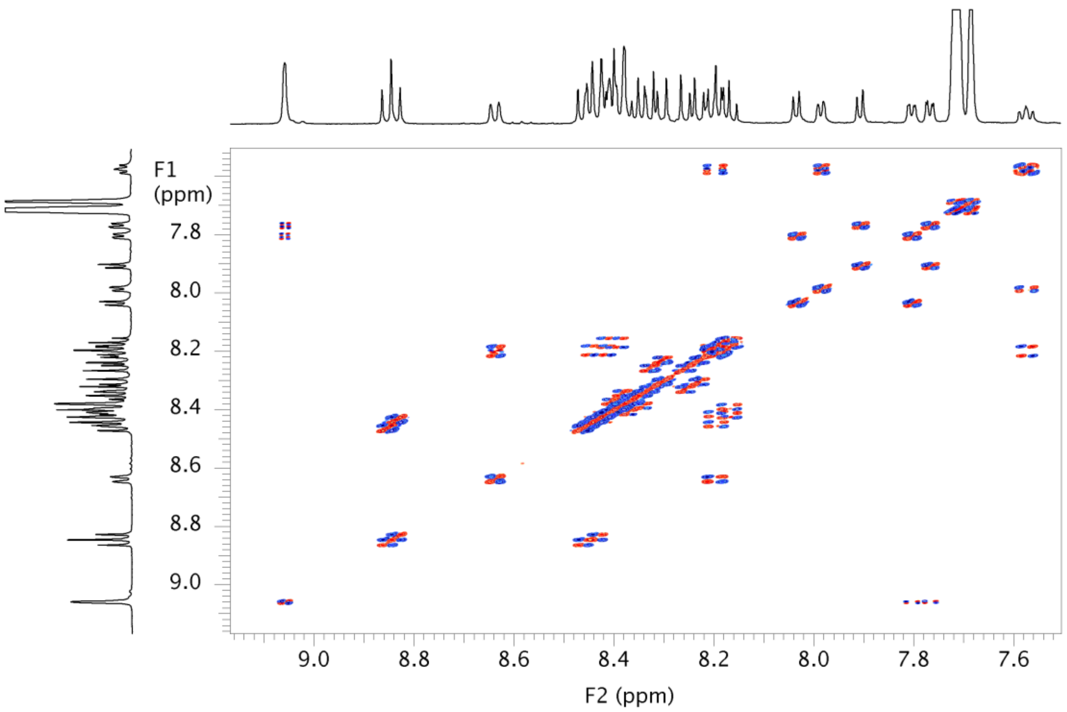

Figure S 96. ^{1}H-^{1}H gDQCOSY (298 K, 500 MHz, Acetonitrile-*d*$_3$) spectrum of compound **Ru2P·BAr^{F}$_4$**.

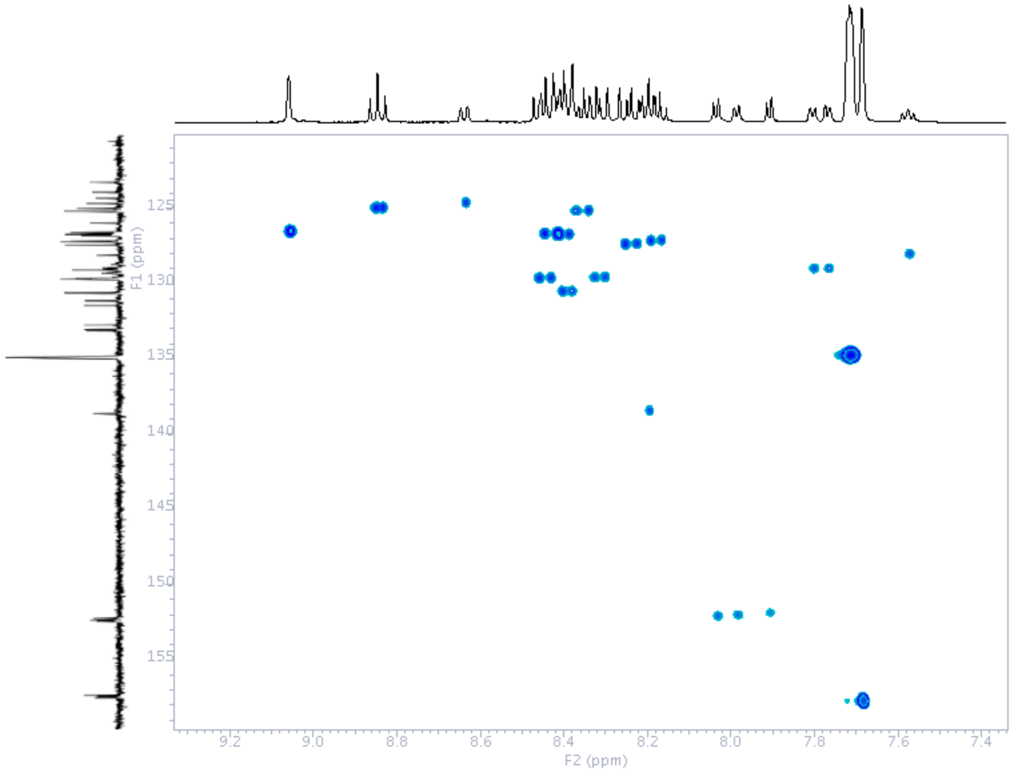

Figure S 97. ^{1}H-^{13}C gHSQCAD-PS (298 K, 500 MHz, Acetonitrile-*d*$_3$) spectrum of compound **Ru2P·BAr^{F}$_4$**.

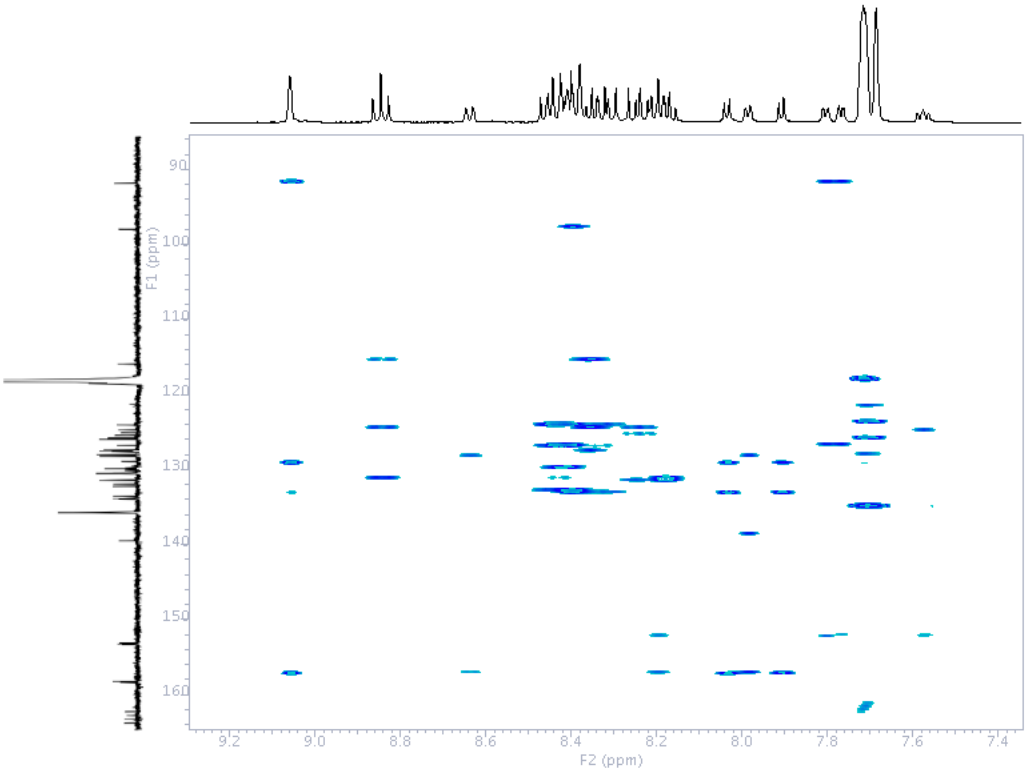

Figure S 98. ^{1}H-^{13}C bsgHMBC (298 K, 500 MHz, Acetonitrile-d_3) spectrum of compound **Ru2P·BAr$^{F}_{4}$**.

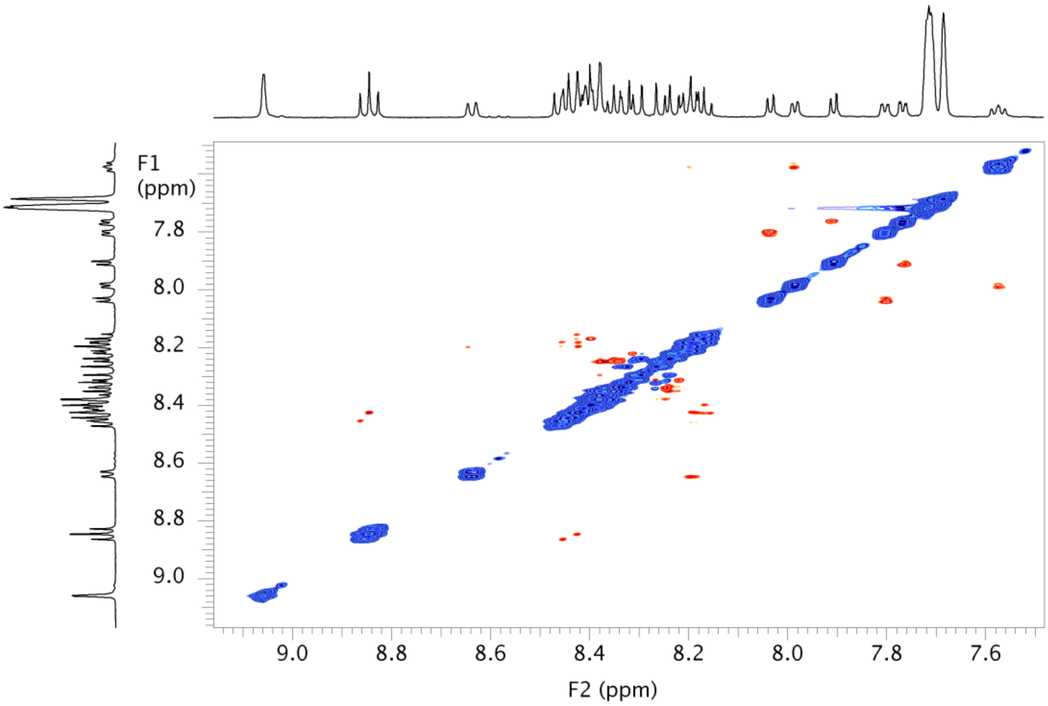

Figure S 99. ^{1}H-^{1}H ROESYAD (298 K, 500 MHz, Acetonitrile-d_3) spectrum of compound **Ru2P·BAr$^{F}_{4}$**.

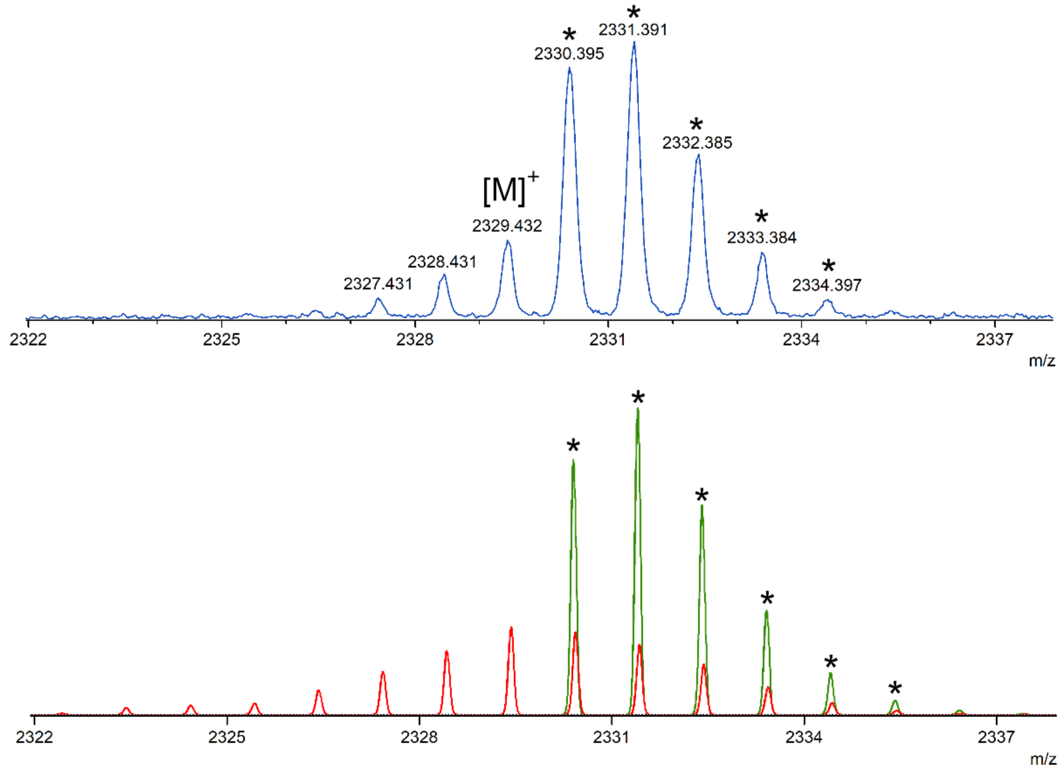

Figure S 100. HRMS (MALDI) of **Ru2P·BAr^F$_4$**, [Ru2P + BAr^F$_4$]$^+$**.** Calculated (red), measured (blue). *Signals correspond to calibration standard (PEG, in green in the calculated spectrum).

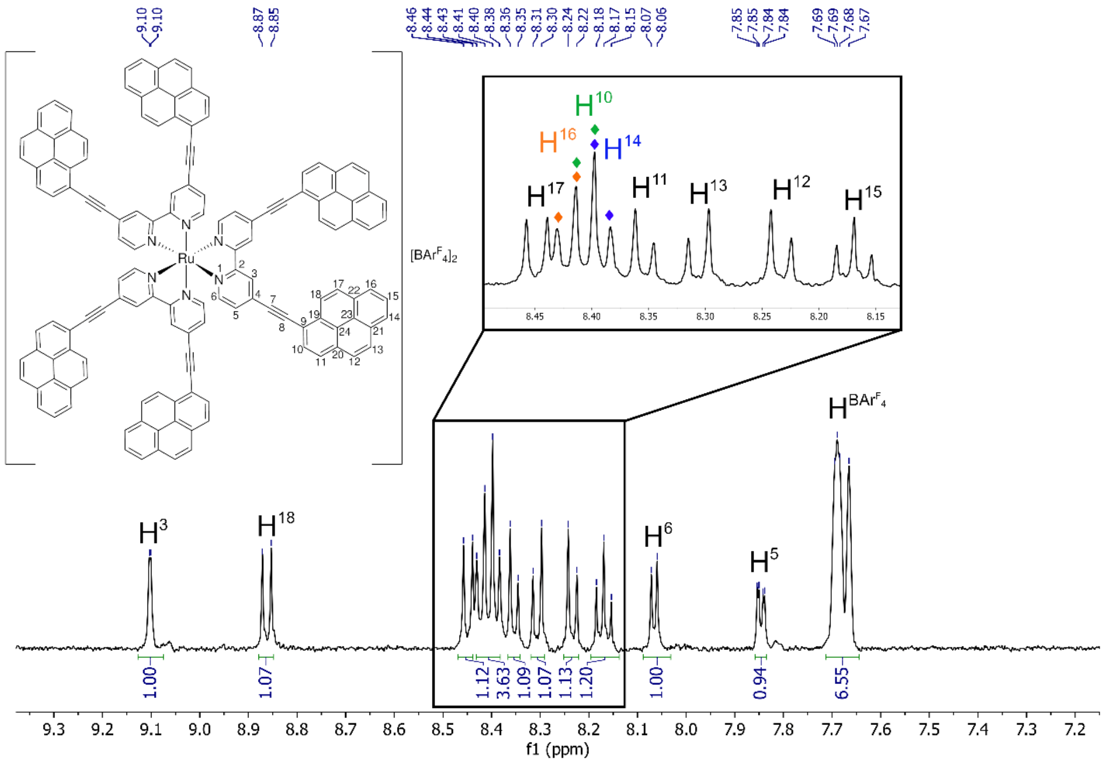

Figure S 101. ^{1}H-NMR (298 K, 500 MHz, Acetonitrile-d_3) spectrum of **Ru3P·BAr^F$_4$**.

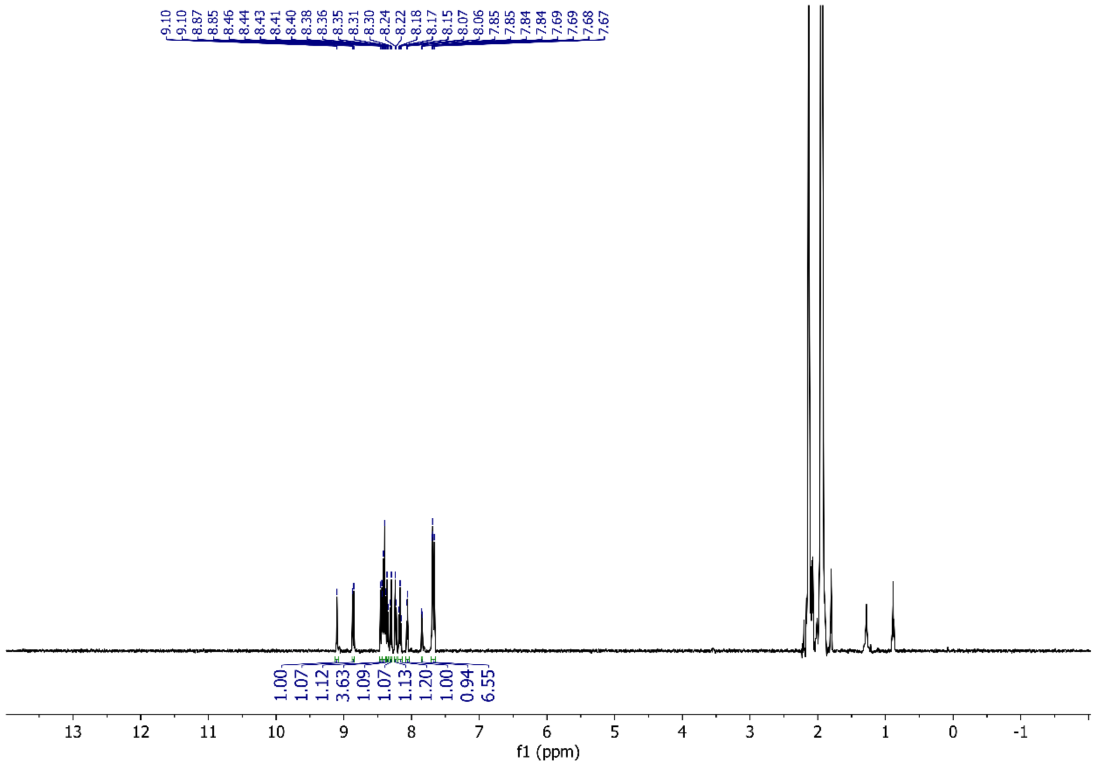

Figure S 102. Full ^{1}H-NMR (298 K, 500 MHz, Acetonitrile-d_3) spectrum of **Ru3P·BAr^{F}$_4$**.

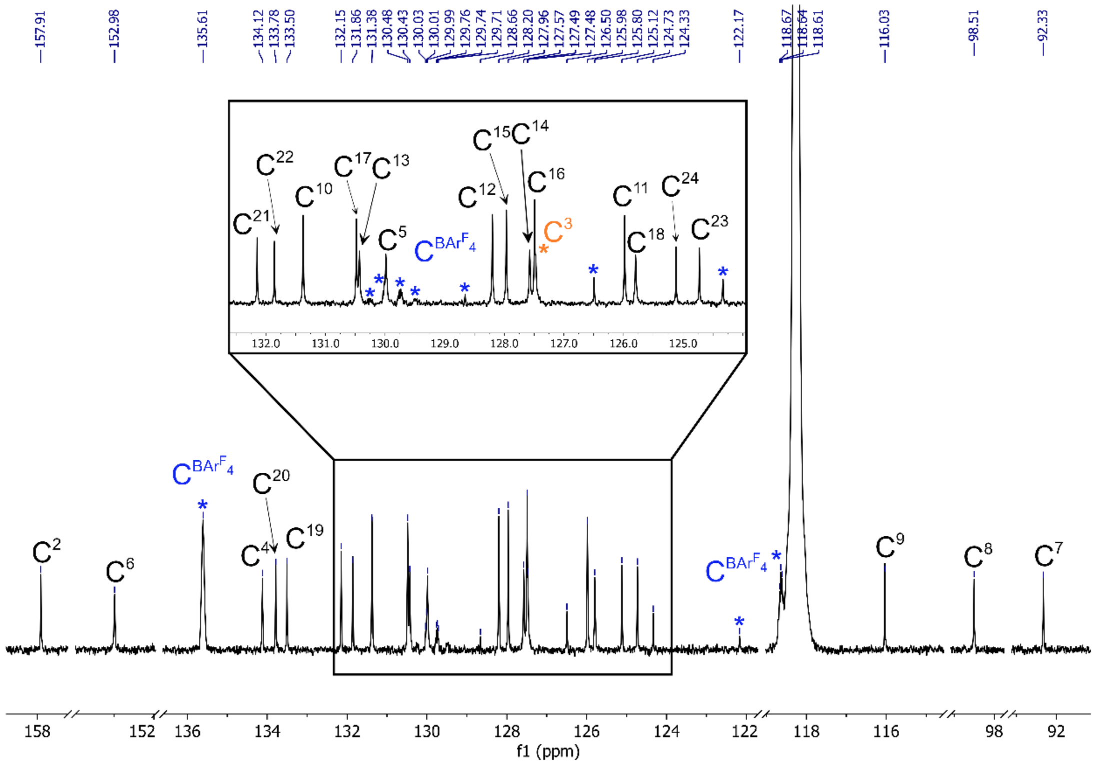

Figure S 103. ^{13}C{^{1}H}-NMR (298 K, 126 MHz, Acetonitrile-d_3) spectrum of **Ru3P·BAr^{F}$_4$**.

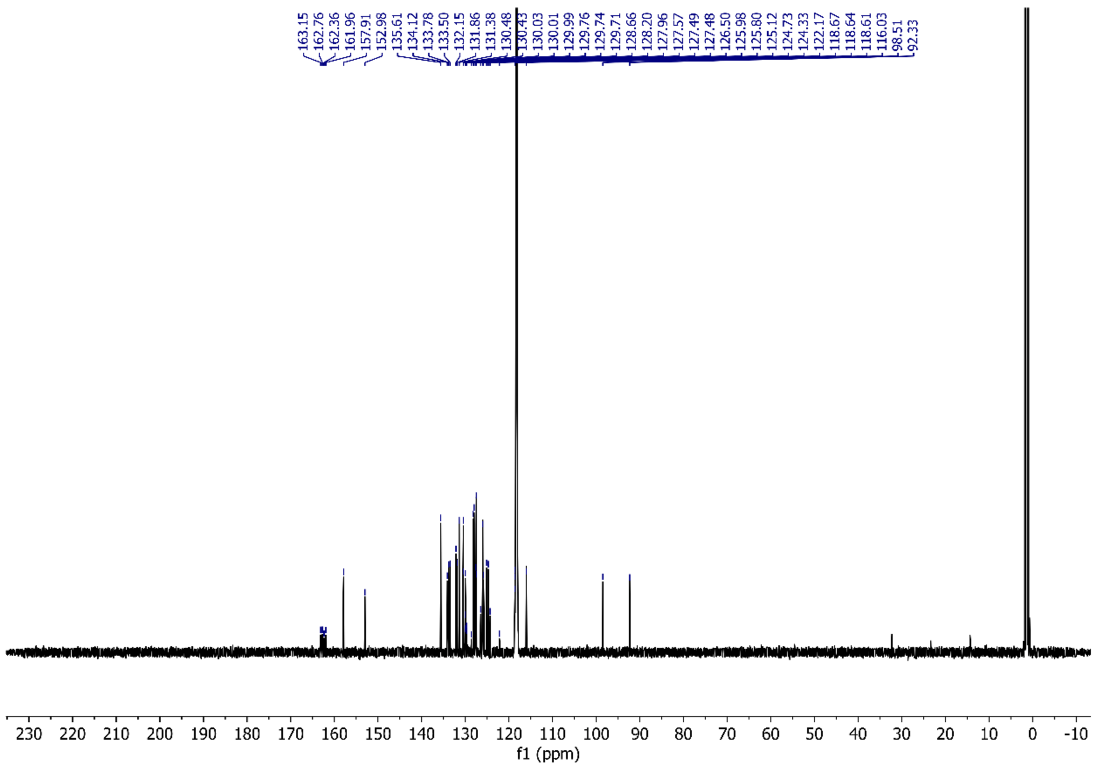

Figure S 104. Full $^{13}C\{^{1}H\}$-NMR (298 K, 126 MHz, Acetonitrile-d_3) spectrum of **Ru3P·BAr$^{F}_{4}$**.

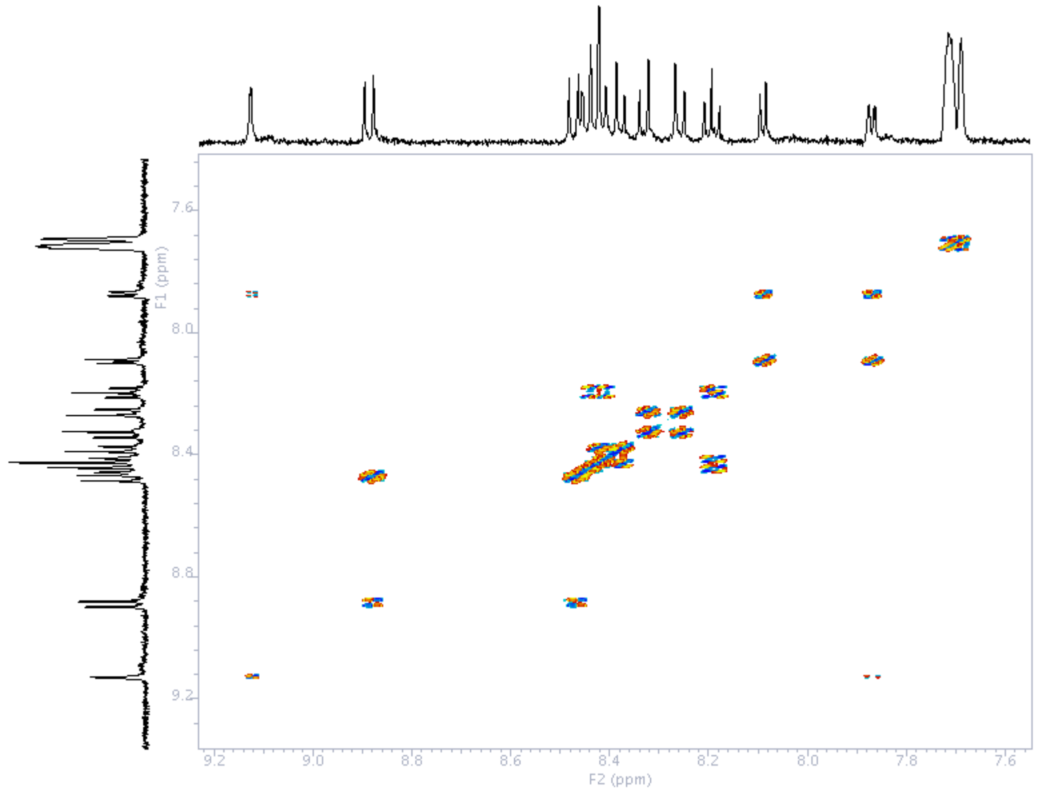

Figure S 105. ^{1}H-^{1}H gDQCOSY (298 K, 500 MHz, Acetonitrile-d_3) spectrum of compound **Ru3P·BAr$^{F}_{4}$**.

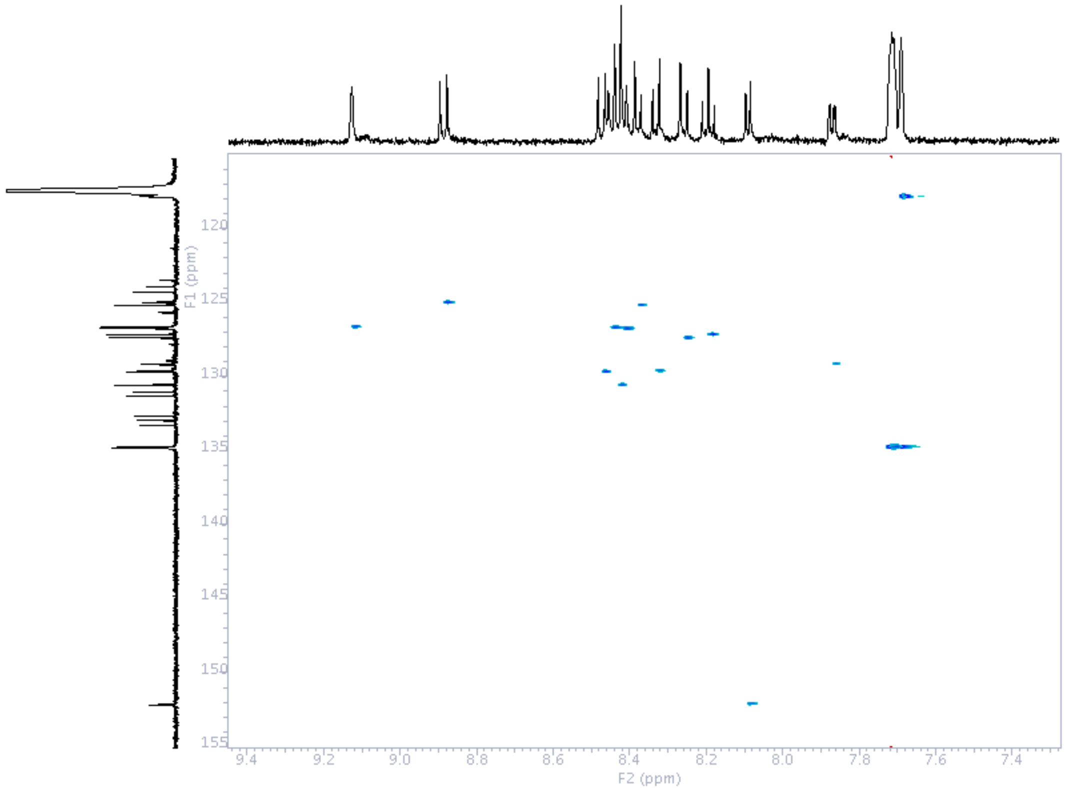

Figure S 106. ^{1}H-^{13}C gHSQCAD-PS (298 K, 500 MHz, Acetonitrile-*d*$_3$) spectrum of compound **Ru3P·BAr^{F}$_4$**.

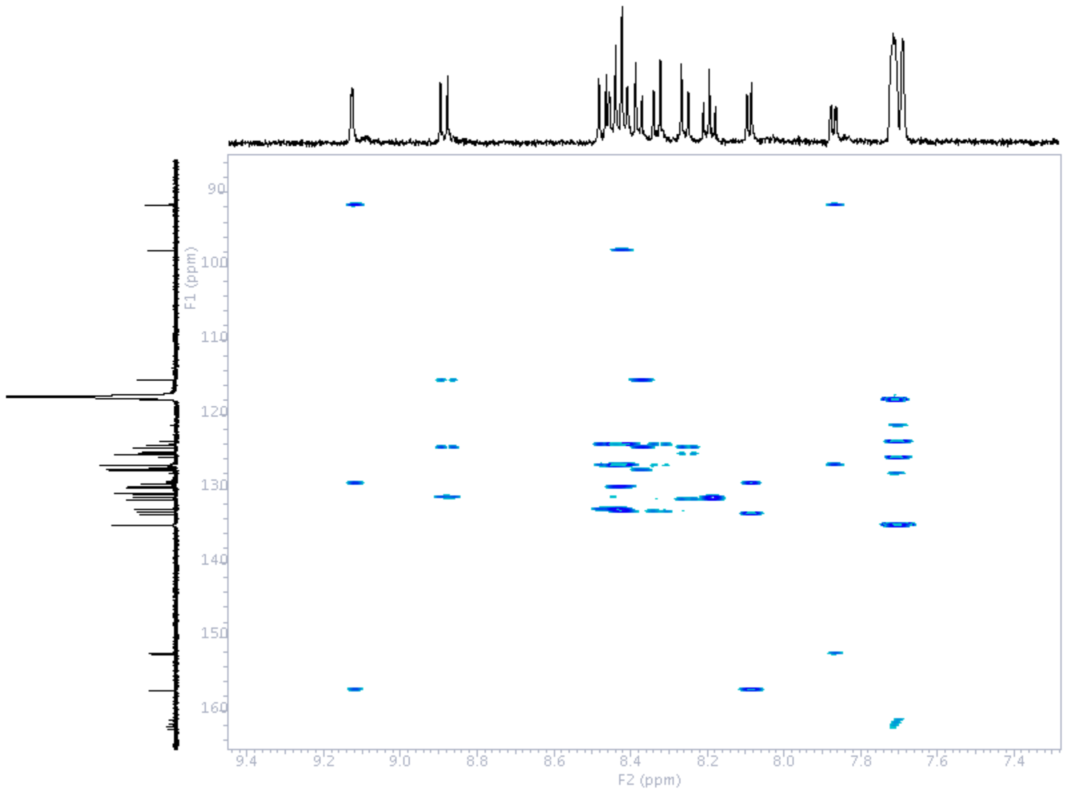

Figure S 107. ^{1}H-^{13}C bsgHMBC (298 K, 500 MHz, Acetonitrile-*d*$_3$) spectrum of compound **Ru3P·BAr^{F}$_4$**.

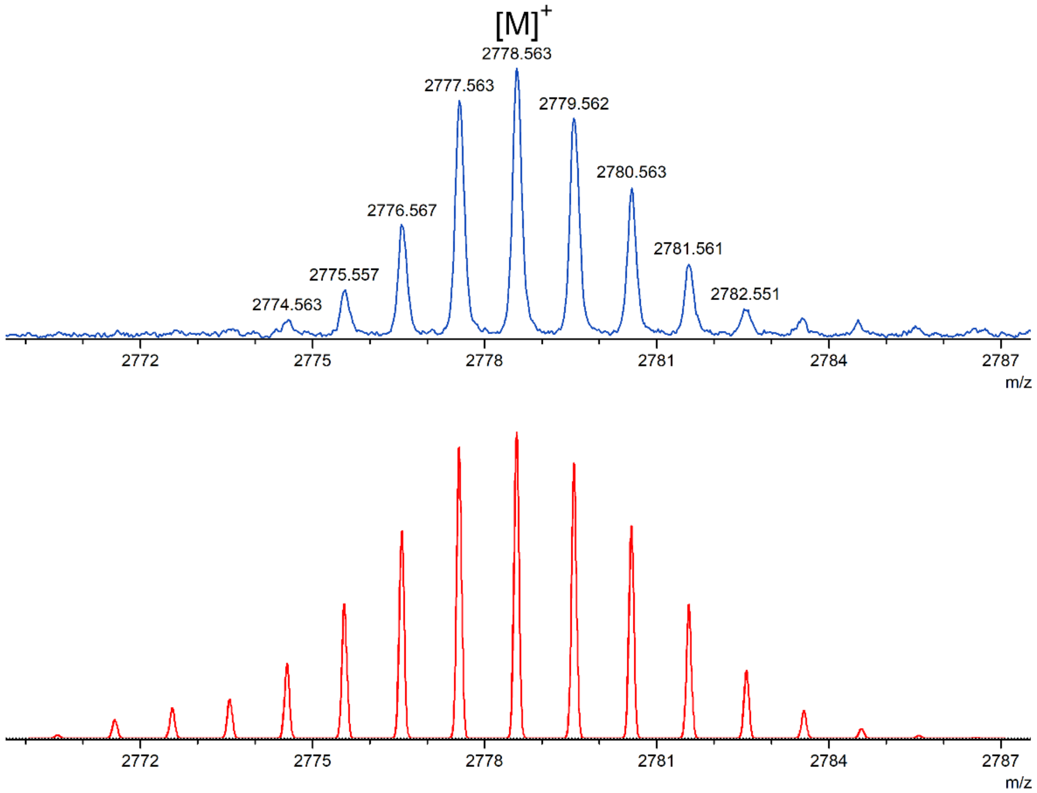

Figure S 108. HRMS (MALDI) of **Ru3P·BAr^{F}**$_4$, [Ru3P + BAr^{F}$_4$]$^+$**.** Calculated (red), measured (blue).

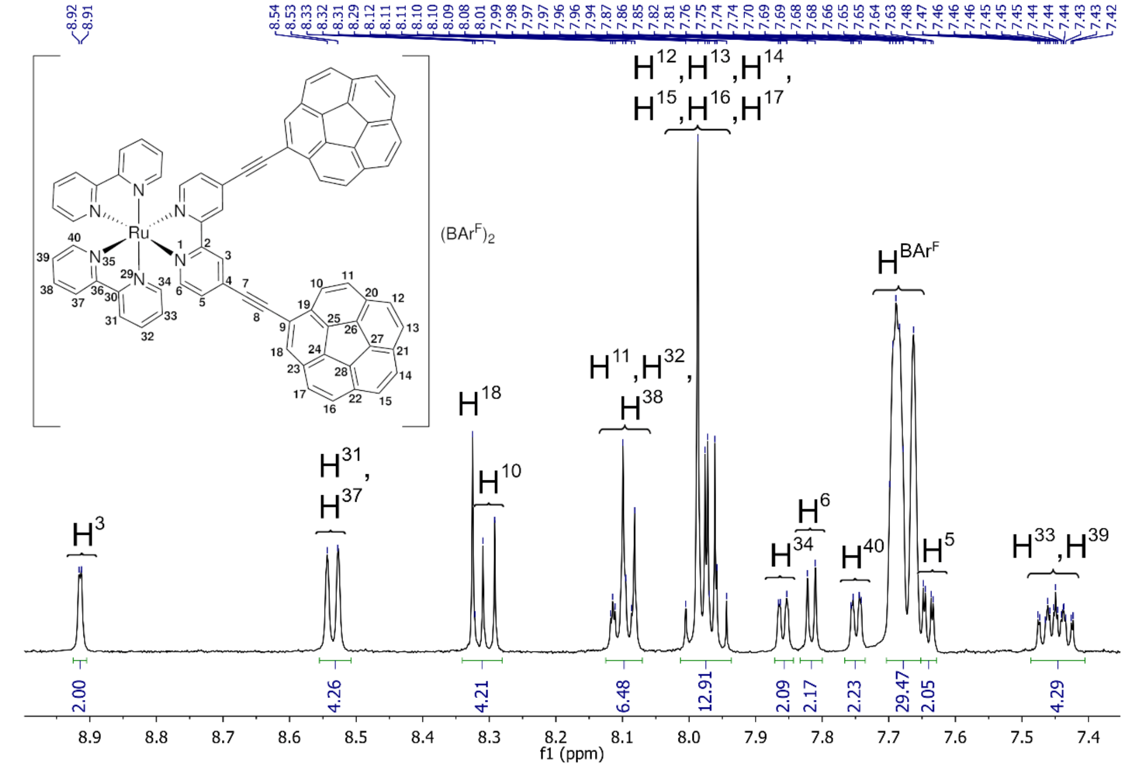

Figure S 109. ^{1}H-NMR (298 K, 500 MHz, Acetonitrile-d_3) spectrum of **Ru1C·BAr^F$_4$**.

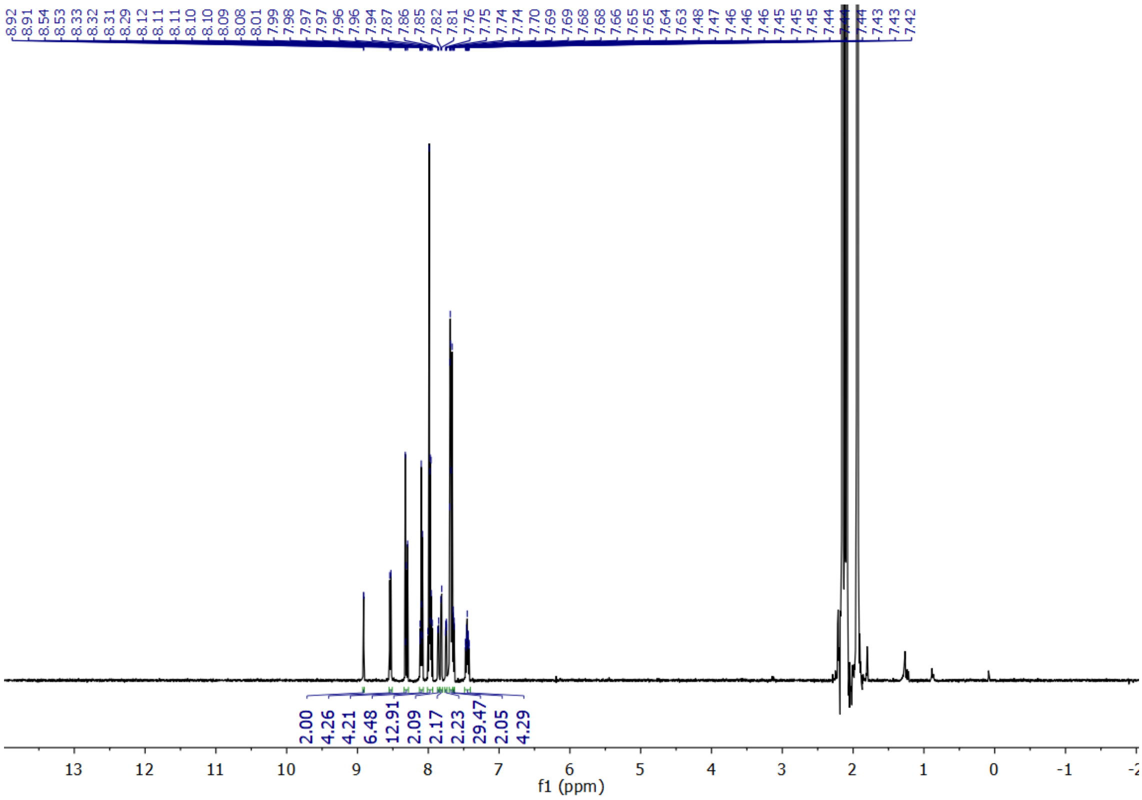

Figure S 110. Full ^{1}H-NMR (298 K, 500 MHz, Acetonitrile-d_3) spectrum of **Ru1C·BAr^F$_4$**.

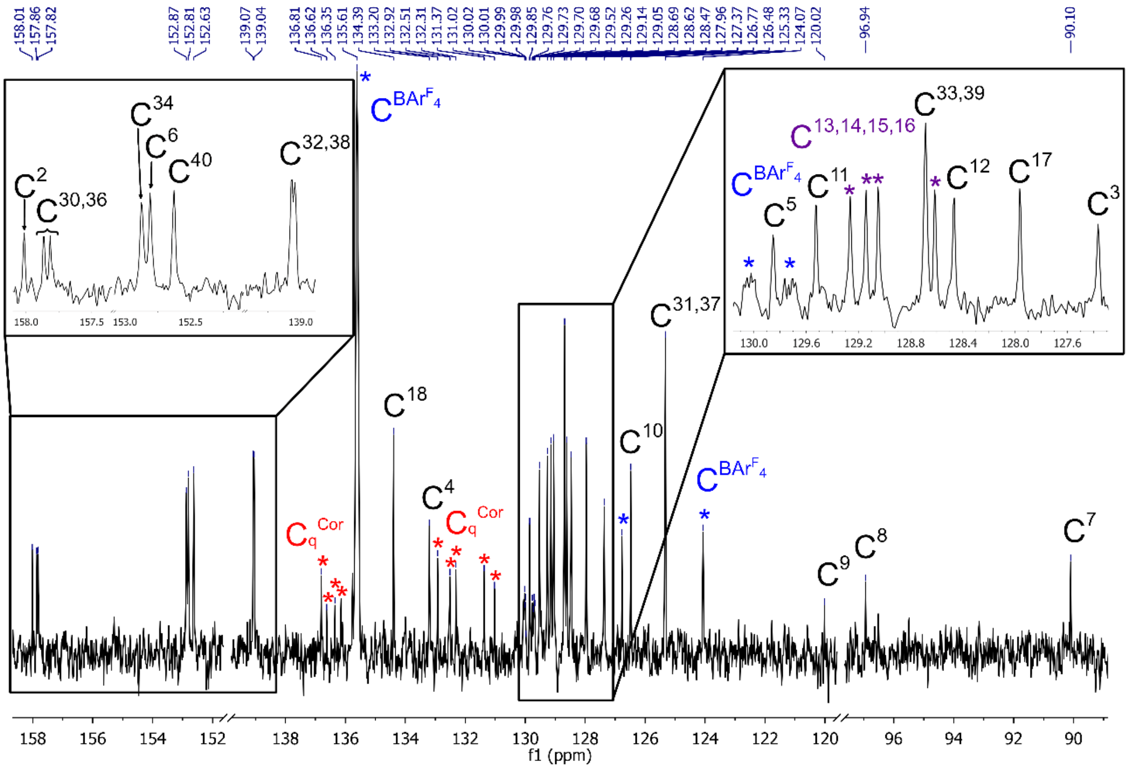

Figure S 111. $^{13}C\{^1H\}$-NMR (298 K, 101 MHz, Acetonitrile-d_3) spectrum of **Ru1C·BArF4**.

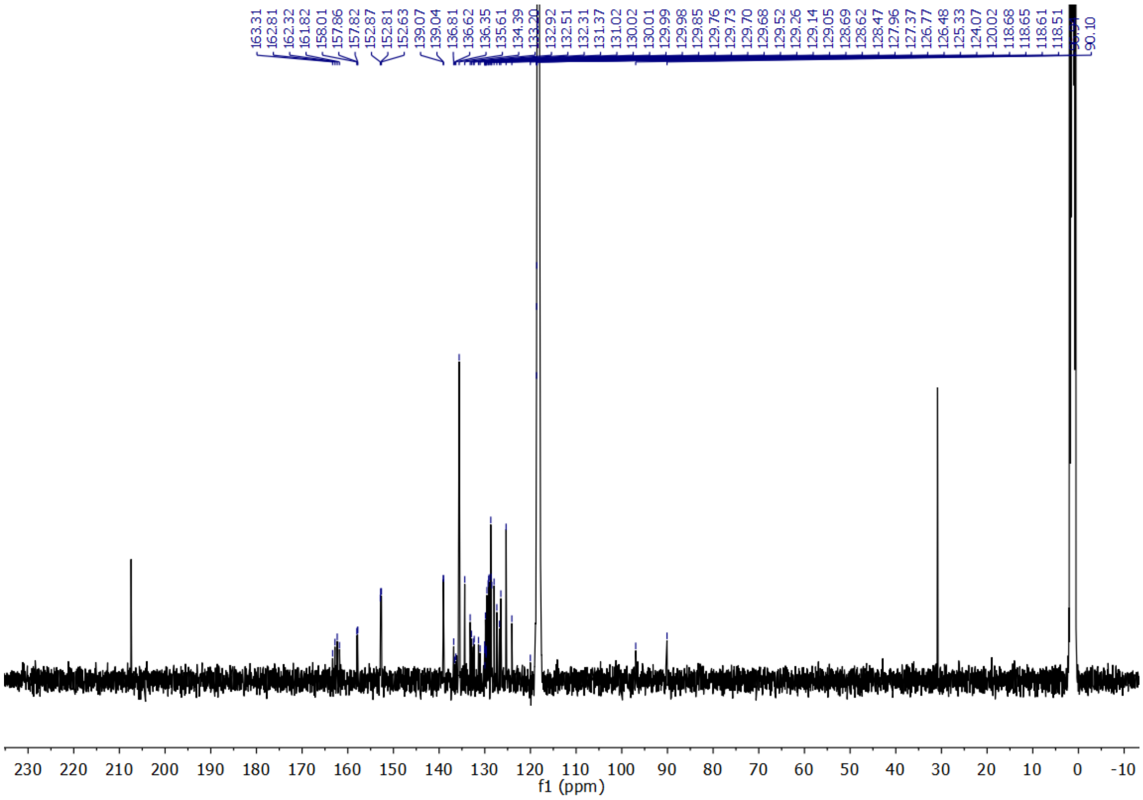

Figure S 112. Full $^{13}C\{^1H\}$-NMR (298 K, 101 MHz, Acetonitrile-d_3) spectrum of **Ru1C·BArF4**.

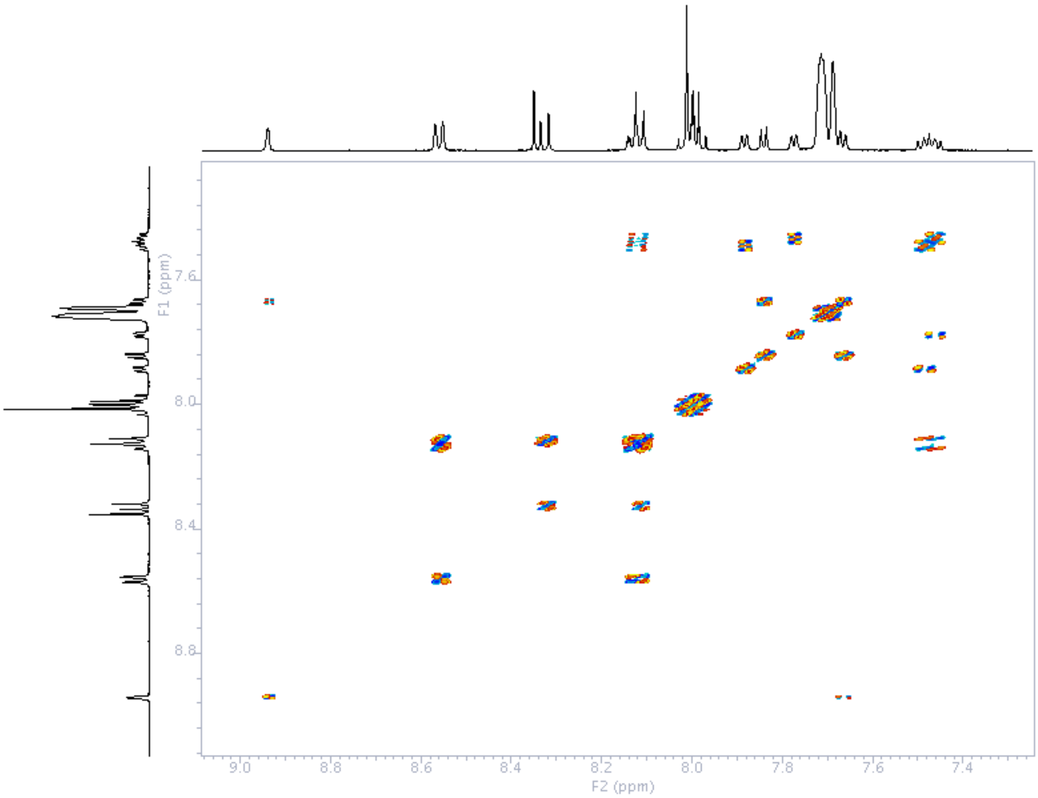

Figure S 113. ^{1}H-^{1}H gDQCOSY (298 K, 500 MHz, Acetonitrile-d_3) spectrum of compound **Ru1C·BAr^{F}$_4$**.

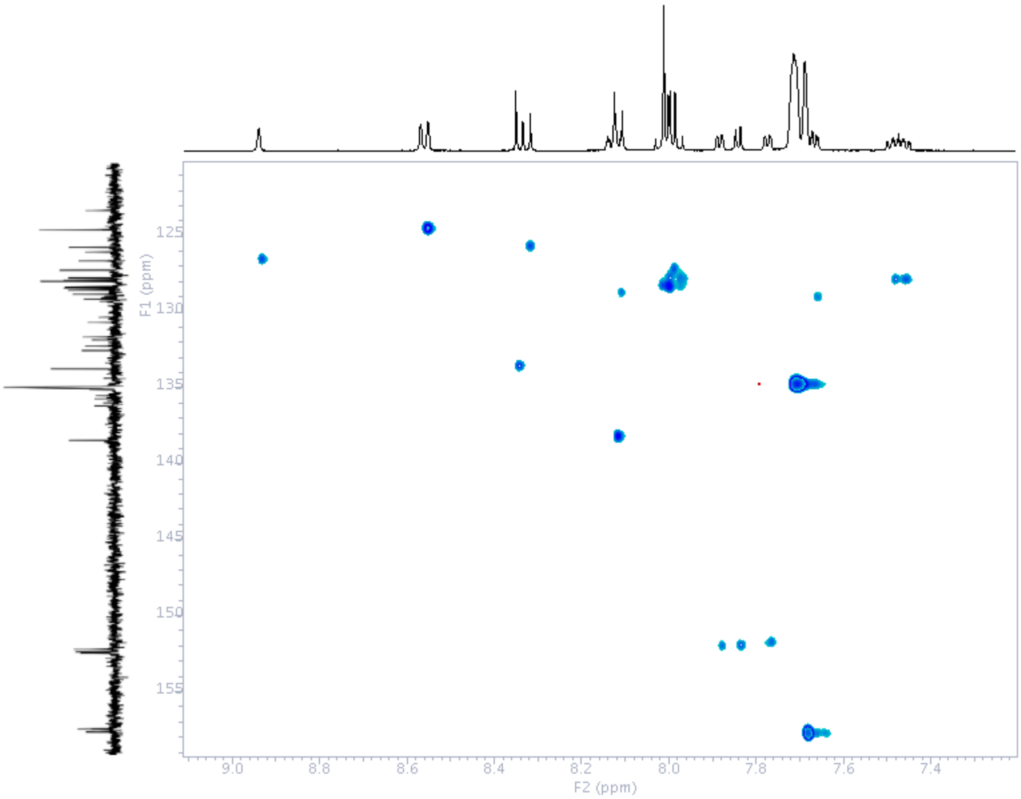

Figure S 114. ^{1}H-^{13}C gHSQCAD-PS (298 K, 500 MHz, Acetonitrile-d_3) spectrum of compound **Ru1C·BAr^{F}$_4$**.

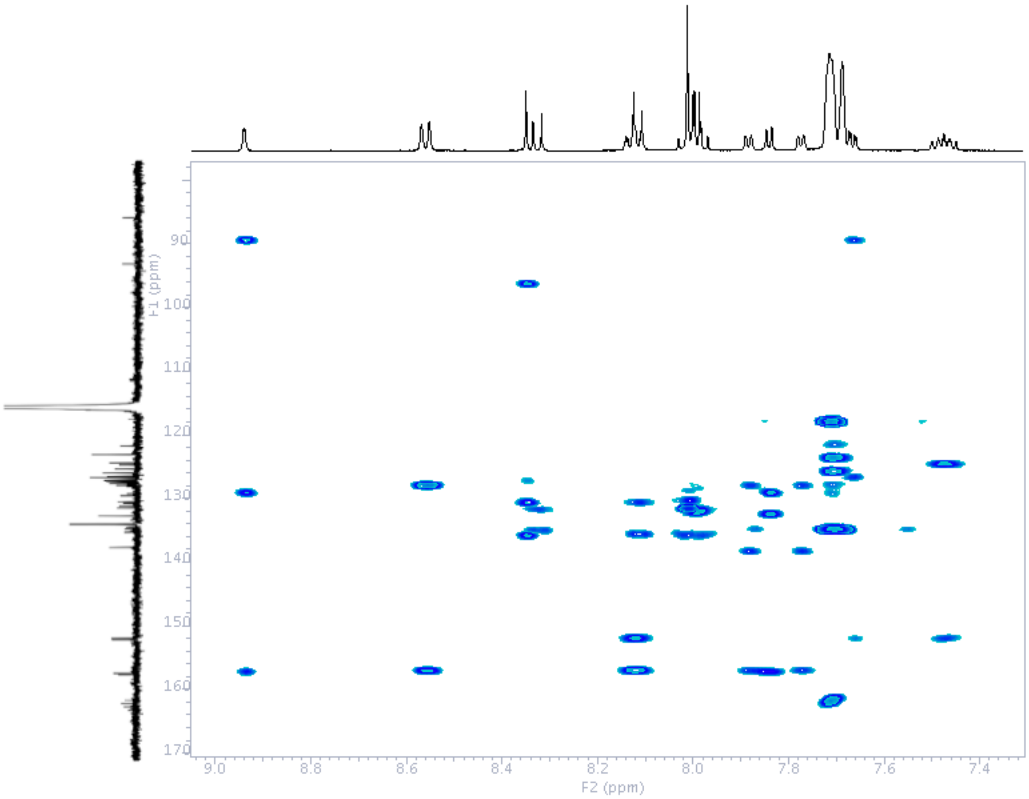

Figure S 115. ^{1}H-^{13}C gc2HMBC (298 K, 500 MHz, Acetonitrile-d_3) spectrum of compound **Ru1C·BAr^{F}$_4$**.

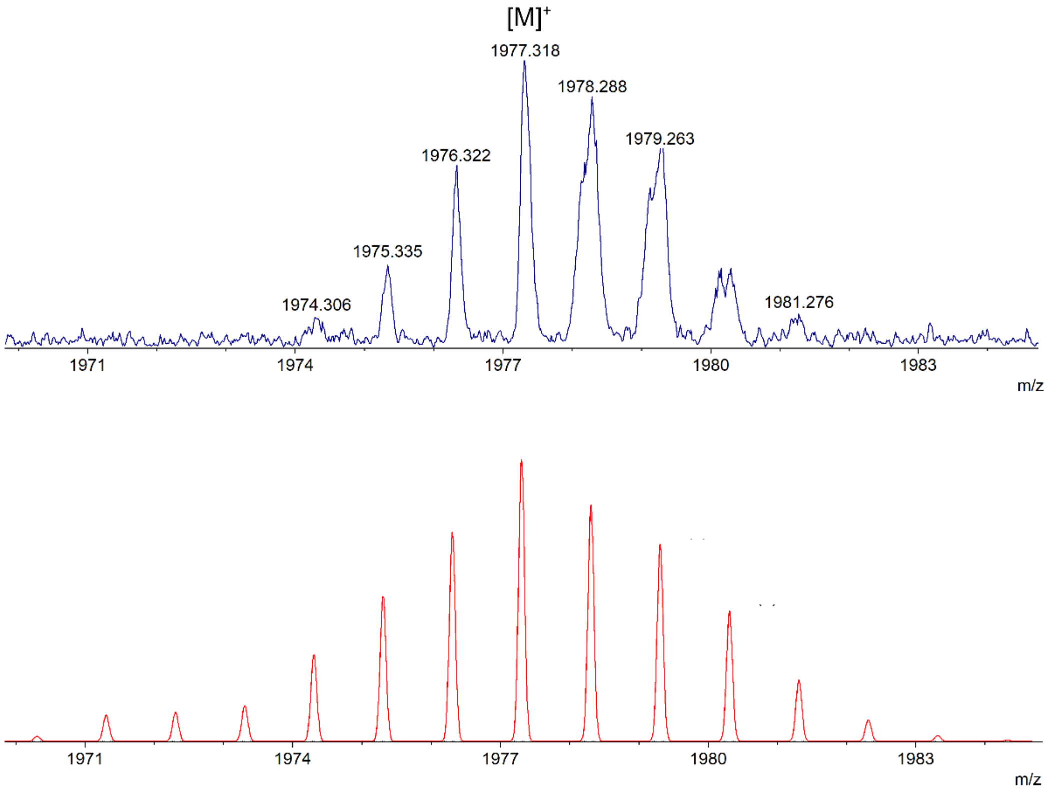

Figure S 116. HRMS (MALDI) of **Ru1C·BAr^{F}$_4$**, [Ru1C + BAr^{F}$_4$]$^+$. Calculated (red), measured (blue).

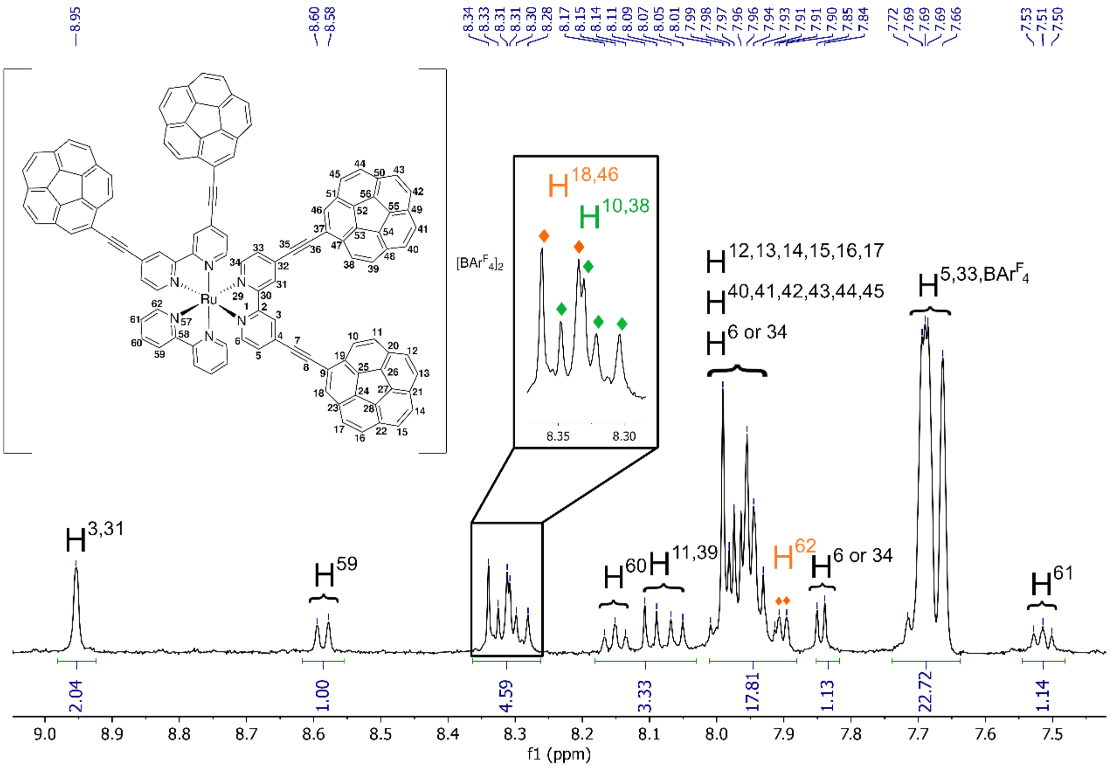

Figure S 117. ^{1}H-NMR (298 K, 500 MHz, Acetonitrile-d_3) spectrum of **Ru2C·BAr$^F_4$**.

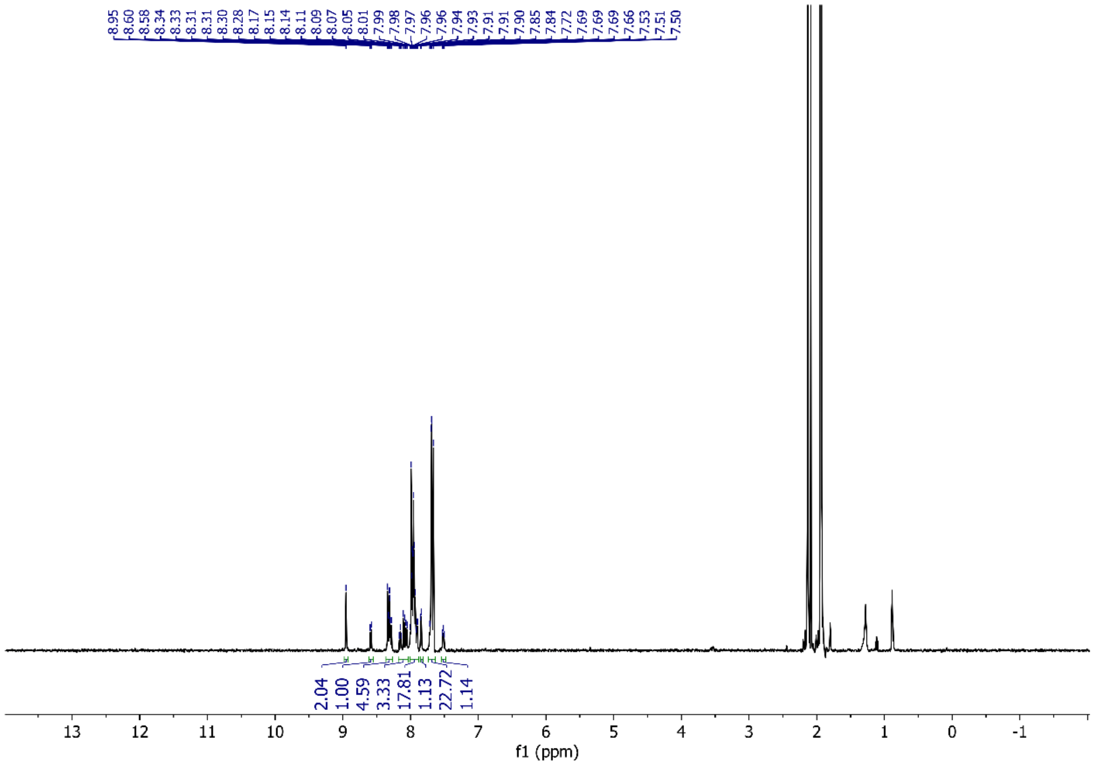

Figure S 118. Full ^{1}H-NMR (298 K, 500 MHz, Acetonitrile-d_3) spectrum of **Ru2C·BAr$^F_4$**.

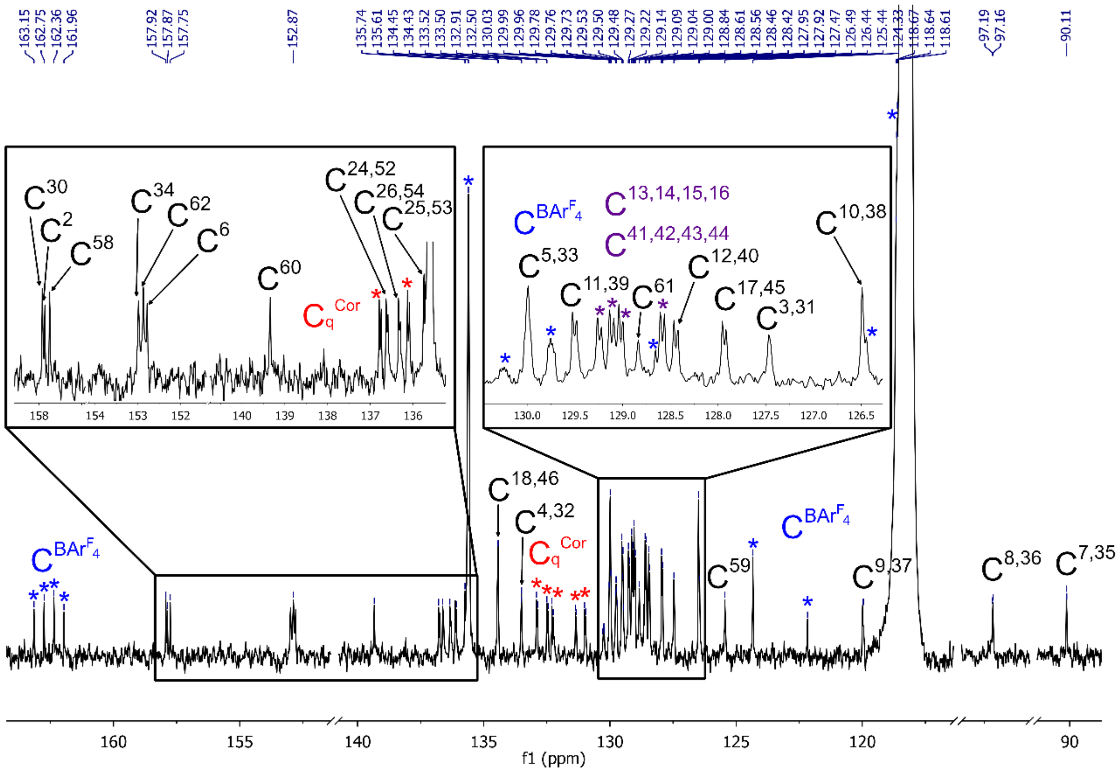

Figure S 119. $^{13}C\{^1H\}$-NMR (298 K, 126 MHz, Acetonitrile-d_3) spectrum of **Ru2C·BAr$^F_4$**.

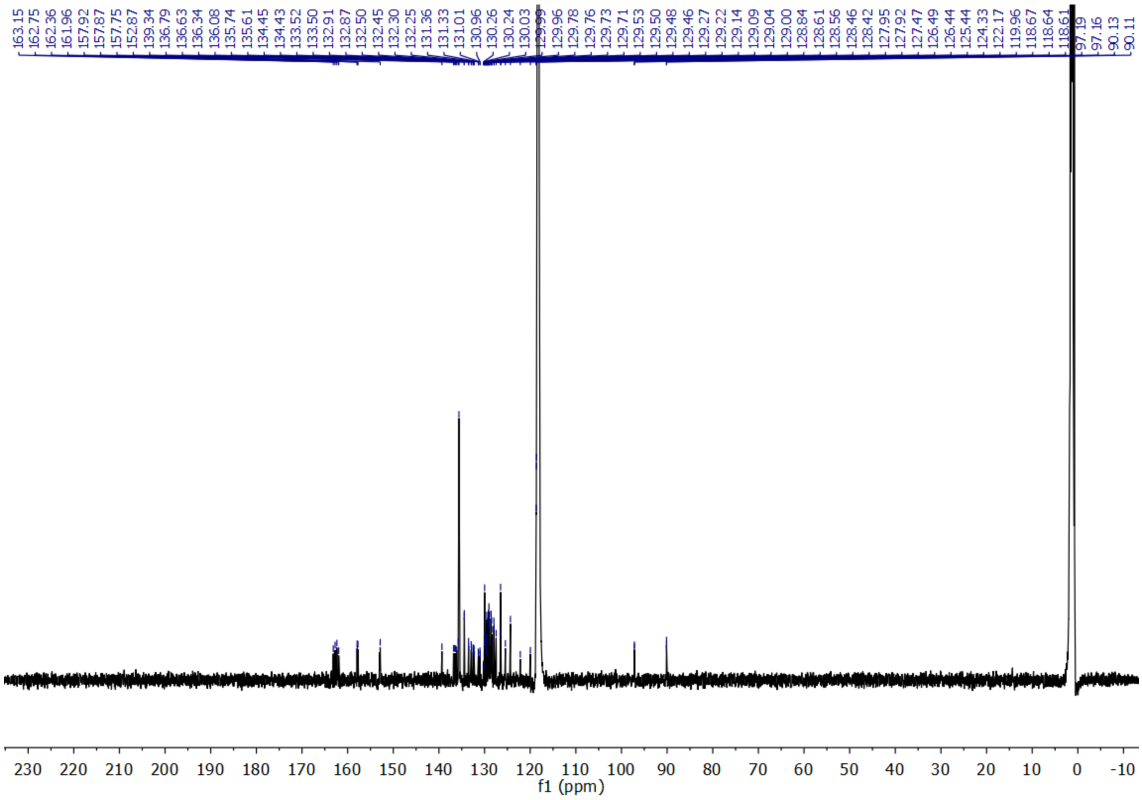

Figure S 120. Full $^{13}C\{^1H\}$-NMR (298 K, 126 MHz, Acetonitrile-d_3) spectrum of **Ru2C·BAr$^F_4$**.

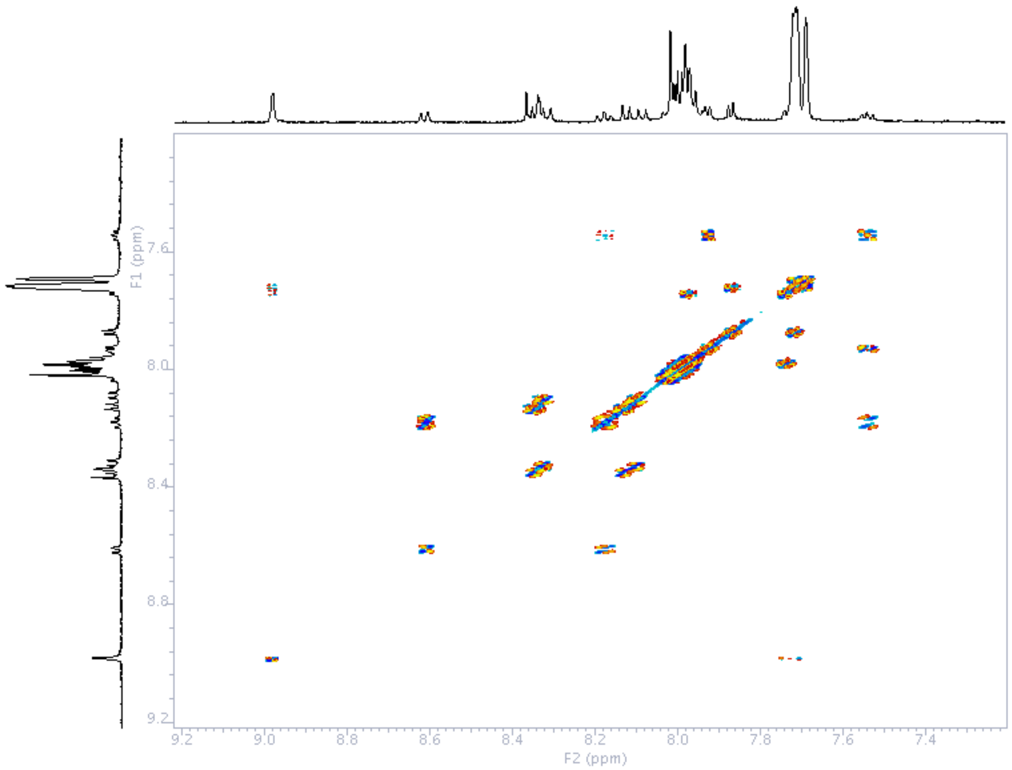

Figure S 121. ^{1}H-^{1}H gDQCOSY (298 K, 500 MHz, Acetonitrile-d_3) spectrum of compound **Ru2C·BAr$^{F}_{4}$**.

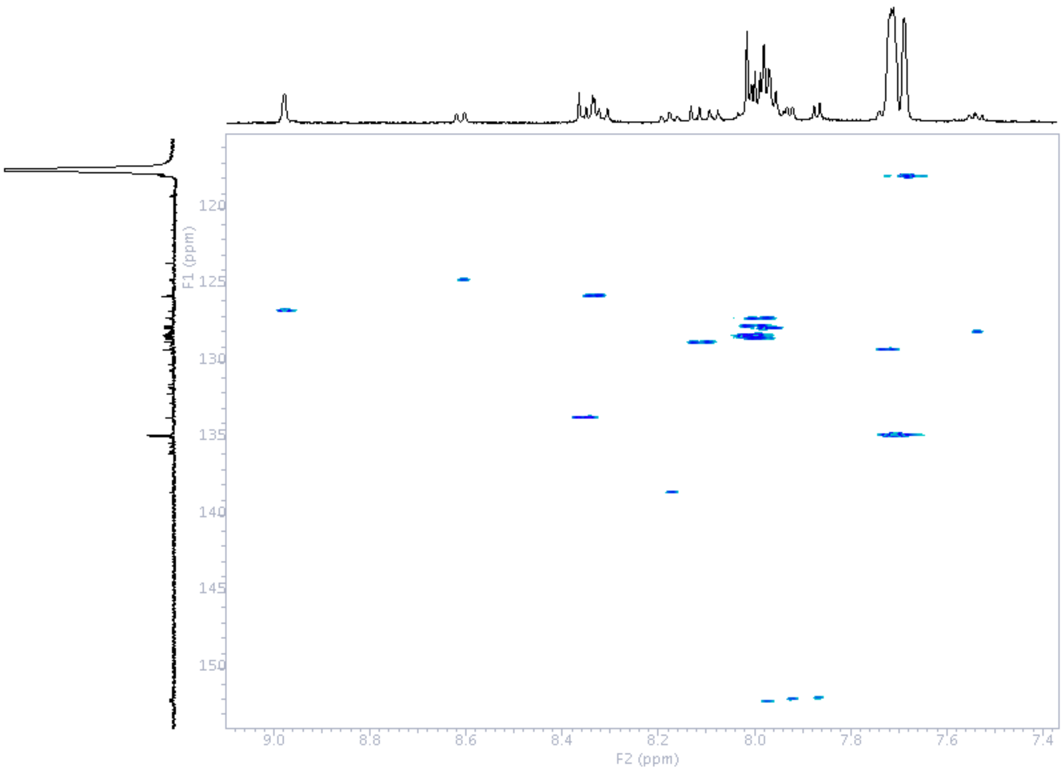

Figure S 122. ^{1}H-^{13}C gHSQCAD-PS (298 K, 500 MHz, Acetonitrile-d_3) spectrum of compound **Ru2C·BAr$^{F}_{4}$**.

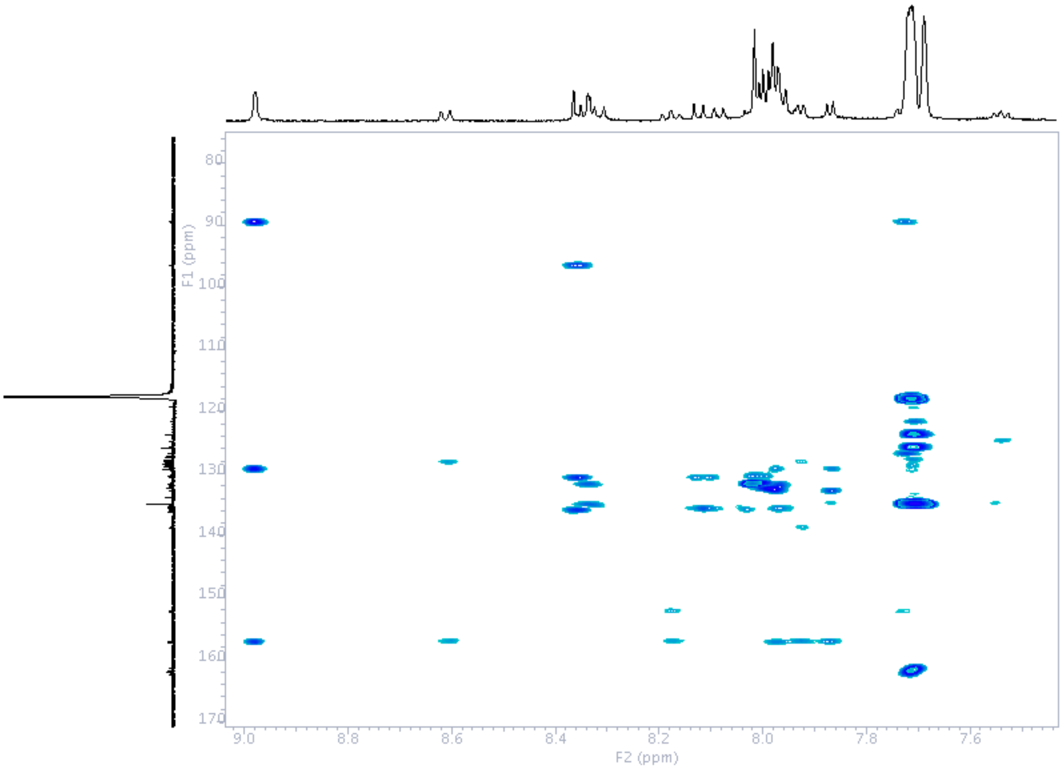

Figure S 123. ^{1}H-^{13}C gc2HMBC (298 K, 500 MHz, Acetonitrile-d_3) spectrum of compound **Ru2C·BAr$^{F}_{4}$**.

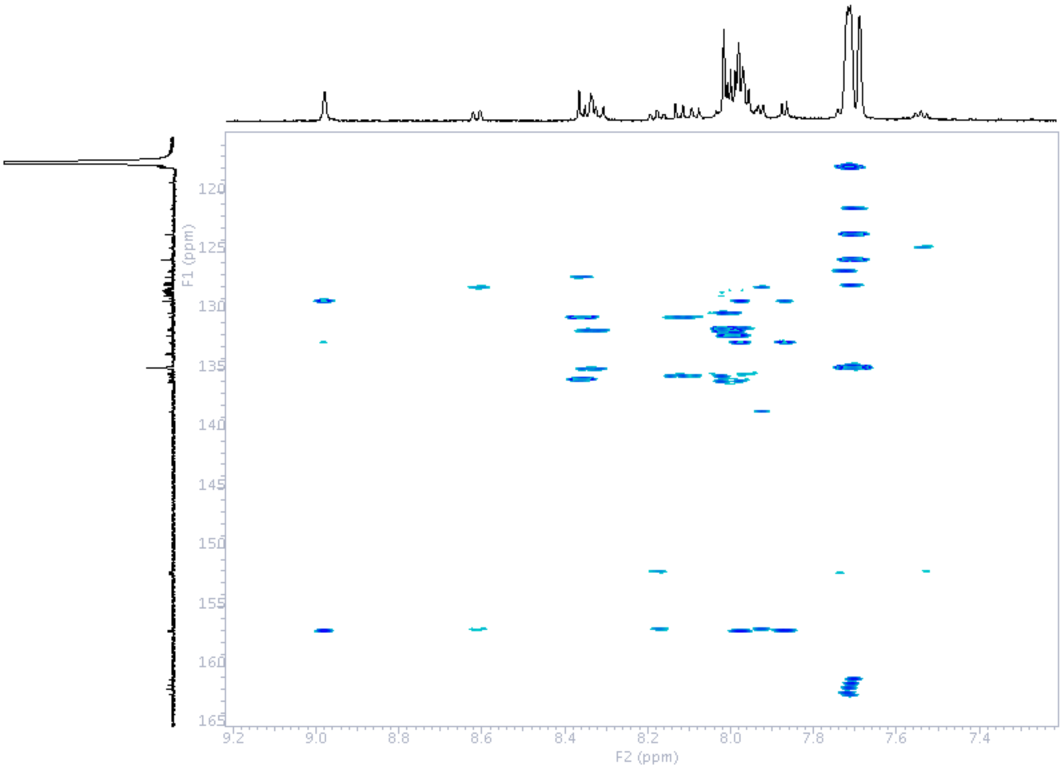

Figure S 124. ^{1}H-^{13}C bsgHMBC (298 K, 500 MHz, Acetonitrile-d_3) spectrum of compound **Ru2C·BAr$^{F}_{4}$**.

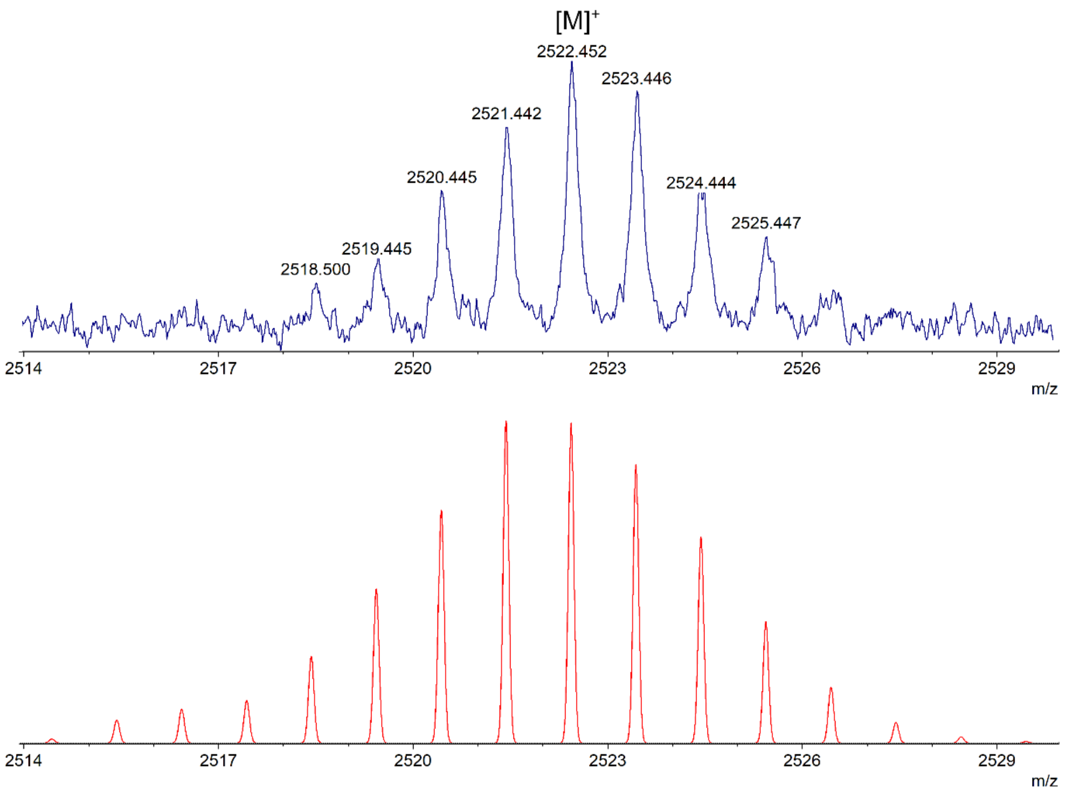

Figure S 125. HRMS (MALDI) of **Ru2C·BAr^{F}$_4$**, [Ru2C + BAr^{F}$_4$]$^+$**.** Calculated (red), measured (blue).

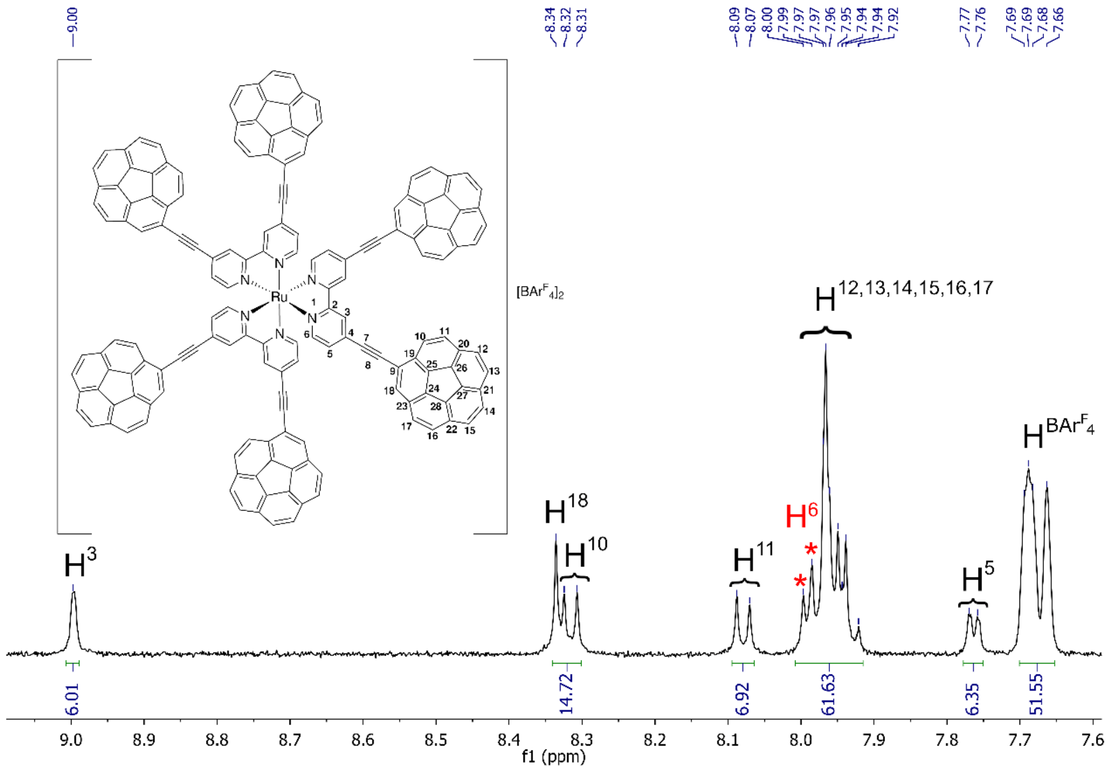

Figure S 126. ^{1}H-NMR (298 K, 500 MHz, Acetonitrile-d_3) spectrum of **Ru3C·BAr$^F{}_4$**.

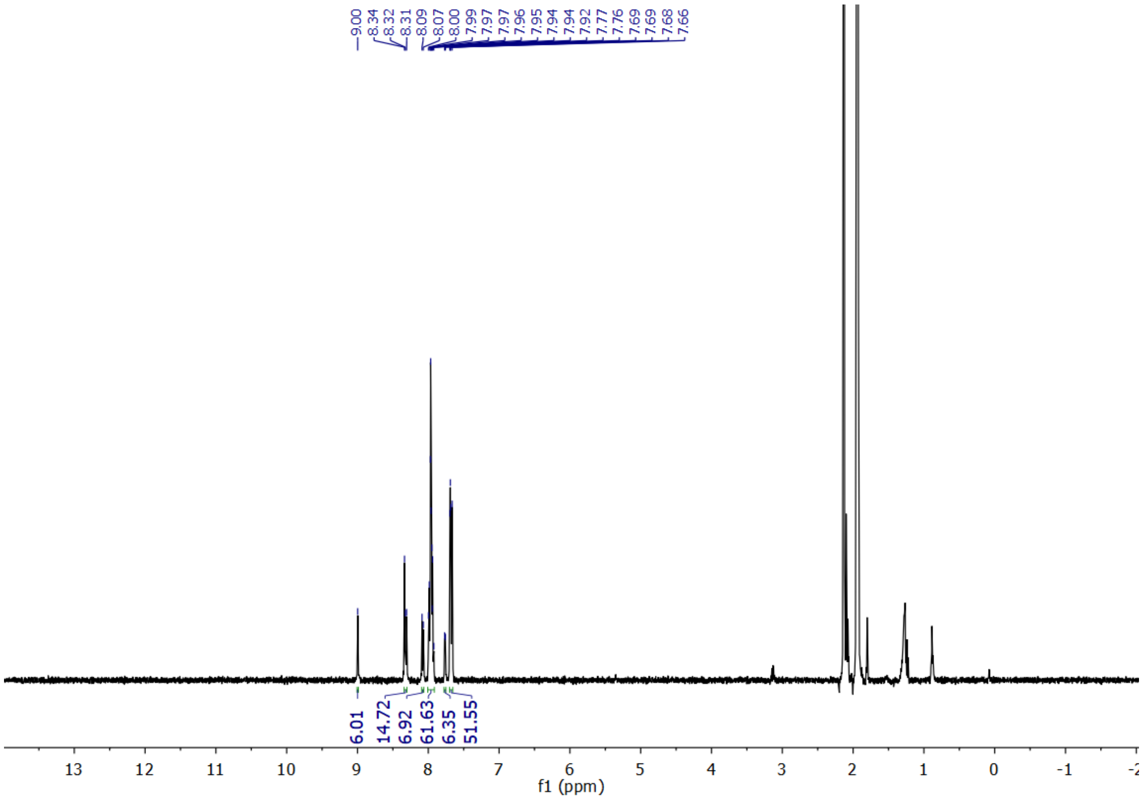

Figure S 127. Full ^{1}H-NMR (298 K, 500 MHz, Acetonitrile-d_3) spectrum of **Ru3C·BAr$^F{}_4$**.

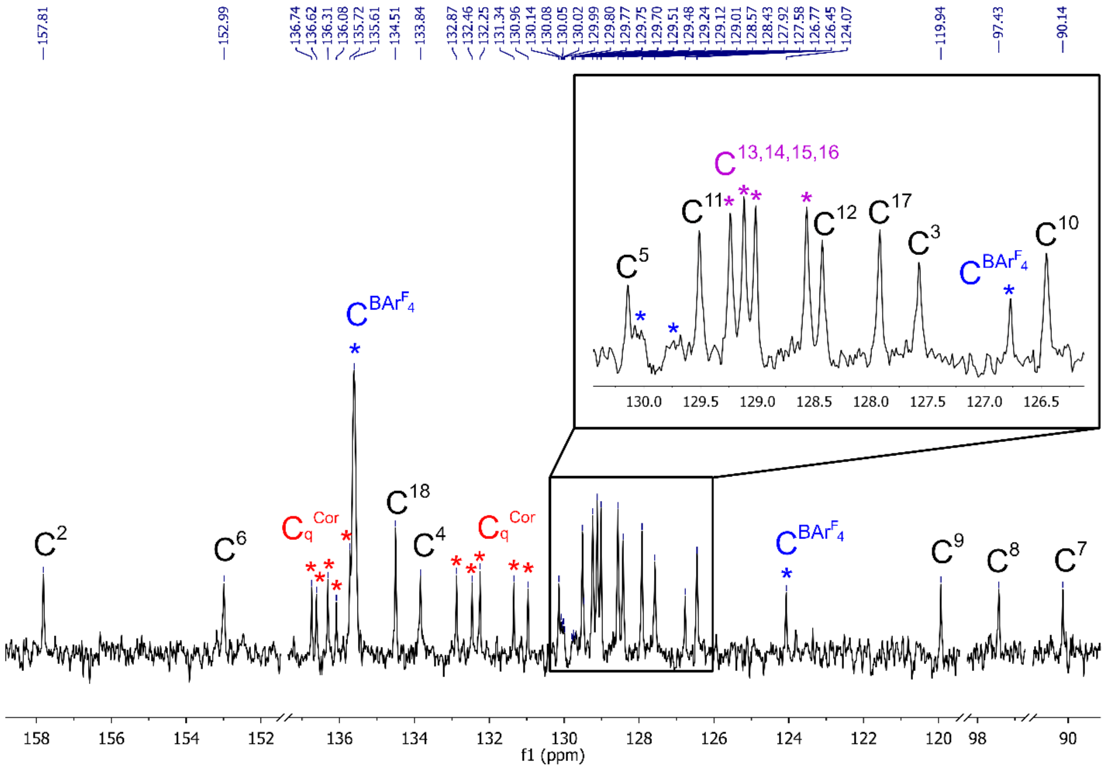

Figure S 128. $^{13}C\{^{1}H\}$-NMR (298 K, 101 MHz, Acetonitrile-d_3) spectrum of **Ru3C·BArF$_4$**.

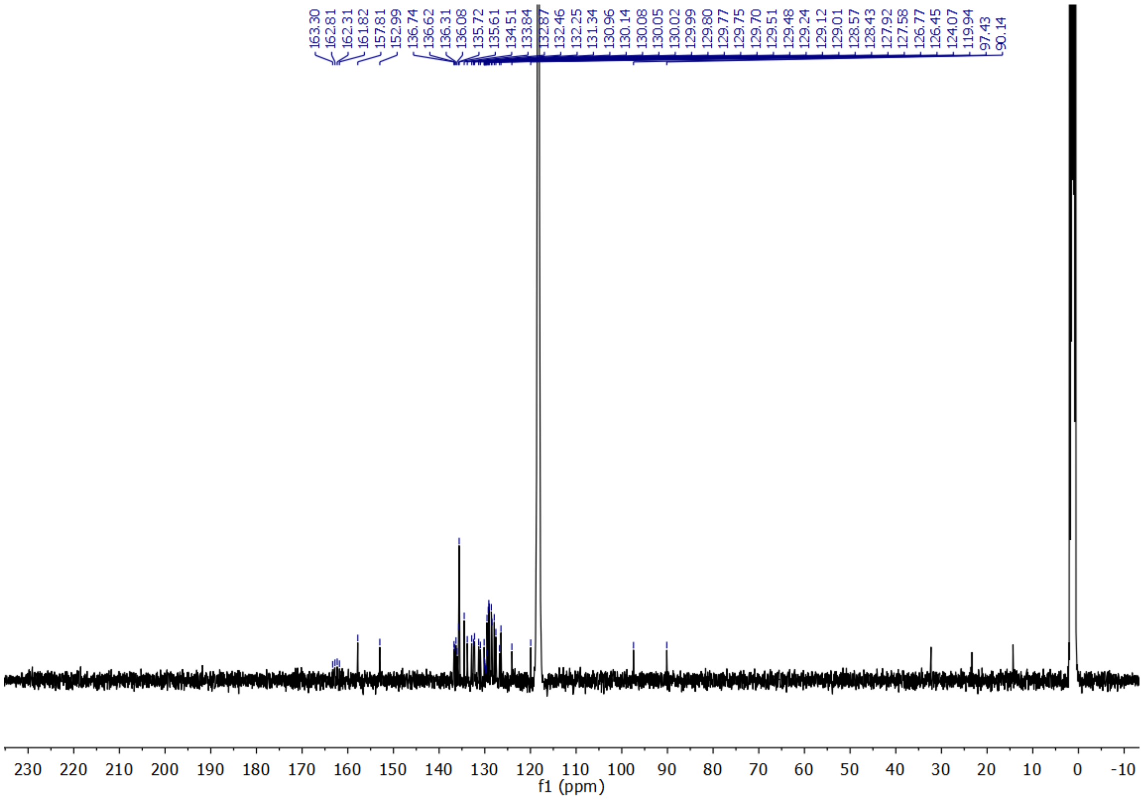

Figure S 129. Full $^{13}C\{^{1}H\}$-NMR (298 K, 101 MHz, Acetonitrile-d_3) spectrum of **Ru3C·BArF$_4$**.

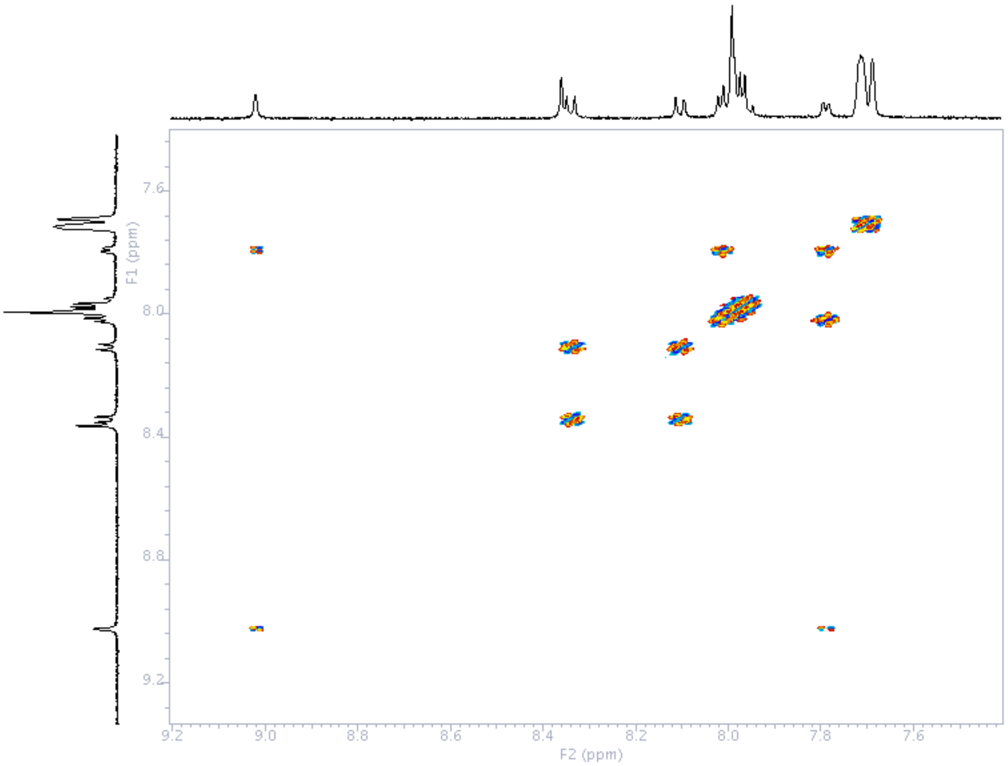

Figure S 130. ^{1}H-^{1}H gDQCOSY (298 K, 500 MHz, Acetonitrile-d_3) spectrum of compound **Ru3C·BAr^{F}$_4$**.

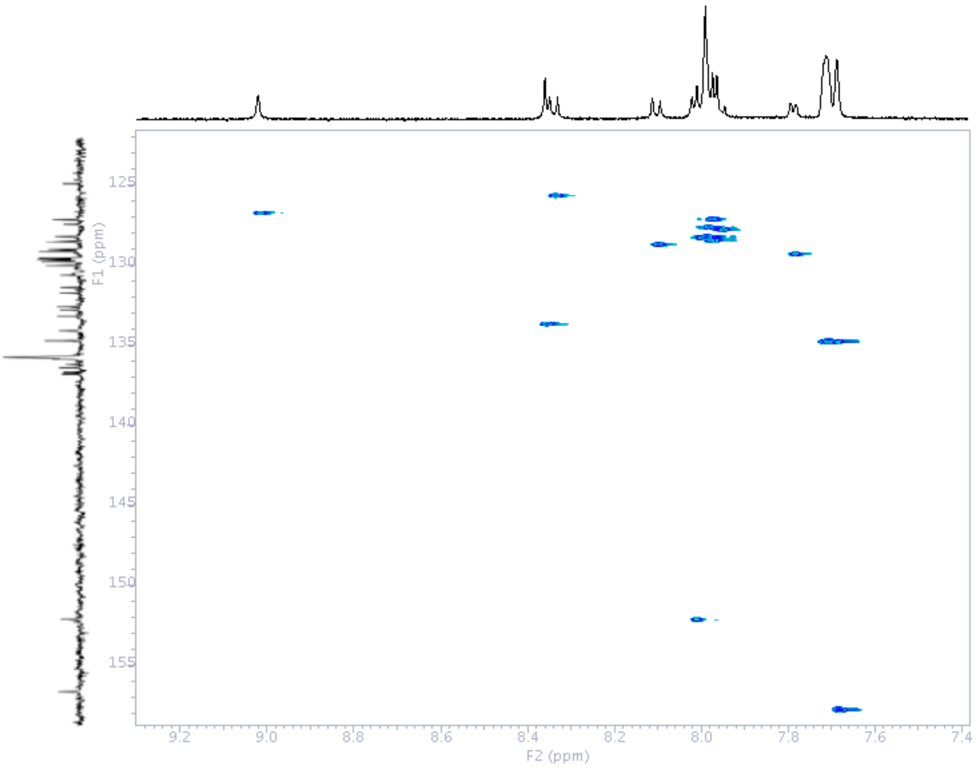

Figure S 131. ^{1}H-^{13}C gHSQCAD-PS (298 K, 500 MHz, Acetonitrile-d_3) spectrum of compound **Ru3C·BAr^{F}$_4$**.

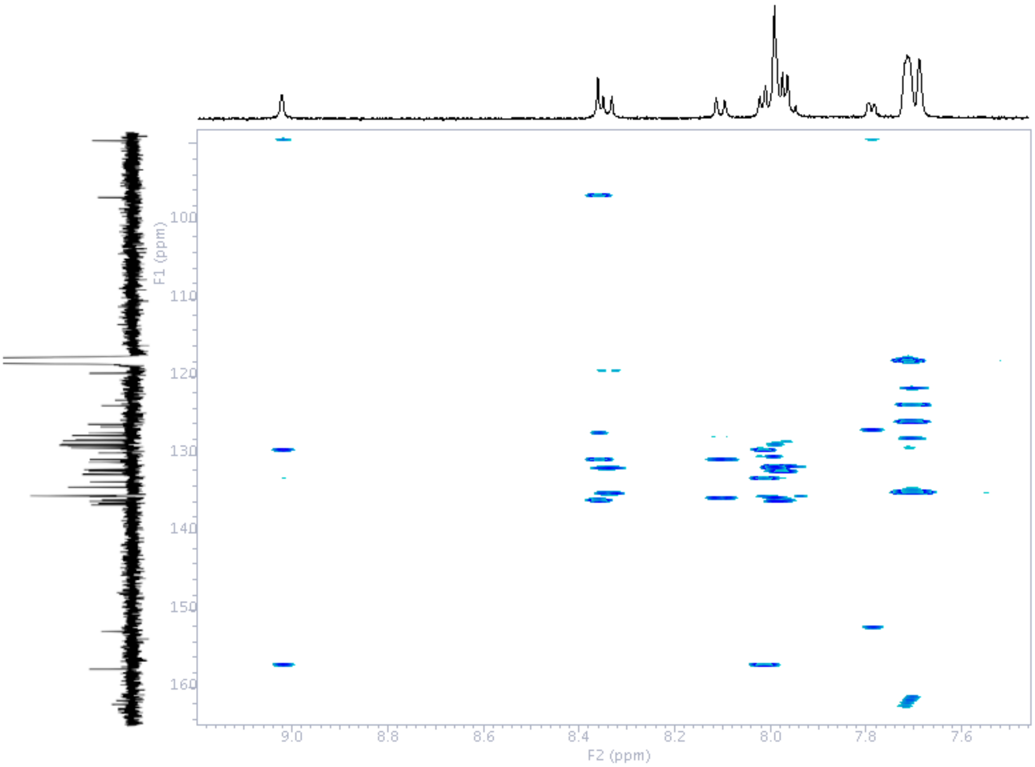

Figure S 132. ^{1}H-^{13}C bsgHMBC (298 K, 500 MHz, Acetonitrile-d_3) spectrum of compound **Ru3C·BAr^{F}$_4$**.

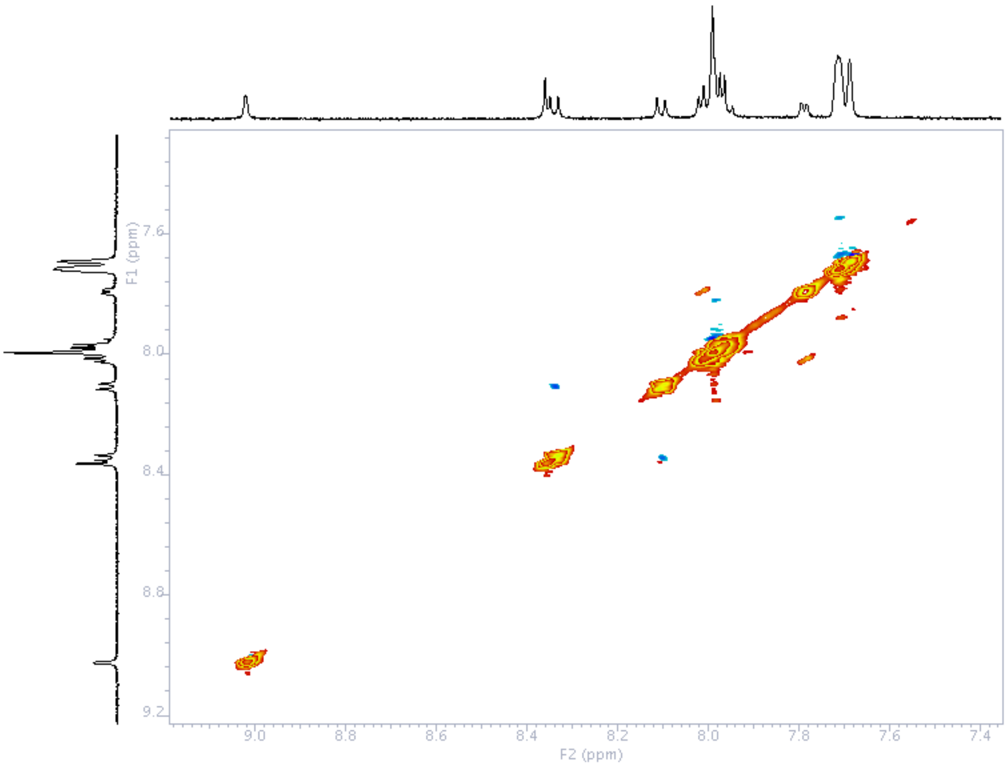

Figure S 133. ^{1}H-^{1}H ROESYAD (298 K, 500 MHz, Acetonitrile-d_3) spectrum of compound **Ru3C·BAr^{F}$_4$**.

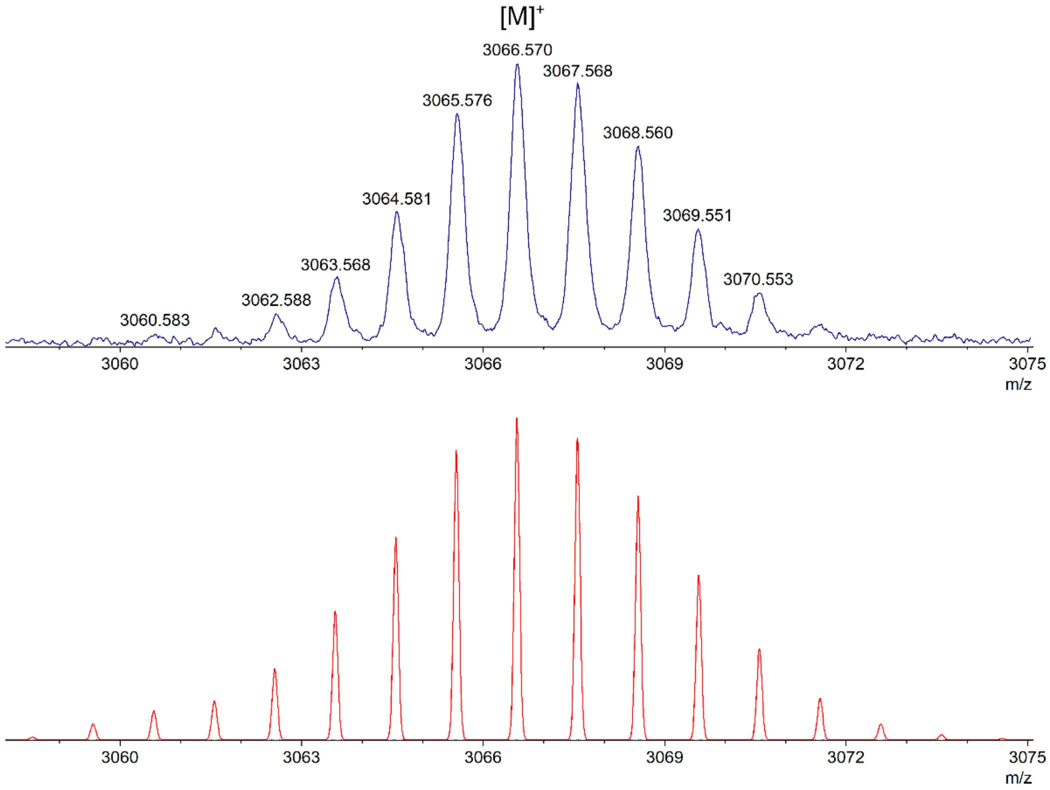

Figure S 134. HRMS (MALDI) of **Ru3C·BAr^F**$_4$, [Ru3C + BAr^F$_4$]$^+$**.** Calculated (red), measured (blue).

UV/Vis Absorption and Fluorescence Studies

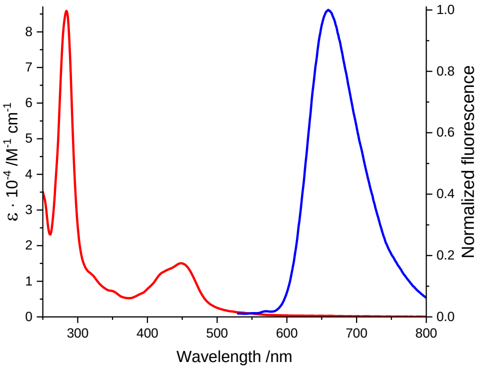

Figure S 135. UV-Vis absorption (red line) and luminescence (blue line) spectra of complex **Ru(bpy)$_3$·BAr^F$_4$** (1 × 10^{-5} M, λ_{ex} = 450 nm) in MeCN.

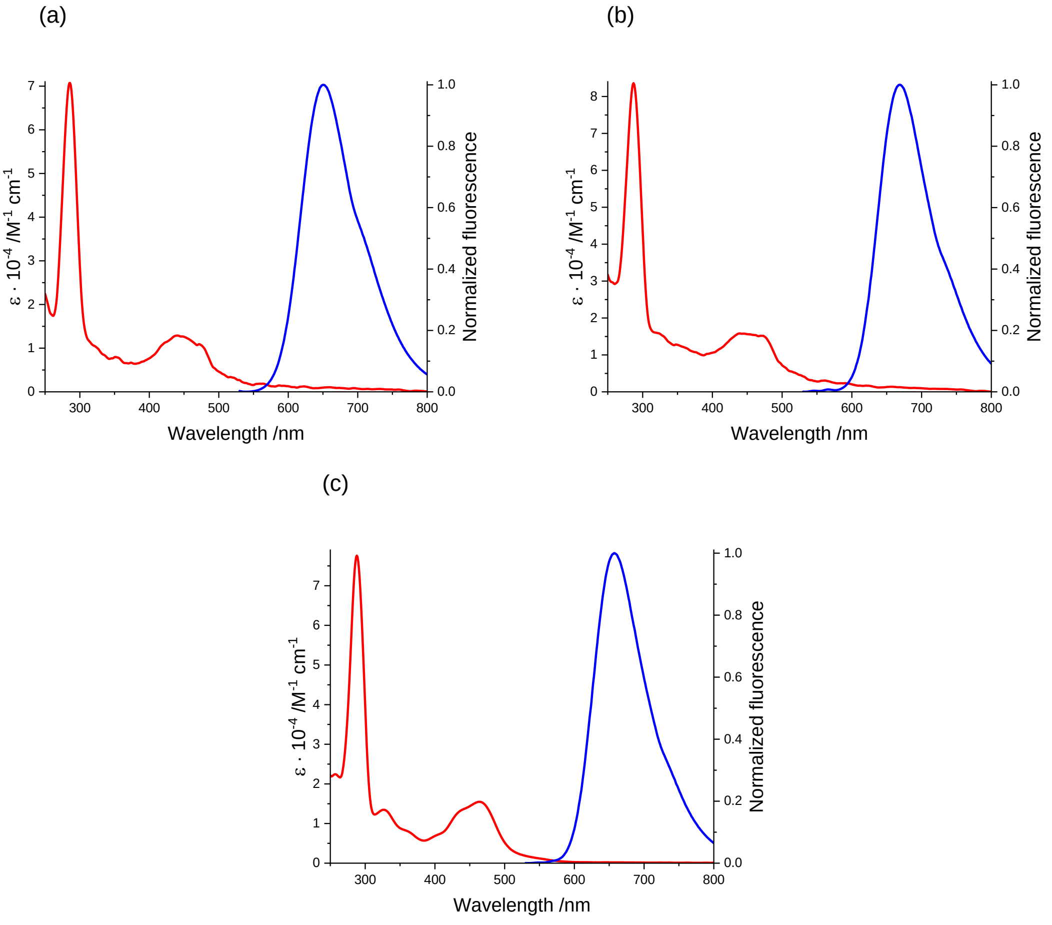

Figure S 136. UV-Vis absorption (red line) and luminescence (blue line) spectra of complexes (a) **Ru1Br·PF$_6$** (1 × 10^{-5} M, λ_{ex} = 449 nm), (b) **Ru2Br·PF$_6$** (1 × 10^{-5} M, λ_{ex} = 463 nm) and (c) **Ru3Br·PF$_6$** (1 × 10^{-5} M, λ_{ex} = 465 nm) in MeCN.

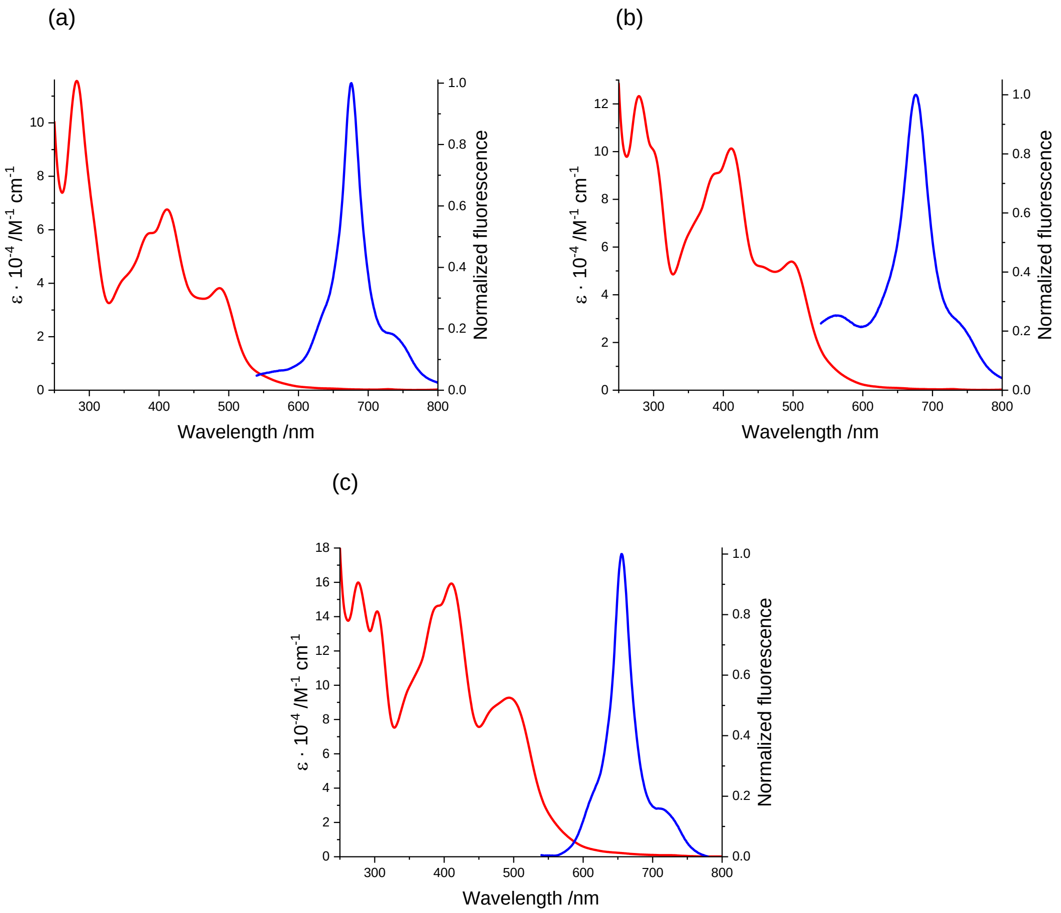

Figure S 137. UV-Vis absorption (red line) and luminescence (blue line) spectra of complexes (a) **Ru1P·BAr^{F}$_{4}$** (1 × 10^{-5} M, λ_{ex} = 509 nm), (b) **Ru2P·BAr^{F}$_{4}$** (1 × 10^{-5} M, λ_{ex} = 488 nm) and (c) **Ru3P·BAr^{F}$_{4}$** (1 × 10^{-5} M, λ_{ex} = 523 nm) in MeCN. The sharp component has been assigned to ligand $S_0 \rightarrow S_1$ transition whereas the broad shoulder to the emissive ^{3}MLCT state.

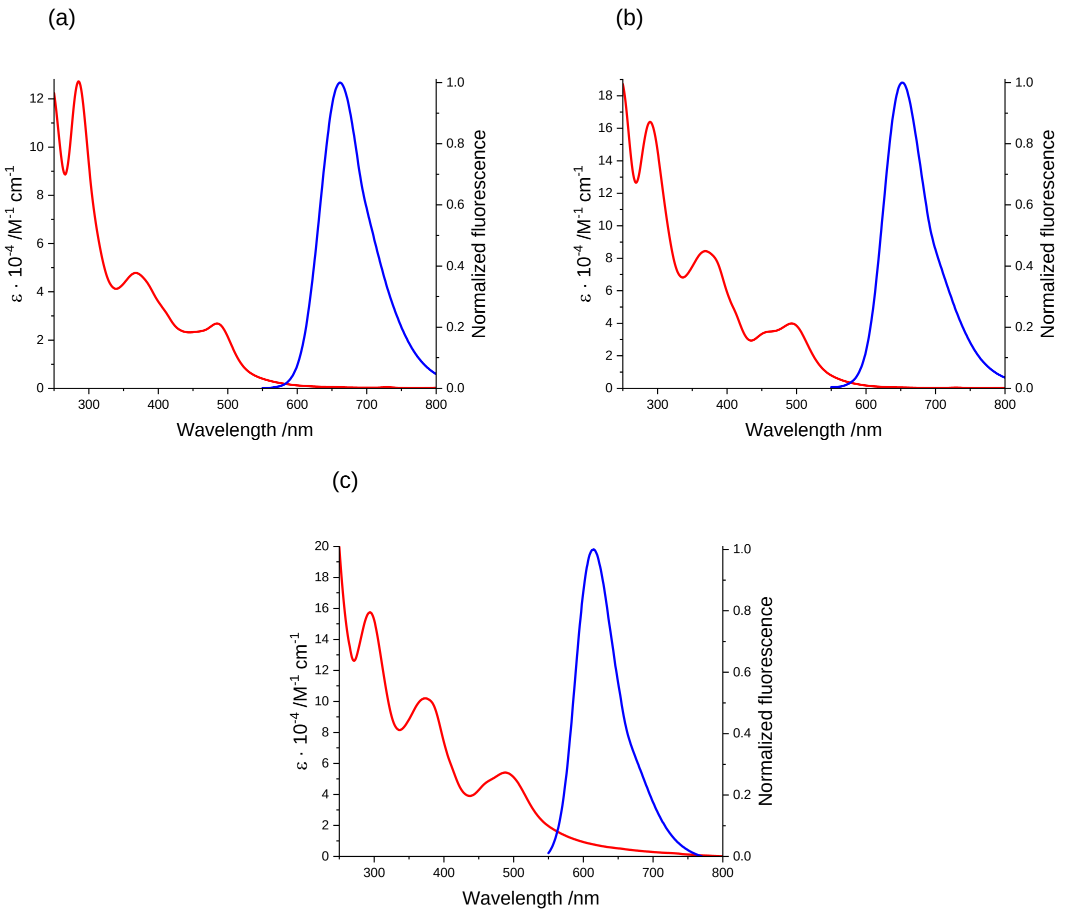

Figure S 138. UV-Vis absorption (red line) and luminescence (blue line) spectra of complexes (a) **Ru1C·BAr$^{F}_{4}$** (1 × 10^{-5} M, λ_{ex} = 488 nm), (b) **Ru2C·BAr$^{F}_{4}$** (1 × 10^{-5} M, λ_{ex} = 499 nm) and (c) **Ru3C·BAr$^{F}_{4}$** (1 × 10^{-5} M, λ_{ex} = 487 nm) in MeCN.

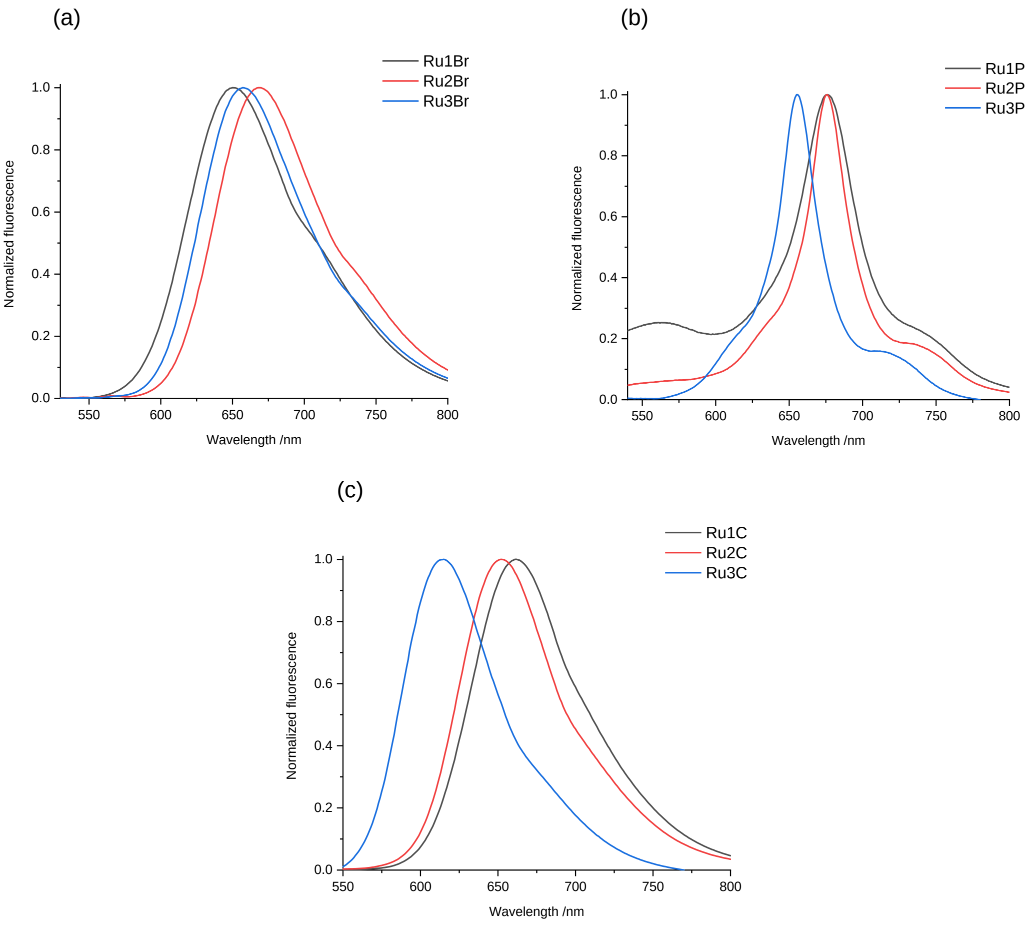

Figure S 139. Normalized fluorescence spectra comparative between complexes (a) **Ru*n*Br·PF_6**, (b) **Ru*n*P·BAr^F_4** and (c) **Ru*n*C·BAr^F_4** in MeCN solution (1×10^{-5} M).

Table S 2. Fluorescence decay lifetimes and quantum yields for the compounds prepared in this work in acetonitrile solution (1×10^{-5} M) measured at the emission maxima. [a]Measured under deareated conditions.

**Compound**	**Φ (%)**	**τ /ns**	**χ^2**
Ru1P·BAr^F_4	0.40	1534	1.07
Ru2P·BAr^F_4	0.28	159	0.98
Ru3P·BAr^F_4	0.22	162	1.02
Ru1C·BAr^F_4	5.3/8.7[a]	326/1108[a]	1.03/1.06[a]
Ru2C·BAr^F_4	1.8/2.6[a]	342/776[a]	1.03/1.11[a]
Ru3C·BAr^F_4	9.5/31[a]	360/1556[a]	1.04/0.99[a]

X-ray Crystallographic Tables

cis-[Ru(Br-bpy)$_2$Cl$_2$]

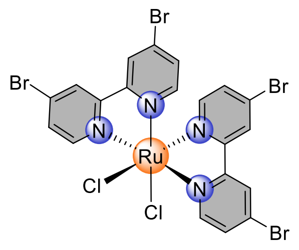

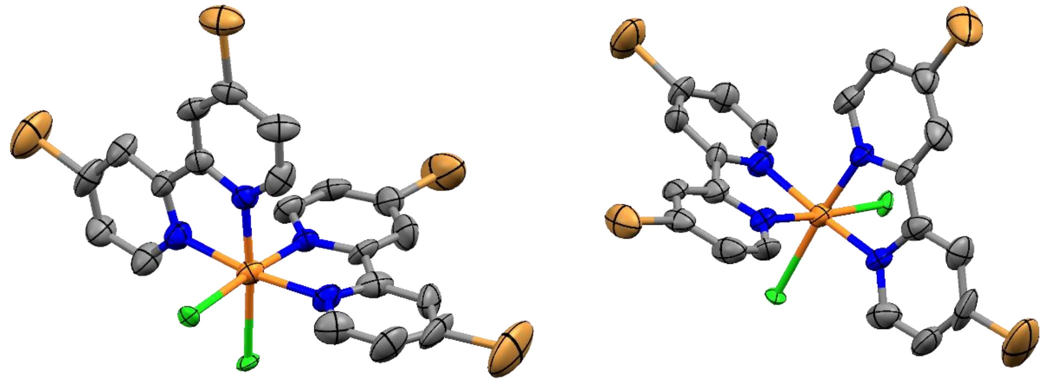

Figure S 140. Schematic representation and crystal structure of compound ***cis*-[Ru(Br-bpy)$_2$Cl$_2$]** (50% ellipsoid contour probability). Solvent and hydrogen atoms were omitted for clarity. Crystallization conditions: layer diffusion recrystallisation from acetonitrile/ether.

Ru1Br·PF$_6$

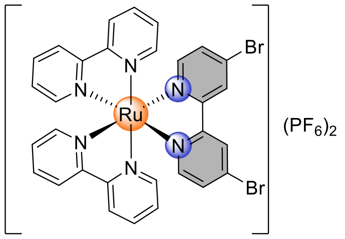

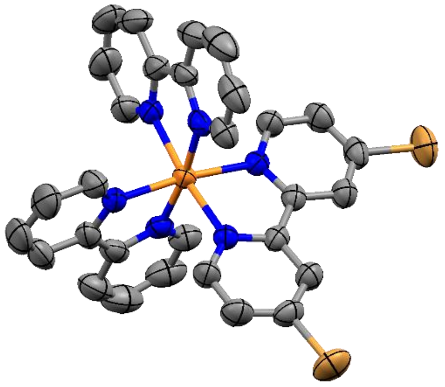

Figure S 141. Schematic representation and crystal structure of compound **Ru1Br·PF$_6$** (50% ellipsoid contour probability). Counterions and hydrogen atoms were omitted for clarity. Crystallization conditions: layer diffusion recrystallisation from acetonitrile/ether. The structure of the cation is equal to one already reported elsewhere[13] but differs in the location of the anions within the unit cell.

Ru(bpy)$_3$·BAr^F

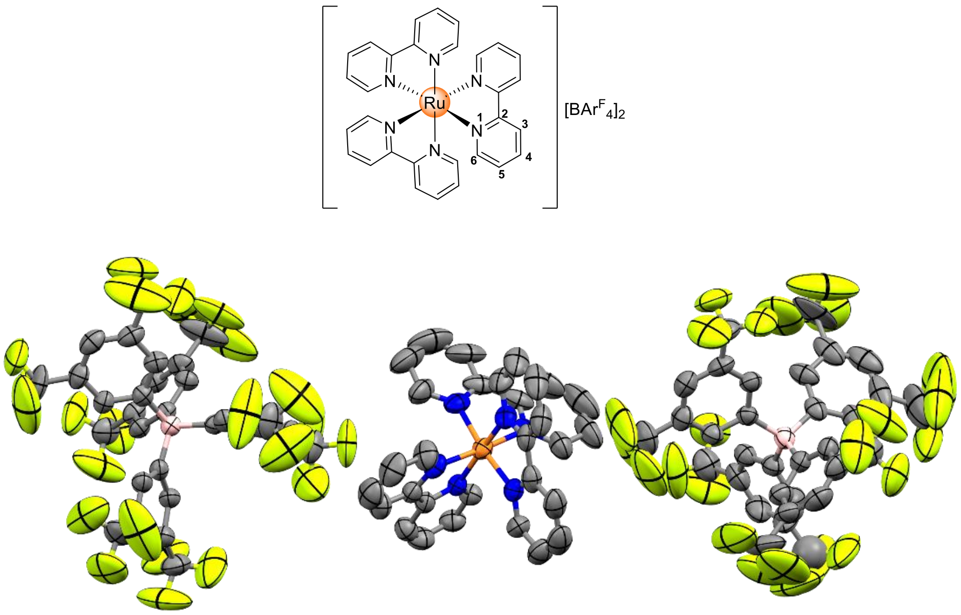

Figure S 142. Schematic representation and crystal structure of compound **Ru(bpy)$_3$·BAr^F$_4$**. The data were not good enough for publication but allowed us to know the atoms' connectivity.

Table S 3. Crystallographic data of compound ***cis*-[Ru(Br-bpy)$_2$Cl$_2$]**.

Identification code	*cis*-[Ru(Br-bpy)$_2$Cl$_2$]
CCDC Number	2392735
Empirical formula	$C_{24}H_{18}Br_4Cl_2N_6Ru$
Formula weight	882.05
Temperature/K	298
Crystal system	triclinic
Space group	P-1
a/Å	10.8940(9)
b/Å	11.7478(12)
c/Å	12.2831(12)
α/°	79.015(9)
β/°	75.269(8)
γ/°	89.523(8)
Volume/Å^3	1491.1(3)
Z	2
ρ_{calc}g/cm^3	1.965
μ/mm^{-1}	6.089
F(000)	844.0
Crystal size/mm^3	0.567 × 0.237 × 0.223
Radiation	MoKα (λ = 0.71073)
2Θ range for data collection/°	4.462 to 49.412
Index ranges	-12 ≤ h ≤ 12, -13 ≤ k ≤ 13, -12 ≤ l ≤ 14
Reflections collected	9274
Independent reflections	5072 [R_{int} = 0.0471, R_{sigma} = 0.0879]
Data/restraints/parameters	5072/0/311
Goodness-of-fit on F^2	1.282
Final R indexes [I>=2σ (I)]	R_1 = 0.1008, wR_2 = 0.3150
Final R indexes [all data]	R_1 = 0.1295, wR_2 = 0.3416
Largest diff. peak/hole / e Å^{-3}	3.96/-1.15

Table S 4. Crystallographic data of compound **Ru1Br·PF₆**.

Identification code	Ru1Br·PF_6
CCDC number	2392734
Empirical formula	$C_{30}H_{22}Br_2F_{13}N_6P_2Ru$
Formula weight	1036.36
Temperature/K	298
Crystal system	monoclinic
Space group	C2/c
a/Å	11.8166(5)
b/Å	27.9121(9)
c/Å	12.9079(5)
α/°	90
β/°	105.155(4)
γ/°	90
Volume/Å^3	4109.3(3)
Z	4
ρ_{calc}g/cm^3	1.675
μ/mm^{-1}	2.492
F(000)	2020.0
Crystal size/mm^3	0.228 × 0.14 × 0.122
Radiation	MoKα (λ = 0.71073)
2Θ range for data collection/°	6.692 to 59.008
Index ranges	-16 ≤ h ≤ 16, -35 ≤ k ≤ 27, -17 ≤ l ≤ 13
Reflections collected	11781
Independent reflections	4810 [R_{int} = 0.0268, R_{sigma} = 0.0383]
Data/restraints/parameters	4810/0/246
Goodness-of-fit on F^2	1.042
Final R indexes [I>=2σ (I)]	R_1 = 0.0546, wR_2 = 0.1398
Final R indexes [all data]	R_1 = 0.0789, wR_2 = 0.1543
Largest diff. peak/hole / e Å^{-3}	0.77/-0.67

Association constants measurements

To estimate the association constants (K_a) of the different **Ru*n*C·BAr^F$_4$** complexes with fullerenes C_{60} and C_{70}, the following method was employed: a solution of each compound (1×10^{-4} — 5×10^{-5} M) in deuterated toluene (tol-d_8) was prepared, and a known volume (0.5 mL) was transferred to an NMR tube covered with a septum. The titration was carried out by adding known portions of a stock solution of the fullerene (1×10^{-3} M) in tol-d_8 to cover a wide range of equivalents. A ^{1}H-NMR experiment was conducted at room temperature (298 K) after each addition. Once all data had been obtained, the changes in chemical shifts ($\Delta\delta$) of selected protons were plotted as a function of the molar fraction of the guest, and the resulting curve was fitted by a nonlinear method using the global analysis approach according to the following equations assuming different equilibria.

1 to 1 equilibrium

$$K_a = \frac{[HG]}{[H][G]} \qquad \text{eq. 1}$$

The change in the chemical shift upon NMR titration is expressed as:

$$\Delta\delta = \Delta\delta_{max}\left(\frac{[HG]}{[H]_0}\right) \qquad \text{eq. 2}$$

Where [HG] is the concentration of the guest of the complex, and is calculated using the following equation:

$$[HG] = \frac{1}{2}\left([G]_0 + [H]_0 + \frac{1}{K_a}\right) - \sqrt{\left([G]_0 + [H]_0 + \frac{1}{K_a}\right)^2 + 4[G]_0[H]_0} \qquad \text{eq. 3}$$

Where:

$[G]_0$ is the total concentration of the guest (C_{60} or C_{70}).

$[H]_0$ is the total concentration of the host (**Ru1C·BAr^F$_4$**, **Ru2C·BAr^F$_4$** or **Ru3C·BAr^F$_4$**).

$\Delta\delta_{max}$ is $\Delta\delta$ at maximum complexation (100% supramolecular complex formation).

K_a is the estimated association constant for 1:1 equilibrium.

1 to 2 equilibrium

$$K_1 = \frac{[HG]}{[H][G]} \qquad \text{eq. 4}$$

$$K_2 = \frac{[HG_2]}{[HG][G]} \qquad \text{eq. 5}$$

$$\beta = K_1K_2 = \frac{[HG_2]}{[H][G]^2} \qquad \text{eq. 6}$$

Changes in chemical shifts upon ^{1}H NMR titration are expressed:

$$\Delta\delta = \frac{\delta_{\Delta HG}K_1[G] + \delta_{\Delta HG_2}K_1K_2[G]^2}{1 + K_1[G] + K_1K_2[G]^2} \qquad \text{eq. 7}$$

Where [G] is the concentration of free guest, and is calculated by the following cubic equation:

$$K_1K_2[G]^3 + K_1\big(1 + K_2(2[H]_0 - [G]_0)\big)[G]^2 + \big(1 + K_1([H]_0 - [G]_0)\big)[G] - [G]_0 = 0 \qquad \text{eq. 8}$$

Where:

$[G]_0$ is the total concentration of the guest (C_{60} or C_{70}).

$[H]_0$ is the total concentration of the host (**Ru2C·BAr^F$_4$**, **Ru3C·BAr^F$_4$**).

$\Delta\delta_{\Delta HG}$ is the $\Delta\delta$ at maximum complexation of the first equilibrium [*HG*].

$\Delta\delta_{\Delta HG2}$ is the $\Delta\delta$ at maximum complexation of the second equilibrium [HG_2].

K_1 is the estimated association constant for the first equilibrium.

K_2 is the estimated association constant for the second equilibrium.

In each case, $\Delta\delta_{max}$ and K_a for a 1:1 equilibrium, $\Delta\delta_{\Delta HG}$, $\Delta\delta_{\Delta HG2}$, K_1 and K_2 for a 1:2 equilibrium were obtained by using the non-linear fitting tool provided by Bindfit v0.5 (http://www.supramolecular.org), according to different binding models (flavours).[14,15] Links to all the fittings of the data are provided below.

The first model is the stepwise (non-degenerate) full 1:2 binding model. This assumes that each host has two non-identical binding sites that allows for cooperativity. In this model all the parameters in equation 7 are evaluated independently.

The second model considered is the stepwise (non-degenerate), additive 1:2 binding model. To reduce the number of parameters to fit in equation 7 the assumption that the proportionality constants of the 1:1 and 1:2 adducts are additive is made. This means that the change in the chemical shifts of the signals from the free host to the 1:1 adduct is 1/2 of the change between the 1:2 adduct and the free host. Then it follows that $\delta_{\Delta HG}$ = 1/2 $\delta_{\Delta HG2}$ and eq. 7 can be simplified to eq. 9, which has three parameters to fit ($\delta_{\Delta HG}$, K_1, and K_2).

$$\Delta\delta = \frac{\delta_{\Delta HG}\left(K_1[G]+2K_1K_2[G]^2\right)}{1+K_1[G]+K_1K_2[G]^2} \qquad \text{eq. 9}$$

The third model is the stepwise non-cooperative 1:2 binding model. In this case, the assumption that the 1:2 binding is non-cooperative is made. So, after considering statistical factors, it is stablished that $K_1 = 4K_2$. Defining $K_{1n} = K_1 = 4K_2$, equation 7 can be simplified to equation 10, which also has three parameters to fit ($\delta_{\Delta HG}$, $\delta_{\Delta HG2}$ and K_{1n})

$$\Delta\delta = \frac{\delta_{\Delta HG}K_{1n}[G]+\delta_{\Delta HG_2}K_{1n}^2[G]^2}{1+K_{1n}[G]+K_{1n}^2[G]^2} \qquad \text{eq. 10}$$

The last model is the statistical 1:2 model. In this model, both assumptions are made, that the binding is non-cooperative ($K_1 = 4K_2$) and that the chemical shifts are all additive ($\delta_{\Delta HG}$ = 1/2 $\delta_{\Delta HG2}$). Now equation 7 is simplified to equation 11, which only has two parameters to fit ($\delta_{\Delta HG}$ and K_{1n}).

$$\Delta\delta = \frac{\delta_{\Delta HG}\left(K_{1n}[G]+2K_{1n}^2[G]^2\right)}{1+K_{1n}[G]+K_{1n}^2[G]^2} \qquad \text{eq. 11}$$

Cooperativity[16] is described by α parameter, which states that macroscopic association constants are related, after accounting for statistical factors, according to equation 12.

$$\alpha = \frac{4K_2}{K_1} \qquad \text{eq. 12}$$

Where:
$\alpha > 1$ means positive cooperativity
$\alpha < 1$ means negative cooperativity
$\alpha = 1$ stands for no cooperativity

1 to 3 equilibrium

$$K_1 = \frac{[HG]}{[H][G]} \qquad \text{eq. 13}$$

$$K_2 = \frac{[HG_2]}{[HG][G]} \qquad \text{eq. 14}$$

$$K_3 = \frac{[HG_3]}{[HG_2][G]} \qquad \text{eq. 15}$$

$$\beta = K_1K_2K_3 = \frac{[HG_3]}{[H][G]^3} \qquad \text{eq. 16}$$

Changes in chemical shifts upon ^{1}H NMR titration are expressed:

$$\Delta\delta = \frac{\delta_{\Delta HG}K_1[G]+\delta_{\Delta HG_2}K_1K_2[G]^2+\delta_{\Delta HG_3}K_1K_2K_3[G]^3}{1+K_1[G]+K_1K_2[G]^2+K_1K_2K_3[G]^3} \quad \text{eq. 17}$$

Where [*G*] is the concentration of free guest, and is calculated by the following quartic equation:

$$K_1K_2K_3[G]^4 + K_1K_2\big(1 + K_3(3[H]_0 - [G]_0)\big)[G]^3 + K_1\big(1 + K_2(2[H]_0 - [G]_0)\big)[G]^2 +$$
$$\big(1 + K_1([H]_0 - [G]_0)\big)[G] - [G]_0 = 0 \quad \text{eq. 18}$$

$[G]_0$ is the total concentration of the guest (C_{60} or C_{70}).

$[H]_0$ is the total concentration of the host (**Ru3C·BAr^F$_4$**).

$\Delta\delta_{\Delta HG}$ is the $\Delta\delta$ at maximum complexation of the first equilibrium [*HG*].

$\Delta\delta_{\Delta HG2}$ is the $\Delta\delta$ at maximum complexation of the second equilibrium [HG_2].

$\Delta\delta_{\Delta HG3}$ is the $\Delta\delta$ at maximum complexation of the third equilibrium [HG_3].

K_1 is the estimated association constant for the first equilibrium.

K_2 is the estimated association constant for the second equilibrium.

K_3 is the estimated association constant for the third equilibrium.

In this case, the data was fitted using Matlab, using a script provided here.[17] The analysis has been done considering different binding models as previously explained for the 1:2 equilibrium.[14,15,17] The first one is the stepwise (non-degenerate) full 1:3 binding model. In this model all the parameters in equation 17 are evaluated independently.

The second model considered is the stepwise (non-degenerate), additive 1:3 binding model. In this case is considered that $\delta_{\Delta HG}$ = 1/2 $\delta_{\Delta HG2}$ = 1/3 $\delta_{\Delta HG3}$ and eq. 17 can be simplified to eq. 19, which has four parameters to fit ($\delta_{\Delta HG}$, K_1, K_2 and K_3)

$$\Delta\delta = \frac{\delta_{\Delta HG}\left(K_1[G]+2K_1K_2[G]^2+3K_1K_2K_3[G]^3\right)}{1+K_1[G]+K_1K_2[G]^2+K_1K_2K_3[G]^3} \quad \text{eq. 19}$$

The third model is the stepwise non-cooperative 1:3 binding model. In this case, it is stablished that $K_1 = 3K_2 = 9K_3$. Defining $K_{1n} = K_1 = 3K_2 = 9K_3$, equation 17 can be simplified to equation 20, which also has four parameters to fit ($\delta_{\Delta HG}$, $\delta_{\Delta HG2}$, $\delta_{\Delta HG3}$ and K_{1n})

$$\Delta\delta = \frac{\delta_{\Delta HG}K_{1n}[G]+\delta_{\Delta HG_2}K_{1n}^2[G]^2+\delta_{\Delta HG_3}K_{1n}^3[G]^3}{1+K_{1n}[G]+K_{1n}^2[G]^2+K_{1n}^3[G]^3} \quad \text{eq. 20}$$

The last model is the statistical 1:3 model. In this model, both assumptions are made, following that $K_1 = 3K_2 = 9K_3$ and $\delta_{\Delta HG}$ = 1/2 $\delta_{\Delta HG2}$ = 1/3 $\delta_{\Delta HG3}$. Now equation 17 is simplified to equation 21, which only has two parameters to fit ($\delta_{\Delta HG}$ and K_{1n}).

$$\Delta\delta = \frac{\delta_{\Delta HG}\left(K_{1n}[G]+2K_{1n}^2[G]^2+3K_{1n}^3[G]^3\right)}{1+K_{1n}[G]+K_{1n}^2[G]^2+K_{1n}^3[G]^3} \quad \text{eq. 21}$$

Similarly, cooperativity[16] is described by α_1 and α_2 parameters, which account for the second and third binding steps, respectively. Again, after considering statistical factors, both parameters relate association constants as follows:

$$\alpha_1 = \frac{3K_2}{K_1} \quad \text{eq. 22}$$

$$\alpha_2 = \frac{3K_3}{K_2} \quad \text{eq. 23}$$

Therefore, a global cooperativity factor can be described.

$$\alpha = \alpha_1 \cdot \alpha_2 = \frac{9K_3}{K_1} \quad \text{eq. 24}$$

Ru1C·BAr$^F_4$ *vs.* C_{60}

1 to 1

http://app.supramolecular.org/bindfit/view/87be75cb-e954-4d78-bb70-1fce034861ea

Ru1C·BAr$^F_4$ *vs.* C_{70}

1 to 1

http://app.supramolecular.org/bindfit/view/cdca1ba7-493c-472b-8e4e-2e465e1d2858

Table S 5. Association constants (M^{-1}) of host **Ru1C·BAr$^F_4$** with fullerenes according to a 1 to 1 model.

Guest	K_a	SSR	SE_y
C_{60}	354	$6.14 \cdot 10^{-6}$	$5.54 \cdot 10^{-4}$
C_{70}	569	$1.64 \cdot 10^{-5}$	$5.15 \cdot 10^{-4}$

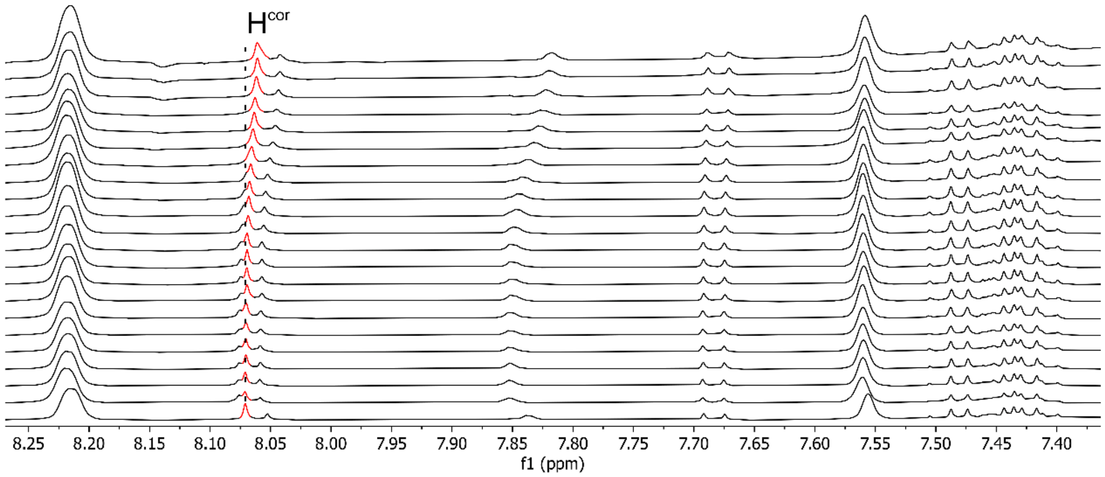

Figure S 143. Stacked ^{1}H-NMR (500 MHz) spectra for the titration of **Ru1C·BAr$^F_4$** with variable concentrations of C_{60} in toluene-d_8 at 298 K. The most significant shift of the corannulene unit (H^{cor}) has been labelled.

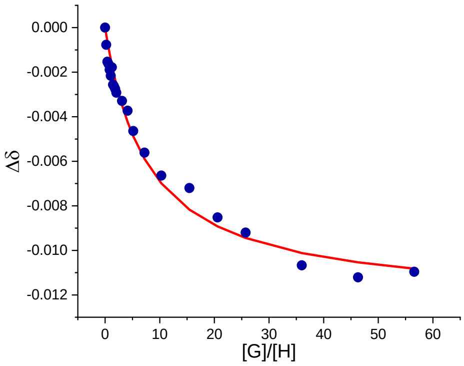

Figure S 144. Non-linear regression for $\Delta\delta$ of selected proton (H^{cor}) for the titration of complex **Ru1C·BAr$^F_4$** with C_{60}.

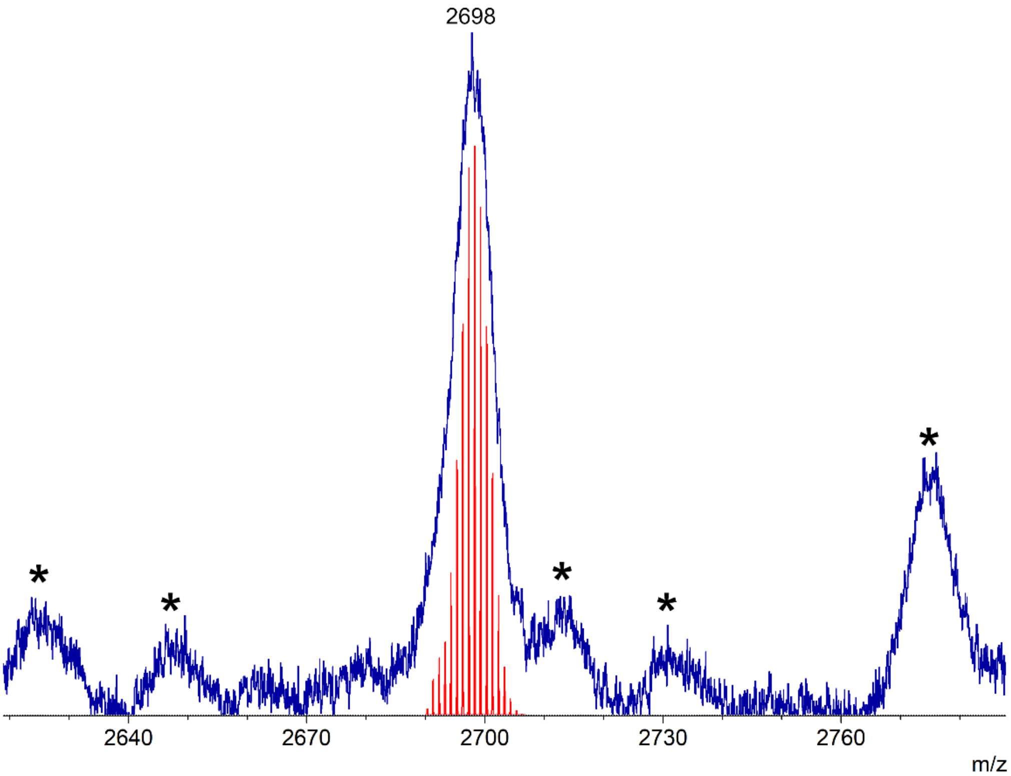

Figure S 145. Detection of adduct **C_{60}@Ru1C·BAr^F_4** in LRMS (MALDI-TOF, linear detection, positive mode). Calculated (red), measured (blue). *Peaks could not be assigned.

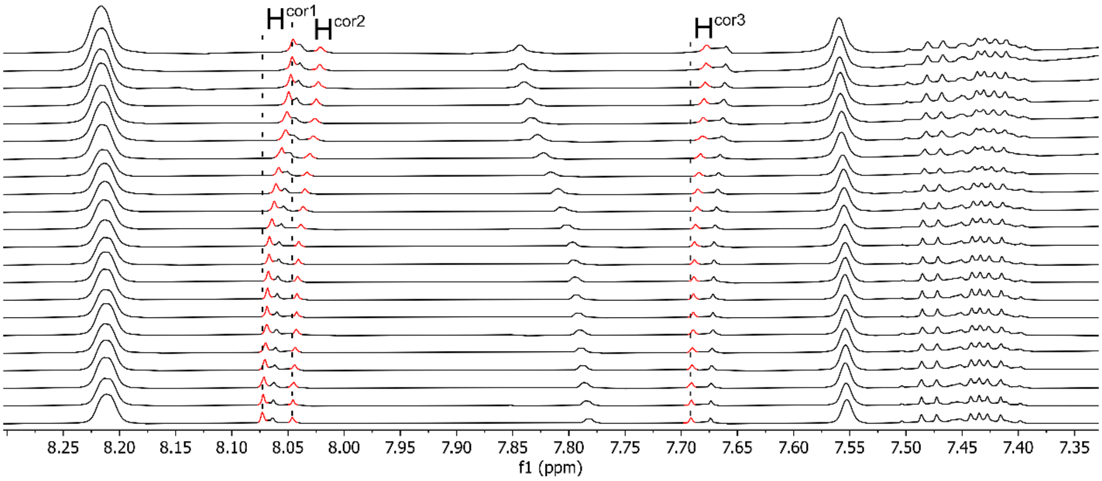

Figure S 146. Stacked ^{1}H-NMR (500 MHz) spectra for the titration of **Ru1C·BAr^F_4** with variable concentrations of C_{70} in toluene-d_8 at 298 K. The most significant shifts of the corannulene unit (H^{cor1}, H^{cor2} and H^{cor3}) have been labelled.

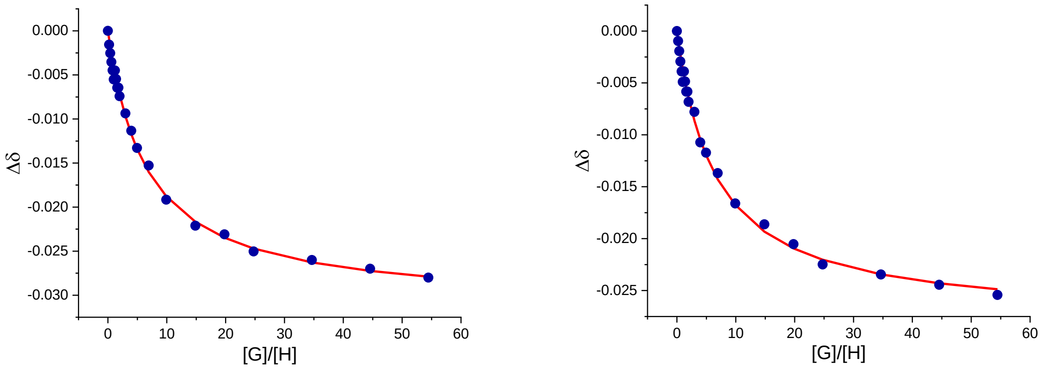

Figure S 147. Non-linear regression for Δδ of selected protons (left plot: H^{cor1}, right plot: H^{cor2}) for the titration of complex **Ru1C·BAr^F$_4$** with C_{70}.

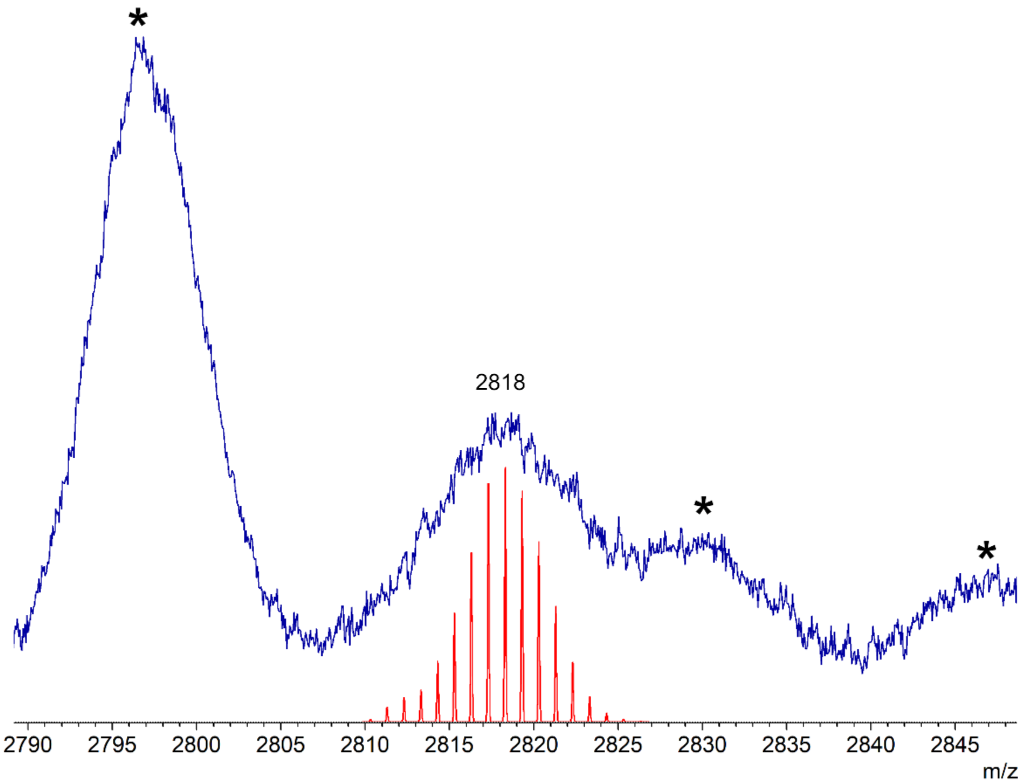

Figure S 148. Detection of adduct **C_{70}@Ru1C·BAr^F$_4$** in LRMS (MALDI-TOF, linear detection, positive mode). Calculated (red), measured (blue). *Peaks could not be assigned.

<u>Ru2C·BAr^F$_4$ *vs.* C_{60}</u>

1 to 1

http://app.supramolecular.org/bindfit/view/4f126ee3-45f3-4a39-b492-4a5e0adaae50

1 to 2 additive

http://app.supramolecular.org/bindfit/view/26f2776e-06e5-4f1e-8329-c4364c1f185d

1 to 2 non-cooperative

http://app.supramolecular.org/bindfit/view/1b0bd544-9174-4ca6-9641-9ecc5e1cdf10

1 to 2 statistical

http://app.supramolecular.org/bindfit/view/71a1d980-d1e1-4e88-92f6-b4ff53de7f07

Table S 6. Association constants (M^{-1}) of host **Ru2C·BAr^F$_4$** with C_{60} according to different binding models. [a]Fit failed.

Model	K_1	K_2	SSR	SE_y	Cov_{fit} ratio
**1 to 1**	506	-	$6.55 \cdot 10^{-6}$	$3.33 \cdot 10^{-4}$	1
**1 to 2 full[a]**	-	-	-	-	-
**1 to 2 additive**	771	97	$6.54 \cdot 10^{-6}$	$3.36 \cdot 10^{-4}$	1.00
**1 to 2 non-coop**	721	180	$6.37 \cdot 10^{-6}$	$3.37 \cdot 10^{-4}$	1.03
**1 to 2 statistical**	1188	297	$6.57 \cdot 10^{-6}$	$3.34 \cdot 10^{-4}$	1.00

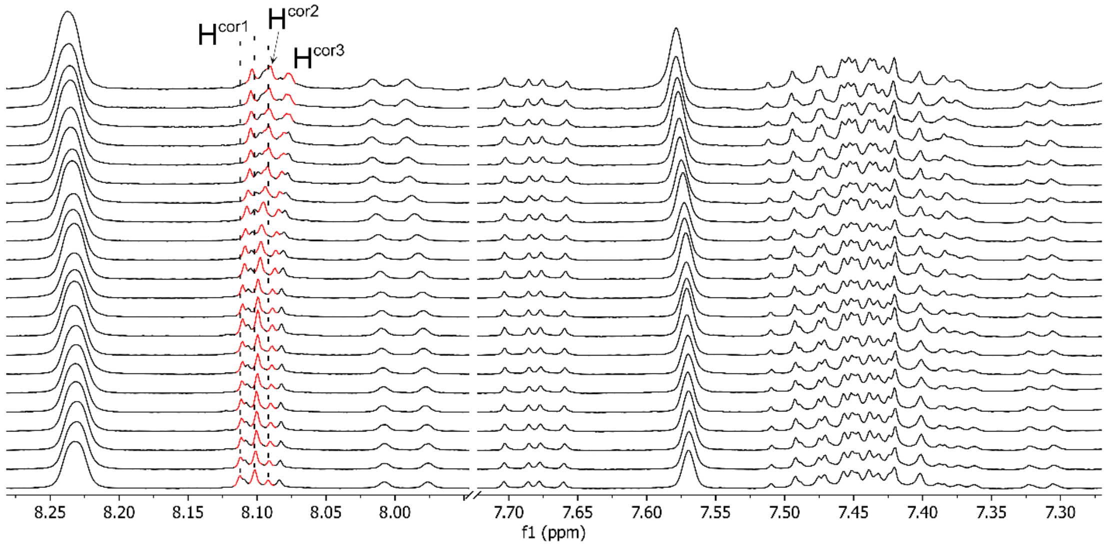

Figure S 149. Stacked ^{1}H-NMR (500 MHz) spectra for the titration of **Ru2C·BAr^F$_4$** with variable concentrations of C_{60} in toluene-d_8 at 298 K. The most significant shifts of the corannulene unit (H^{cor1}, H^{cor2} and H^{cor3}) have been labelled.

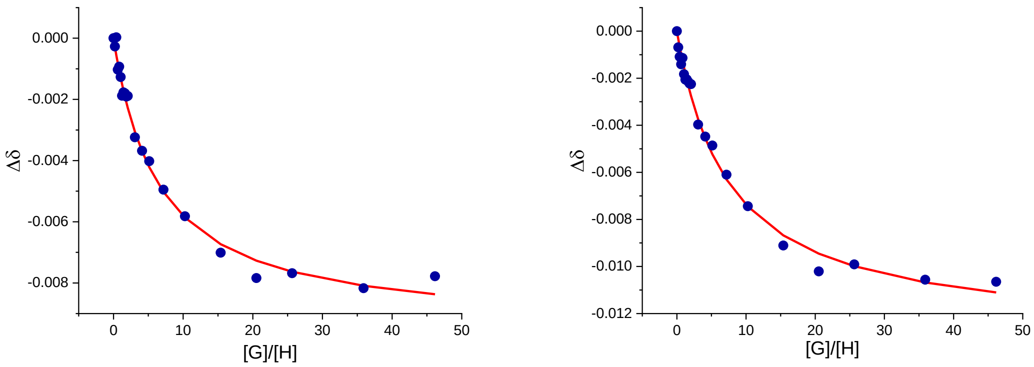

Figure S 150. Non-linear regression for Δδ of selected protons (left plot: H^{cor1}, right plot: H^{cor2}) for the titration of complex **Ru2C·BAr^F$_4$** with C_{60} obtained with the 1:2 non-cooperative binding model.

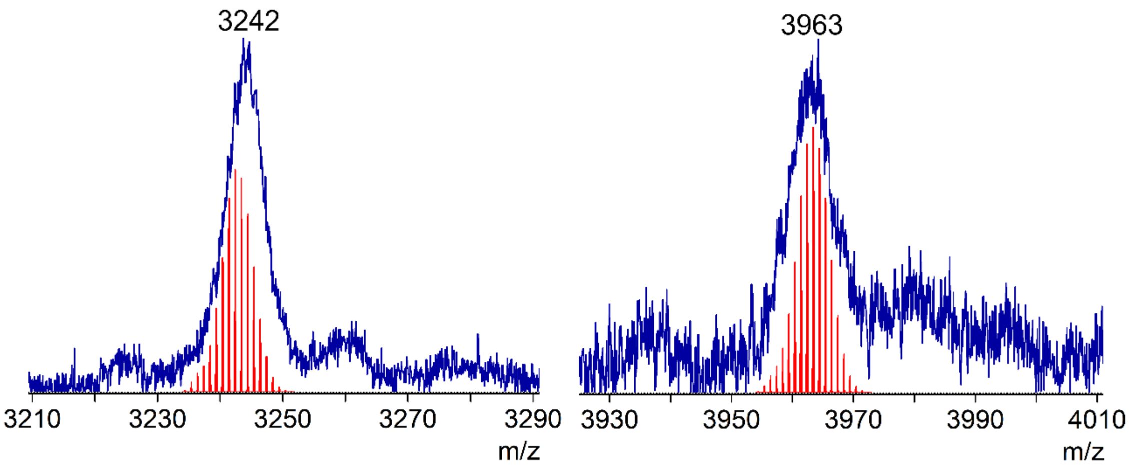

Figure S 151. Detection of adducts **C_{60}@Ru2C·BAr$^F{}_4$** (left) and **$(C_{60})_2$@Ru2C·BAr$^F{}_4$** (right) in LRMS (MALDI-TOF, linear detection, positive mode). Calculated (red), measured (blue).

Ru2C·BAr$^F{}_4$ *vs.* C_{70}

1 to 1

http://app.supramolecular.org/bindfit/view/7ea18d64-413d-48dc-85ec-fdd68265a443

1 to 2 full

http://app.supramolecular.org/bindfit/view/68eb74e0-ad3b-4c4f-9a2d-107e7fdde5fb

1 to 2 additive

http://app.supramolecular.org/bindfit/view/2fe960d6-4023-47e6-9b61-a4c6063e7618

1 to 2 non-cooperative

http://app.supramolecular.org/bindfit/view/1a96e51d-5c9b-47d4-bb47-3af5ba1317b0

1 to 2 statistical

http://app.supramolecular.org/bindfit/view/e795a7e9-f489-42c5-aa32-145e6d055c67

Table S 7. Association constants (M^{-1}) of host **Ru2C·BAr$^F{}_4$** with C_{70} according to different binding models.

Model	K_1	K_2	SSR	SE_y	Cov_{fit} ratio
**1 to 1**	707	-	$4.14 \cdot 10^{-5}$	$7.24 \cdot 10^{-4}$	1
**1 to 2 full**	1981	1623	$1.77 \cdot 10^{-5}$	$4.89 \cdot 10^{-4}$	2.21
**1 to 2 additive**	2384	1498	$2.78 \cdot 10^{-5}$	$5.97 \cdot 10^{-4}$	1.53
**1 to 2 non-coop**	4932	1233	$1.79 \cdot 10^{-5}$	$4.88 \cdot 10^{-4}$	2.18
**1 to 2 statistical**	1710	427	$3.92 \cdot 10^{-5}$	$7.05 \cdot 10^{-4}$	1.06

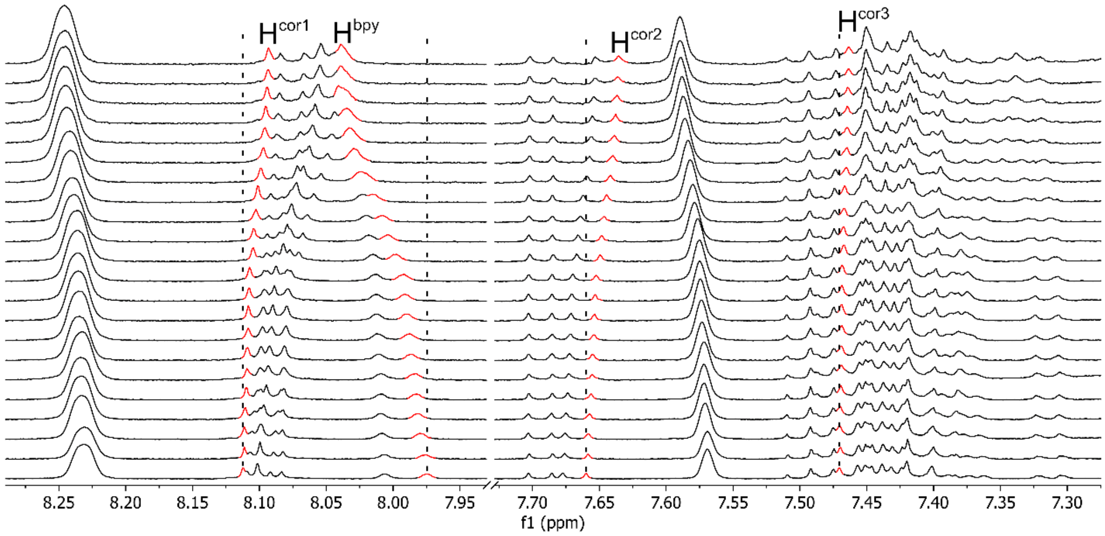

Figure S 152. Stacked ^{1}H-NMR (500 MHz) spectra for the titration of **Ru2C·BAr^F$_4$** with variable concentrations of C_{70} in toluene-d_8 at 298 K. The most significant shifts of the complex (H^{cor1}, H^{bpy}, H^{cor2} and H^{cor3}) have been labelled.

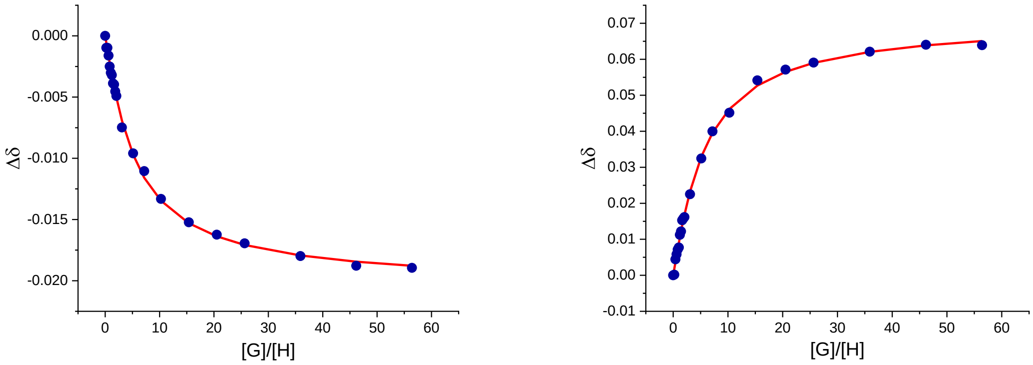

Figure S 153. Non-linear regression for $\Delta\delta$ of selected protons (left plot: H^{cor1}, right plot: H^{bpy}) for the titration of complex **Ru2C·BAr^F$_4$** with C_{70} obtained with the 1:2 non-cooperative binding model.

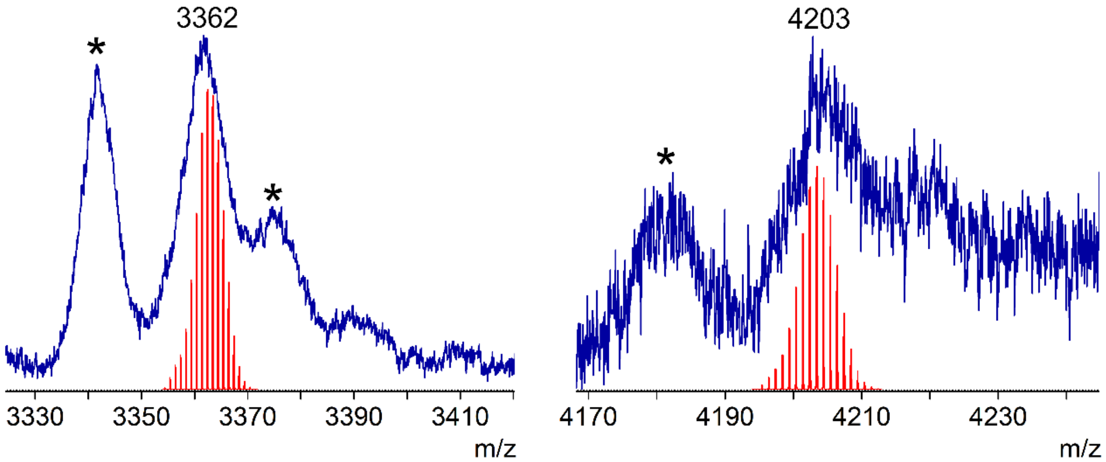

Figure S 154. Detection of adducts **C_{70}@Ru2C·BAr^F$_4$** (left) and **$(C_{70})_2$@Ru2C·BAr^F$_4$** (right) in LRMS (MALDI-TOF, linear detection, positive mode). Calculated (red), measured (blue). *Peaks could not be assigned.

Ru3C·BAr^F$_4$ *vs.* C_{60}

1 to 1

http://app.supramolecular.org/bindfit/view/01e4f8d8-be4a-48ac-b256-9ca82bca55ac

1 to 2 full

http://app.supramolecular.org/bindfit/view/5917f5aa-31a4-4a0a-82cc-a8e385920460

1 to 2 additive

http://app.supramolecular.org/bindfit/view/8841f138-53ae-4f6f-b9de-1c5ea49d8f53

1 to 2 non-cooperative

http://app.supramolecular.org/bindfit/view/567f73de-01d0-4d0e-8a35-b7d9012d2ef9

1 to 2 statistical

http://app.supramolecular.org/bindfit/view/26cae91a-9c79-4869-b7e3-933208a693ac

Table S 8. Association constants (M^{-1}) of host **Ru3C·BAr^F$_4$** with C_{60} according to different binding models.

Model	K_1	K_2	K_3	SSR	SE_y	Cov_{fit} ratio
**1 to 1**	1623	-	-	$1.32 \cdot 10^{-5}$	$4.62 \cdot 10^{-4}$	1
**1 to 2 full**	13700	-297	-	$5.94 \cdot 10^{-5}$	$3.20 \cdot 10^{-4}$	2.15
**1 to 2 additive**	2057	253	-	$1.27 \cdot 10^{-5}$	$4.56 \cdot 10^{-4}$	1.03
**1 to 2 non-coop**	2331	583	-	$1.23 \cdot 10^{-5}$	$4.56 \cdot 10^{-4}$	1.10
**1 to 2 statistical**	2715	679	-	$1.35 \cdot 10^{-5}$	$4.66 \cdot 10^{-4}$	0.99
**1 to 3 full**	$3.82 \cdot 10^{-4}$	$4.75 \cdot 10^{13}$	$6.91 \cdot 10^{-10}$	$5.04 \cdot 10^{-6}$	$3.06 \cdot 10^{-4}$	2.54
**1 to 3 additive**	7491	164	8272	$7.26 \cdot 10^{-6}$	$3.48 \cdot 10^{-4}$	1.76
**1 to 3 non-coop**	5968	1989	663	$5.51 \cdot 10^{-6}$	$3.14 \cdot 10^{-4}$	2.32
**1 to 3 statistical**	4390	1463	488	$1.38 \cdot 10^{-5}$	$4.71 \cdot 10^{-4}$	0.97

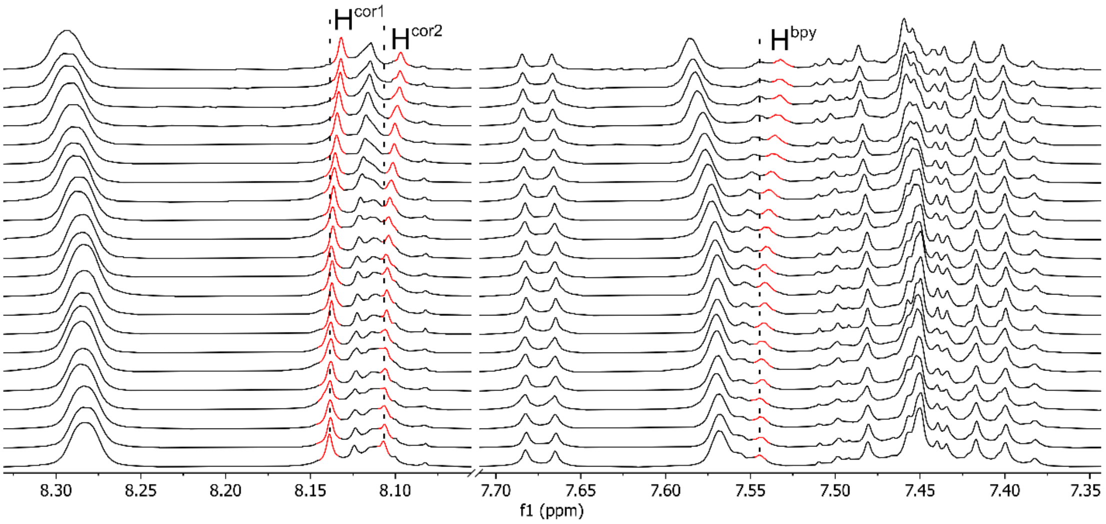

Figure S 155. Stacked ^{1}H-NMR (500 MHz) spectra for the titration of **Ru3C·BAr^F$_4$** with variable concentrations of C_{60} in toluene-d_8 at 298 K. The most significant shifts of the complex (H^{cor1}, H^{cor2} and H^{bpy}) have been labelled.

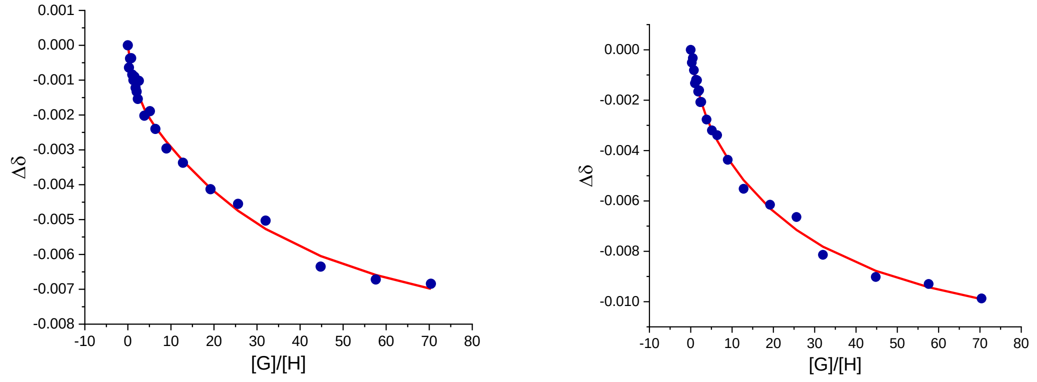

Figure S 156. Non-linear regression for Δδ of selected protons (left plot: H^{cor1}, right plot: H^{cor2}) for the titration of complex **Ru3C·BAr^F$_4$** with C_{60} obtained with the 1:3 non-cooperative binding model.

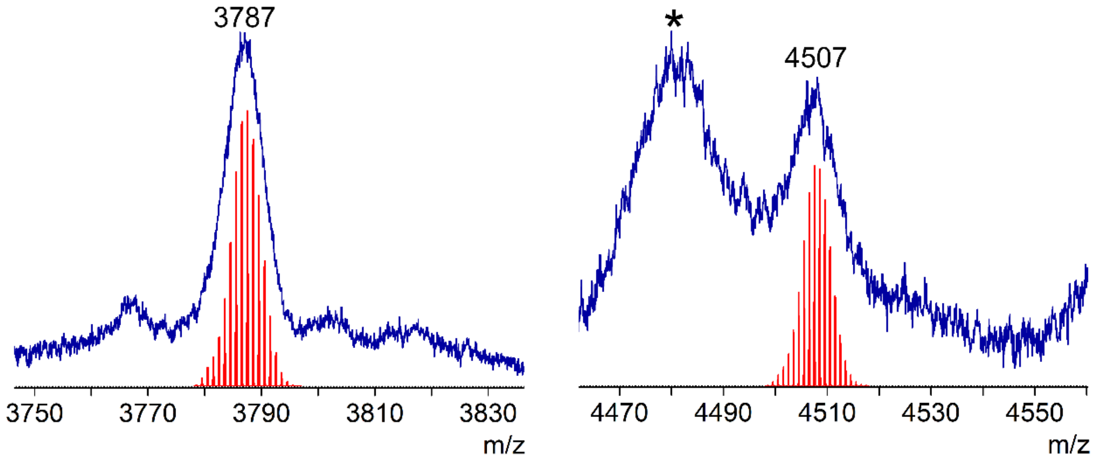

Figure S 157. Detection of adducts **C_{60}@Ru3C·BAr^F$_4$** (left) and **(C_{60})$_2$@Ru3C·BAr^F$_4$** (right) in LRMS (MALDI-TOF, linear detection, positive mode). Calculated (red), measured (blue). *Peaks could not be assigned.

Ru3C·BAr^F$_4$ *vs.* C_{70}

1 to 1

http://app.supramolecular.org/bindfit/view/d8bbac3f-452a-4d1e-b733-f06de1f1d902

1 to 2 full

http://app.supramolecular.org/bindfit/view/c686c077-3e05-4067-aa21-d6066c58bb23

1 to 2 additive

http://app.supramolecular.org/bindfit/view/f408dae9-4200-4c44-a508-a5796032948a

1 to 2 non-cooperative

http://app.supramolecular.org/bindfit/view/e5c01e4f-fbcd-4f31-b22f-d928c142923d

1 to 2 statistical

http://app.supramolecular.org/bindfit/view/542c04ef-9f0d-4de4-b4c6-0c99e3927c05

Table S 9. Association constants (M^{-1}) of host **Ru3C·BAr^F$_4$** with C_{70} according to different binding models.

Model	K_1	K_2	K_3	SSR	SE_y	Cov_{fit} ratio
**1 to 1**	1692	-	-	$4.96 \cdot 10^{-5}$	$7.73 \cdot 10^{-4}$	1
**1 to 2 full**	36920	1205	-	$1.26 \cdot 10^{-5}$	$4.02 \cdot 10^{-4}$	3.63
**1 to 2 additive**	2001	70	-	$4.95 \cdot 10^{-5}$	$7.77 \cdot 10^{-4}$	1.00
**1 to 2 non-coop**	4168	1042	-	$1.59 \cdot 10^{-5}$	$4.48 \cdot 10^{-4}$	2.88
**1 to 2 statistical**	4046	1011	-	$5.05 \cdot 10^{-5}$	$7.80 \cdot 10^{-4}$	0.98
**1 to 3 full**	$1.16 \cdot 10^{9}$	2763	996	$1.07 \cdot 10^{-5}$	$3.84 \cdot 10^{-4}$	4.25
**1 to 3 additive**	3464	564	$5.43 \cdot 10^{-7}$	$4.95 \cdot 10^{-5}$	$7.82 \cdot 10^{-4}$	1.00
**1 to 3 non-coop**	13060	4353	1451	$1.14 \cdot 10^{-5}$	$3.91 \cdot 10^{-4}$	3.99
**1 to 3 statistical**	7427	2476	825	$5.30 \cdot 10^{-5}$	$7.99 \cdot 10^{-4}$	0.94

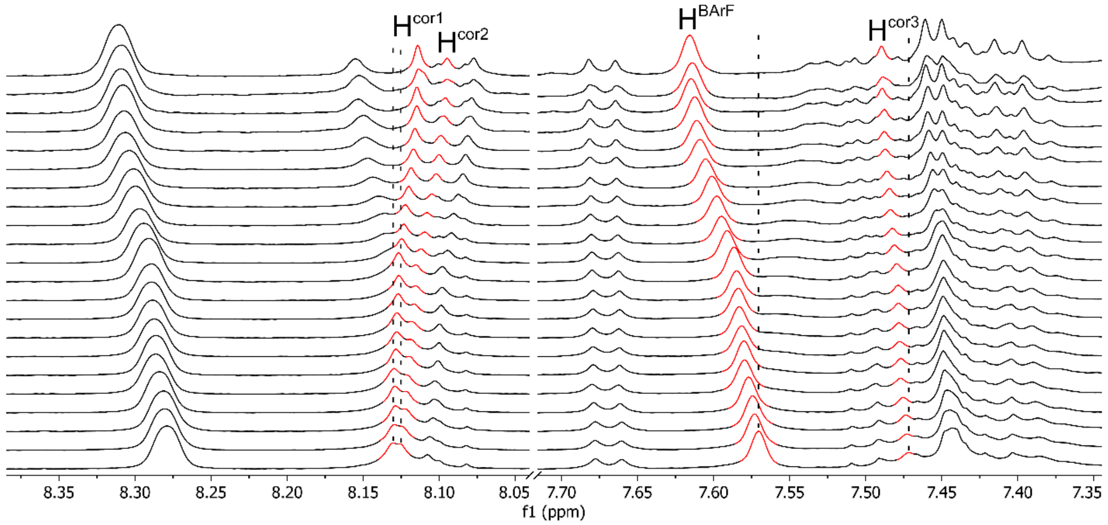

Figure S 158. Stacked ^{1}H-NMR (500 MHz) spectra for the titration of **Ru3C·BAr^F$_4$** with variable concentrations of C_{70} in toluene-d_8 at 298 K. The most significant shifts of the complex (H^{cor1}, H^{cor2}, H^{BArF} and H^{cor3}) have been labelled.

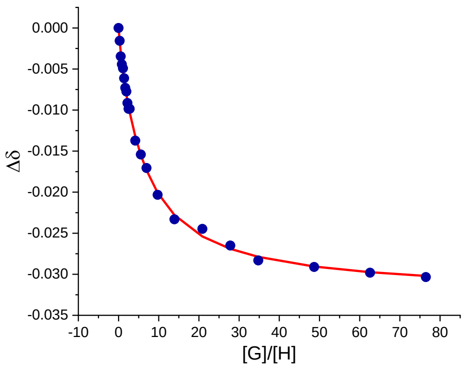

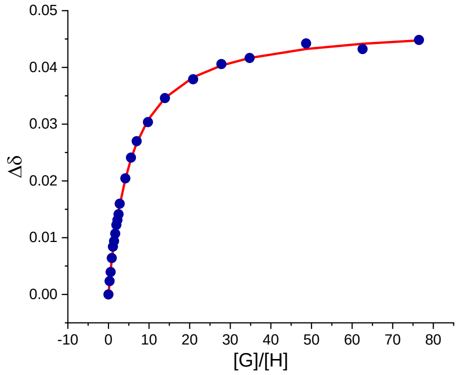

Figure S 159. Non-linear regression for $\Delta\delta$ of selected protons (left plot: H^{cor2}, right plot: H^{cor4}) for the titration of complex **Ru3C·BAr^F$_4$** with C_{70} obtained with the 1:3 non-cooperative binding model.

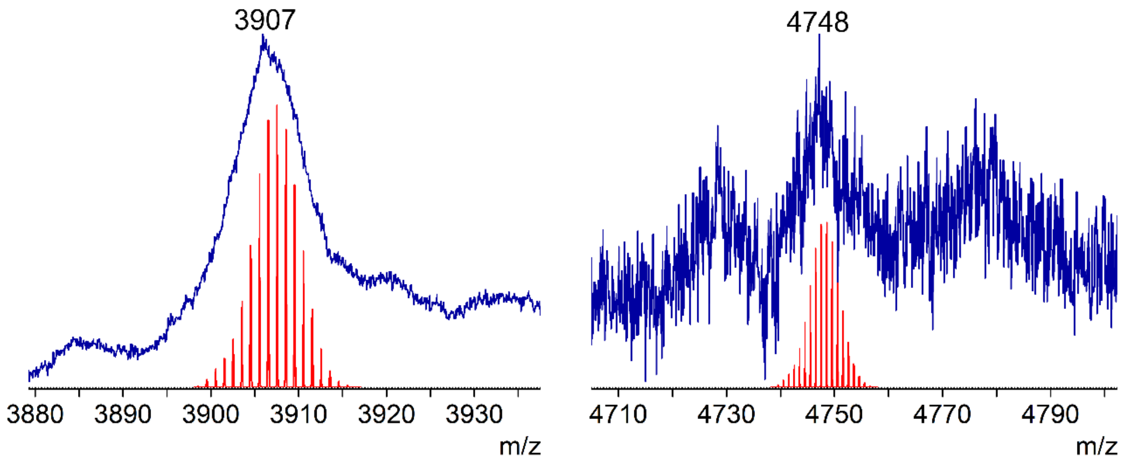

Figure S 160. Detection of adducts **C_{70}@Ru3C·BAr$^{F}_{4}$** (left) and **$(C_{70})_2$@Ru3C·BAr$^{F}_{4}$** (right) in LRMS (MALDI-TOF, linear detection, positive mode). Calculated (red), measured (blue).

Table S 10. Summary of the association constants (M^{-1}) obtained for the adducts formed between the complexes and C_{60}. [a]Uncertainties for the 1 to 3 non-cooperative binding model were estimated with Monte Carlo simulations.[15,18] [b]In M^{-2}. [c]In M^{-3}.

Compound	K_1	K_2	K_3	β
**Ru1C·BAr$^{F}_{4}$**	(3.54 ± 0.19) × 10^2	-	-	-
**Ru2C·BAr$^{F}_{4}$**	(7.21 ± 0.15) × 10^2	(1.80 ± 0.04) × 10^2	-	1.30 × 10^5[b]
**Ru3C·BAr$^{F}_{4}$[a]**	(5.97 ± 0.27) × 10^3	(1.99 ± 0.09) × 10^3	(6.63 ± 0.30) × 10^2	7.88 × 10^9[c]

Table S 11. Summary of the association constants (M^{-1}) obtained for the adducts formed between the complexes and C_{70}. [a]Uncertainties for the 1 to 3 full and non-cooperative binding models were estimated with Monte Carlo simulations.[15,18] [b]In M^{-2}. [c]In M^{-3}.

Compound	K_1	K_2	K_3	β
**Ru1C·BAr$^{F}_{4}$**	(5.69 ± 0.08) × 10^2	-	-	-
**Ru2C·BAr$^{F}_{4}$ full**	(1.98 ± 0.08) × 10^3	(1.62 ± 0.08) × 10^3	-	3.22 × 10^6[b]
**Ru2C·BAr$^{F}_{4}$ non-coop**	(4.93 ± 0.05) × 10^3	(1.23 ± 0.01) × 10^3	-	6.08 × 10^6[b]
**Ru3C·BAr$^{F}_{4}$ full[a]**	(1.16 ± 0.05) × 10^9	(2.76 ± 0.12) × 10^3	(9.96 ± 0.60) × 10^2	3.19 × 10^{15}[c]
**Ru3C·BAr$^{F}_{4}$ non-coop[a]**	(1.31 ± 0.05) × 10^4	(4.35 ± 0.18) × 10^3	(1.45 ± 0.06) × 10^3	8.25 × 10^{10}[c]

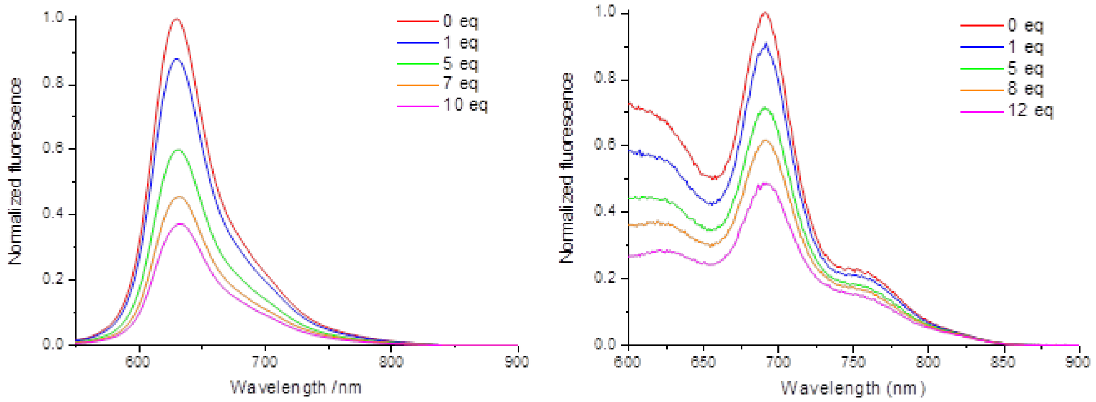

Figure S 161. Emission quenching experiments of compound **Ru1C** (left) and **Ru1P** (right) at a constant concentration of 3 × 10^{-4} M in toluene upon addition of several aliquots of C_{60}. Signal decay is of the same order of magnitude in both systems. Given that complex **Ru1P** does not undergo any fullerene binding as observed by NMR, the quenching is due to collisions between the emitter and the quencher (dynamic quenching),[19] precluding detailed analysis.

DOSY experiments

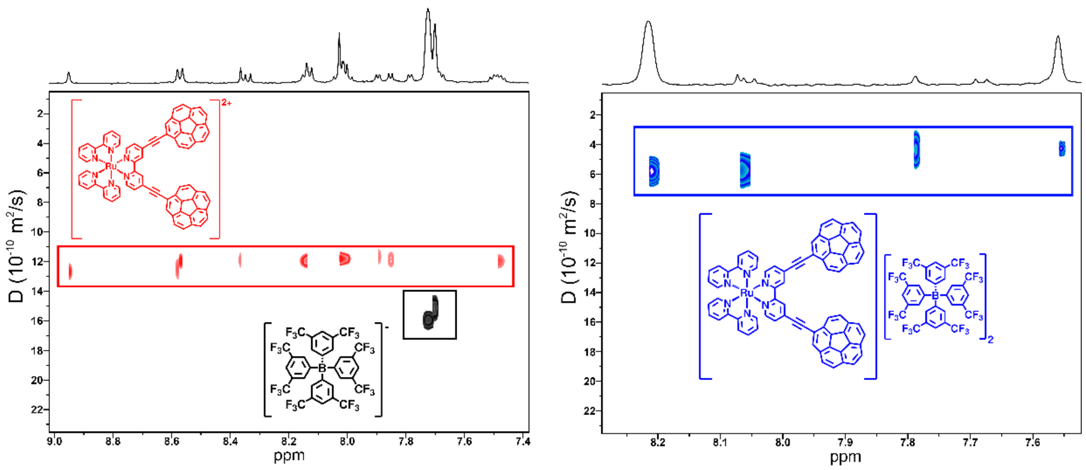

Figure S 162. 2D ^{1}H-DOSY NMR (298 K, 500 MHz) spectra of **Ru1C·BAr^F$_4$** in acetonitrile-d_3 (left) and toluene-d_8 (right).

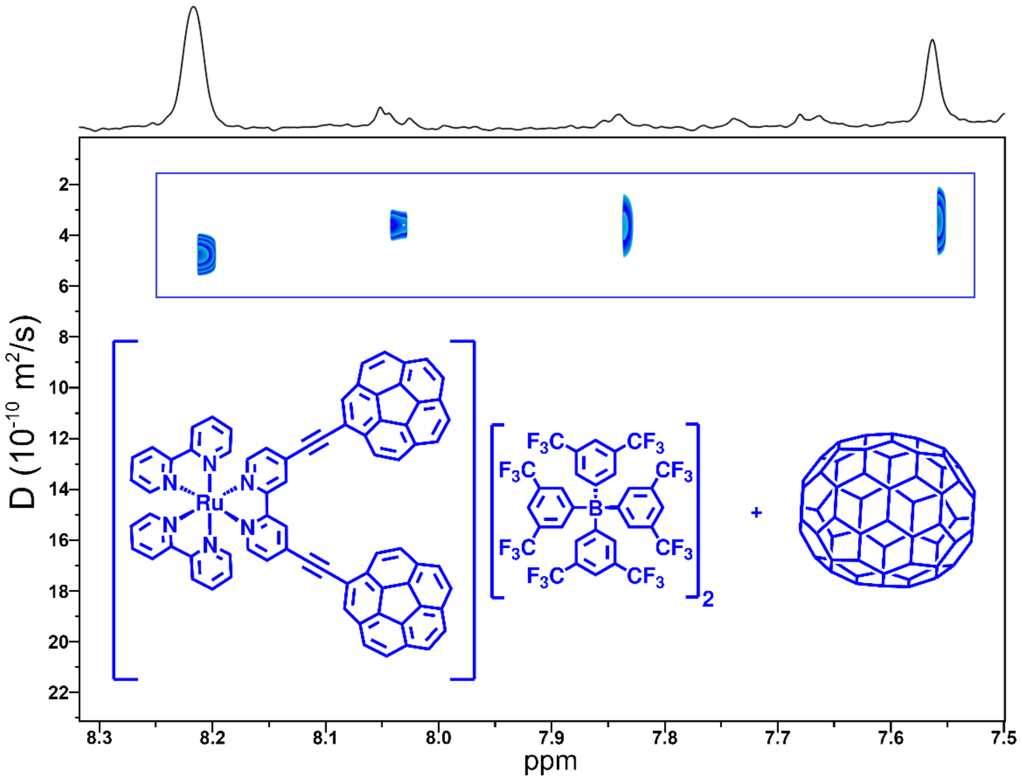

Figure S 163. 2D ^{1}H-DOSY NMR spectrum (298 K, 500 MHz) of the mixture of **Ru1C·BAr^F$_4$** with C_{70} in toluene-d_8.

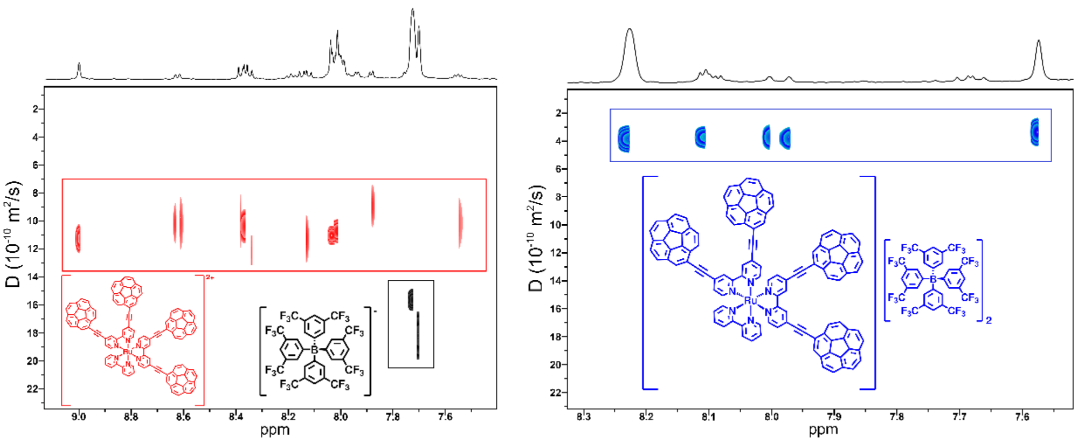

Figure S 164. 2D ^{1}H-DOSY NMR (298 K, 500 MHz) spectra of **Ru2C·BAr^F$_4$** in acetonitrile-d_3 (left) and toluene-d_8 (right).

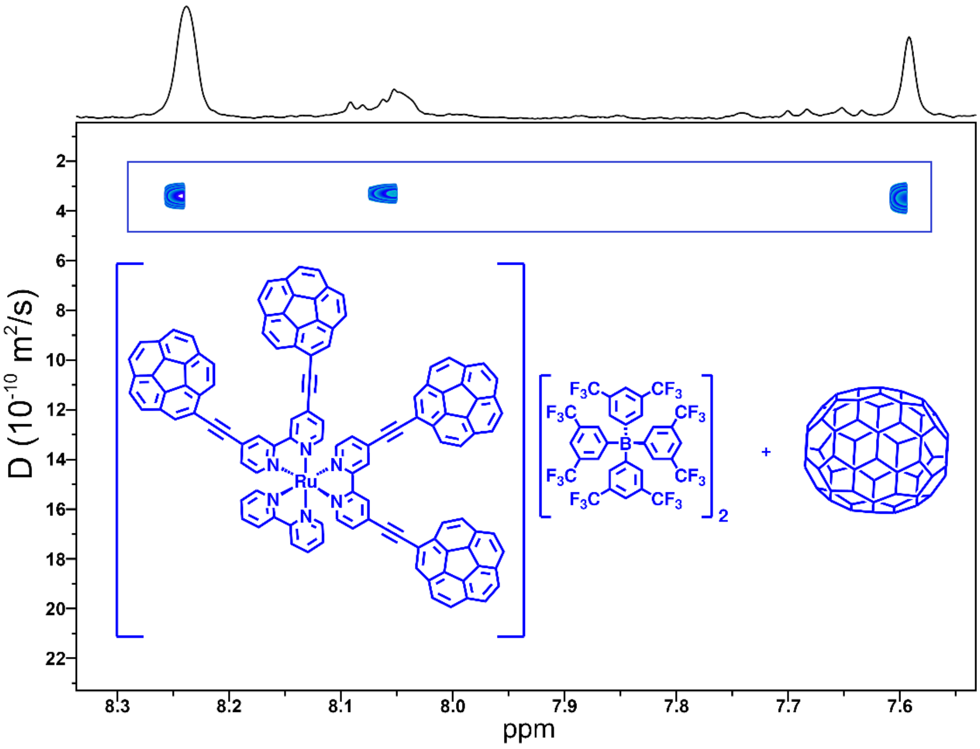

Figure S 165. 2D ^{1}H-DOSY NMR spectrum (298 K, 500 MHz) of the mixture of **Ru2C·BAr^F$_4$** with C_{70} in toluene-d_8.

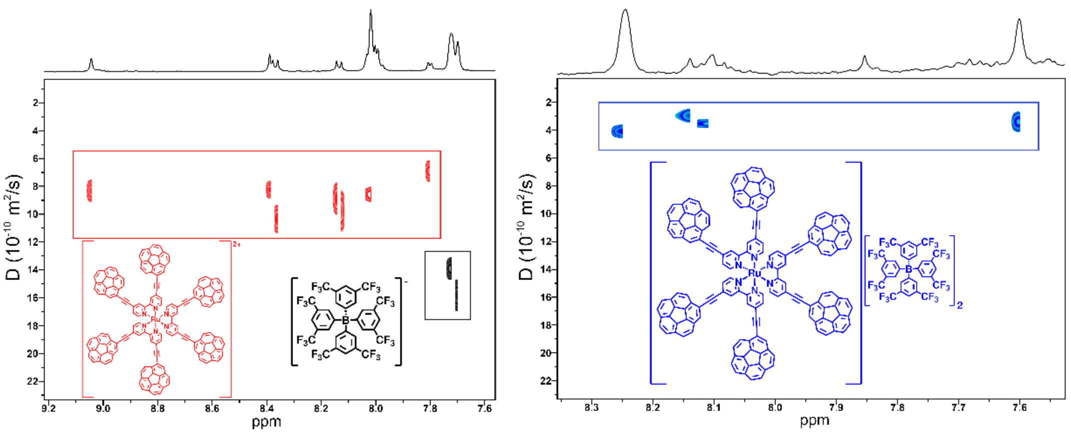

Figure S 166. 2D ^{1}H-DOSY NMR (298 K, 500 MHz) spectra of **Ru3C·BAr^F$_4$** in acetonitrile-d_3 (left) and toluene-d_8 (right).

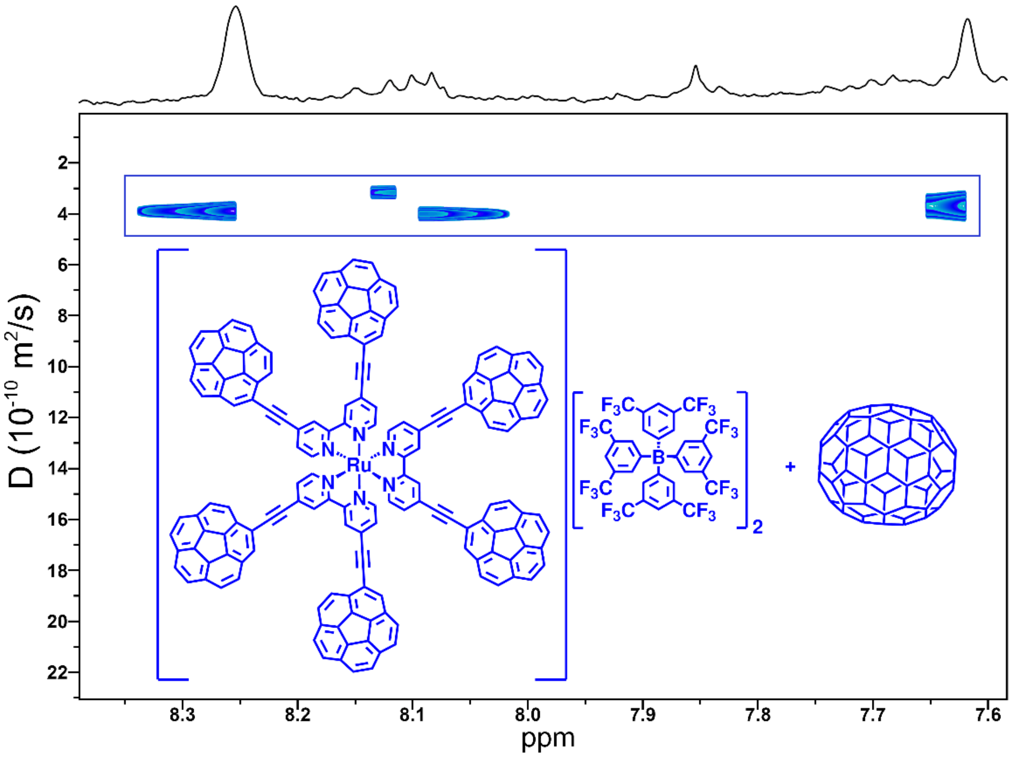

Figure S 167. 2D ^{1}H-DOSY NMR spectrum (298 K, 500 MHz) of the mixture of **Ru3C·BAr^F$_4$** with C_{70} in toluene-d_8.

Computational Calculations Details

In order to optimize the geometries of the complexes **Ru1C**, **Ru2C** and **Ru3C** prepared in this work, DFT calculations were performed. Different functionals were tested, namely CAM-B3LYP,[20] PBE1PBE (also known as PBE0),[21–24] TPSSh,[25,26] and wB97XD.[27] People and collaborators' split valence basis set 6-31G(d,p)[28–31] was chosen for light atoms along with Los Alamos Electron Core Potential and the associated double zeta basis set LANL2DZ[32,33] for Ru atom. The functional PBE1PBE was chosen due to the lowest computational cost.

In the case of the supramolecular adducts **C_{60}@Ru1C**, **$(C_{60})_2$@Ru2C** and **$(C_{60})_3$@Ru3C**, the following procedure was followed. First, the C_{60} fragment was placed at an idealized position (halfway between two corannulene centers), and the structure of a surrogate protonated bpy **C_{60}@HL1C** was used replacing the metal fragment by a hydrogen to reduce the computational cost. This structure was optimized using PBE1PBE[21–24] functional with the Becke-Johnson damping empirical dispersion correction[34], and 6-31G(d,p)[28–31] as the basis set. Then, for **C_{60}@Ru1C**, the hydrogen atom was replaced by $Ru(bpy)_2$ fragment and the resulting structure was pre-optimized using Grimme's GFN2-xTB semiempirical quantum mechanical method[35,36] with toluene as the solvent of choice, keeping the supramolecular assembly moiety frozen. The resulting structure was finally optimized in gas phase using PBE1PBE[21–24] functional along with 6-31G(d,p)[28–31] basis set for light atoms and LANL2DZ[32,33] for the Ru atom. In the case of **$(C_{60})_2$@Ru2C**, the **C_{60}@HL1C** fragment was duplicated, the second fragment placed at an idealized position in an octahedral geometry and the proton replaced by Ru(bpy) fragment. For this adduct, two possible conformers were defined (v_1 and v_2 respectively) depending on the relative orientation of the corannulene moieties. Both conformers were optimized following the same protocol defined for **C_{60}@Ru1C**. Finally, for **$(C_{60})_3$@Ru3C**, surrogate aduct **C_{60}@HL1C** was triplicated and placed in an octahedral geometry, replacing the central proton by a Ru(II) atom. Four different conformers arose for this adduct, named v_1v_1, v_1v_2, v_2v_1 and v_2v_2, with different relative orientation of the three corannulene units. All conformers were optimized following the same protocol defined for **C_{60}@Ru1C**. Once all the geometries were optimized, the impact of the solvent was described with single-point calculations via the Polarizable Continuum Model (PCM)[37,38] choosing toluene as the solvent (ε = 2.3741).

Interaction energies were calculated in the gas phase over the structure of the most stable conformer for each adduct (**C_{60}@Ru1C**, **$(C_{60})_2$@Ru2C – v_1** and **$(C_{60})_3$@Ru3C – v_2v_2**), considering basis set superposition error (BSSE) with the Boys-Bernardi functional counterpoise scheme[39–42] using equation 25.

$$E_{int}(AB) = E_{AB}^{\alpha\beta}(AB) - E_{AB}^{\alpha\beta}(A) - E_{AB}^{\alpha\beta}(B) \qquad \text{eq. 25}$$

Subscripts denote the geometry used (AB, inclusion complex in all cases) and the superscripts refer to the basis set ($\alpha\beta$, the one belonging to supramolecular assembly in all cases). A and B correspond to host and guest entities that interact to furnish the AB adduct. For the multitopic hosts **Ru2C – v_1** and **Ru3C – v_2v_2**, the fullerenes were treated as a single entity.

All DFT calculations described above were performed in Gaussian 16 package.[43]

Non-covalent interactions were evaluated by the location of critical points where the reduced density gradient decreases at low electronic density values according to Yang and collaborators' scheme with the help of the NCIPlot package[44,45] and Multiwfn.[46] Calculations were performed with promolecular densities, and gradient isosurfaces were plotted with an isovalue of 0.3 a.u. and colored on a RGB scale according to values of the sign of λ_2 (second eigenvalue of the electron-density Hessian). Red indicates repulsion, green means weak attraction, and blue represents strong attraction. Graphics were visualized in VMD[47] and Gnuplot.[48]

Ru1C E = -3262.5522019 a. u

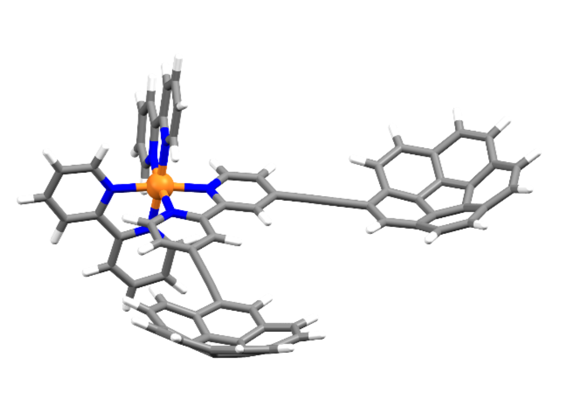

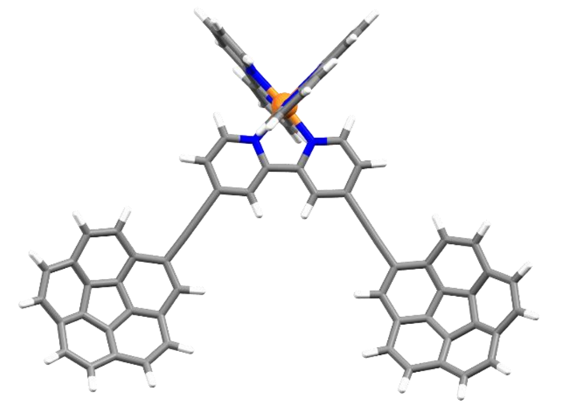

C	2.660079	-2.863084	0.018376	C	-10.877752	4.811114	0.861419	N	-1.419243	-5.883675	0.253273
C	0.743527	-1.557549	-0.124282	H	-11.334666	2.751688	1.144165	N	1.457067	-5.887861	-0.007042
C	1.507354	-0.404513	-0.203936	C	-9.751862	7.168487	0.828920	N	-0.304105	-4.379037	2.088155
C	2.913526	-0.475904	-0.173528	H	-8.436109	8.825998	1.063119	C	-3.297009	-7.868396	0.756618
C	3.483492	-1.760812	-0.055071	H	-11.732055	5.116103	1.459914	C	-0.933078	-4.448129	4.792860
H	3.072458	-3.861961	0.108118	H	-10.519861	7.654212	1.425128	C	-1.839292	-6.115736	1.521745
H	1.036928	0.566075	-0.296690	C	7.472409	1.563806	0.053273	C	-1.232754	-5.254059	2.547639
H	4.559891	-1.881586	-0.027377	C	6.674189	2.645624	-0.458341	C	-2.780454	-7.106587	1.795912
N	1.318303	-2.783634	-0.011824	C	8.791318	1.751486	0.432699	C	-1.562770	-5.305517	3.900657
C	-0.730044	-1.562772	-0.152478	C	7.368760	3.805518	-0.722781	C	-1.918301	-6.624065	-0.746786
C	-1.498717	-0.413387	-0.237806	C	5.226027	2.808427	-0.489600	C	0.298794	-3.549877	2.952107
C	-2.640992	-2.883767	-0.114733	C	9.441354	3.035493	0.339996	C	-2.857704	-7.622678	-0.540351
C	-2.904326	-0.494895	-0.269349	H	9.316765	0.921667	0.898510	C	0.015888	-3.553214	4.309573
H	-1.032369	0.562501	-0.283730	C	8.717820	3.992862	-0.344168	H	-3.107863	-7.286940	2.812719
C	-3.468954	-1.785828	-0.201514	C	6.755375	5.079935	-0.790769	H	-2.308418	-6.005348	4.258256
H	-3.049261	-3.887100	-0.064667	C	4.627491	4.080624	-0.556265	H	-1.545000	-6.392403	-1.738006
H	-4.544655	-1.914349	-0.219927	C	10.579840	3.547594	1.062312	H	1.028010	-2.871196	2.524483
N	-1.299576	-2.795215	-0.095671	C	8.936242	5.380645	-0.182540	H	-3.232667	-8.193009	-1.382735
C	-5.222331	2.781699	-0.623116	C	5.397081	5.282240	-0.609067	H	0.531012	-2.863475	4.968632
C	-6.669197	2.610284	-0.665684	C	7.724703	6.052478	-0.464976	H	-1.182148	-4.477733	5.848400
C	-4.629611	4.058340	-0.633359	H	3.544624	4.129591	-0.478810	H	-4.029965	-8.642681	0.957509
C	-7.357682	3.771790	-0.938783	H	11.244700	2.856390	1.573329	N	0.331966	-4.603551	-1.997888
C	-7.484231	1.513200	-0.217004	C	10.790935	4.906261	1.223858	C	3.356902	-7.895357	-0.288418
C	-5.403603	5.256571	-0.697661	C	9.888008	5.890229	0.678580	C	0.972693	-4.958753	-4.677337
H	-3.552130	4.111559	-0.502191	C	5.030054	6.633014	-0.251520	C	1.885089	-6.250046	-1.241922
C	-6.750112	5.050751	-0.949582	C	7.394207	7.274168	0.091784	C	1.273218	-5.511455	-2.356844
C	-8.724757	3.943416	-0.622583	H	11.613224	5.229608	1.856578	C	2.837760	-7.254345	-1.405027
H	-7.023527	0.546298	-0.033886	C	9.655026	7.254925	1.085401	C	1.609588	-5.705495	-3.695246
C	-8.821281	1.685301	0.101445	C	5.982226	7.577440	0.083889	C	1.958235	-6.512436	1.068198
C	-5.063296	6.601270	-0.293766	H	3.980015	6.895780	-0.156496	C	-0.278754	-3.882330	-2.948840
C	-7.740503	6.010633	-0.650709	C	8.469296	7.914447	0.806257	C	2.908349	-7.517676	0.973198
C	-9.474475	2.967412	0.004705	H	10.390316	7.758297	1.707635	C	0.009641	-4.029884	-4.297164
C	-8.959887	5.326134	-0.442135	H	5.645087	8.549283	0.434447	H	3.172030	-7.538694	-2.395480
H	-9.363359	0.842610	0.522980	H	8.320238	8.908344	1.219323	H	2.365813	-6.429935	-3.972581
H	-4.020884	6.867689	-0.141930	H	7.008660	0.598041	0.234728	H	1.577805	-6.180403	2.027667
C	-6.036844	7.532932	0.014943	C	-3.700786	0.653151	-0.370661	H	-1.018893	-3.171003	-2.600392
C	-7.445524	7.221793	-0.052542	C	-4.392538	1.653309	-0.481429	H	3.284451	-7.991304	1.873033
C	-10.650138	3.457462	0.681299	C	3.706251	0.675671	-0.263456	H	-0.512017	-3.423288	-5.028859
C	-9.955816	5.811663	0.382151	C	4.396741	1.677843	-0.363268	H	1.226935	-5.099422	-5.722637
H	-5.723538	8.498759	0.402303	Ru	0.013320	-4.395575	0.040270	H	4.098742	-8.678745	-0.402786
C	-8.558321	7.840469	0.622131								

Ru2C E = - 4946.9679613 a. u

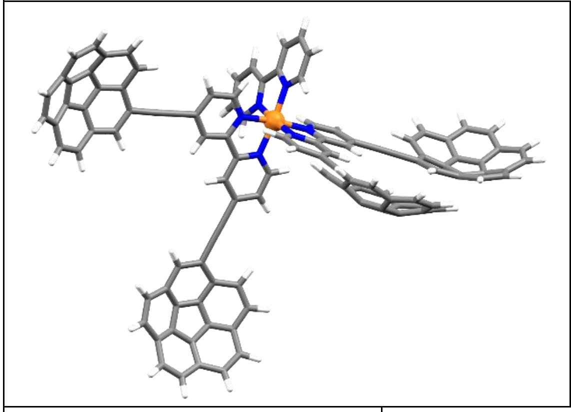

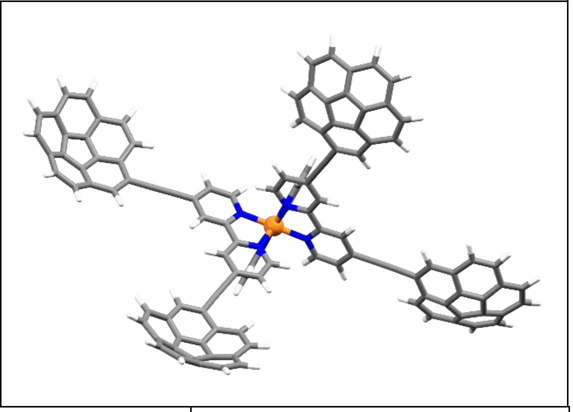

C	2.636124	-1.148903	-3.687314
C	2.666634	0.544885	-2.098513
C	4.046340	0.457835	-1.998753
C	4.759625	-0.471410	-2.778063
C	4.006290	-1.292788	-3.641012
H	2.031509	-1.766063	-4.342762
H	4.595145	1.105863	-1.327308
H	4.499183	-2.027103	-4.266986
N	1.964061	-0.256935	-2.940051
C	1.851668	1.494901	-1.320705
C	2.379877	2.374317	-0.388837
C	-0.292565	2.292071	-0.916468
C	1.539288	3.260185	0.310150
H	3.443056	2.394177	-0.185769
C	0.161475	3.195396	0.020796
H	-1.346629	2.220917	-1.160359
H	-0.536419	3.850199	0.528887
N	0.520365	1.457930	-1.586733
C	2.992173	5.928757	2.961041
C	2.056741	6.825789	3.627285
C	4.363174	5.929540	3.273419
C	2.665664	7.793275	4.396219
C	0.636630	6.701875	3.820624
C	4.921731	6.811656	4.249114
H	4.983846	5.168827	2.807568
C	4.048110	7.786064	4.701770
C	1.994922	8.479430	5.434529
H	0.074988	5.999333	3.211018
C	-0.017430	7.367952	4.843856
C	6.141545	6.692568	5.013488
C	4.232339	8.473302	5.920932
C	0.681063	8.220650	5.773790
C	2.962904	8.897021	6.377132
H	-1.073822	7.163936	4.999110
H	6.912355	5.999330	4.687831
C	6.311614	7.355075	6.215095
C	5.287256	8.205868	6.773574
C	0.318546	8.618730	7.111787
C	2.672564	9.074382	7.715644
H	7.210445	7.155456	6.792486
C	5.064556	8.602885	8.140875
C	1.266920	9.023802	8.035424
H	-0.712093	8.514410	7.440340
C	3.820214	9.015318	8.588150
H	5.865112	8.492269	8.867290
H	0.944399	9.221196	9.054328
H	3.693328	9.212073	9.649504
C	8.927269	-2.904265	-4.091157
C	9.446887	-1.712506	-3.475358
C	9.755150	-3.953240	-4.455613
C	10.821206	-1.617133	-3.462646
C	8.781021	-0.728373	-2.631721
C	11.180636	-3.916137	-4.240205
H	9.297266	-4.864989	-4.830816
C	11.665724	-2.685076	-3.843460
C	11.534736	-0.802290	-2.550818
C	9.499871	0.074728	-1.728517
C	12.123316	-5.000315	-4.113479
C	12.898894	-2.527001	-3.169585
C	10.919334	-0.016247	-1.591143
C	12.819510	-1.360027	-2.375277

H	8.928117	0.717421	-1.064456
H	11.849523	-5.991403	-4.465374
C	13.328717	-4.846187	-3.450208
C	13.715452	-3.593502	-2.848557
C	11.767647	0.359431	-0.484043
C	13.553302	-1.187557	-1.216342
H	13.954758	-5.722790	-3.306036
C	14.642755	-3.332831	-1.774264
C	13.019078	-0.201199	-0.307033
H	11.385588	1.031233	0.279810
C	14.566843	-2.188421	-0.997637
H	15.369538	-4.093491	-1.501389
H	13.574581	0.048678	0.593019
H	15.236120	-2.095121	-0.146666
H	7.852261	-3.027625	-4.190279
C	2.053766	4.170061	1.246319
C	2.506298	4.975030	2.043862
C	6.157475	-0.567650	-2.703275
C	7.374887	-0.633043	-2.652363
Ru	-0.086644	0.058820	-2.989806
N	-2.123673	0.393270	-2.783012
N	-0.526875	-1.266793	-4.520689
N	-0.561231	-1.198308	-1.412554
C	-4.880384	0.697458	-2.341598
C	-1.386635	-2.758998	0.770488
C	-2.741454	-0.302358	-1.793470
C	-1.857725	-1.182388	-1.008556
C	-4.100641	-0.171042	-1.555811
C	-2.291206	-1.944684	0.064699
C	-2.861511	1.218104	-3.545152
C	0.310993	-1.972135	-0.744732
C	-4.215769	1.402418	-3.365429
C	-0.047861	-2.757120	0.330393
H	-4.581147	-0.730835	-0.763513
H	-3.326608	-1.920030	0.379877
H	-2.323430	1.749826	-4.322150
H	1.333984	-1.946665	-1.103281
H	-4.762890	2.085237	-4.004508
H	0.695822	-3.361687	0.835782
N	0.218311	1.240994	-4.665106
C	-1.136023	-2.868886	-6.707054
C	0.658433	2.631752	-7.029497
C	-0.420584	-0.769883	-5.778101
C	0.024264	0.629232	-5.859770
C	-0.720618	-1.556885	-6.888647
C	0.239251	1.308647	-7.057570
C	-0.932762	-2.533069	-4.349518
C	0.625763	2.518057	-4.642424
C	-1.244759	-3.366690	-5.412474
C	0.855065	3.248726	-5.798239
H	-0.635637	-1.149868	-7.889039
H	0.085653	0.809944	-8.006983
H	-1.000988	-2.871000	-3.321591
H	0.764941	2.951319	-3.658402
H	-1.567798	-4.384010	-5.222345
H	1.183449	4.279549	-5.727407
H	0.830643	3.171008	-7.955009
H	-1.373384	-3.491169	-7.563474
H	-6.936747	-7.740402	6.571969
H	-15.724941	-0.185130	-1.404448

C	-2.380655	-8.188145	7.108461
C	-12.787714	3.189578	-5.125462
C	-13.131934	-0.373021	-0.074823
H	-16.103869	1.154462	-3.381417
H	-0.921054	-11.223948	7.133182
C	-4.835995	-8.099680	7.081892
C	-12.883836	1.623786	-1.344346
H	0.765454	-10.025879	5.880676
H	-8.218075	2.582419	-4.183525
C	-6.255023	0.856855	-2.109584
C	-5.704598	-6.247398	5.691900
H	-3.629432	-11.216534	7.890300
C	-14.970362	0.543783	-1.687439
C	-2.541466	-4.943558	3.957598
C	-15.186762	1.311652	-2.819746
C	-1.396559	-7.488708	6.372092
C	-10.217694	3.148061	-4.662637
C	-1.547878	-5.764472	4.637488
H	-4.593705	-4.392435	3.729558
C	-9.466271	0.453990	-0.674646
C	-11.611115	3.170115	-4.291591
C	-2.009781	-6.353350	5.794273
C	-10.871368	0.550782	-0.433329
C	-3.373958	-6.355743	6.173268
H	-8.872902	-0.289351	-0.148995
C	-9.555488	2.151508	-2.507432
C	-11.772692	-0.398626	0.177157
H	-6.554808	-5.786905	5.196043
C	-4.372429	-5.758578	5.422331
H	0.175677	-5.809027	3.291889
C	-2.163423	-4.184629	2.831274
C	-13.106064	2.409498	-2.498619
H	-12.699690	3.482091	-6.168398
C	0.312611	-7.379019	4.728748
C	-4.788443	-9.469545	7.526887
C	-14.013283	2.734715	-4.669750
C	-3.592772	-10.159451	7.640279
C	-3.601293	-7.484463	6.990034
H	-14.840487	2.686692	-5.372999
H	-9.931095	3.434343	-5.671399
H	-13.748771	-1.167604	0.336611
C	-9.236718	2.662413	-3.813907
C	-1.076932	-10.166411	6.937037
C	-7.446147	1.007950	-1.891967
C	-5.921894	-7.362643	6.479766
H	-11.370187	-1.208922	0.778982
C	-10.854292	2.365834	-2.100827
C	-3.893934	-4.954959	4.341782
C	-0.296821	-8.109755	5.812401
H	-5.716025	-10.010868	7.692289
C	-14.188110	2.216735	-3.334904
H	1.221144	-7.763553	4.272214
C	-0.282449	-6.261457	4.167158
C	-8.820709	1.219086	-1.662254
C	-0.111567	-9.480877	6.219605
C	-2.323567	-9.551417	7.322153
C	-11.853048	2.870218	-2.965023
C	-13.730634	0.602584	-0.955086
C	-1.801698	-3.530844	1.866741
C	-11.493442	1.591178	-1.102831

Ru3C E = - 6631.3841534 a. u

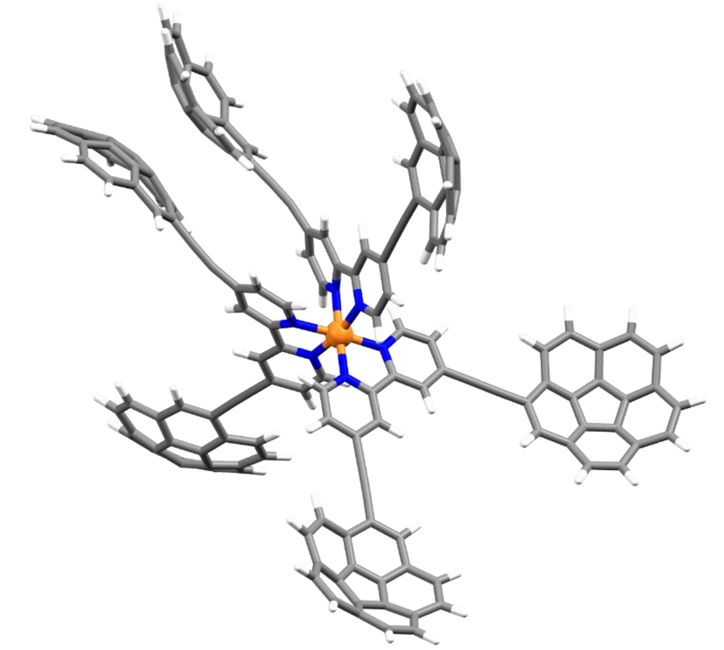

C	-2.484056	0.540506	2.600578	N	0.575611	-0.175678	-1.355003	C	-1.514118	-13.812326	2.734532
C	-3.813584	0.469025	0.695453	N	0.599001	0.311631	1.316699	C	5.809895	-0.800558	-2.442888
C	-4.956881	0.659224	1.456217	N	-0.935495	-1.892009	-0.076377	C	2.786645	-12.610200	-0.672164
C	-4.864706	0.798243	2.852549	C	3.210083	-0.669375	-2.146754	H	9.683816	0.596450	-5.728922
C	-3.576514	0.732679	3.419693	C	-0.548129	-4.670891	-0.175190	C	9.329859	-1.128659	-1.467226
H	-1.477609	0.493961	3.001601	C	1.102248	-1.419647	-1.232643	C	1.125383	-9.196998	-0.948614
H	-5.931785	0.711108	0.988023	C	0.181599	-2.412885	-0.649825	C	-2.233496	-11.613456	1.867519
H	-3.443337	0.839374	4.489668	C	2.413390	-1.689077	-1.595793	H	2.635451	-15.061983	0.907445
N	-2.583183	0.406234	1.267908	C	0.398717	-3.779332	-0.712692	C	12.519232	-2.700003	-1.504449
C	-3.818746	0.323710	-0.770442	C	1.289408	0.767232	-1.988906	H	-3.279423	-9.934140	2.807096
C	-4.970114	0.329871	-1.542654	C	-1.849323	-2.735140	0.429679	C	-2.048057	-9.232552	1.219744
C	-2.501155	0.036213	-2.663622	C	2.584586	0.562493	-2.416823	C	7.222050	-0.748568	-2.474903
C	-4.888257	0.188345	-2.939410	C	-1.700766	-4.106829	0.406959	C	-2.322873	-12.691990	2.820931
H	-5.944373	0.442976	-1.084022	H	2.849082	-2.663738	-1.412239	C	-0.535940	-13.969979	1.686195
C	-3.602088	0.034225	-3.493898	H	1.283739	-4.174526	-1.196491	C	10.301710	-1.919460	-0.817434
H	-1.496986	-0.083380	-3.055104	H	0.805742	1.731364	-2.103509	C	12.079450	-0.832295	-3.704212
H	-3.477697	-0.084308	-4.563685	H	-2.724409	-2.271923	0.872808	C	-0.356211	-6.061094	-0.232779
N	-2.589417	0.183793	-1.331584	H	3.138249	1.364890	-2.889556	C	9.958893	-0.454869	-2.537482
C	-8.175714	0.280337	-5.276521	H	-2.468991	-4.744802	0.827533	C	11.321809	-0.506436	2.601267
C	-8.011445	0.117422	-6.715071	N	-0.649151	2.220583	0.025803	H	5.532175	12.639257	0.759804
C	-9.447480	0.392085	-4.689270	C	3.269262	0.437926	2.131721	C	2.703612	14.774365	-1.611510
C	-9.171731	0.306814	-7.433618	C	0.114576	4.920186	0.137444	C	0.271791	9.804890	-0.230423
C	-6.923390	-0.457702	-7.460081	C	1.292671	1.471418	1.200856	C	11.343826	-1.151441	4.887318
C	-10.649481	0.363455	-5.462849	C	0.522856	2.582401	0.611793	C	-0.738588	9.652231	-1.243143
H	-9.498449	0.414028	-3.604000	C	2.625187	1.557703	1.575609	C	13.333844	0.080359	3.710170
C	-10.447799	0.421799	-6.831294	C	0.924690	3.905934	0.681308	C	12.772128	1.181849	1.563661
C	-9.352557	-0.199567	-8.741301	C	1.170885	-0.720804	1.954926	H	13.563875	2.955727	0.547675
H	-5.957973	-0.587206	-6.978604	C	-1.432824	3.182061	-0.487617	C	0.493695	6.270764	0.203228
C	-7.104179	-0.960697	-8.737828	C	2.478142	-0.696474	2.394231	C	8.104498	0.517945	1.447564
C	-11.996687	0.027360	-5.065400	C	-1.097863	4.520326	-0.459057	C	12.065720	-0.605523	3.760705
C	-11.415361	-0.004708	-7.766843	H	3.192290	2.463313	1.397473	H	7.094035	-1.067083	4.354261
C	-8.387327	-0.940212	-9.395679	H	1.850213	4.175127	1.175727	H	6.884153	1.973934	0.360594
C	-10.738364	-0.393897	-8.945604	H	0.557987	-1.609151	2.063808	C	11.662680	0.361693	1.539239
H	-6.269271	-1.467354	-9.215262	H	-2.358510	2.844792	-0.941499	C	7.780043	-0.766850	3.566759
H	-12.259342	0.025506	-4.010949	H	2.912177	-1.567610	2.870247	C	4.659487	0.403838	2.356577
C	-12.937241	-0.403979	-5.982655	H	-1.766881	5.258233	-0.885629	C	8.948986	2.240814	-0.104759
C	-12.632452	-0.539447	-7.387403	H	3.776464	-13.058066	-0.701695	C	1.184510	13.219137	-0.657118
C	-8.890659	-1.771061	-10.461737	H	13.934738	-1.702791	-4.482120	H	-1.848104	10.525027	-2.835398
C	-11.238332	-1.344990	-9.813372	C	-0.622000	-13.031989	0.676134	H	-1.124336	8.661108	-1.465633
H	-13.905569	-0.732319	-5.614244	C	11.095782	-3.960480	0.083795	C	4.166703	11.054733	1.142978
C	-13.263724	-1.357836	-8.391754	C	11.447243	-0.193058	-4.835637	H	9.482708	-1.693109	5.766960
C	-10.247280	-1.963922	-10.659149	H	14.300049	-3.246037	-2.661068	C	0.641429	11.106350	0.029882
H	-8.193215	-2.336882	-11.073727	H	-1.561869	-14.546850	3.534167	C	7.890953	1.597178	0.518745
C	-12.599552	-1.740960	-9.545193	C	1.695579	-13.345313	-0.077223	H	11.870432	-1.328989	5.821375
H	-14.253941	-1.764772	-8.205320	C	11.319853	-0.829821	-2.550672	C	11.545374	2.621756	-0.036100
H	-10.562966	-2.674636	-11.418387	H	-2.975024	-12.590118	3.684324	C	1.153848	8.811144	0.367536
H	-13.095352	-2.434645	-10.219220	H	6.568970	-2.685428	-0.347927	C	-0.525760	13.127580	-2.819323
C	-6.852814	1.168821	7.422249	C	4.593741	-0.826662	-2.359517	C	10.324158	1.871875	0.134107
C	-7.942714	1.587280	6.581578	C	2.641938	-11.306145	-1.108778	C	3.707146	14.331504	-0.765759
C	-7.023786	0.954508	8.779889	H	0.782884	-15.579449	2.372602	H	4.961825	10.409192	1.506181
C	-9.091008	1.944513	7.253964	C	13.242165	-1.684099	-3.644852	C	5.860017	0.212468	2.449907
C	-8.123194	1.441073	5.143192	C	-0.045061	-8.668579	-0.377687	C	2.379653	9.172061	0.952862
C	-8.298159	1.135199	9.430336	C	13.451054	-2.570169	-2.599366	H	14.016024	0.006042	4.552861

H	-6.189168	0.550297	9.347125	C	-1.445270	-11.885704	0.766230	C	12.705920	2.296041	0.647528
C	-9.261771	1.733075	8.641474	C	8.574732	-3.230529	0.132854	C	9.975376	-1.358393	4.858023
C	-10.372996	1.948276	6.653390	C	-1.059032	-9.528911	0.217869	C	9.922697	-0.693175	2.577501
C	-9.400715	1.445297	4.557836	H	1.879959	-8.492614	-1.288267	C	4.491138	12.329923	0.717908
C	-8.798974	0.560612	10.654747	C	7.853925	-0.090980	-3.543436	C	3.515719	13.210122	0.119058
C	-10.647296	1.609140	8.896088	C	9.989069	-3.051656	-0.094848	C	10.474550	0.708690	0.859514
C	-10.593039	1.601873	5.331190	C	-0.867294	-10.870516	-0.030227	C	13.669248	0.928275	2.665954
C	-11.334086	1.747536	7.667932	C	9.280056	-0.020318	-3.660753	C	9.399319	0.058219	1.502051
H	-9.465268	1.235886	3.493475	C	0.314740	-11.391817	-0.609248	C	0.818427	7.443742	0.283414
H	-8.101407	0.128858	11.367574	H	7.221303	0.299816	-4.335886	H	-1.177348	13.121006	-3.689094
C	-10.155438	0.436015	10.901987	C	7.984223	-1.408353	-1.423455	C	-0.592857	12.040529	-1.873782
C	-11.148918	0.874552	9.952616	C	10.119338	0.197634	-4.816679	H	8.729313	3.102966	-0.729330
C	-11.949786	1.210267	5.028128	H	3.520519	-10.777226	-1.468220	H	11.542063	3.520766	-0.646752
C	-12.563222	1.163825	7.423781	C	1.386536	-10.602074	-0.989017	C	9.182433	-1.030179	3.695452
H	-10.470836	-0.089776	11.799369	H	-2.297260	-8.196451	1.431969	C	-1.149994	10.718941	-2.024999
C	-12.518798	0.452333	9.788749	C	-0.192578	-7.267612	-0.304351	H	3.028118	8.369513	1.293924
C	-12.884215	1.000419	6.025597	C	11.531271	-1.736747	-1.487385	H	0.486633	14.866754	-3.511033
H	-12.226231	0.987344	4.001121	H	10.965653	-4.850859	0.693241	C	7.251694	-0.030914	2.493150
C	-13.192539	0.590001	8.586450	C	-2.607280	-10.227355	2.004611	H	4.689340	14.789893	-0.843643
H	-13.015452	-0.072382	10.600751	C	1.725263	-14.475251	0.816907	C	1.877626	11.458519	0.623237
H	-13.861705	0.616752	5.745407	C	12.295135	-3.795176	-0.590394	C	0.214240	12.195861	-0.763487
H	-14.190670	0.168828	8.501377	C	0.663564	-14.771236	1.655740	H	2.938389	15.563669	-2.320892
H	-5.894961	0.925531	6.970639	C	0.462711	-12.728126	-0.178412	C	2.209623	12.765222	0.204390
C	-6.040272	0.203933	-3.743179	H	13.054775	-4.565274	-0.484808	C	1.406869	14.143526	-1.658854
C	-7.041818	0.236076	-4.438351	H	8.235173	-4.055021	0.754603	H	14.601647	1.482711	2.734004
C	-6.007903	1.002920	3.642950	H	12.000433	-0.088241	-5.765311	C	0.426616	14.127164	-2.716919
C	-7.000572	1.199872	4.323529	C	7.618832	-2.448959	-0.497858	C	2.828662	10.528248	1.006446
Ru	-0.995782	0.178263	-0.025933								

C_{60}@Ru1C E = - 5546.4878021 a. u

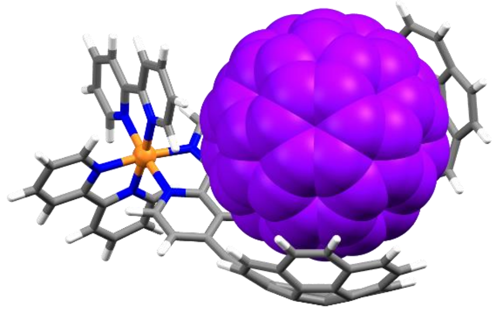

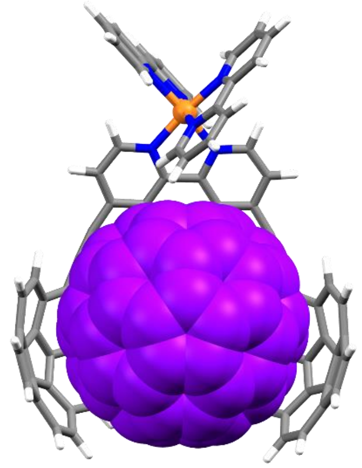

C	5.104127	2.621384	-0.648470	H	-0.839343	3.366346	-4.170863	C	-1.271449	2.164119	3.821600
C	3.985420	0.748346	-1.448082	H	-3.608232	6.845945	2.509106	C	-1.139715	2.932633	2.600513
C	2.962765	1.541917	-1.940757	C	-5.142154	6.316500	1.131770	C	-2.455890	3.412654	2.231465
C	2.988710	2.934394	-1.746675	C	-5.511689	5.941344	-0.211117	C	-3.400027	2.943160	3.224604
C	4.109462	3.471594	-1.088533	C	-3.727465	3.555312	-4.225504	C	-0.240135	2.531841	1.615945
H	5.978351	3.004089	-0.133822	C	-5.458482	4.548454	-2.766954	C	0.561493	1.340139	1.812527
H	2.104127	1.103640	-2.432464	H	-5.877943	6.241935	1.928103	C	0.672821	0.657724	0.536509
H	4.189022	4.538827	-0.918870	C	-6.621898	5.142305	-0.670700	C	-0.065262	1.428137	-0.444902
N	5.051062	1.288075	-0.802056	C	-5.040314	3.631348	-3.801135	C	-0.627425	2.583477	0.221645
C	3.972650	-0.722097	-1.512451	H	-3.453977	2.784139	-4.940437	C	0.428272	0.598670	2.985154
C	2.925853	-1.451764	-2.050926	C	-6.597536	4.479829	-1.886339	C	-0.501212	1.021073	4.013042
C	5.072754	-2.676802	-0.906454	H	-7.468797	4.977631	-0.009759	C	-1.095246	-0.165825	4.593121
C	2.932059	-2.856308	-1.985108	H	-5.754980	2.914849	-4.197223	C	-0.532085	-1.322126	3.926509
H	2.062971	-0.957373	-2.477759	H	-7.425506	3.821125	-2.134142	C	0.409825	-0.851347	2.931978
C	4.055320	-3.468177	-1.400941	C	-4.842914	1.265791	-1.058945	H	0.520124	5.878748	-0.088924
H	5.951186	-3.118226	-0.449108	C	-4.279862	0.110422	-1.727726	C	1.810632	-3.579304	-2.431059
H	4.119042	-4.547432	-1.328520	C	-4.874660	-1.075475	-1.145332	C	0.723484	-4.052429	-2.714506
N	5.044477	-1.334494	-0.946054	C	-5.807962	-0.653233	-0.120223	C	1.887009	3.714337	-2.142101
C	-0.587146	-4.525163	-2.946798	C	-5.788291	0.794595	-0.066801	C	0.811966	4.232741	-2.390682
C	-1.154787	-5.527522	-2.057411	C	-1.975636	-2.988469	-0.352747	C	8.717041	1.769518	-0.364630
C	-1.377483	-3.822751	-3.870007	C	-2.918788	-3.463642	0.636722	C	9.705634	2.695175	-0.035301
C	-2.478498	-5.819596	-2.312792	C	-4.234751	-2.987041	0.265423	H	10.408485	3.038345	-0.784870
C	-0.657921	-6.006880	-0.792796	C	-4.104573	-2.217844	-0.954532	C	9.789304	3.176105	1.264327
C	-2.786674	-4.031880	-3.982058	C	-2.708586	-2.218308	-1.335362	C	8.879265	2.717385	2.211383
H	-0.906596	-3.011424	-4.418025	C	-4.744562	-2.639182	2.645089	H	8.907398	3.064385	3.238159
C	-3.270742	-5.092056	-3.235003	C	-5.307588	-1.482231	3.311145	C	7.920252	1.795315	1.820927
C	-3.341897	-6.351933	-1.324075	C	-6.040667	-0.711332	2.327527	H	7.186730	1.408615	2.519534
H	0.390805	-5.865974	-0.547011	C	-5.931381	-1.391228	1.053162	N	7.833603	1.331001	0.566201
C	-1.502776	-6.533192	0.168013	C	-5.129301	-2.582038	1.249419	Ru	6.451191	-0.068590	-0.096895
C	-3.798127	-3.132751	-4.487871	C	-5.269200	1.355013	3.415730	C	8.542078	1.199067	-1.708831
C	-4.619309	-5.176283	-2.819328	C	-4.674488	2.542264	2.836052	C	9.319701	1.550832	-2.810466
C	-2.928715	-6.622070	-0.033812	C	-5.059051	2.598167	1.440300	H	10.107394	2.288360	-2.714376
C	-4.662077	-5.949007	-1.635505	C	-5.892929	1.447329	1.157817	C	9.076398	0.953343	-4.039949
H	-1.084513	-6.794132	1.136689	C	-6.021936	0.678726	2.378786	C	8.055544	0.013842	-4.141027
H	-3.506737	-2.313782	-5.139808	C	-2.825111	3.462144	0.891905	H	7.828679	-0.476801	-5.080848
C	-5.113186	-3.213440	-4.071131	C	-1.894072	3.036388	-0.130992	C	7.316136	-0.291551	-3.008358
C	-5.553084	-4.199721	-3.112392	C	-2.646642	2.360846	-1.166890	H	6.508709	-1.014635	-3.033440
C	-3.992060	-6.675003	0.939536	C	-4.042633	2.369986	-0.785692	N	7.550251	0.280887	-1.818907
C	-5.640668	-5.787337	-0.673644	C	-4.153051	3.050190	0.487824	C	7.302976	-1.990046	1.942552
H	-5.811381	-2.452698	-4.409972	C	-0.787127	0.778469	-1.444033	C	8.054498	-2.961126	2.602451
C	-6.692066	-4.176147	-2.229254	C	-0.807165	-0.670300	-1.496749	H	7.762214	-3.306275	3.586811
C	-5.281939	-6.275844	0.635293	C	-2.136621	-1.083865	-1.900764	C	9.186303	-3.484888	1.992822
H	-3.762482	-6.945983	1.966566	C	-2.937423	0.106020	-2.100049	H	9.778888	-4.241119	2.496892
C	-6.732769	-4.931186	-1.069285	C	-2.104776	1.256118	-1.814754	C	9.545540	-3.023164	0.730507
H	-7.505393	-3.482508	-2.424716	C	0.652431	-0.735276	0.486373	H	10.421167	-3.403198	0.216408
H	-6.017483	-6.248787	1.434874	C	0.522496	-1.506183	1.708333	C	8.757951	-2.054343	0.128034
H	-7.577274	-4.800879	-0.397707	C	-0.310520	-2.658216	1.425213	H	8.993982	-1.662360	-0.854945
C	-0.525404	6.060683	-0.320985	C	-0.698016	-2.596749	0.031331	C	6.087223	-1.376187	2.497687
C	-1.032735	5.692880	-1.618405	C	-0.104461	-1.410920	-0.548789	C	5.533106	-1.726935	3.727316
C	-1.358480	6.528558	0.679511	C	-1.221073	-3.106944	2.378197	H	5.992089	-2.495555	4.337417
C	-2.349588	6.033293	-1.847899	C	-1.332979	-2.426918	3.652758	C	4.382388	-1.086814	4.167303
C	-0.486928	4.751876	-2.585147	C	-2.729651	-2.423448	4.037878	H	3.939543	-1.350577	5.121977
C	-2.782207	6.663915	0.487693	C	-3.481570	-3.101559	3.001732	C	3.806245	-0.110512	3.362482
H	-0.934608	6.703879	1.664887	C	-2.549540	-3.522212	1.975942	H	2.901586	0.409590	3.654218
C	-3.201193	6.505257	-0.819103	C	-3.270283	-1.312001	4.675993	C	4.399474	0.186249	2.144868
C	-3.157497	5.398124	-2.823174	C	-2.437436	-0.160966	4.957884	H	3.974766	0.928388	1.478572
C	-1.292417	4.141915	-3.559718	C	-3.238665	1.029926	4.762125	N	7.662331	-1.548057	0.711840
C	-3.843874	6.662353	1.464260	C	-4.566827	0.614567	4.360325	N	5.517530	-0.420810	1.721521
C	-4.529935	6.157165	-1.159066	C	-4.586451	-0.832623	4.307114	H	9.673996	1.219717	-4.905396
C	-2.696664	4.389728	-3.652296	C	-2.667919	2.170590	4.207379	H	10.554577	3.896817	1.532721
C	-4.503929	5.478828	-2.399934								

$(C_{60})_2$@Ru2C – v_1 E = -9514.8468374 a. u

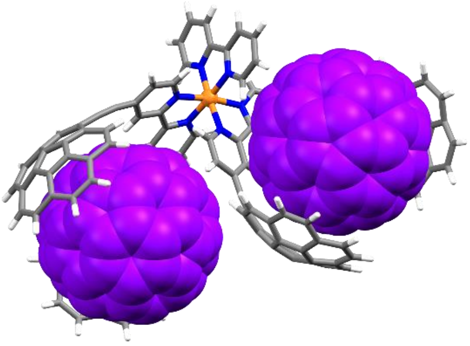

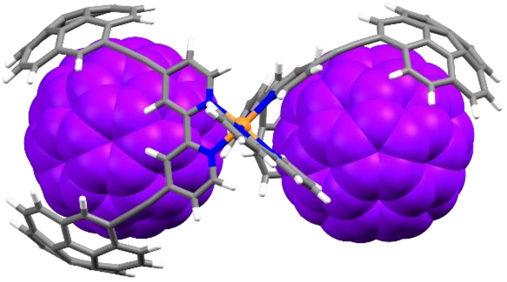

C	-1.089479	-4.792713	-3.192581	C	3.925263	-5.387951	-4.153778	C	10.027229	2.476841	-1.613276
C	-2.261022	-4.760644	-1.187316	C	0.918427	-9.811616	-1.906565	C	9.488817	2.726205	-2.935252
C	-3.432066	-4.429080	-1.850368	C	2.302764	-6.378110	-2.682725	C	8.594143	3.862097	-2.848001
C	-3.425045	-4.222247	-3.239674	C	1.547479	-7.499875	-2.104980	C	8.578318	4.314390	-1.472112
C	-2.211150	-4.436540	-3.916267	C	3.268353	-6.515378	-3.677883	C	9.464548	3.458798	-0.708917
H	-0.134926	-4.951081	-3.681954	C	1.679102	-8.824456	-2.518200	C	7.103136	2.775569	-4.472939
H	-4.352958	-4.267558	-1.305962	C	2.634066	-4.073695	-2.632418	C	5.670855	2.565995	-4.421437
H	-2.150057	-4.311660	-4.990907	C	-0.048398	-8.116240	-0.527012	C	5.106884	3.548570	-3.518566
N	-1.091364	-4.928636	-1.856481	C	3.602623	-4.143905	-3.622560	C	6.191910	4.366680	-3.011847
C	-2.170193	-4.886245	0.276435	C	0.037480	-9.451072	-0.891713	C	7.424973	3.887011	-3.601464
C	-3.240844	-4.662865	1.127387	H	3.510302	-7.493205	-4.077007	C	6.049817	0.140231	-4.547925
C	-0.765903	-5.285506	2.082407	H	2.366224	-9.085058	-3.314319	C	5.504491	-0.925057	-3.731226
C	-3.061648	-4.721273	2.519595	H	2.356117	-3.128143	-2.181255	C	4.267931	-0.449418	-3.141688
H	-4.213094	-4.385575	0.740819	H	-0.719282	-7.779138	0.255278	C	4.052772	0.911327	-3.591870
C	-1.783034	-5.064144	2.991357	H	4.093961	-3.237082	-3.956900	C	5.153659	1.274586	-4.460383
H	0.236947	-5.535176	2.411492	H	-0.577657	-10.188042	-0.387850	C	3.932000	-0.815822	-1.839107
H	-1.589770	-5.138683	4.054950	H	1.010747	-10.846180	-2.219954	C	4.818647	-1.674258	-1.076099
N	-0.935851	-5.186462	0.754478	H	4.680896	-5.481346	-4.926743	C	4.805050	-1.217519	0.299198
C	-6.207649	-3.383381	4.641636	H	5.624023	6.468209	3.572626	C	3.911443	-0.080784	0.386393
C	-5.984595	-2.361753	5.652898	H	12.088963	2.609014	3.326138	C	3.369091	0.167067	-0.934463
C	-7.484837	-3.638460	4.120318	C	2.895262	7.031245	-0.101406	C	5.977130	-1.237169	1.050774
C	-7.142537	-1.802072	6.152430	C	12.285548	-2.224483	0.088575	C	7.209203	-1.713711	0.459306
C	-4.776490	-1.646573	5.977641	C	10.027425	1.131385	4.778211	C	8.292980	-0.899071	0.969274
C	-8.640579	-2.906200	4.538841	H	13.313082	1.683318	1.456801	C	7.731159	0.080544	1.873735
H	-7.557537	-4.334614	3.289413	H	4.200758	7.541279	-3.166691	C	6.298492	-0.128910	1.926999
C	-8.424826	-2.057826	5.610609	C	4.446364	7.006372	1.804859	C	9.347672	-0.550559	0.135469
C	-7.171220	-0.518005	6.749258	C	11.216277	-0.622934	3.699839	C	9.364086	-1.004962	-1.240550
H	-3.818704	-2.055975	5.669137	H	2.354346	6.108287	-3.794312	C	9.912657	0.059847	-2.055120
C	-4.807382	-0.393786	6.565544	H	8.491923	-4.762104	1.218260	C	10.235857	1.170866	-1.181479
C	-9.888147	-2.693774	3.842010	C	5.694683	-4.239825	2.828791	C	9.885897	0.792152	0.171873
C	-9.244590	-0.936357	5.871956	C	3.823492	5.337681	3.516592	C	9.396455	0.300413	-3.324282
C	-6.047265	0.274708	6.878263	H	5.873367	8.293319	-1.047488	C	8.311456	-0.515127	-3.830000
C	-8.468199	0.016991	6.569840	C	12.030584	1.538436	3.149047	C	7.425471	0.341430	-4.591985
H	-3.867636	0.134985	6.701904	C	1.107396	3.326765	1.734306	C	7.963307	1.685691	-4.557172
H	-10.168577	-3.362350	3.032576	C	12.732216	1.007609	2.079139	C	9.181235	1.660582	-3.773979
C	-10.684267	-1.592367	4.097169	C	1.818856	6.188651	-0.466642	C	7.789520	-1.541996	-3.049660
C	-10.318756	-0.592970	5.072722	C	10.360161	-3.884307	0.686943	C	8.327340	-1.794127	-1.728319
C	-6.320396	1.683024	7.033362	C	0.893062	4.172267	0.570257	C	7.225407	-2.156790	-0.858563
C	-8.714939	1.374918	6.501423	H	2.130066	2.996560	3.578089	C	6.007069	-2.129539	-1.643229
H	-11.562241	-1.434537	3.476385	C	7.885257	-1.983515	4.418201	C	6.356064	-1.748790	-2.997312
C	-10.717257	0.789075	5.170093	C	11.392153	-2.920415	0.982739	C	-5.150048	2.971354	3.504321
C	-7.589043	2.206314	6.851629	C	1.454915	5.428457	0.672788	C	-4.109459	3.544603	2.674234
H	-5.496597	2.370789	7.204230	C	8.997282	-1.107463	4.627441	C	-4.707848	4.582342	1.859414
C	-9.953497	1.724619	5.849026	C	2.303772	5.811738	1.740699	C	-6.117666	4.650807	2.186148
H	-11.591562	1.132924	4.623955	H	6.921706	-1.750543	4.862809	C	-6.391987	3.656058	3.202802
H	-7.713380	3.285373	6.887492	C	9.150675	-3.376914	2.777437	C	-3.585826	3.652814	-1.394435
H	-10.258086	2.767154	5.808128	C	9.007813	0.255912	5.103713	C	-4.826416	4.337088	-1.695368
C	-5.522283	-0.628598	-5.897878	H	4.105082	4.774443	4.402124	C	-5.260112	5.026000	-0.496535
C	-6.687370	-1.403673	-5.554927	C	2.625152	4.974174	2.796125	C	-4.288096	4.766906	0.545656
C	-5.616874	0.699806	-6.274590	H	-0.029926	2.847544	-0.906771	C	-3.253847	3.917859	-0.008379
C	-7.893537	-0.791636	-5.824503	C	0.777397	1.959093	1.616058	C	-7.590166	4.469467	-1.056001
C	-6.794266	-2.571542	-4.692775	C	11.932603	-1.164715	2.608395	C	-8.630961	3.896492	-0.226984
C	-6.883114	1.388275	-6.340433	H	12.439449	-2.607281	-0.916792	C	-8.297288	4.161643	1.157803
H	-4.697721	1.258628	-6.430080	C	0.827575	4.545009	-1.867746	C	-7.050922	4.898727	1.184734
C	-7.986951	0.567912	-6.208671	C	5.379907	7.662758	0.924480	C	-6.613454	5.088785	-0.183172
C	-9.104056	-1.144735	-5.179566	C	12.867416	-1.015196	0.429751	C	-9.168178	1.942788	1.763461
C	-8.001159	-2.918681	-4.066821	C	5.094273	7.878770	-0.413196	C	-8.569390	0.903959	2.575651
C	-7.169154	2.799396	-6.248635	C	3.189603	6.804624	1.263492	C	-7.593248	1.523197	3.447045
C	-9.252083	1.050398	-5.799621	H	13.457191	-0.495092	-0.320490	C	-7.588696	2.946889	3.176002
C	-9.195219	-2.147510	-4.229889	H	10.363906	-4.390969	-0.274594	C	-8.561539	3.206096	2.133829
C	-9.944360	-0.009076	-5.169131	H	9.935624	2.169632	5.086103	C	-6.139118	-0.433264	3.161661
H	-7.978678	-3.736809	-3.352363	C	9.291297	-4.100404	1.539318	C	-4.729367	-0.504460	2.837389
H	-6.368896	3.517551	-6.406531	C	3.536780	7.180057	-2.385867	C	-4.121230	0.756994	3.210336
C	-8.408295	3.270370	-5.848514	C	6.735667	-3.727022	3.202270	C	-5.156914	1.605408	3.764346
C	-9.488039	2.378773	-5.501690	C	4.689540	6.304591	3.042463	C	-6.402954	0.867431	3.735660
C	-10.350347	-2.061692	-3.367340	H	8.150784	0.636448	5.652826	C	-2.693354	0.619040	1.217761

C	-10.914233	0.194959	-4.205374	C	10.246821	-2.620560	3.136372	C	-2.425799	1.617046	0.203377
H	-8.534228	4.340773	-5.708640	C	1.922856	3.729107	2.802888	C	-2.678527	2.923124	0.775578
C	-10.642539	2.607102	-4.667697	C	1.630899	5.738334	-1.759938	C	-3.111752	2.732074	2.144324
C	-11.165548	-0.945102	-3.355366	H	6.371332	7.916034	1.290255	C	-3.120253	1.308572	2.417318
H	-10.534463	-2.848865	-2.641237	C	12.617256	-0.379533	1.700504	C	-3.343231	0.095880	-1.499414
C	-11.321048	1.569254	-4.050352	H	0.567628	4.162780	-2.851565	C	-4.312608	0.355321	-2.544313
H	-10.940288	3.626755	-4.437712	C	0.483686	3.792900	-0.759105	C	-4.305646	1.779240	-2.820442
H	-11.963740	-0.895145	-2.619506	C	7.944572	-3.071299	3.533410	C	-3.331181	2.401000	-1.947193
H	-12.125585	1.814078	-3.362076	C	2.480439	6.360944	-2.744936	C	-2.738756	1.361591	-1.129283
H	-4.536686	-1.065684	-5.765558	C	3.846487	7.463577	-1.006424	C	-5.499423	2.435585	-3.103780
C	-4.131320	-4.385477	3.374091	C	11.337882	-2.400351	2.261304	C	-6.746382	1.697647	-3.127001
C	-5.096974	-3.965899	3.986733	C	11.142686	0.734427	3.950942	C	-7.780848	2.546316	-2.575690
C	-4.590730	-3.759216	-3.882410	C	0.628280	0.781154	1.343573	C	-7.174722	3.809061	-2.207762
C	-5.619341	-3.248396	-4.289192	C	10.172288	-1.518837	4.022831	C	-5.763714	3.740757	-2.533562
Ru	0.587477	-5.143836	-0.660407	C	7.394791	4.776046	-0.904896	C	-8.780008	1.995972	-1.779964
N	2.173877	-5.072829	0.683944	C	6.177104	4.798808	-1.689960	C	-8.785969	0.574431	-1.506080
N	1.995531	-5.158891	-2.173396	C	5.076622	4.433972	-0.820347	C	-9.224663	0.382738	-0.137990
N	0.650957	-3.125216	-0.200588	C	5.613987	4.185161	0.500245	C	-9.489583	1.687950	0.433760
C	4.476261	-4.587415	2.209429	C	7.046199	4.396119	0.449961	C	-9.214589	2.684680	-0.581997
C	0.601691	-0.541327	0.866757	C	3.513001	1.854275	-2.721052	C	-8.653696	-0.616567	0.643954
C	2.371213	-3.862044	1.266306	C	3.167505	1.474500	-1.365598	C	-7.616704	-1.465437	0.088861
C	1.426947	-2.808655	0.866629	C	3.494101	2.585227	-0.494321	C	-6.643070	-1.724092	1.131274
C	3.483894	-3.606472	2.051157	C	4.037064	3.650727	-1.307359	C	-7.076652	-1.030503	2.326950
C	1.385984	-1.551055	1.446952	C	4.050454	3.199705	-2.684352	C	-8.317348	-0.348481	2.026648
C	3.081098	-6.039938	0.895419	C	4.221603	0.985965	1.225224	C	-5.286989	-1.796528	0.820625
C	-0.098211	-2.164557	-0.760671	C	5.437579	0.961224	2.012973	C	-4.849423	-1.603466	-0.549163
C	4.234737	-5.845954	1.631635	C	5.973908	2.308636	2.050937	C	-3.601580	-0.865211	-0.523171
C	-0.126754	-0.870638	-0.287458	C	5.090427	3.162390	1.283668	C	-3.271240	-0.597327	0.862867
H	3.650829	-2.621749	2.466413	C	4.011095	2.345657	0.773221	C	-4.309329	-1.174470	1.692989
H	1.985781	-1.322408	2.318361	C	8.245766	1.371531	1.908709	C	-5.785304	-1.346474	-1.547864
H	2.871555	-6.995511	0.426937	C	9.345523	1.734760	1.040610	C	-7.196016	-1.279777	-1.224228
H	-0.690507	-2.453038	-1.621131	C	9.130701	3.095313	0.591729	C	-7.792407	-0.239217	-2.037503
H	4.955968	-6.647930	1.735709	C	7.897009	3.574405	1.183472	C	-6.752706	0.333621	-2.863991
H	-0.713917	-0.120002	-0.799221	C	7.349880	2.507777	1.998275	C	-5.511730	-0.348075	-2.560378
N	0.686933	-7.160850	-1.112469								

$(C_{60})_2$@Ru2C – v_2 E = - 9514.8439063 a. u

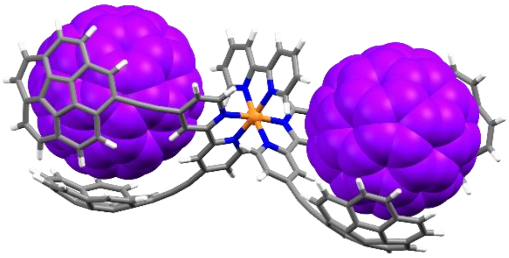

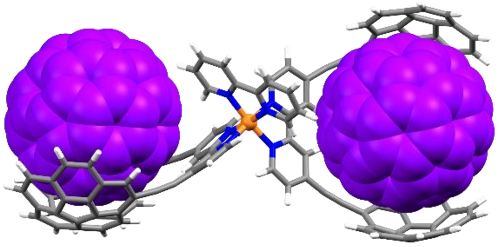

C	-1.380346	1.238753	-2.522840
C	-2.328872	1.109198	-0.406624
C	-3.364086	1.948161	-0.792307
C	-3.435750	2.418118	-2.114030
C	-2.390039	2.059040	-2.983668
H	-0.565802	0.934407	-3.170691
H	-4.159860	2.199703	-0.103419
H	-2.388358	2.401938	-4.011466
N	-1.344913	0.753608	-1.271292
C	-2.254269	0.470380	0.916779
C	-3.174006	0.698048	1.926758
C	-1.158099	-1.110834	2.219601
C	-3.116479	-0.046471	3.117392
H	-3.978877	1.408478	1.796122
C	-2.061485	-0.964461	3.254339
H	-0.331641	-1.809200	2.290582
H	-1.961213	-1.559465	4.154234
N	-1.249514	-0.430515	1.065964
C	-6.410501	0.265804	5.381630
C	-7.201387	1.465876	5.162338
C	-6.968462	-0.897647	5.933589
C	-8.479587	1.400404	5.675525
C	-6.961626	2.565873	4.263446
C	-8.356492	-0.987694	6.271458
H	-6.345067	-1.786849	5.965230
C	-9.044348	0.210944	6.196371
C	-9.535128	2.206069	5.185025
H	-5.959145	2.725721	3.876906
C	-7.992926	3.360948	3.795869
C	-9.194968	-2.159373	6.378472
C	-10.444942	0.277875	6.022683
C	-9.366257	3.124492	4.167204
C	-10.747723	1.507026	5.392122
H	-7.762766	4.121605	3.053970
H	-8.735694	-3.134905	6.512457
C	-10.564070	-2.091371	6.196736
C	-11.234735	-0.849566	5.893368
C	-10.579385	3.477489	3.472952
C	-11.856854	1.678516	4.586200
H	-11.131482	-3.018207	6.192392
C	-12.498918	-0.620615	5.238418
C	-11.762892	2.787106	3.667500
H	-10.547089	4.250844	2.710721
C	-12.793700	0.581304	4.616340
H	-13.209531	-1.437793	5.148620
H	-12.617584	3.036461	3.043860
H	-13.725168	0.660287	4.062049
C	-7.822012	4.751928	-1.144768
C	-7.892046	4.514818	-2.564285
C	-8.961728	4.914380	-0.376664
C	-9.147103	4.667226	-3.117612
C	-6.930503	3.870088	-3.445111
C	-10.285469	4.846078	-0.946145
H	-8.847867	5.001795	0.700808
C	-10.311422	4.822034	-2.326927
C	-9.531282	4.068407	-4.342200
C	-7.315398	3.313667	-4.676594
C	-11.543797	4.523930	-0.318816
C	-11.407903	4.310033	-3.060991
C	-8.675330	3.307326	-5.118123
C	-10.926343	3.848495	-4.306737
H	-6.564345	2.756779	-5.229775
H	-11.636976	4.592375	0.761091
C	-12.613469	4.017502	-1.036839
C	-12.536839	3.788842	-2.458408
C	-9.329822	2.409014	-6.039986
C	-11.542488	2.839493	-5.023576
H	-13.501026	3.705322	-0.492768
C	-13.304793	2.898111	-3.293781
C	-10.693483	2.184697	-5.990540
H	-8.730572	1.821491	-6.730117
C	-12.832020	2.445817	-4.514277
H	-14.242829	2.494793	-2.921562
H	-11.117260	1.427909	-6.645340
H	-13.415825	1.704692	-5.053456
H	-6.857463	4.711645	-0.646931
C	-4.140890	0.093985	4.072153
C	-5.150261	0.170677	4.750383
C	-4.566703	3.124694	-2.567422
C	-5.639228	3.536696	-2.975474
Ru	0.036035	-0.613249	-0.545923
N	1.423700	-1.850633	0.372124
N	1.150578	-0.819557	-2.283696
N	1.264130	0.767200	0.388319
C	3.549145	-3.301102	1.479151
C	3.041666	2.460311	1.738143
C	2.370784	-1.219914	1.112620
C	2.254996	0.246777	1.157058
C	3.416390	-1.919506	1.695671
C	3.130224	1.062700	1.856428
C	1.512774	-3.180294	0.208205
C	1.143309	2.101541	0.307176
C	2.545099	-3.935848	0.727514
C	2.002499	2.975228	0.944410
H	4.179479	-1.405029	2.263632
H	3.923494	0.640646	2.458590
H	0.725209	-3.637840	-0.380119
H	0.330545	2.464880	-0.311640
H	2.589147	-5.002240	0.540439
H	1.880347	4.045119	0.824141
N	-1.018359	-2.099849	-1.540428
C	2.554629	-1.314248	-4.628080
C	-2.311672	-4.035012	-3.056360
C	0.706647	-1.759064	-3.156015
C	-0.524058	-2.455767	-2.753036
C	1.396455	-2.022812	-4.337865
C	-1.159526	-3.421858	-3.530583
C	2.272570	-0.142508	-2.565431
C	-2.135399	-2.688386	-1.090924
C	3.000817	-0.355481	-3.725997
C	-2.808156	-3.663362	-1.812636
H	1.037198	-2.779239	-5.025192
H	-0.761318	-3.693694	-4.500740
H	2.590583	0.586313	-1.828700
H	-2.494596	-2.357329	-0.123237
H	3.903799	0.216854	-3.903861
H	-3.707279	-4.108578	-1.402137
H	-2.816980	-4.787961	-3.651995
H	3.103972	-1.512074	-5.542528
H	11.538807	3.130485	4.891810
H	14.043131	-3.566862	2.195419
C	10.158952	6.411616	1.960859
C	10.684842	-6.182908	-1.774355
C	11.604259	-3.651609	3.802346
H	14.195294	-4.499721	-0.031004
H	11.647174	6.816667	-1.035254
C	11.157445	4.802303	3.527160
C	11.193024	-5.362753	2.203328
H	9.362620	7.246073	-1.706039
H	6.250430	-5.778651	-0.268984
C	4.702701	-3.979277	1.920772
C	9.458648	3.568306	4.831189
H	13.253259	5.586561	0.914404
C	13.251342	-4.229887	1.857263
C	6.205010	4.543560	3.067897
C	13.338440	-4.765072	0.582727
C	8.822151	6.656436	1.568193
C	8.177122	-6.249554	-1.048851
C	6.704703	5.593544	2.194423
H	6.609192	3.098284	4.585788
C	7.888761	-4.318793	3.408334
C	9.601443	-6.332763	-0.833370
C	7.969407	6.041179	2.517136
C	9.304171	-4.501098	3.511166
C	8.783880	5.424207	3.497448
H	7.379936	-3.639277	4.086343
C	7.756670	-5.672733	1.313612
C	10.279138	-3.685005	4.196096
H	9.226667	2.735663	5.489676
C	8.380807	4.328398	4.241012
H	5.220306	5.735440	0.594072
C	4.994112	3.896656	2.729789
C	11.282212	-5.909318	0.902797
H	10.483557	-6.271995	-2.838558
C	7.063553	6.606897	-0.027422
C	12.340719	4.895540	2.708803
C	11.957079	-5.812288	-1.372817
C	12.361748	5.629938	1.534529
C	10.135614	5.656757	3.156281
H	12.706748	-5.625385	-2.137164
H	7.784453	-6.341913	-2.058094
H	12.271942	-2.948518	4.293319
C	7.298594	-5.931277	-0.028243
C	10.868596	6.772819	-0.278327
C	5.808101	-4.423776	2.177397
C	10.778869	3.795355	4.489803

$(C_{60})_3$@Ru3C – v_1v_1 E = -13483.195143 a. u

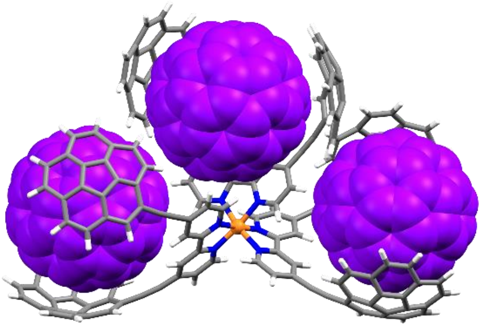

C	1.221532	-4.627237	1.569049
C	2.440442	-4.644184	-0.41064
C	3.493215	-5.299968	0.207531
C	3.423847	-5.601053	1.575008
C	2.236463	-5.26965	2.253377
H	0.300496	-4.339364	2.064144
H	4.386896	-5.558143	-0.345192
H	2.126986	-5.499306	3.306691
N	1.31694	-4.296597	0.271759
C	2.451234	-4.241556	-1.828979
C	3.592123	-4.209235	-2.62018
C	1.143905	-3.470006	-3.589516
C	3.502505	-3.744221	-3.94007
H	4.565944	-4.439696	-2.201288
C	2.22516	-3.448814	-4.446478
H	0.158477	-3.154582	-3.914376
H	2.10357	-3.133644	-5.476214
N	1.255499	-3.801128	-2.29454
C	6.768796	-2.33794	-5.809453
C	6.741338	-0.883874	-5.795836
C	7.911275	-3.051281	-6.181703
C	7.852281	-0.289748	-6.354297
C	5.893791	-0.005169	-5.034749
C	9.12629	-2.386195	-6.555746
H	7.89711	-4.131456	-6.067566
C	9.012844	-1.016029	-6.717945
C	8.24434	1.032572	-6.030846
H	4.977103	-0.39400	-4.599734
C	6.274705	1.28876	-4.727398
C	10.479801	-2.881438	-6.502177
C	10.121941	-0.143334	-6.621791
C	7.544456	1.828967	-5.141329
C	9.64764	1.118218	-6.188886
H	5.636362	1.860716	-4.060155
H	10.654036	-3.951999	-6.439162
C	11.56248	-2.025528	-6.404907
C	11.401415	-0.59284	-6.35237
C	8.310657	2.898361	-4.55307
C	10.426889	2.000018	-5.462874
H	12.551483	-2.455168	-6.269768
C	12.283524	0.402118	-5.79604
C	9.685002	2.977998	-4.706011
H	7.821775	3.599715	-3.879966
C	11.819394	1.635495	-5.371644
H	13.324254	0.148702	-5.612551
H	10.222195	3.744257	-4.153386
H	12.513362	2.305125	-4.870625
C	6.35349	-4.826531	5.213933
C	7.030465	-5.991837	4.705837
C	6.989189	-3.922236	6.04526
C	8.263577	-6.240982	5.269124
C	6.761356	-6.760007	3.496815
C	8.366218	-4.086103	6.443613
H	6.460321	-3.013108	6.319563
C	8.913688	-5.311233	6.115908
C	9.257511	-7.028479	4.638705
C	7.745124	-7.544617	2.886152
C	9.322602	-3.095498	6.871442
C	10.307949	-5.523689	6.005462
C	9.077036	-7.628979	3.404776
C	10.520532	-6.587927	5.097334
H	7.507497	-7.996777	1.927175
H	8.97142	-2.122523	7.205085
C	10.68668	-3.301978	6.758889
C	11.233105	-4.518279	6.208419
C	10.300116	-7.968169	2.713822
C	11.671865	-6.710848	4.342565
H	11.356035	-2.482363	7.006482
N	-1.005661	-1.772573	-2.029802
N	-1.163836	-2.387482	0.745753
N	1.280201	-1.483493	-0.792313
C	-2.345853	0.346227	-3.268028
C	3.094154	0.586595	-1.239965
C	-0.355106	-0.603618	-2.270191
C	0.992292	-0.509789	-1.687642
C	-0.992511	0.459356	-2.869801
C	1.895677	0.504738	-1.968412
C	-2.263306	-1.917169	-2.464582
C	2.395293	-1.379943	-0.054216
C	-2.969057	-0.903295	-3.095013
C	3.294617	-0.350691	-0.211777
H	-0.535612	1.440038	-2.889003
H	1.671481	1.240579	-2.728763
H	-2.736395	-2.869733	-2.254742
H	2.559907	-2.156406	0.683956
H	-4.001266	-1.087551	-3.381444
H	4.162909	-0.284788	0.427866
N	-1.483591	-4.537475	-0.723713
C	-3.008212	-1.444438	2.620608
C	-3.715466	-6.222766	-0.583107
C	-2.274238	-3.112217	1.029909
C	-2.444411	-4.325615	0.212818
C	-3.193576	-2.67371	1.96909
C	-3.54768	-5.157442	0.317257
C	-0.978484	-1.210349	1.359982
C	-1.604756	-5.592233	-1.547057
C	-1.863521	-0.701876	2.289411
C	-2.691255	-6.443547	-1.522631
H	-4.09142	-3.235548	2.172183
H	-4.32161	-4.963398	1.05004
H	-0.088782	-0.660188	1.076319
H	-0.794262	-5.724462	-2.25557
H	-1.681223	0.266907	2.739581
H	-2.759186	-7.265475	-2.225528
H	4.634311	9.389158	-2.126658
H	-4.087134	10.843079	-3.19625
C	6.509605	7.243236	1.487636
C	0.878773	8.879067	0.060091
C	-3.589007	6.004879	2.934548
C	0.802752	2.551525	1.502947
C	-7.033377	6.757236	-0.306141
C	-3.321136	8.586466	-4.87961
H	-5.600138	10.638965	-1.319367
C	-1.536504	8.811214	-0.400734
H	5.497895	7.955191	4.622458
C	5.643469	8.64374	-0.331908
C	1.873568	3.246035	0.814497
C	-5.315312	7.776279	-3.868116
H	5.761557	5.560449	4.864789
C	-0.850115	2.974888	3.275177
C	2.621941	4.210507	1.48003
H	-5.399459	2.633187	-1.874472
C	-3.055322	1.497563	-3.688554
C	5.191702	7.354004	-2.392229
C	-0.816198	3.600443	-1.494214
H	5.087323	10.015714	2.77271
C	-4.670917	9.931797	-3.097648
C	-3.469036	3.897735	1.035706
C	2.518442	5.947986	3.048345
C	5.522248	3.756465	-1.190197
C	-3.10391	4.699647	3.331715
C	2.030339	8.001363	0.030684
C	2.978507	5.44483	0.810031
C	2.57623	5.66744	-0.501744
C	-5.537042	9.814663	-2.02483
C	-3.635014	7.461907	0.951284
H	4.79903	10.517749	0.422535
C	1.177787	7.959959	3.49097
C	-2.882338	8.420642	1.619478
C	-6.262308	8.59627	-1.760797
H	5.83298	3.136268	3.458074
C	-0.914092	4.049455	4.245208
C	5.921786	3.200423	1.333782
C	-2.32081	7.812738	-1.098395
C	-4.129893	3.866545	-4.113797
C	5.985147	6.012397	3.902058
C	5.992001	8.040705	2.491342
C	-6.447169	6.276875	-2.555308
C	-4.44744	8.842868	-4.015257
C	4.041486	1.609168	-1.4298
C	-5.012266	6.47359	-4.329447
C	-3.047789	3.664432	2.403255
C	-0.285751	2.355637	0.564749
C	-9.686894	1.736054	5.493169
C	-9.832198	1.523268	0.64748
H	-12.273925	-7.526312	2.552441
C	-11.207332	-4.726156	-1.257167
C	-8.253675	-4.75984	0.938397
C	-13.659349	-7.180737	-1.469719
C	-7.800928	0.664118	1.43551
C	-8.042527	-8.144727	-1.742678
C	-7.410857	-2.800894	2.171123
C	-10.64741	-0.367987	6.051428
C	-7.694857	-7.664501	-3.05634
C	-7.152632	-0.495464	1.841825
C	-11.979502	1.546281	4.889204
C	-10.592924	3.298253	3.835994
H	-10.920417	4.519217	2.052561
C	-9.575171	-2.238939	-4.081262
C	-7.647759	-4.725197	-1.445885
C	-4.90257	-6.983548	-0.557064
C	-6.291957	1.504485	4.426227
C	-10.840006	0.97418	5.560828
C	-10.529891	1.219875	-2.090001
H	-6.661594	-1.698251	5.683584
C	-12.747578	-2.863804	-0.812521
H	-4.749531	2.023201	2.957729
C	-9.073667	0.110709	-3.553872
C	-9.567316	2.870293	4.657718
C	-8.272917	0.887122	-2.630173
C	-6.903886	-0.6503	5.532354
C	-3.976137	-0.944628	3.517488
C	-6.427074	3.32252	2.764708
C	-11.591399	-8.242971	-1.948537
C	-10.467943	0.317945	-3.220914
C	-10.509833	-4.427589	1.480017
C	-9.953499	-5.260302	-0.767345
H	-8.345897	-6.982239	-4.963391
H	-6.666334	-7.382148	-3.262169
C	-8.899467	-3.448459	-3.655577
C	-10.984328	-4.202598	-2.58807
C	-9.70916	-3.651792	2.405192
C	-10.304716	-8.038412	1.934823
H	-9.303112	-1.956514	6.495429
C	-7.576958	1.190135	0.103902
C	-12.367922	-0.934905	-2.289735
C	-7.868038	-1.497266	2.601676
C	-9.351008	-8.556488	-1.601602
C	-5.720113	2.293533	3.363708
C	-7.381989	-1.670944	-3.486129
C	-11.367769	-0.73772	-3.318407
H	-11.511011	-1.010904	6.199848
C	-8.835512	4.262317	2.399476
C	-11.134211	1.186043	0.29624

C	12.516535	-4.752804	5.59253	C	6.650907	5.842501	1.625475	C	-6.928795	-3.724291	-2.207585
C	11.532649	-7.528734	3.160468	C	-1.430626	7.075629	-1.974609	C	-9.193083	0.872297	1.773084
H	10.248489	-8.499804	1.767676	C	0.827025	5.350021	-2.027359	C	-6.250381	-1.175274	0.938301
C	12.725825	-5.796221	4.706653	C	-6.479594	4.346961	-1.157869	C	-11.238649	-0.443251	2.13617
H	13.322402	-4.04118	5.752189	C	6.074043	3.988284	0.136879	C	-7.337733	-7.974887	-0.48016
H	12.407002	-7.731523	2.547692	H	4.871458	4.569256	-3.055336	C	-11.214487	-7.064437	-4.409785
H	13.687019	-5.863683	4.204312	C	2.336538	4.521858	2.865394	C	-7.780501	3.640754	3.159039
C	11.871723	-4.181857	1.796282	C	-3.444407	4.793427	-4.910957	C	-5.975354	-1.577276	-1.469554
C	11.909522	-4.675082	0.435282	C	-1.607988	2.470483	0.988436	C	-13.464106	-7.387912	-0.11436
C	12.73978	-3.775969	-0.340736	C	-0.556652	6.843985	4.604826	C	-12.65087	-0.593781	0.128889
C	13.217597	-2.730886	0.541608	C	-6.763629	5.757526	-1.313568	C	-7.065037	0.378652	-2.160468
C	12.681701	-2.982323	1.863518	C	0.418556	4.595023	4.40123	H	-9.908683	-7.860482	2.930988
C	10.600205	-3.057401	-3.09816	C	6.553931	5.266299	0.33442	C	-4.885158	-0.494811	4.189735
C	11.408932	-1.85719	-3.033541	C	-3.823437	6.170701	-4.972697	C	-9.881267	-0.091702	2.500947
C	12.51978	-2.114366	-2.140612	C	-1.89618	2.783916	2.374214	C	-7.994851	-8.078117	0.754606
C	12.398421	-3.473614	-1.65459	C	6.372338	6.316071	-0.600326	H	-12.940932	1.043345	4.946444
C	11.211142	-4.056123	-2.245558	H	-2.535634	4.456168	-5.400362	C	-10.17126	4.103806	2.721588
C	12.343598	0.191211	-1.304611	C	-5.267991	4.260202	-3.298768	C	-9.386166	-0.908528	6.22061
C	12.306509	0.682621	0.057303	C	-2.460049	8.188327	2.985886	C	-8.395419	1.186694	5.664278
C	12.917739	-0.317069	0.909468	C	-1.128717	8.735281	3.141222	C	-9.173109	1.571747	-1.723708
C	13.333198	-1.425267	0.074256	C	2.092666	6.970836	-0.902016	C	-11.657187	-7.849252	1.717932
C	12.979069	-1.110975	-1.294305	C	-0.727551	9.304633	1.871134	C	-12.240476	-7.948177	0.401718
C	11.253474	0.189376	2.647125	C	-3.92913	5.155311	0.653657	C	-8.199657	3.036419	4.329572
C	10.422861	-0.707364	3.424348	C	0.593434	5.96456	4.575988	C	-12.532847	-2.359697	-2.091609
C	11.057571	-2.009027	3.432321	C	-3.024259	7.316526	-5.336608	C	-6.614339	-0.926155	-2.595715
C	12.282506	-1.917206	2.665386	H	4.717951	7.313227	-3.369268	C	-11.860431	2.650022	4.063842
C	12.40331	-0.558174	2.178827	C	-3.996282	6.228402	1.623576	C	-12.170925	-2.614084	1.445629
C	8.841046	-3.056801	3.289501	C	-2.580356	3.160974	0.160253	C	-7.543135	-3.097244	-3.287525
C	8.362961	-4.10191	2.409153	C	5.731224	6.145454	-1.815962	C	-7.472369	1.991946	4.950762
C	9.510707	-4.848903	1.939569	C	2.919315	6.515903	1.778593	C	-12.808648	-1.962618	0.319802
C	10.69624	-4.268144	2.533009	H	5.622278	2.160728	1.249835	C	-12.426392	-0.069804	-1.202557
C	10.282042	-3.16044	3.366522	C	4.806732	2.555917	-1.399423	C	-6.012947	-7.478592	-0.483343
C	8.433365	-5.073725	-0.257448	C	-6.198912	7.654552	-2.770357	C	-9.588841	-4.406618	-2.920443
C	8.971341	-4.818172	-1.578218	H	-7.328082	6.452024	0.692762	H	-11.10204	-6.577728	-5.37491
C	10.414691	-4.921704	-1.501865	C	6.041956	3.759621	2.592562	C	-10.061074	-7.649159	-3.772618
C	10.770575	-5.238023	-0.133784	C	-0.453412	4.825335	-2.178744	H	-5.970556	3.83094	1.913816
C	9.546012	-5.329444	0.635342	C	-0.162384	8.693369	-0.844161	C	-6.12782	-2.948782	-1.282073
C	7.236662	-3.109632	-1.928731	C	5.29344	9.589857	0.697234	C	-9.611151	-5.108092	0.571738
C	7.358686	-1.750136	-2.413954	C	-3.348934	7.151598	-0.434333	H	-8.595229	4.777841	1.479123
C	8.583032	-1.656842	-3.180158	C	-6.801489	8.104357	-0.520261	C	-8.189937	-0.15742	5.918503
C	9.215711	-2.959944	-3.173995	C	1.009586	6.776709	-1.844108	C	-6.026056	-0.669267	-0.338327
C	8.384344	-3.858984	-2.400023	C	-2.018059	4.894651	4.270705	C	-10.144851	-2.398678	2.821804
C	9.356957	-0.504017	-3.123852	C	5.457277	9.301954	2.041202	C	-8.656116	-7.433621	-4.024568
C	8.943395	0.600311	-2.282946	C	6.342005	7.536705	0.113509	H	-7.425145	-7.833528	1.646665
C	10.128993	1.18495	-1.691953	H	-6.935642	8.791341	0.311198	C	-6.704024	0.542019	-0.767455
C	11.277305	0.437767	-2.162732	C	-0.096547	7.620044	-1.815362	H	-13.251748	-6.463199	-4.275883
C	10.80003	-0.608547	-3.042397	H	-6.72653	3.851891	-0.223084	C	-11.851466	0.181288	1.055258
C	10.093575	1.65953	-0.384983	C	-1.836277	6.319751	4.456078	C	-11.716155	-3.918917	1.009184
C	8.86892	1.5677	0.38465	H	-2.631661	9.394101	-5.11124	C	-7.292719	-4.571903	-0.050854
C	9.223354	1.251446	1.753746	C	-0.195652	8.07692	3.935152	C	-9.20564	-1.30073	2.921689
C	10.666856	1.149864	1.829649	C	2.450864	7.768409	1.396847	C	-5.996675	0.128906	4.804362
C	11.204327	1.401878	0.508198	C	1.309271	3.857728	3.528845	C	-11.402445	-1.869058	2.335345
C	8.424639	0.392561	2.503859	C	1.794808	4.665398	-1.196445	H	-14.221376	-7.029138	0.577567
C	7.240717	-0.194712	1.908825	C	0.524245	2.856046	2.832829	C	-10.008094	-8.642374	-0.349932
C	7.117036	-1.554809	2.39512	C	-1.811962	9.110057	0.930023	C	-10.333288	-8.309384	-2.590383
C	8.229454	-1.808737	3.286628	C	-5.755064	3.635737	-2.096812	C	-8.313445	-3.856022	2.070476
C	9.03662	-0.608003	3.354378	C	5.834812	7.381212	3.763355	C	-12.072647	-4.072867	-0.38725
C	6.655174	-2.56138	1.548537	C	-3.578223	2.573186	-3.924419	C	-11.632811	-3.043014	-2.997346
C	6.304481	-2.248548	0.178201	C	-3.527567	5.726537	-0.616369	H	-14.562961	-6.667109	-1.78727
C	6.714929	-3.358038	-0.659732	C	1.563907	8.507061	2.271318	C	-11.487993	1.0281	-1.100142
C	7.326178	-4.359568	0.194589	C	-2.674609	5.021004	-1.455069	C	-11.391676	-8.453284	-0.56549
C	7.289864	-3.863257	1.556714	C	5.143935	8.539037	-1.681175	C	-12.652658	-7.513254	-2.448284
C	6.434782	-0.943257	-0.290547	C	0.114899	2.937929	-0.700181	C	-10.913016	-2.040637	-3.756911
C	6.904055	0.105953	0.591843	C	-1.605412	5.706143	-2.1514	H	-12.733989	2.974536	3.504484
C	7.731603	1.006282	-0.186501	H	-2.11267	7.17135	-5.909856	C	-12.446298	-6.997537	-3.779117
C	7.766231	0.517213	-1.549098	C	0.591356	9.193466	1.445115	C	-8.952182	-5.054407	-1.793474
C	6.961878	-0.686036	-1.612973	C	-2.189061	3.7197	-1.052113	C	-9.396461	-8.341677	0.853508
H	5.351335	-4.599295	4.860937	C	-5.713523	5.547	-3.522787	C	-6.412169	-2.602654	1.139895
C	4.660295	-3.440146	-4.6916	C	1.665206	6.654626	3.887584	C	-8.83296	1.721308	-0.383691
C	5.644708	-2.998277	-5.252645	C	-2.806999	7.006028	3.630204	C	-8.636684	-1.140345	-3.978116
C	4.529978	-6.151757	2.253314	C	5.395097	4.791761	-2.129701	C	-6.353526	-3.473326	0.051648
C	5.548562	-6.508089	2.815255	C	6.298067	5.166626	2.778616				
Ru	-0.000372	-3.100012	-0.796431	C	1.447448	3.483445	-0.548485				

$(C_{60})_3$@Ru3C – v_1v_2 E = -13483.188654 a. u

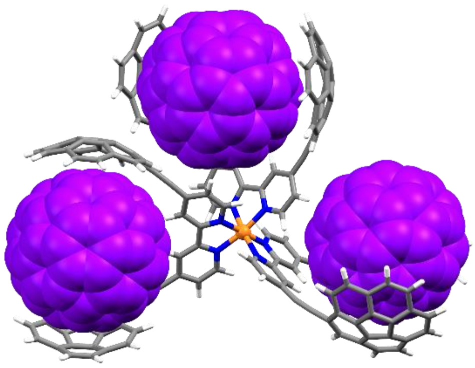

C	2.986106	-0.215856	-0.360348	N	-0.373069	-3.384127	-2.468773	H	-13.609974	1.044362	0.467408
C	1.733686	0.919061	-1.949768	N	1.934091	-3.215117	-0.628135	C	-11.907681	-2.787948	2.568639
C	2.314063	2.123696	-1.584725	N	-0.666021	-1.372888	-0.805423	C	-11.106302	-7.078227	1.479652
C	3.245063	2.168306	-0.534426	C	-2.420194	-5.226986	-2.991258	C	-9.56556	-10.696598	-0.567647
C	3.570388	0.953324	0.086191	C	-3.109791	-0.155506	-0.233664	H	-6.795473	1.499755	4.722503
H	3.240731	-1.166507	0.092569	C	-1.644562	-3.137695	-2.06156	C	-8.073589	-3.837573	4.673437
H	2.030244	3.0481	-2.070778	C	-1.820776	-1.907903	-1.27477	C	-6.508551	1.85542	2.645783
H	4.271997	0.911018	0.909655	C	-2.678603	-4.02648	-2.310826	C	-9.851823	-6.779486	-1.050015
N	2.076481	-0.247905	-1.346109	C	-3.046442	-1.308811	-1.035039	C	-5.535751	-7.780827	-3.349982
C	0.674459	0.806885	-2.964342	C	-0.122035	-4.524924	-3.127567	C	-9.698699	1.683988	4.718438
C	0.077996	1.904692	-3.567863	C	-0.735415	-0.344609	0.051563	C	-11.478395	1.923311	3.02178
C	-0.716482	-0.643257	-4.126462	C	-1.09945	-5.463685	-3.405986	C	-7.279977	-10.38186	-1.406395
C	-0.995231	1.721698	-4.453069	C	-1.92636	0.266298	0.389418	C	-10.268951	-9.334884	-3.044445
H	0.376272	2.912454	-3.308074	H	-3.678421	-3.825882	-1.942773	C	-4.3184	0.554678	-0.102643
C	-1.367061	0.400138	-4.755196	H	-3.956876	-1.703718	-1.469884	C	-7.834052	-9.304982	-3.348473
H	-0.991585	-1.673908	-4.321247	H	0.907405	-4.675874	-3.435059	C	-6.81881	-6.069211	3.439304
H	-2.171376	0.201231	-5.453407	H	0.208349	0.022659	0.439093	C	-5.536302	-3.379495	1.479065
N	0.261774	-0.458985	-3.225984	H	-0.844186	-6.375265	-3.933441	C	12.906145	-2.891722	-5.238746
C	-3.244791	4.91218	-5.447349	H	-1.927076	1.106869	1.067392	C	12.22997	0.442191	-1.247861
C	-4.637873	4.783599	-5.045533	N	2.616102	-2.528322	-3.055037	H	12.037586	-7.625167	2.409734
C	-2.700928	6.143062	-5.835425	C	3.52462	-4.735002	1.103699	C	9.254896	-2.895441	3.713111
C	-5.395045	5.917723	-5.25426	C	5.030127	-3.218506	-4.289653	C	8.086814	-4.232314	0.487911
C	-5.24255	3.793521	-4.192107	C	3.100981	-3.754313	-1.068691	C	12.333807	-4.951581	5.719433
C	-3.480292	7.345079	-5.857992	C	3.483793	-3.371398	-2.437923	C	10.677763	-0.59672	-2.660176
H	-1.630325	6.188409	-6.013804	C	3.896889	-4.526354	-0.23593	C	6.776778	-5.628164	4.658115
C	-4.834393	7.160083	-5.639337	C	4.673224	-3.755089	-3.04174	C	9.011644	-3.595563	-1.699416
C	-6.582324	6.182618	-4.528812	C	1.542231	-3.460845	0.63185	C	13.277095	-4.798687	-3.868332
H	-4.720971	2.857507	-4.01258	C	2.925441	-2.057902	-4.272934	C	6.192124	-4.464799	5.275694
C	-6.402488	4.056103	-3.485561	C	2.297197	-4.195368	1.524628	C	9.815414	-1.678845	-2.779915
C	-3.036091	8.716129	-5.772541	C	4.107035	-2.363515	-4.916224	C	14.741658	-2.637118	-3.753295
C	-5.676325	8.189932	-5.158247	H	4.844619	-4.921714	-0.578783	C	13.736044	-0.62721	-4.786377
C	-7.070755	5.331287	-3.556344	H	5.365352	-4.419382	-2.540847	H	13.909809	1.507665	-4.32377
C	-6.752992	7.586108	-4.468044	H	0.59534	-3.017924	0.921459	C	7.98412	0.948065	3.031562
H	-6.75101	3.319389	-2.768087	H	2.193114	-1.397097	-4.722912	C	6.55149	-2.545338	1.411738
H	-2.000452	8.956523	-5.996997	H	1.952111	-4.337285	2.541963	C	4.412411	-5.395017	1.975638
C	-3.859531	9.719003	-5.294323	H	4.326448	-1.933893	-5.886443	C	9.45955	-2.25711	-5.879556
C	-5.210157	9.456537	-4.85789	H	-12.097755	1.139269	-1.879985	C	13.727842	-3.499302	-4.307764
C	-7.955125	5.94863	-2.598557	H	-12.291402	-9.373402	-2.202185	C	11.324192	2.045006	0.915934
C	-7.42236	8.213883	-3.433869	C	-10.524582	2.526708	2.222718	H	9.151289	-5.442246	-4.5675
H	-3.440928	10.713209	-5.161999	C	-11.745823	-3.766541	-0.855744	C	11.517269	-1.93671	3.624514
C	-6.042284	10.200711	-3.945579	C	-9.079997	-7.03823	3.470139	H	7.802004	-0.829967	-5.870987
C	-8.121391	7.321911	-2.541013	C	-6.178706	-2.242317	2.108075	C	9.026883	2.329041	1.28382
H	-8.430895	5.332953	-1.83938	C	-7.585103	-10.803179	0.910665	C	12.910443	-1.497645	-5.472558
C	-7.094928	9.608864	-3.268356	C	-10.305429	-8.316115	-4.066855	C	9.251955	2.310533	-0.147611
H	-5.785657	11.229409	-3.707235	H	-11.706946	-10.503629	-0.145425	C	9.660602	-4.587018	-5.002899
H	-8.724323	7.737088	-1.737724	C	-11.131573	-6.148601	-0.798327	C	6.310163	-3.44839	-4.832454
H	-7.624447	10.195208	-2.521968	H	-11.735629	1.171696	5.061803	C	9.666395	0.201746	-5.862794
C	3.884918	5.24263	3.219753	C	-11.628491	1.907259	0.118171	C	10.060411	-5.566697	6.030153
C	4.362316	6.08001	2.15033	C	-6.82347	-1.464264	1.072158	C	10.307411	2.164498	1.940308
C	3.452339	5.774331	4.42243	C	-9.014539	-9.865313	-2.809413	C	10.159864	-4.497508	1.550631
C	4.553718	7.403892	2.486712	H	-9.407038	1.3292	5.703343	C	8.300258	-3.532407	2.834073
C	4.36564	5.824996	0.71726	C	-6.796583	-3.676011	4.008361	H	6.428991	-2.787271	6.562274
C	3.446175	7.195087	4.671146	C	-8.050783	-0.858798	1.316797	H	5.219931	-4.117686	4.937729
H	3.012793	5.102697	5.155314	H	-3.955423	-8.498356	-0.995674	C	7.086133	-0.181741	2.904373
C	4.103237	7.945095	3.714681	C	-3.465811	-6.157093	-3.201225	C	8.734047	-1.591253	4.068861
C	4.585343	8.452199	1.534554	C	-10.031936	1.621994	-1.744749	C	10.386103	-4.520462	0.120845
C	4.407332	6.869024	-0.217184	C	-6.018453	-4.460583	-0.683436	C	9.915243	-7.685029	2.52292
C	2.648811	7.965552	5.592881	H	-13.48448	1.055531	2.884149	H	11.706504	-6.230466	-3.752829
C	3.853574	9.322997	3.51761	C	-11.262961	-9.69594	-2.063631	C	10.155805	0.708422	-2.308602
C	4.434691	8.244344	0.175014	C	-6.572678	-6.723832	2.170079	C	11.512324	0.499723	3.292148
C	4.158493	9.638687	2.173554	C	-10.117577	-1.111327	2.389493	C	10.196487	-2.970104	-2.251866
H	4.295328	6.608117	-1.265852	C	-6.660045	1.735966	0.047767	C	7.976629	-6.035198	5.205721
H	2.148054	7.462326	6.415739	C	-8.046327	-6.223367	4.075665	C	8.882242	-0.937159	-5.909941
C	2.401157	9.313761	5.396505	C	-11.108048	-2.465858	-0.890993	C	6.93554	1.089612	0.939844
C	2.927521	10.029804	4.261028	C	-9.083006	-0.880609	0.30234	C	10.399511	1.407849	3.104052
C	4.017266	9.406761	-0.572778	C	-8.848808	-1.499713	-0.920239	H	13.900084	-5.378178	-3.192105

C	3.552973	10.679538	1.494293	C	-10.927934	-10.343343	-0.886059	C	12.037984	1.107794	-5.253986
H	1.715163	9.816965	6.072802	C	-9.87862	-7.683545	1.235616	C	12.85454	0.354658	-0.007948
C	2.464337	11.245563	3.638559	C	-9.162359	2.613528	2.595619	C	6.172592	-1.251531	0.880107
C	3.596387	10.563865	0.056543	C	-9.063313	-5.882843	-1.867605	C	11.958243	-0.761042	-2.006772
H	3.936242	9.344943	-1.654686	C	-7.812342	-3.336428	-1.9423	C	8.395739	-1.501743	-2.553028
C	2.762005	11.555124	2.321934	C	-5.39907	-9.777902	-0.089313	C	12.977487	-2.093737	-0.207854
H	1.785378	11.897741	4.18157	C	-7.09528	2.16326	1.366456	C	6.47371	-6.239967	3.374208
H	3.200209	11.369833	-0.555328	H	-7.184969	1.212383	-1.952772	C	9.265302	-3.167217	7.13235
H	2.307063	12.438531	1.881965	C	-8.689376	-1.002051	2.607524	C	11.104405	0.135911	-5.768708
C	0.084278	9.35569	1.903581	C	-6.597952	-7.478338	-4.209523	C	6.875591	-0.130204	-1.195509
C	-0.058883	9.693867	0.50207	C	-5.541596	-4.618815	2.113036	C	12.473533	-5.919329	4.737693
C	-1.362224	10.299238	0.318417	C	-10.872385	-4.057487	4.245495	C	13.009561	-0.919659	1.953701
C	-2.02428	10.337565	1.606655	C	-6.696874	-10.404775	-0.154278	C	8.331227	1.69734	-0.991674
C	-1.130129	9.754714	2.586238	C	-8.863488	-2.64927	4.423109	H	9.838898	-8.126845	1.532999
C	-2.653648	8.371935	-2.38712	C	-8.387374	2.643781	1.409158	C	7.482482	-3.536929	-5.151598
C	-3.867515	8.770699	-1.706723	C	-7.840265	-8.191563	-4.167625	C	12.32481	-2.002663	-1.497912
C	-3.524335	9.79944	-0.746162	C	-6.181385	-4.769035	3.404576	C	7.406216	-7.045537	2.702265
C	-2.097147	10.036314	-0.833461	C	-9.275103	2.581914	0.305453	H	15.473732	-3.047839	-3.063209
C	-1.559187	9.153759	-1.848462	H	-6.498732	-6.602327	-4.844333	C	13.288984	0.744402	-4.785427
C	-5.165733	8.845043	0.817887	C	-5.627318	-8.847525	-2.363487	C	12.024025	-5.287626	-4.189839
C	-5.021447	8.506735	2.219711	C	-11.349352	-6.423643	2.749396	C	11.621494	-3.384085	-5.563402
C	-3.926802	9.288031	2.758052	C	-12.138685	-5.235737	2.49703	C	10.671851	2.135047	-0.374286
C	-3.39402	10.108576	1.690214	C	-9.881001	-2.310143	-1.530111	C	11.170847	-7.397443	3.024531
C	-4.159642	9.835342	0.490804	C	-12.382778	-5.154768	1.071345	C	11.354228	-6.700424	4.27521
C	-3.269247	7.362948	4.138727	C	-7.568528	-7.500144	1.587088	C	11.631764	-1.127914	-5.951281
C	-1.96718	6.756029	4.32294	C	-10.235047	-2.757061	4.212616	C	11.017531	-0.683804	3.965036
C	-0.961478	7.746403	3.996803	C	-9.151585	-7.772171	-4.603015	C	7.146837	1.07355	-0.436138
C	-1.642499	8.966103	3.611416	H	-9.841014	1.249179	-2.747296	C	14.745242	-1.271321	-3.981819
C	-3.068603	8.72849	3.699034	C	-8.845607	-7.665236	2.25159	C	12.263699	-3.260014	1.838806
C	0.410388	5.97586	2.984905	C	-5.783459	-5.827088	1.34886	C	6.439324	-0.09426	1.611444
C	1.069367	5.939189	1.695424	C	-8.921914	2.040645	-0.920616	C	10.83207	-2.295174	-6.006437
C	1.270211	7.304116	1.257523	C	-10.359809	-1.034034	0.963562	C	12.535124	-2.056753	2.599856
C	0.739699	8.186353	2.275081	H	-5.470742	1.538431	2.689517	C	12.488157	0.38442	2.307451
C	0.203338	7.364652	3.340726	C	-5.385053	1.139344	-0.073563	C	5.335785	-5.817087	2.648952
C	0.795764	6.599718	-1.050107	C	-8.672713	-10.527055	-1.607963	C	7.453255	-1.425303	3.410963
C	-0.098854	7.183185	-2.02984	H	-7.195313	-10.909342	1.919493	H	8.988671	-2.208965	7.563715
C	-0.306665	8.573435	-1.674325	C	-7.265782	1.834092	3.801527	C	8.236913	-4.024778	6.594593
C	0.456968	8.847362	-0.474523	C	-7.053051	-4.478116	-1.70037	H	9.171075	1.166636	-5.793664
C	1.135057	7.62819	-0.087745	C	-11.132297	-4.859512	-1.460182	C	6.394101	-1.268809	-0.552637
C	-1.351179	5.067396	-2.106944	C	-12.732168	1.477776	0.93893	C	8.741745	-4.319371	1.777618
C	-2.778307	4.830572	-2.017008	C	-9.238986	-7.530812	-0.054878	H	11.723632	2.141864	-5.140548
C	-3.458386	6.050646	-2.400605	C	-8.948906	-10.940121	0.713915	C	11.090165	-4.521758	-4.981118
C	-2.453896	7.039372	-2.728186	C	-9.241494	-3.447365	-2.160708	C	7.891715	-0.249018	-2.222692
C	-1.150443	6.432927	-2.548537	C	-8.686065	-5.085899	4.703593	C	11.522161	-3.919823	-0.411893
C	-4.622761	6.43245	-1.745795	C	-12.659489	1.483729	2.32095	C	6.886329	-3.704787	6.201293
C	-5.156813	5.611205	-0.678271	C	-10.596551	2.516288	0.809017	H	7.151814	-7.373974	1.698377
C	-5.697602	6.492076	0.336543	H	-9.576448	-11.148297	1.576429	C	8.791882	0.880708	-2.095459
C	-5.496008	7.858111	-0.10509	C	-9.853386	-4.696331	-2.122739	H	11.33933	-2.741751	7.348823
C	-4.831792	7.819553	-1.391759	H	-4.840677	-9.804252	0.842799	C	13.236842	-0.938257	0.522461
C	-5.560897	6.168277	1.682751	C	-10.114035	-5.198366	4.487002	C	11.078583	-3.881361	2.392875
C	-4.877263	4.950736	2.067036	H	-11.261576	-7.893512	-4.364351	C	7.012825	-3.363967	0.307461
C	-4.112137	5.224618	3.266701	C	-11.905587	-4.076608	3.230254	C	11.425446	-3.129767	-1.623322
C	-4.32116	6.613016	3.622519	C	-11.35204	-1.814415	0.379499	C	8.869745	-3.507279	-5.42212
C	-5.215872	7.196564	2.643911	C	-8.074052	-1.752547	3.603786	C	12.48172	-3.278634	0.464296
C	-2.86165	4.64104	3.447596	C	-7.569813	-2.130376	-1.176445	H	13.436917	-6.021915	4.245425
C	-2.322892	3.763773	2.42844	C	-6.795869	-2.38697	3.347908	C	8.915741	-6.834333	4.510965
C	-0.894973	3.996072	2.339661	C	-11.745216	-6.293365	0.441832	C	8.686033	-5.252828	6.149554
C	-0.552124	5.023104	3.302742	C	-4.890361	-9.032635	-1.139109	C	9.103772	-4.35496	-0.537077
C	-1.766855	5.421045	3.986021	C	-11.029927	1.593901	4.351352	C	10.617098	-3.065343	3.495817
C	-0.256707	3.957505	1.101455	C	-4.415666	-6.909512	-3.31066	C	9.596455	-0.507187	4.191553
C	-1.022239	3.684465	-0.098857	C	-7.813459	-7.417752	0.162281	H	13.193396	-4.330883	5.958472
C	-0.487416	4.503708	-1.169352	C	-12.142748	-2.710078	1.199168	C	12.39163	1.171826	1.095515
C	0.608142	5.285732	-0.62873	C	-7.054294	-6.561183	-0.625699	C	10.201578	-6.54883	5.023225
C	0.749232	4.948111	0.77364	C	-11.319374	1.558324	-1.247942	C	11.063058	-4.667501	6.340309
C	-2.395194	3.468784	-0.01255	C	-5.783582	-3.299208	0.051468	C	9.215203	0.785934	3.659544
C	-3.058031	3.503661	1.275622	C	-7.692576	-5.776552	-1.660992	H	15.479917	-0.663	-3.460762
C	-4.362172	4.105483	1.090441	H	-9.244171	-6.944271	-5.300752	C	10.609626	-3.4717	7.008544
C	-4.508129	4.437557	-0.311308	C	-12.385297	-3.917247	0.435599	C	7.184805	-2.627088	2.647272
C	-3.292528	4.039762	-0.995098	C	-6.017329	-5.750585	-0.021124	C	8.712422	-7.300407	3.22393
H	3.765693	4.177108	3.044539	C	-6.760902	-9.623724	-2.482253	C	7.898224	-2.687448	-1.885896
C	-1.715551	2.830023	-4.948983	C	-10.874994	-1.972116	3.175939	C	11.115214	1.35077	-1.434205
C	-2.38812	3.805378	-5.230123	C	-10.357922	-6.405265	3.724995	C	7.888539	1.735092	1.819366
C	3.778528	3.398962	-0.105981	C	-7.542406	1.681199	-1.039685	C	6.915466	-2.575246	-0.905648
C	4.101919	4.516145	0.253112	C	-8.682108	2.108445	3.789811				
Ru	0.998968	-1.914019	-1.928499	C	-6.577949	-2.112218	-0.199133				

$(C_{60})_3$@Ru3C – v_2v_1 E = -13483.209704 a. u

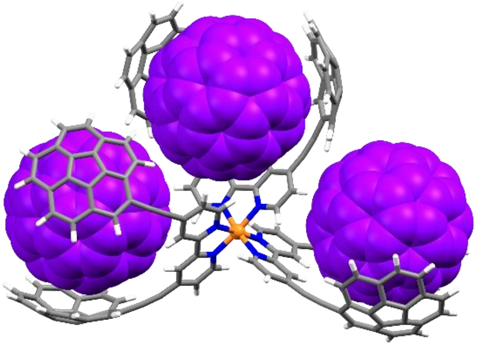

C	2.518844	-0.68274	0.319254	N	-1.072553	-4.23662	-0.180338	H	-13.359904	-7.760955	1.171649
C	1.077009	-0.092071	-1.401078	N	1.58454	-3.630887	0.972217	C	-11.199032	-4.027937	-3.123291
C	1.892621	0.969667	-1.759371	N	-1.049372	-2.00138	1.187427	C	-11.819763	-0.191504	-0.85681
C	3.05427	1.239055	-1.018396	C	-3.042273	-6.215866	0.042912	C	-11.29378	2.661068	3.194729
C	3.336086	0.399113	0.070655	C	-3.048389	-1.217083	2.979729	H	-7.434642	-8.179322	-4.272518
H	2.743995	-1.375847	1.12189	C	-2.079651	-4.102329	0.720982	C	-7.479655	-1.610997	-3.862519
H	1.625698	1.621289	-2.581298	C	-2.077595	-2.839052	1.47545	C	-6.763946	-8.497181	-2.278935
H	4.19164	0.586749	0.70532	C	-3.057108	-5.074424	0.861424	C	-11.02769	-0.958683	1.759352
N	1.417421	-0.942178	-0.400676	C	-3.072425	-2.481832	2.373671	C	-6.431739	0.109423	4.695334
C	-0.240097	-0.347695	-2.002173	C	-1.031255	-5.341175	-0.941607	C	-10.293543	-8.409923	-3.732492
C	-0.899396	0.587053	-2.783652	C	-0.99309	-0.808954	1.80174	C	-11.721822	-8.636102	-1.72798
C	-2.076614	-1.765392	-2.069607	C	-1.983362	-6.340387	-0.872339	C	-8.927613	2.690242	3.855921
C	-2.225857	0.361497	-3.180176	C	-1.953657	-0.383917	2.698319	C	-11.398226	0.395989	5.020297
H	-0.438208	1.538885	-3.012561	H	-3.868444	-4.95061	1.567002	C	-4.102129	-7.141892	0.128685
C	-2.801875	-0.871872	-2.836286	H	-3.90983	-3.138932	2.564977	C	-9.010472	0.899944	5.282963
H	-2.509511	-2.711502	-1.763787	H	-0.200889	-5.400922	-1.636647	C	-7.140513	0.358455	-1.841597
H	-3.816911	-1.107605	-3.129845	H	-0.152622	-0.175624	1.539131	C	-5.551282	-2.45719	-0.33545
N	-0.831664	-1.513505	-1.634537	H	-1.915252	-7.204495	-1.522576	C	11.174355	-1.07028	-2.904555
C	-4.095179	3.696485	-4.404268	H	-1.87708	0.596918	3.149472	C	8.9454	-5.20064	5.722207
C	-5.330661	4.01516	-3.704061	N	1.616126	-3.418928	-1.646455	C	9.942369	-2.177224	-6.570841
C	-3.396728	4.665728	-5.136722	C	3.672925	-4.70811	2.499857	C	9.351894	-6.776022	3.87308
C	-5.829957	5.271638	-3.973343	C	3.817971	-4.055455	-3.255363	C	12.488098	-2.855817	1.120358
C	-5.923024	3.354986	-2.571312	C	2.694323	-4.125188	0.364921	C	11.481121	0.258655	-2.416883
C	-3.851384	6.021033	-5.23484	C	2.725522	-3.977061	-1.099223	C	7.12094	-0.844081	-2.315407
H	-2.426882	4.389462	-5.540926	C	3.735352	-4.674412	1.097177	C	12.130583	-3.631907	6.015303
C	-5.110702	6.246473	-4.706246	C	3.822225	-4.316605	-1.87605	C	10.768861	-6.028066	-4.323484
C	-6.702114	5.947811	-3.08578	C	1.495482	-3.705518	2.309832	C	8.631625	-3.981762	-1.538888
H	-5.619665	2.34162	-2.330836	C	1.582082	-3.2207	-2.97328	C	9.982723	1.981908	-1.499976
C	-6.785962	4.011631	-1.712011	C	2.501609	-4.220617	3.104823	H	14.131123	-5.298931	3.762033
C	-3.096855	7.217624	-5.524879	C	2.644201	-3.51574	-3.80695	C	10.043474	-4.346357	-5.845703
C	-5.539659	7.522437	-4.276253	H	4.62814	-5.042089	0.607864	C	7.140412	-3.660932	5.872205
C	-7.123064	5.403757	-1.886441	H	4.71842	-4.722863	-1.426926	C	6.766619	-2.564399	1.83229
C	-6.518636	7.337994	-3.271961	H	0.584827	-3.308682	2.744419	C	6.467491	-0.125938	1.672986
H	-7.136238	3.476228	-0.833016	H	0.667367	-2.786096	-3.361323	C	9.509181	1.253779	2.912696
H	-2.126653	7.133035	-6.007031	H	2.390683	-4.236033	4.1826	H	7.540097	-1.787302	6.796357
C	-3.517477	8.464297	-5.098441	H	2.576721	-3.313065	-4.869269	C	9.670695	-5.365629	-4.987131
C	-4.734577	8.644091	-4.343293	H	-11.424246	-7.807703	3.208125	C	9.902446	-2.3562	3.519616
C	-7.569236	6.371181	-0.913731	H	-13.446768	0.199401	4.268496	C	7.661498	-2.103025	-2.550598
C	-6.749099	8.266063	-2.274308	C	-10.621666	-9.201285	-1.111466	C	12.047118	1.385241	-0.304844
H	-2.861787	9.315351	-5.262578	C	-11.949157	-4.106304	0.361278	C	12.794417	-1.526886	1.610712
C	-5.117562	9.707922	-3.448106	C	-9.530275	0.79768	-2.214135	C	9.614957	-3.390415	-2.423935
C	-7.391357	7.732293	-1.098108	C	-5.726249	-3.464265	-1.36421	C	11.201547	-2.47962	3.042495
H	-7.963718	6.030687	0.040442	C	-9.554192	3.77325	1.835267	C	13.270754	-5.123099	4.402222
C	-6.076518	9.527832	-2.465294	C	-11.088818	-0.886023	5.60749	H	6.10808	-3.386521	5.674483
H	-4.581182	10.652537	-3.48055	H	-13.3593	2.080955	2.751229	C	6.707202	0.953377	0.736195
H	-7.66121	8.403163	-0.286706	C	-12.012633	-1.742641	1.042401	C	7.135543	-5.733422	4.273164
H	-6.253354	10.337739	-1.762377	H	-12.370823	-7.947135	-3.704203	C	10.955501	-3.36673	-2.057124
C	5.41749	4.722284	1.115221	C	-11.325724	-8.573412	1.157119	C	7.754387	1.795482	1.278369
C	5.417153	5.310763	-0.200211	C	-6.30937	-4.639312	-0.746538	C	10.427525	-4.500049	0.062718
C	5.471946	5.499527	2.258612	C	-10.338375	1.28294	4.98628	C	7.273821	-1.911147	-5.929224
C	5.689408	6.662734	-0.235793	H	-10.199759	-8.080081	-4.763679	C	11.369846	-3.931821	-0.788544
C	4.865287	4.780365	-1.437502	C	-6.355924	-1.691437	-2.951701	C	9.459081	-0.886963	-6.46896
C	5.518951	6.940436	2.201069	C	-7.261358	-5.382944	-1.434283	H	6.230624	-1.771491	-5.660375
H	5.376925	5.006566	3.222712	H	-5.314554	1.981377	2.761468	H	14.364052	-3.735028	-4.378222
C	5.734828	7.456492	0.937405	C	-4.137897	-0.770439	3.75895	C	6.167309	-1.404277	1.203256
C	5.311308	7.500297	-1.313434	C	-9.411985	-8.261889	2.691001	C	7.395153	-4.380789	-5.072319
C	4.522507	5.614835	-2.51171	C	-6.709593	-2.200368	1.822546	H	12.510185	0.613811	-5.937888
C	5.111518	7.911722	3.186236	H	-13.688012	-7.811595	-1.224616	C	11.759662	-2.346	6.55378
C	5.379323	8.778731	0.581957	C	-12.543669	0.803406	4.245021	C	13.276285	-4.006187	5.222227
C	4.656475	7.036747	-2.442365	C	-7.319003	0.643896	-0.432951	H	14.141336	-3.348766	5.192419
C	5.123477	8.807429	-0.808789	C	-9.082545	-5.260127	-2.903053	C	7.61751	1.307423	-1.523114
H	4.008039	5.161037	-3.354122	C	-6.41728	-8.376417	0.310509	C	8.852824	-3.190323	2.977907
H	4.981596	7.601169	4.219534	C	-8.220857	0.435491	-2.714503	C	6.097047	-1.651482	-0.223184
C	4.760781	9.204598	2.83695	C	-10.997119	-5.191148	0.240698	C	10.491916	1.841488	2.025723
C	4.784985	9.657385	1.468294	C	-8.43014	-5.888172	-0.743374	C	12.382871	-4.325163	-5.104505
C	3.947529	8.049324	-3.18996	C	-8.607254	-5.623296	0.609146	C	9.374496	-2.981236	6.687505
C	4.265716	9.719308	-1.395933	C	-12.493696	1.881563	3.377538	C	10.449119	-2.036828	6.875577

H	4.369707	9.861626	3.609397
C	4.063665	10.739747	0.844798
C	3.761013	9.324367	-2.689205
H	3.464086	7.781307	-4.125615
C	3.81678	10.770082	-0.517988
H	3.619401	11.511902	1.467554
H	3.136503	10.014397	-3.250411
H	3.188806	11.564174	-0.913
C	1.653773	8.355639	1.255997
C	1.147408	8.685266	-0.06003
C	-0.070414	9.451726	0.106771
C	-0.316546	9.597343	1.527354
C	0.749824	8.920495	2.238027
C	-2.317318	7.66042	-2.003579
C	-3.220821	8.224094	-1.023166
C	-2.491787	9.216346	-0.259862
C	-1.136006	9.266542	-0.768897
C	-1.028088	8.304387	-1.847427
C	-3.704054	8.508736	1.759872
C	-3.196949	8.179495	3.076892
C	-1.908404	8.822574	3.234195
C	-1.618594	9.548868	2.015075
C	-2.728156	9.355491	1.103863
C	-1.108417	6.852988	4.46879
C	0.109305	6.086013	4.302583
C	1.085881	6.932776	3.648147
C	0.471246	8.223765	3.409527
C	-0.884718	8.173426	3.916721
C	1.881521	4.981292	2.381895
C	2.123016	4.837785	0.961342
C	2.348177	6.158298	0.408209
C	2.243622	7.11812	1.485529
C	1.95414	6.392026	2.705722
C	1.135012	5.481761	-1.620081
C	0.070333	6.158453	-2.332607
C	0.142692	7.57038	-2.009536
C	1.250555	7.763561	-1.096129
C	1.862868	6.474345	-0.85514
C	-1.390413	4.221226	-1.928922
C	-2.74517	4.172234	-1.417928
C	-3.359707	5.461905	-1.658674
C	-2.386011	6.30726	-2.315081
C	-1.168062	5.54086	-2.484627
C	-4.226697	6.00332	-0.717549
C	-4.517214	5.275864	0.501173
C	-4.628742	6.236559	1.580175
C	-4.405624	7.557783	1.026167
C	-4.15745	7.411803	-0.393374
C	-4.142352	5.921606	2.8453
C	-3.525186	4.633376	3.081541
C	-2.41625	4.827309	3.992652
C	-2.346476	6.236647	4.318915
C	-3.412928	6.913347	3.609854
C	-1.246675	4.090089	3.836123
C	-1.138225	3.131615	2.75527
C	0.218958	3.17343	2.249877
C	0.947889	4.165911	3.014936
C	0.04255	4.730449	3.995191
C	0.454535	3.03294	0.883418
C	-0.655712	2.83714	-0.028749
C	-0.365298	3.569001	-1.247821
C	0.923036	4.212473	-1.089953
C	1.426844	3.884267	0.228519
C	-1.960117	2.791362	0.457634
C	-2.204341	2.945282	1.87744
C	-3.422445	3.709888	2.04513
C	-3.932478	4.033266	0.728278
C	-3.028441	3.46681	-0.253999
H	5.280858	3.649563	1.215277
C	-2.948014	1.407263	-3.798749
C	-3.473794	2.45034	-4.143727
C	3.848086	2.367543	-1.296693
C	4.383194	3.452668	-1.434539
Ru	0.25475	-2.651886	-0.276975
C	-10.876029	0.562967	-0.167106
C	-9.351329	-9.275129	-1.728897
C	-10.189547	-1.870834	2.508288
C	-8.323364	-3.99963	2.274307
C	-7.098983	3.080832	2.390641
C	-7.092731	-8.79434	-0.908189
H	-6.549458	-7.901984	2.387051
C	-7.664126	-4.995684	-2.770376
C	-7.299482	-0.73789	5.396697
C	-5.776585	-1.113658	-0.628374
C	-10.258891	-2.125584	-4.119758
C	-8.495705	3.289308	2.687486
C	-7.943578	-2.958472	-4.117727
C	-8.36932	-9.278021	-0.707941
C	-8.669029	-0.393672	5.638362
C	-6.188394	-0.725352	-1.961941
C	-9.038048	-9.211235	0.538027
H	-6.937282	-1.729549	5.652983
C	-6.872331	1.398495	4.181562
C	-11.634779	-0.471991	-2.26665
C	-12.100933	-1.81994	-2.520249
C	-9.916938	-5.267405	1.113061
C	-12.573258	-2.373207	-1.267945
C	-8.580132	0.984487	0.047024
C	-9.306596	-3.210421	-4.241329
C	-9.791485	-1.262091	5.904492
H	-9.045025	-7.891633	3.644535
C	-9.706663	1.067603	-0.860436
C	-6.477724	-0.26764	0.318033
C	-8.467513	-8.670823	1.677319
C	-9.554478	-5.812788	-1.650117
H	-5.757761	-8.168784	-2.523512
C	-5.14444	-7.764489	0.227089
C	-10.286978	2.384256	4.100476
H	-9.305782	4.300465	0.917541
C	-7.724557	-8.501295	-3.275717
C	-7.87634	-2.704175	2.517527
C	-11.783561	-3.140468	1.349311
C	-12.572254	-8.179133	0.550587
C	-10.470699	0.16975	1.166819
C	-10.884865	3.474321	2.076068
C	-9.74346	-4.263828	2.142672
C	-8.394529	-0.569691	-3.742463
C	-12.759782	-8.208593	-0.821831
C	-10.428782	-9.167204	0.288837
H	-11.625414	3.786038	1.344159
C	-10.656182	-3.219755	2.256275
H	-6.6506	3.572859	1.531115
C	-9.812664	-0.830543	-3.877226
H	-11.882254	-1.616782	5.740148
C	-11.427554	-2.63052	-3.428493
C	-10.811863	-5.470136	-1.168171
C	-7.104501	-3.870887	-3.367307
C	-7.621121	-4.842596	1.327871
C	-6.118917	-3.088055	-2.648169
C	-12.400131	-1.367107	-0.23981
C	-6.328859	2.173465	3.096201
C	-11.535618	-8.333509	-3.125929
C	-5.175916	-0.383576	4.264218
C	-9.053687	0.430648	1.299188
C	-11.651892	-4.559398	-1.919936
C	-8.249488	-0.44534	2.01769
C	-10.771101	-8.214526	2.440647
C	-6.030648	-3.011844	0.916914
C	-8.828662	-1.618345	2.635373
H	-9.611411	-2.274147	6.256903
C	-12.353941	-3.715197	-0.97418
C	-6.939517	-0.804169	1.51404
C	-8.136657	1.769986	4.587086
C	-9.88725	-4.386269	-3.625644
C	-10.514918	0.014	-2.932608
C	-7.084316	-8.330375	1.544026
C	-9.110452	-8.792756	-3.000902
C	-6.49522	-4.361302	0.664105
C	7.959625	-6.629854	3.576706
H	10.178887	-7.862243	2.156198
C	9.142523	-4.117819	1.984476
C	10.272335	1.055149	-2.496697
C	11.733353	-6.878199	3.227447
H	10.219215	-1.021923	7.188733
C	12.658681	-2.014191	-1.185416
C	12.35025	0.420967	-1.342992
C	11.999884	-1.294498	2.800117
C	4.785468	-5.165549	3.235494
C	8.166264	1.233306	2.548102
C	6.639852	0.714676	-0.634155
C	7.424029	-3.184987	-1.616758
C	13.427271	-2.101309	-5.371299
C	13.488831	-3.41527	-4.937455
C	12.117148	-5.980168	4.290605
C	10.477163	0.127098	-6.339013
C	8.233943	-4.291033	0.868951
C	7.917295	0.343134	-2.562105
C	9.028526	-4.524176	-0.320161
H	12.478866	-7.195576	2.503122
H	12.508593	-1.561801	6.627004
C	13.019582	-0.489937	0.710798
C	10.495511	-4.248114	1.487253
C	9.778633	-1.096098	-3.284587
C	9.345495	-1.043546	3.773924
C	7.370904	0.046995	2.792239
C	12.95163	-0.738216	-0.714945
C	11.363431	-3.845225	-5.905202
C	7.855256	-3.225934	-5.830788
C	11.806495	-0.193884	-6.121389
C	5.85154	-5.467677	3.741265
C	9.77164	-6.097585	5.003659
C	6.655621	-2.966223	-0.475511
C	10.419065	-7.259326	3.027812
C	8.697923	2.358167	0.42466
H	10.187486	1.174343	-6.316695
C	6.087041	-4.365999	-4.530823
H	14.256205	-1.439944	-5.132934
C	9.784354	-4.262009	6.371036
C	10.109677	0.094751	3.541702
C	12.254099	-1.561245	-6.013635
C	11.465182	-0.033421	3.043845
C	11.12432	-4.578574	6.047653
H	7.882729	-6.172802	-4.021129
C	7.961894	-2.747337	6.510333
C	10.888787	2.151139	-0.382105
C	12.461666	0.82256	0.964
C	9.162843	-3.317475	-6.259429
C	7.948594	-1.068127	3.390757
C	9.015147	-2.232585	-3.049561
C	9.219756	0.217269	-3.034285
C	8.037593	-0.79883	-6.236736
C	7.64403	-2.395926	2.897569
H	7.563062	0.178354	-6.20219
C	11.504263	-3.443712	2.005894
C	11.751382	-2.182994	-2.302155
C	8.273117	-5.399657	-4.67705
C	11.117147	-5.715497	5.207432
C	12.057815	-5.529888	-4.377799
C	10.094699	2.382981	0.80693
H	10.568715	-6.884467	-3.685211
C	6.330278	-0.612955	-1.122969
H	12.825334	-6.015319	-3.780754
C	7.06892	-3.531254	0.79445
C	11.300573	-2.503887	-6.347994
H	7.535661	-7.130994	2.710987
C	11.700001	1.045829	2.106398
C	7.648736	-4.905625	5.354172
C	12.423171	-3.094929	-0.249034
C	4.98887	-4.26853	-4.01357
C	8.62821	2.109492	-1.001866

$(C_{60})_3$@Ru3C – v_2v_2 E = -13483.209709 a. u

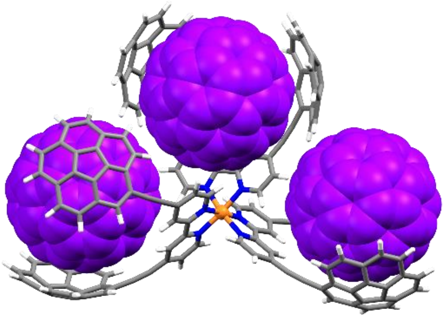

C	-1.034259	-5.311657	-0.904404
C	-2.07138	-4.07221	0.764559
C	-3.037656	-5.052484	0.924624
C	-3.024051	-6.198298	0.112806
C	-1.977716	-6.317737	-0.81774
H	-0.212256	-5.368998	-1.609537
H	-3.839904	-4.931018	1.640726
H	-1.91191	-7.184583	-1.464467
N	-1.074198	-4.203379	-0.1484
C	-2.074176	-2.801667	1.506673
C	-3.066889	-2.442503	2.406769
C	-1.00879	-0.756143	1.798074
C	-3.054173	-1.167741	2.991745
H	-3.893158	-3.107944	2.617071
C	-1.970251	-0.327385	2.69191
H	-0.176057	-0.118323	1.522436
H	-1.901911	0.661355	3.126159
N	-1.055939	-1.957909	1.201309
C	-6.450811	0.149289	4.684157
C	-6.904935	1.427801	4.155636
C	-7.311947	-0.703947	5.386575
C	-8.178521	1.783667	4.546525
C	-6.363636	2.202627	3.068672
C	-8.68806	-0.377519	5.614346
H	-6.937303	-1.687603	5.655295
C	-9.045428	0.907315	5.243309
C	-8.976962	2.684773	3.799755
H	-5.344409	2.02255	2.741751
C	-7.141491	3.091265	2.348267
C	-9.799747	-1.25979	5.880111
C	-10.376313	1.267527	4.931667
C	-8.544022	3.280107	2.629994
C	-10.333628	2.360585	4.035261
H	-6.69253	3.582908	1.488938
H	-9.607784	-2.2656	6.243923
C	-11.099773	-0.906154	5.568262
C	-11.423018	0.365124	4.965364
C	-9.60209	3.740098	1.764113
C	-11.336487	2.613528	3.118174
H	-11.883322	-1.647404	5.701322
C	-12.567446	0.74755	4.17608
C	-10.930089	3.422543	1.995608
H	-9.35362	4.263069	0.843958
C	-12.525982	1.817627	3.298298
H	-13.461464	0.130137	4.197658
H	-11.668654	3.71644	1.254383
H	-13.388918	1.997651	2.662555
C	-6.707028	-8.540951	-2.188886
C	-7.043537	-8.823469	-0.816747
C	-7.660463	-8.561907	-3.192116
C	-8.319404	-9.310752	-0.619966
C	-6.378637	-8.387983	0.401816
C	-9.046984	-8.856565	-2.923625
H	-7.364999	-8.250013	-4.19047
C	-9.294345	-9.324821	-1.64757
C	-8.997099	-9.232631	0.620383
C	-7.054522	-8.331566	1.629857
C	-10.226837	-8.487722	-3.667547
C	-10.56918	-9.24967	-1.039709
C	-8.436969	-8.676892	1.757371
C	-10.386288	-9.198942	0.361383
H	-6.527485	-7.891297	2.471681
H	-10.127808	-8.169669	-4.70191
C	-11.473254	-8.40962	-3.070267
C	-11.667851	-8.697103	-1.670198
C	-9.390642	-8.261887	2.759965
C	-11.292376	-8.600607	1.216922
H	-12.306165	-8.034461	-3.659073
N	1.413519	-0.905698	-0.389196
N	1.613108	-3.393091	-1.617979
N	-0.835601	-1.486759	-1.622492
C	3.04543	1.276224	-1.016283
C	-2.229627	0.383074	-3.17407
C	1.073675	-0.062953	-1.396222
C	-0.243045	-0.32364	-1.996459
C	1.887584	0.998884	-1.759648
C	-0.901974	0.608032	-2.782669
C	2.51233	-0.639052	0.332188
C	-2.082393	-1.73839	-2.053034
C	3.326721	0.443909	0.078907
C	-2.808154	-0.846202	-2.820496
H	1.622488	1.643671	-2.587591
H	-0.439923	1.558332	-3.014706
H	2.736259	-1.325713	1.140454
H	-2.516266	-2.682045	-1.741005
H	4.180499	0.639897	0.713277
H	-3.826388	-1.074412	-3.108966
N	1.576831	-3.585124	1.001982
C	3.809059	-4.063002	-3.220562
C	3.662548	-4.650762	2.542173
C	2.718566	-3.953826	-1.065185
C	2.686297	-4.086645	0.40045
C	3.811849	-4.311222	-1.83866
C	3.727093	-4.628093	1.139372
C	1.580374	-3.206489	-2.946526
C	1.486571	-3.648119	2.340321
C	2.640141	-3.518134	-3.777254
C	2.490615	-4.1573	3.141021
H	4.703902	-4.722856	-1.38577
H	4.620028	-5.000739	0.654567
H	0.669227	-2.767421	-3.337875
H	0.576041	-3.246008	2.770259
H	2.574797	-3.324471	-4.841372
H	2.378094	-4.163607	4.218705
H	3.150864	10.051325	-3.247196
H	-4.607306	10.659803	-3.47646
C	5.361098	8.806481	0.601106
C	-0.030475	9.442399	0.029722
C	-4.157191	6.040716	2.848012
C	0.383439	3.03178	0.934629
C	-7.592992	6.371191	-0.917705
C	-3.540767	8.474717	-5.097857
H	-6.279712	10.340691	-1.759507
C	-2.456691	9.242331	-0.326663
H	4.326765	9.879389	3.623708
C	4.263337	9.751852	-1.383364
C	1.369093	3.852107	0.260517
C	-5.56161	7.529692	-4.276647
H	4.938352	7.618834	4.232366
C	-1.202558	3.19721	2.808099
C	2.083332	4.808223	0.972379
H	-5.637295	2.345882	-2.339954
C	-2.953387	1.425269	-3.796541
C	3.962202	8.086561	-3.18464
C	-1.447903	4.196099	-1.896754
H	3.591674	11.535576	1.479813
C	-5.142287	9.714377	-3.445335
C	-3.985914	4.106568	0.769286
C	1.94637	6.400263	2.685343
C	4.86676	4.813101	-1.432402
C	-3.562359	4.746719	3.109049
C	1.173043	8.651101	-0.124235
C	2.329726	6.113336	0.392259
C	1.847259	6.412526	-0.876308
C	-6.101467	9.531831	-2.463247
C	-4.396413	7.644297	0.99691
H	3.181291	11.594649	-0.903951
C	0.498149	8.272016	3.355392
C	-3.676006	8.597347	1.709574
C	-6.772594	8.268976	-2.274013
H	5.348201	5.026978	3.231603
C	-1.291446	4.178764	3.869531
C	5.404132	4.748594	1.123728
C	-3.205126	8.247975	-1.068004
C	-4.113229	3.705406	-4.410478
C	5.075336	7.932186	3.200857
C	4.75878	9.682028	1.485143
C	-6.72305	5.95203	-3.089342
C	-4.75772	8.652269	-4.341888
C	3.837818	2.405153	-1.297145
C	-5.131915	6.254809	-4.709072
C	-3.478195	3.801031	2.091357
C	-0.73261	2.837288	0.028851
C	11.182333	-1.118766	-2.914577
C	8.94363	-5.135624	5.754647
C	9.957039	-2.300659	-6.563826
C	9.32509	-6.744425	3.929317
C	12.459717	-2.851338	1.145398
C	11.492975	0.215641	-2.444566
C	7.126293	-0.861809	-2.354927
C	12.139297	-3.580226	5.990594
C	10.734044	-6.115142	-4.238141
C	8.615109	-3.995174	-1.521563
C	9.997961	1.960273	-1.563169
H	14.109714	-5.297654	3.748885
C	10.032758	-4.456042	-5.796063
C	7.150151	-3.580656	5.890627
C	6.735575	-2.518614	1.814574
C	6.451868	-0.081455	1.616642
C	9.491544	1.300867	2.856737
H	7.569475	-1.694379	6.779688
C	9.645831	-5.455163	-4.920266
C	9.86126	-2.302	3.519871
C	7.662181	-2.126633	-2.567132
C	12.051178	1.370477	-0.345599
C	12.769991	-1.516722	1.617942
C	9.607284	-3.422767	-2.409091
C	11.162627	-2.439392	3.053025
C	13.256568	-5.105465	4.393985
H	6.118312	-3.302851	5.695163
C	6.702674	0.983191	0.666116
C	7.119818	-5.677621	4.326056
C	10.945509	-3.401367	-2.034381
C	7.750546	1.827734	1.202932
C	10.397225	-4.500331	0.098756
C	7.288684	-1.994871	-5.941125
C	11.348236	-3.949713	-0.754635
C	9.486614	-1.003742	-6.491157
H	6.245581	-1.839221	-5.681062
H	14.352443	-3.859095	-4.320064
C	6.147138	-1.36485	1.164335
C	7.381165	-4.448752	-5.035207
H	12.550797	0.476292	-5.978245
C	11.780711	-2.282396	6.508152
C	13.275308	-3.973854	5.193118
H	14.142842	-3.320985	5.143201
C	7.629266	1.298319	-1.591861
C	8.810668	-3.138194	2.983147
C	6.083815	-1.632774	-0.258403
C	10.483216	1.869933	1.967565
C	12.368754	-4.443758	-5.043832
C	9.394363	-2.902823	6.677902
C	10.475301	-1.960712	6.837973
C	7.932227	-6.592414	3.639501

C	-12.714426	-8.265318	-0.775902	C	5.716143	7.483835	0.955598	H	10.130576	-7.865776	2.225089
C	-10.748211	-8.224903	2.500076	C	-2.31412	7.648798	-2.038709	C	9.101563	-4.082696	2.006068
H	-9.032356	-7.878881	3.711735	C	0.045815	6.098575	-2.343059	C	10.289084	1.017346	-2.544141
C	-12.536774	-8.219954	0.597315	C	-6.807015	4.013396	-1.719443	C	11.700134	-6.873152	3.265939
H	-13.642189	-7.878247	-1.18923	C	5.410251	5.34059	-0.190184	H	10.253034	-0.938941	7.133666
H	-11.408428	-7.812523	3.258482	H	4.022436	5.198381	-3.353544	C	12.649925	-2.044937	-1.171445
H	-13.331181	-7.799471	1.208107	C	1.848281	4.984787	2.390395	C	12.355799	0.389224	-1.367249
C	-10.69627	-5.557931	-1.146061	C	-3.41642	4.676608	-5.141777	C	11.969024	-1.262327	2.798448
C	-10.894052	-5.260808	0.257301	C	-2.036272	2.825345	0.519466	C	4.773329	-5.101527	3.284794
C	-11.872655	-4.197633	0.356798	C	-1.102841	6.95102	4.4456	C	8.150928	1.282042	2.483485
C	-12.281147	-3.837897	-0.98635	C	-7.14483	5.405606	-1.891369	C	6.642324	0.724557	-0.700738
C	-11.554929	-4.679928	-1.915686	C	0.00921	4.799377	4.012533	C	7.412528	-3.19327	-1.61955
C	-10.176553	-1.885971	2.474029	C	5.680668	6.693222	-0.220009	C	13.436838	-2.236293	-5.349842
C	-11.033402	-1.006374	1.707413	C	-3.872794	6.031418	-5.238853	C	13.482967	-3.541833	-4.889562
C	-11.995967	-1.825445	0.999432	C	-2.273995	3.0121	1.936562	C	12.097756	-5.958469	4.309503
C	-11.734463	-3.212254	1.329669	C	5.309844	7.533258	-1.298132	C	10.514868	0.002065	-6.378542
C	-10.609315	-3.24959	2.242154	H	-2.445614	4.402601	-5.54517	C	8.199434	-4.267808	0.887252
C	-11.832835	-0.300611	-0.923779	C	-5.349743	4.021482	-3.710394	C	7.930814	0.317037	-2.614321
C	-11.635009	-0.599229	-2.32818	C	-3.171227	8.285776	3.031716	C	9.000662	-4.522633	-0.292978
C	-12.067209	-1.962	-2.561684	C	-1.871122	8.908982	3.173166	H	12.437798	-7.206909	2.540992
C	-12.531354	-2.506391	-1.302501	C	1.257232	7.707164	-1.141884	H	12.5346	-1.501195	6.55865
C	-12.387027	-1.479996	-0.290133	C	-1.571742	9.605401	1.939066	C	13.006665	-0.494494	0.70424
C	-10.211811	-2.248472	-4.149154	C	-4.549575	5.354394	0.518396	C	10.457217	-4.227549	1.519744
C	-9.233143	-3.311747	-4.249699	C	0.100743	6.159421	4.29228	C	9.788878	-1.142657	-3.303166
C	-9.787789	-4.491198	-3.617243	C	-3.119586	7.229191	-5.527155	C	9.309778	-0.982592	3.750486
C	-11.110009	-4.156962	-3.125672	H	3.487168	7.821022	-4.125254	C	7.347844	0.103415	2.739726
C	-11.370809	-2.770526	-3.454282	C	-4.641399	6.338606	1.577987	C	12.946611	-0.763689	-0.718088
C	-7.55427	-5.032509	-2.743725	C	-3.094332	3.504764	-0.20324	C	11.357802	-3.969204	-5.858365
C	-7.147901	-5.388363	-1.400023	C	4.664958	7.072127	-2.433655	C	7.855841	-3.313457	-5.813668
C	-8.307058	-5.910665	-0.705592	C	2.24512	7.096204	1.450041	C	11.839769	-0.327954	-6.148056
C	-9.428842	-5.877117	-1.617543	H	5.27025	3.675076	1.220111	C	5.836498	-5.406725	3.794755
C	-8.965198	-5.333073	-2.877295	C	4.378858	3.487428	-1.433966	C	9.758308	-6.050206	5.045243
C	-7.531948	-4.813253	1.351921	C	-6.540545	7.342649	-3.272929	C	6.637538	-2.953795	-0.487532
C	-8.258441	-3.972375	2.281763	H	-7.988943	6.028953	0.03523	C	10.382112	-7.249445	3.083992
C	-9.67111	-4.272935	2.148931	C	5.448491	5.5229	2.269593	C	8.702727	2.372426	0.347129
C	-9.81604	-5.296902	1.135037	C	-1.203637	5.500101	-2.479273	H	10.236295	1.052551	-6.37943
C	-8.496124	-5.629058	0.642049	C	-1.101498	9.258274	-0.839536	C	6.072182	-4.410778	-4.497646
C	-6.686687	-2.141942	1.807178	C	3.806259	10.799643	-0.506057	H	14.271739	-1.579112	-5.12103
C	-6.949583	-0.757207	1.474857	C	-4.154435	7.465258	-0.419892	C	9.79374	-4.191607	6.381252
C	-8.269679	-0.42199	1.967679	C	-7.415757	7.732751	-1.099493	C	10.081552	0.148078	3.506844
C	-8.822785	-1.59852	2.602246	C	0.143843	7.515274	-2.048722	C	12.272565	-1.697146	-6.009031
C	-7.843906	-2.662689	2.505606	C	-2.447492	4.939597	4.013384	C	11.439481	0.005096	3.019789
C	-9.091736	0.422721	1.232288	C	4.041672	10.765453	0.858694	C	11.129	-4.521293	6.05099
C	-8.626616	0.968009	-0.026722	C	5.116586	8.838816	-0.791485	H	7.845393	-6.224505	-3.947055
C	-9.750963	1.009426	-0.939802	H	-7.687189	8.401901	-0.287217	C	7.982054	-2.662429	6.507722
C	-10.91059	0.487638	-0.242703	C	-1.01337	8.272926	-1.89871	C	10.8976	2.141283	-0.441964
C	-10.5015	0.125669	1.098951	H	-7.158621	3.475426	-0.842732	C	12.454211	0.824531	0.934184
C	-9.562237	0.722247	-2.288394	C	-2.351991	6.353744	4.310865	C	9.164557	-3.426582	-6.233699
C	-8.242009	0.384691	-2.778289	H	-2.885838	9.3265	-5.26114	C	7.915368	-1.005549	3.3586
C	-8.387111	-0.641787	-3.7899	C	-0.857247	8.255869	3.86645	C	9.01788	-2.271171	-3.055596
C	-9.797929	-0.939289	-3.925705	C	1.676885	8.339164	1.196894	C	9.235499	0.177354	-3.076552
C	-10.524445	-0.096964	-2.997649	C	0.901867	4.199254	3.041832	C	8.065172	-0.8967	-6.267354
C	-7.447029	-1.662614	-3.889646	C	1.100077	5.417692	-1.619255	C	7.606841	-2.338878	2.883119
C	-6.325499	-1.701367	-2.973615	C	0.153682	3.204706	2.29864	H	7.60002	0.085544	-6.256076
C	-6.056044	-3.086604	-2.646421	C	-2.687005	9.413233	1.034461	C	11.466965	-3.420679	2.032998
C	-7.019445	-3.904329	-3.356619	C	-5.942856	3.358921	-2.579456	C	11.74925	-2.225473	-2.291629
C	-7.877157	-3.024812	-4.124909	C	4.724803	9.225265	2.85239	C	8.246653	-5.468968	-4.616698
C	-5.659923	-3.432465	-1.355011	C	-3.487184	2.462048	-4.14781	C	11.107907	-5.672939	5.231137
C	-5.513911	-2.405072	-0.341897	C	-4.249281	6.051936	-0.715323	C	12.028025	-5.630783	-4.295461
C	-5.984672	-2.950894	0.917318	C	0.78593	8.939965	2.169439	C	10.097033	2.39524	0.738238
C	-6.415302	-4.315146	0.684693	C	-3.39409	5.476615	-1.64694	H	10.522563	-6.957243	-3.584701
C	-6.21702	-4.611186	-0.72075	C	3.770825	9.360243	-2.682272	C	6.328389	-0.608599	-1.17175
C	-5.770838	-1.072517	-0.657533	C	-0.432821	3.540257	-1.20455	H	12.787679	-6.112454	-3.685498
C	-6.186187	-0.715679	-1.998906	C	-2.407415	6.291127	-2.322472	C	7.039027	-3.502788	0.793375
C	-7.164284	0.346837	-1.89949	H	-2.149524	7.146548	-6.009921	C	11.310667	-2.63647	-6.328088
C	-7.355735	0.649915	-0.496356	C	-0.27022	9.621151	1.447622	H	7.499089	-7.103628	2.784317
C	-6.49601	-0.228921	0.272229	C	-2.801762	4.181447	-1.38174	C	11.686356	1.069015	2.068088
H	-5.701004	-8.209914	-2.430604	C	-5.850149	5.278092	-3.977717	C	7.646498	-4.837453	5.390375
C	-4.145524	-0.719458	3.767108	C	1.090437	6.975322	3.618646	C	12.402408	-3.110489	-0.220714
C	-5.187673	-0.333481	4.264462	C	-3.408163	7.034647	3.590553	C	4.977138	-4.295482	-3.9775
C	-4.075636	-7.132087	0.21338	C	4.531547	5.650299	-2.506879	C	8.641018	2.102373	-1.075839
C	-5.1115	-7.764141	0.319026	C	5.492242	6.964101	2.216303				
Ru	0.249963	-2.616277	-0.256557	C	0.867081	4.163313	-1.06288				

Table S 12. Summary of the interaction energies of the supramolecular adducts with C_{60} computed with the counterpoise scheme.[39–42]

Adduct	C_{60}@Ru1C	$(C_{60})_2$@Ru2C – v_1	$(C_{60})_3$@Ru3C – v_2v_2
E_{int} / kcalmol^{-1}	-35.84	-75.73	-113.83

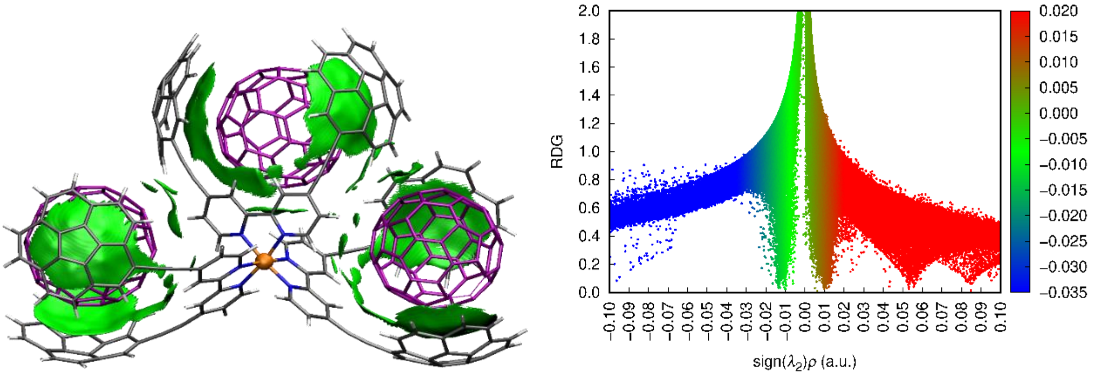

Figure S 168. NCI plot (isovalue = 0.3 a.u.) (left) and plot of the reduced density gradient versus the electron density multiplied by the sign of the second Hessian eigenvalue (λ_2) (right) of the supramolecular adduct **$(C_{60})_3$@Ru3C - v_2v_2**.

In order to evaluate the BAr^F_4 anion impact in the recognition process, further calculations were performed. To do so, a crystal structure of **$Ru(bpy)_3 \cdot BAr^F_4$** was used as starting point to locate the BAr^F_4 anions. From this structure, **Ru1C·BAr^F_4** complex was modelled, with the BAr^F_4 anions in different positions regarding the corannulene substituents (Figure S 169). The geometries of the different structures were optimized using Grimme's GFN2-xTB semiempirical quantum mechanical method[35,36] with toluene as the solvent of choice. Then the obtained structures were further optimized with computational DFT methods in gas phase using PBE1PBE[21–24] functional along with 6-31G(d,p)[28–31] basis set for light atoms and LANL2DZ[32,33] for the Ru atom. The impact of the solvent was described by single-point calculation via the Polarizable Continuum Model (PCM)[37,38] choosing toluene as the solvent (ε = 2.3741). Moreover, the interaction energies were calculated using the counterpoise scheme,[39–42] and the non-covalent interactions were analyzed in the same manner as the **$(C_{60})_n$@Ru*n*C** adducts described before.[44,45,47,48]

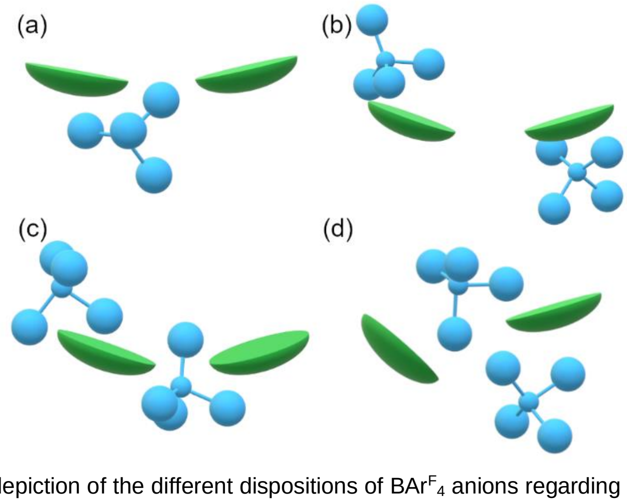

Figure S 169. Cartoon depiction of the different dispositions of BAr^F_4 anions regarding the corannulene moiety in **Ru1C·BAr^F_4**: (a) v_1, (b) v_2, (c) v_3 and (d) v_4. Note: in v_1 the second anion is not represented as it is not close to any corannulene unit.

Ru1C·BAr$^{F}_{4}$ – v$_{1}$ E = -10551.5499453 a. u

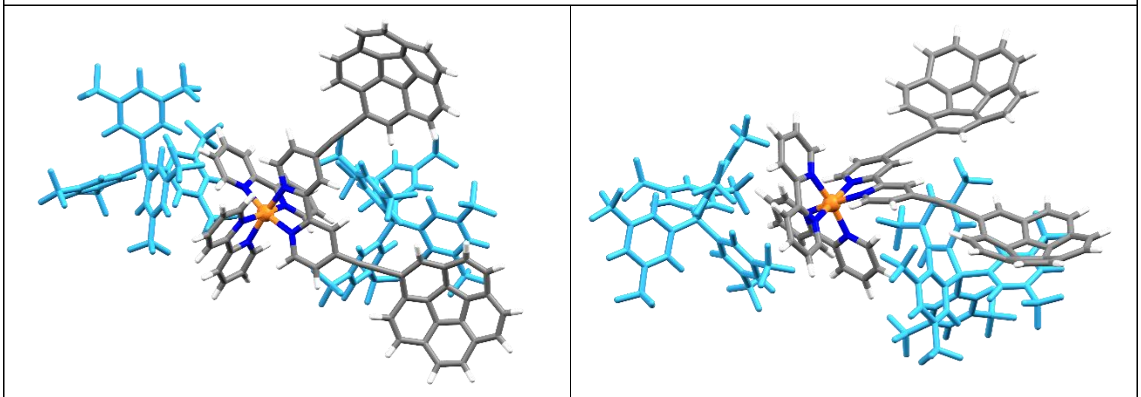

Ru	1.604427	-0.421874	-1.704935	F	-8.100521	-0.136264	6.168495	F	10.787236	-5.602322	1.851746
N	3.112044	0.001003	-0.343284	C	-9.267215	-2.566115	1.174798	C	11.104184	2.659939	1.54117
N	2.730641	-0.068362	-3.401901	C	-9.142994	-0.189758	1.2145	F	5.410436	0.152067	4.67453
N	0.802433	1.486872	-1.476882	C	-3.540748	2.204693	0.30898	C	5.511939	-2.1852	2.603224
N	-0.158539	-0.597167	-2.779999	H	-3.160849	3.188205	0.060109	H	4.663801	-2.583834	3.149359
N	2.244555	-2.330103	-2.175389	F	-6.639453	-0.019716	7.764024	C	7.542866	-0.868101	2.532542
C	-0.474186	1.606057	-1.916805	C	-5.175371	0.435344	0.033904	H	8.257376	-0.25045	3.068853
N	0.702604	-0.952487	0.09653	H	-6.039671	0.051788	-0.500117	C	6.451898	-1.380604	3.234923
C	1.381785	2.549191	-0.895623	F	-11.314277	-3.650548	0.706531	F	12.878646	-2.03388	-2.934308
H	2.404676	2.413772	-0.562714	C	-3.922918	-5.568636	0.484304	C	10.11349	3.53532	1.978835
C	3.063277	-2.425932	-3.251018	H	-3.563763	-6.545545	0.185283	H	10.366709	4.518777	2.356661
C	-1.042938	0.405148	-2.542858	F	-1.522889	-1.384798	5.712427	F	4.93697	-4.637989	0.746682
C	0.729799	3.751722	-0.702673	F	-10.123909	-4.42007	2.337893	F	11.019149	-4.166341	3.445744
H	1.234039	4.568139	-0.199832	C	-9.915115	-1.338644	1.108918	C	7.653881	3.963077	2.363432
C	-0.597871	3.887779	-1.137581	H	-10.984918	-1.280677	0.951197	F	7.322757	-0.640377	5.300791
C	-1.366431	5.046536	-0.905962	C	-6.827708	0.182476	6.449318	F	3.438613	-3.10232	0.951693
C	1.859353	-3.447253	-1.544976	F	-9.591754	1.900875	2.20411	F	12.75615	4.099365	2.415255
H	1.200826	-3.313411	-0.696625	C	-4.695944	-1.102665	6.181377	F	13.373657	2.104636	1.837105
C	2.909202	1.129997	-3.975152	H	-4.490532	-0.96821	7.23686	F	14.304421	-3.102225	-1.702492
H	2.395935	1.959436	-3.502219	C	-1.697328	1.938583	1.962355	C	13.40345	-2.107226	-1.697206
C	4.272872	0.59153	-0.657956	F	-4.066337	-6.624038	3.571386	F	4.737417	-3.134096	-0.788526
H	4.53143	0.626674	-1.708504	F	-1.527714	1.481055	3.207935	F	12.899764	3.570246	0.325194
C	-1.180587	2.789745	-1.787889	B	-5.477644	-1.571988	1.783362	F	14.076502	-0.960202	-1.498108
H	-2.191204	2.876568	-2.164322	F	-9.48615	-4.74371	0.303981	C	6.201293	-0.953776	4.647643
C	3.349916	-1.154905	-3.928466	F	-9.178602	1.873099	0.088308	F	5.555206	-1.892759	5.35564
C	-0.503449	-1.521165	0.230092	C	-4.927503	-6.590028	2.538956	C	12.536347	3.10755	1.53511
H	-1.064446	-1.685675	-0.680752	F	-1.61549	3.27706	1.995795	C	-5.422419	-1.466635	-3.16639
C	2.750792	-0.0915	0.959979	C	-9.756385	1.169459	1.090104	C	-10.576018	-4.222846	-2.66649
C	3.705962	1.309989	-5.094455	C	-10.052719	-3.841885	1.126177	C	-9.210768	-4.560544	-2.812014
H	3.836161	2.302468	-5.508938	F	-2.479588	-5.853984	-1.775615	C	-10.651718	-2.887207	-2.210309
C	2.276845	-4.70734	-1.942665	F	-11.071351	1.122492	0.82832	C	-8.441648	-3.430794	-2.450041
H	1.935479	-5.580002	-1.397885	C	-2.845269	-4.590642	-1.526895	C	-9.333958	-2.396009	-2.085874
C	-2.393192	0.258523	-2.810818	C	-2.512951	-2.292823	5.908589	C	-11.577344	-4.816187	-3.408607
H	-3.102836	1.022408	-2.528392	F	-6.570162	2.769894	-1.145429	C	-8.76334	-5.514932	-3.703078
C	-1.913329	-1.890019	-3.763649	F	-4.798182	-7.75384	1.879909	C	-11.733881	-2.064891	-2.455421
H	-2.219244	-2.817577	-4.227932	F	-4.649034	3.580389	-1.744654	C	-7.178705	-3.198459	-2.946152
C	-0.587864	-1.683662	-3.439343	F	-2.099508	-3.418929	5.296339	C	-9.021881	-1.053208	-2.185935
H	0.160197	-2.438503	-3.65108	C	-5.309765	2.437886	-1.44251	C	-11.149078	-5.957524	-4.180665
C	4.167627	-1.039969	-5.05054	F	-1.693226	-3.868706	-1.454304	C	-12.794981	-4.045593	-3.485778
H	4.68663	-1.904383	-5.443269	F	-5.363099	1.728328	-2.595645	C	-9.812128	-6.290643	-4.319841
C	3.538427	-3.66118	-3.685698	F	-6.162081	-6.550777	3.059001	C	-7.368415	-5.393641	-4.052261
H	4.220027	-3.721157	-4.523918	F	-3.489034	-4.143872	-2.624415	C	-12.870825	-2.739028	-3.030982
C	-2.870524	-0.94278	-3.360661	F	-2.557156	-2.534532	7.223046	C	-11.453287	-0.653629	-2.328722
C	-4.252294	-1.210099	-3.389371	C	8.470716	-0.195527	-1.209649	C	-6.61098	-4.291759	-3.691495
C	1.448409	-0.722901	1.203373	F	8.650697	-3.065373	-4.30895	C	-6.754732	-1.808105	-2.850879
C	-1.014958	-1.910082	1.458232	F	12.766101	-4.977135	2.452911	C	-10.163916	-0.169861	-2.200369
H	-1.99277	-2.377947	1.496909	C	10.226068	-1.541677	0.303243	C	-7.650419	-0.787509	-2.489758
C	4.343645	0.20257	-5.64253	C	9.461327	0.939551	0.985312	H	-11.884087	-6.526323	-4.744724
H	4.983606	0.304439	-6.512979	C	8.367031	-1.244971	-2.13726	H	-13.659664	-4.464876	-3.994265
C	3.555653	0.432852	1.968215	H	8.760637	-2.223624	-1.872556	H	-9.550081	-7.107971	-4.987031
H	3.265159	0.347722	3.00795	C	7.401791	1.22708	-2.890846	H	-6.911367	-6.144604	-4.691962
C	5.088442	1.180006	0.295235	C	11.241841	-1.446212	-0.657083	H	-13.791201	-2.185763	-3.199537
H	5.982702	1.697257	-0.024368	H	11.175562	-0.682706	-1.429157	H	-12.265933	0.062818	-2.416413
C	3.144116	-4.814775	-3.0232	C	7.287037	0.165607	-3.778695	H	-10.008012	0.903927	-2.170701
H	3.509892	-5.782866	-3.348477	H	6.848362	0.307252	-4.75673	H	-7.292274	0.236449	-2.533966
C	0.986477	-1.078896	2.467334	C	6.761153	-1.911999	0.549641	C	-2.125957	5.971877	-0.683254
H	1.598856	-0.89686	3.342293	H	6.832224	-2.122105	-0.512017	C	-5.386411	10.353419	-0.004999
C	-0.25755	-1.679898	2.601529	C	8.492154	1.832252	1.451765	C	-4.787608	9.080793	0.127808
H	-0.625988	-1.957744	3.58265	H	7.45517	1.515954	1.455046	C	-4.370776	11.32844	0.129478
C	4.726257	1.100272	1.634505	C	7.777168	-1.074759	-3.388475	C	-3.402744	9.267607	0.352224
H	5.34011	1.544412	2.410587	C	7.992031	1.048789	-1.639805	C	-3.145313	10.658274	0.352764
C	-7.083721	-1.496903	1.46302	H	8.090216	1.911346	-0.98534	C	-6.53612	10.562774	-0.743309
C	-5.254189	-1.466392	3.408569	C	11.454726	-3.426951	1.251737	C	-5.31077	7.941828	-0.457395
C	-6.129623	-0.778535	4.256097	F	6.945766	3.385508	3.359254	C	-4.444537	12.57162	-0.468446
H	-7.055729	-0.375618	3.855896	F	6.540419	-3.018607	-3.857672	C	-2.449503	8.335593	0.003218
C	-4.099032	-1.983497	4.012397	C	12.455844	-3.307307	0.293758	C	-1.92257	11.192422	-0.005633

H	-3.403443	-2.568761	3.416533	H	13.30696	-3.97766	0.292773	C	-7.205588	9.356428	-1.167445
C	-4.870888	-2.997231	1.267241	C	5.685644	-2.450203	1.249996	C	-6.723226	11.923417	-1.181113
C	-7.883342	-2.638846	1.323363	C	10.784806	1.392773	1.053507	C	-6.625268	8.108295	-1.030624
H	-7.424108	-3.622803	1.329763	H	11.591882	0.742266	0.727906	C	-4.371145	6.874731	-0.628331
C	-3.545496	0.264041	1.748748	C	10.365018	-2.55915	1.254787	C	-5.72826	12.877959	-1.050675
H	-3.12123	-0.230984	2.615899	H	9.607402	-2.679665	2.023943	C	-3.171934	13.227871	-0.641826
C	-4.691964	-0.273695	1.140708	F	5.573772	2.689015	-2.655635	C	-2.997267	7.050906	-0.407215
C	-7.766104	-0.272242	1.413427	C	7.725059	-1.100029	1.160633	C	-1.118639	8.858527	-0.157297
H	-7.21096	0.65617	1.519595	C	8.795529	3.102819	1.929529	C	-1.972417	12.572728	-0.420922
C	-2.965157	1.45722	1.332075	C	7.587582	-2.260852	-4.278424	C	-0.868403	10.220522	-0.159883
C	-4.664128	-5.411315	1.649382	C	11.511659	-4.540248	2.25349	H	-8.155056	9.429973	-1.691509
F	-0.616278	1.515648	1.252088	F	7.299096	-1.91113	-5.550002	H	-7.632182	12.19293	-1.712828
C	-3.814179	-1.799221	5.362579	F	7.531902	3.583319	-2.817201	H	-7.138324	7.246062	-1.448163
C	-3.662737	-4.444636	-0.287606	C	6.798754	2.550442	-3.237652	H	-4.699766	5.916071	-1.019022
F	-6.700645	1.504099	6.231723	B	8.996764	-0.47703	0.321773	H	-5.895309	13.860145	-1.485737
C	-5.86139	-0.603305	5.613306	F	6.596607	2.697436	-4.559768	H	-3.142833	14.236988	-1.045079
C	-4.639317	1.66861	-0.349708	C	12.339375	-2.305474	-0.661323	H	-1.047653	13.092364	-0.658471
C	-4.137389	-3.18161	0.089806	F	8.034962	5.170775	2.79039	H	0.141511	10.558766	-0.37767
H	-3.934003	-2.323746	-0.54901	C	4.711205	-3.332349	0.543259	H	-5.588919	-4.218246	-4.048899
C	-5.113636	-4.153412	2.030983	F	6.773344	4.149104	1.352578	H	-0.302316	8.174844	-0.373656
H	-5.6744	-4.062329	2.957458								

Ru1C·BAr$^{F}_{4}$ – v$_2$ E = -10550.9892811 a. u

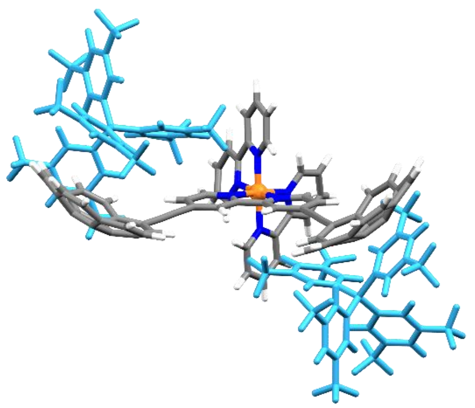
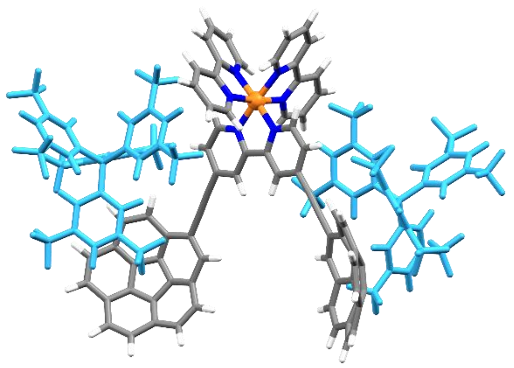

Ru	6.284373	30.542176	6.039400	H	5.880239	31.471244	1.966666	H	8.635941	42.084115	10.021052
N	7.028205	32.396911	6.110537	F	4.260510	35.187177	-7.163104	C	9.846805	39.404951	11.721781
N	7.659362	29.645411	7.202383	C	3.383435	30.893710	-0.263176	H	10.361430	39.195499	12.647767
N	7.208964	30.071808	4.323415	H	2.779792	30.072127	-0.619855	C	9.575757	40.729126	11.407616
N	5.600696	28.672174	5.792099	F	-0.504857	27.789670	-5.335254	F	11.270984	33.704908	16.163889
N	5.421099	30.781535	7.820229	C	-2.073522	32.550830	-0.563349	C	14.054763	36.748710	9.571767
C	7.126757	28.762337	3.984306	H	-3.109194	32.515530	-0.264065	H	15.045805	36.754923	9.144993
N	4.867039	31.503072	5.008061	F	2.948776	37.649346	-1.662863	F	6.444021	40.854515	8.592598
C	7.932202	30.890307	3.553735	F	-0.714648	29.772803	-6.135846	F	6.863716	38.402260	15.142757
H	7.972502	31.930277	3.846975	C	1.993423	28.769586	-4.650623	C	13.291163	35.401240	7.637310
C	6.005161	30.092896	8.832803	H	1.963988	27.900774	-5.289528	F	9.380812	41.753050	13.536392
C	6.251078	27.965546	4.839043	C	4.413909	34.221849	-6.240316	F	8.204681	41.360061	7.477585
C	8.603455	30.460300	2.424847	F	5.442038	29.271129	-4.780133	F	14.806055	39.480706	11.209679
H	9.162511	31.169129	1.835177	C	3.450827	35.814089	-4.579043	F	14.804749	38.044715	12.810148
C	8.536079	29.119290	2.074112	H	3.772297	36.638301	-5.196600	F	12.249865	35.521027	16.762698
C	8.528944	36.283099	5.655194	C	5.872981	34.057162	1.091648	C	11.765191	34.868671	15.688366
C	4.310149	31.480309	8.071086	F	-2.281772	35.329628	-2.740454	F	7.414591	39.359376	7.401646
H	3.883537	32.019718	7.237314	F	5.188315	35.147623	1.480192	F	16.107627	37.771433	11.121571
C	8.829698	29.142757	6.802984	B	2.133537	32.392250	-2.021964	F	12.826352	34.537483	14.938932
H	9.155332	29.418613	5.809254	F	-1.514980	29.305597	-4.195557	C	10.035604	41.796494	12.356842
C	8.182347	32.755221	6.683380	F	4.954562	27.908995	-3.195299	F	9.875651	43.043375	11.889763
H	8.703244	31.981542	7.229332	C	-2.673235	34.077082	-2.456520	C	14.869978	38.157234	11.471648
C	7.789645	28.264618	2.865743	F	6.694315	34.464629	0.107936	C	1.501585	34.537601	2.084663
H	7.710831	27.223267	2.602072	C	4.463443	28.464563	-4.324344	C	-2.058313	37.787857	-0.527415
C	7.234915	29.397244	8.463792	C	-0.458586	29.090458	-4.998985	C	-0.970547	36.943298	-0.223423
C	3.727904	30.970378	4.550997	F	-2.676760	31.214932	1.803044	C	-3.244002	37.137897	-0.120198
H	3.564372	29.929182	4.790591	F	4.356521	27.465056	-5.211200	C	-1.483192	35.767518	0.373348
C	6.340369	33.321827	5.396275	C	-1.475182	30.952929	1.272703	C	-2.888122	35.893454	0.443955
C	9.605751	28.331121	7.606021	C	2.514959	37.430564	-2.919475	C	-1.943384	39.164538	-0.579437
H	10.545159	27.951448	7.237858	F	3.482368	28.442917	1.031484	C	0.298093	37.426326	0.047943
C	3.725943	31.530742	9.321567	F	-3.876522	34.175772	-1.872653	C	-4.385135	37.824191	0.247964
H	2.831758	32.112974	9.473271	F	5.598704	28.657286	0.712133	C	-0.762673	34.998779	1.263604
C	6.038397	26.598024	4.690500	F	1.199599	37.718079	-2.915604	C	-3.648966	35.267195	1.413285
H	6.575018	26.042730	3.939080	C	4.518616	29.257952	1.252327	C	-0.594828	39.645960	-0.515260
C	4.441165	26.697632	6.458234	F	-0.593875	31.015954	2.290867	C	-3.184333	39.862724	-0.423211
H	3.708992	26.241422	7.105005	F	4.733106	29.229820	2.582399	C	0.464861	38.826330	-0.214040
C	4.704174	28.048892	6.561490	F	-2.894557	33.486386	-3.655162	C	1.134356	36.515536	0.756741
H	4.172649	28.664877	7.274531	F	-1.505309	29.650416	0.914277	C	-4.340510	39.228482	-0.033801
C	7.958772	28.567699	9.315153	F	3.097918	38.373668	-3.676970	C	-5.286128	37.068401	1.067283
H	7.597309	28.359933	10.308601	C	9.003411	35.623874	10.730103	C	0.640454	35.345840	1.328153
C	5.470507	30.111915	10.119056	F	5.226029	36.114435	9.070782	C	-1.556660	34.202579	2.145720

H	5.960598	29.581983	10.918657	F	7.780353	37.783811	16.987716	C	-4.937710	35.859336	1.619314
C	5.134380	25.952232	5.514832	C	9.876373	36.621304	12.889490	C	-2.925301	34.326020	2.212451
C	2.201206	33.802372	2.745228	C	11.446059	36.737298	10.686393	H	-0.418133	40.708497	-0.615896
C	5.106662	32.820412	4.792628	C	7.695181	35.764272	10.271924	H	-3.191467	40.939619	-0.526359
C	2.811909	31.676652	3.807655	H	7.231903	36.738097	10.261173	H	1.445732	39.262634	-0.085647
H	1.909851	31.207012	3.447778	C	8.755901	33.224353	10.414376	H	2.171973	36.768894	0.928725
C	9.151272	28.018317	8.879327	C	10.836475	35.888243	13.584379	H	-5.222529	39.825408	0.156386
C	6.810839	34.614864	5.245980	H	11.692130	35.490540	13.059502	H	-6.237854	37.507801	1.334611
H	6.265010	35.341337	4.667134	C	7.481837	33.386624	9.885693	H	-1.053100	33.554930	2.849776
C	8.714361	34.018604	6.579635	H	6.904692	32.539478	9.547398	H	-5.624746	35.381656	2.304625
H	9.661742	34.251040	7.038802	C	8.818929	38.704198	9.702757	H	-3.461166	33.758021	2.960185
C	4.318358	30.837075	10.368387	H	8.550267	37.944150	8.985127	C	8.929018	37.409811	5.458815
C	4.226556	33.600137	4.062731	C	11.731479	36.090211	9.485147	C	11.053011	42.698651	6.578746
H	4.414119	34.648825	3.901208	H	10.938200	35.575854	8.963692	C	11.422025	41.357394	6.820767
C	3.067973	33.027041	3.525473	C	6.962914	34.671416	9.827666	C	10.191248	42.721279	5.460055
C	8.025742	34.987850	5.834037	C	9.503985	34.321328	10.809836	C	10.781041	40.548085	5.855601
C	2.061947	31.051443	-2.956808	H	10.496543	34.164584	11.203973	C	10.021585	41.392834	5.013497
C	2.595058	33.641886	-2.959931	C	8.679814	36.873201	14.986053	C	10.998706	43.646983	7.582894
C	3.264327	33.441817	-4.166556	F	12.207172	34.821595	7.096334	C	11.768797	40.894577	8.075981
H	3.465551	32.438950	-4.509925	F	5.422227	34.209291	8.098335	C	9.228142	43.696800	5.280732
C	2.369457	34.967350	-2.586113	C	9.648713	36.154524	15.671853	C	10.444218	39.233120	6.106902
H	1.849609	35.181858	-1.662297	H	9.569809	35.985574	16.734995	C	8.875188	40.967633	4.362566
C	0.644495	32.596467	-1.393523	C	8.491766	40.024005	9.420426	C	11.573643	43.225007	8.826114
C	0.879952	30.614238	-3.550125	C	12.506295	37.392532	11.311668	C	10.164827	44.772354	7.283026
H	-0.040938	31.148632	-3.372457	H	12.339318	37.916511	12.240573	C	11.942999	41.922002	9.058866
C	4.038898	33.172103	-0.333926	C	8.787506	37.094413	13.620831	C	11.637227	39.479714	8.238627
H	3.961467	34.170022	-0.738005	H	8.014745	37.655462	13.116768	C	9.327446	44.796010	6.192790
C	3.220266	32.160774	-0.829669	F	9.587469	31.277436	9.344206	C	8.160769	43.318237	4.401782
C	3.212251	30.317705	-3.251019	C	9.478222	38.353890	10.881532	C	11.002920	38.693357	7.306959
H	4.161813	30.621939	-2.833673	C	13.007168	36.094704	8.939123	C	9.368314	38.731582	5.282324
C	4.980813	32.929193	0.659331	C	5.575993	34.842524	9.280000	C	7.993401	42.027617	3.965360
C	-1.665099	33.315568	-1.644814	C	7.482637	37.373796	15.742247	C	8.631556	39.568497	4.446563
F	6.669020	33.736061	2.127529	F	4.634068	34.313844	10.087775	H	11.633791	43.935155	9.639999
C	2.792780	36.028678	-3.378378	F	10.426198	31.752867	11.262040	H	10.124055	45.590567	7.989678
C	-1.099396	31.842929	0.124084	C	9.303726	31.832558	10.543910	H	12.296279	41.643025	10.042822
F	5.746688	34.110016	-6.046248	B	9.932902	36.839694	11.277144	H	11.952661	39.034499	9.171153
C	3.676620	34.500889	-4.962303	F	8.422755	30.987611	11.114998	H	8.652730	45.633240	6.073767
C	4.320559	30.649703	0.728619	C	10.727332	35.664423	14.949438	H	7.408585	44.054372	4.151592
C	0.228924	31.872972	-0.277617	F	13.775235	36.235834	6.699760	H	10.837639	37.649443	7.530643
H	0.949202	31.298154	0.283920	C	7.658899	40.385709	8.226053	H	7.115145	41.781511	3.384105
C	-0.337785	33.331809	-2.056490	F	14.206397	34.419669	7.764583	H	7.773368	39.148482	3.939493
H	-0.075994	33.912703	-2.929113	F	6.540025	36.415016	15.890550	H	9.044364	28.748473	1.198272
F	4.033683	33.075365	-6.829563	C	13.784549	37.393425	10.769355	H	4.965420	24.891425	5.414290
C	0.844896	29.495753	-4.372540	F	11.342574	41.677487	12.665461	H	9.721754	27.371281	9.527283
C	3.179202	29.202024	-4.077460	C	8.874518	41.059405	10.260062	H	3.894806	30.865929	11.359710
C	5.140171	31.664711	1.204196								

Ru1C·BAr^{F}$_4$ – v$_3$ E = -10551.557061 a. u

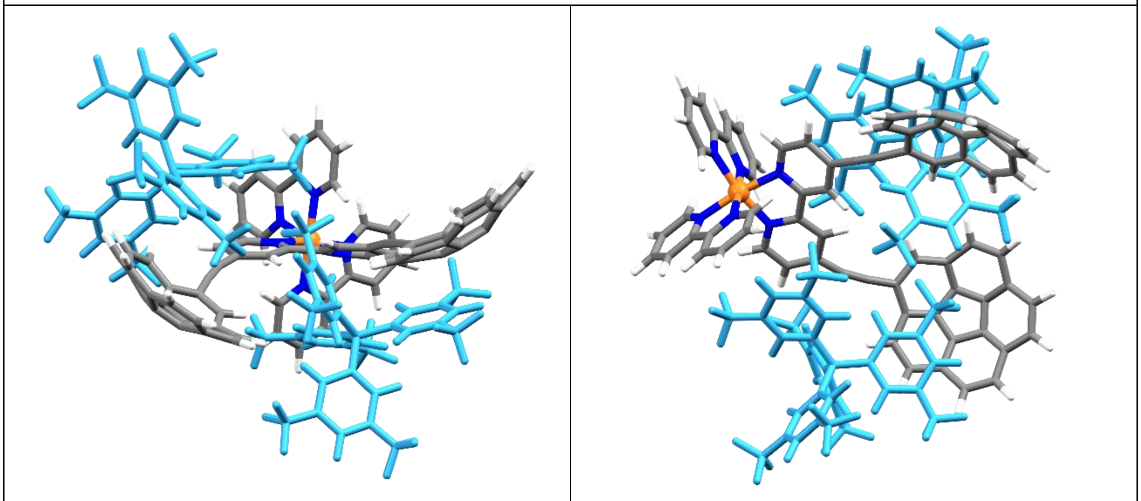

Ru	-0.026021	5.726036	-0.448143	H	2.147503	3.496533	-4.152213	H	-3.863442	-5.907058	0.969330
N	-1.046913	3.936699	-0.741741	F	3.436758	-5.870677	-1.426039	C	-5.927863	-3.257294	1.492688
N	-1.706692	6.925345	-0.591980	C	4.496496	1.779049	-2.398415	H	-6.949762	-3.065086	1.809366
N	0.571834	5.902475	-2.417734	H	5.530169	1.905170	-2.097198	C	-5.503083	-4.581163	1.405297
N	1.269759	7.337230	-0.347450	F	8.655328	-2.052452	-6.159451	F	-10.167748	2.327695	2.398228
N	-0.684007	5.751697	1.524878	C	7.972595	0.032505	1.311449	C	-7.468954	-0.572868	-3.062601
C	1.533685	6.825751	-2.661208	H	8.800557	0.204095	1.988784	H	-7.870029	-0.579160	-4.068644
N	1.473433	4.346593	-0.000066	F	1.294420	-0.638589	2.574888	F	-1.269540	-4.670458	1.517709
C	0.083390	5.182900	-3.435861	F	9.212138	-2.609010	-4.146465	F	-6.445890	-2.107750	6.040316
H	-0.665854	4.445098	-3.183477	C	6.133157	-2.669015	-5.302069	C	-5.804439	1.173493	-3.730440
C	-1.822491	6.455901	1.744968	H	6.489664	-3.123273	-6.218551	F	-7.113685	-5.530983	2.838198
C	1.957952	7.598260	-1.487080	C	3.051116	-5.490942	-0.193692	F	-1.846757	-5.122459	-0.506827
C	0.527535	5.342526	-4.740584	F	3.263030	-4.577276	-5.007627	F	-8.988316	-3.527575	-1.838890
H	0.111870	4.715304	-5.521487	C	1.850896	-3.498005	0.714911	F	-10.302656	-1.818395	-1.917267

C	1.525893	6.274762	-4.999202	H	1.179381	-4.155129	1.253668	F	-11.272899	0.954007	3.650789
C	-2.335876	0.093320	-1.865974	C	0.641667	1.287380	-4.115533	C	-10.375001	1.020076	2.653967
C	-0.104945	5.113645	2.552350	F	8.015462	-3.535722	1.192816	F	-1.208162	-3.117789	0.013633
H	0.788741	4.547153	2.325388	F	0.076293	0.108759	-3.849847	F	-9.307210	-2.489550	-3.714902
C	-2.169958	7.476402	-1.723635	B	4.601869	-0.758393	-1.549105	F	-10.952311	0.504208	1.556274
H	-1.566897	7.312883	-2.609489	F	8.879865	-0.533430	-4.631296	C	-6.502091	-5.667408	1.651548
C	-2.263886	3.771479	-1.282902	F	2.822827	-2.813974	-6.167532	F	-5.954310	-6.892408	1.616370
H	-2.832885	4.675098	-1.460688	C	8.664413	-2.363138	1.112493	C	-9.147318	-2.311662	-2.394125
C	2.031607	7.024423	-3.945831	F	0.784595	1.385017	-5.447310	C	4.261057	0.539673	2.671182
H	2.811973	7.753661	-4.124971	C	3.829756	-3.590667	-5.728181	C	7.349850	-3.434057	4.906850
C	-2.408479	7.087551	0.555964	C	8.432021	-1.780413	-4.862283	C	6.711124	-2.546252	4.014159
C	2.701516	4.618606	0.474333	F	8.543090	2.639633	1.972424	C	7.965437	-2.662599	5.919752
H	3.064379	5.626139	0.311318	F	4.398326	-4.155797	-6.803862	C	6.943615	-1.224877	4.463436
C	-0.312640	2.830877	-0.470024	C	7.307011	2.439681	1.499803	C	7.718711	-1.297222	5.643434
C	-3.352537	8.195961	-1.774089	C	0.709207	-1.509545	1.725158	C	6.910089	-4.728532	5.109033
H	-3.688293	8.613635	-2.716515	F	5.875214	4.100652	-3.015312	C	5.610688	-2.905309	3.259839
C	-0.616405	5.152307	3.840669	F	9.296416	-2.168337	2.283238	C	8.179693	-3.139544	7.198147
H	-0.129575	4.585418	4.624789	F	4.311205	4.759752	-4.361113	C	6.097369	-0.173613	4.189444
C	2.984603	8.540912	-1.504469	F	0.053645	-2.403725	2.460306	C	7.678159	-0.326301	6.625260
H	3.549278	8.715014	-2.412280	C	4.552208	4.168092	-3.172703	C	5.914879	-5.178822	4.164954
C	2.554459	8.991128	0.806961	F	6.454951	2.729206	2.515721	C	7.325846	-5.306851	6.362667
H	2.753902	9.519280	1.732511	F	4.095527	5.045660	-2.235061	C	5.295988	-4.313354	3.281044
C	1.556737	8.030520	0.763666	F	9.616769	-2.508128	0.174115	C	4.832921	-1.802429	2.782700
H	0.969418	7.778397	1.639590	F	7.072256	3.382775	0.562294	C	7.927952	-4.551432	7.355580
C	-3.612935	7.788983	0.568525	F	-0.221834	-0.784246	1.050737	C	8.353504	-2.112344	8.196126
H	-4.189264	7.865040	1.481478	C	-4.785230	0.578825	1.412439	C	5.065241	-0.487153	3.210267
C	-2.371903	6.554574	3.021590	F	-1.275019	0.304152	3.598572	C	6.211335	0.943541	5.088738
H	-3.279659	7.120451	3.184194	F	-8.349444	-1.317746	6.709102	C	8.116954	-0.775230	7.924097
C	3.290736	9.242203	-0.347326	C	-6.933450	-0.518213	2.221296	C	6.964194	0.869015	6.249343
C	3.631374	1.438984	2.144842	C	-6.398005	-0.579309	-0.418140	H	5.574437	-6.210520	4.199236
C	1.004307	3.081273	0.124788	C	-3.565645	0.504320	2.097152	H	7.079673	-6.343234	6.579687
C	3.467922	3.696360	1.160142	H	-3.150396	-0.464372	2.355684	H	4.507250	-4.695383	2.641099
H	4.456634	3.957842	1.518037	C	-4.588913	3.018443	1.548029	H	3.994133	-1.973248	2.116376
C	-4.092469	8.348144	-0.605496	C	-8.164254	0.095012	1.973751	H	8.130114	-5.025453	8.312966
C	-0.754328	1.563387	-0.792663	H	-8.419100	0.398153	0.961207	H	8.589045	-2.397126	9.218662
H	-0.128909	0.700878	-0.610719	C	-3.375365	2.919399	2.221765	H	5.627360	1.838669	4.897856
C	-2.779752	2.536624	-1.628972	H	-2.852163	3.805753	2.560923	H	8.176856	-0.062896	8.743298
H	-3.749552	2.482687	-2.105679	C	-3.779105	-2.501617	0.807866	H	6.937299	1.712141	6.935376
C	-1.767133	5.893128	4.081470	H	-3.083407	-1.718566	0.520611	C	-2.363096	-1.048146	-2.293231
C	1.689735	2.117260	0.839073	C	-5.888539	0.246314	-1.423335	C	-3.110427	-6.784479	-3.092202
H	1.219887	1.166249	1.036400	H	-5.048463	0.893045	-1.193014	C	-3.927518	-5.629231	-3.104756
C	2.942196	2.410944	1.400111	C	-2.878839	1.650836	2.499511	C	-1.781478	-6.388175	-3.372872
C	-2.002258	1.385091	-1.417114	C	-5.272509	1.871680	1.159422	C	-3.099302	-4.512008	-3.375124
C	5.201754	-1.485032	-2.887834	H	-6.239517	1.982921	0.677389	C	-1.774091	-4.983684	-3.540819
C	3.564443	-1.765312	-0.778385	C	-7.601842	-0.675864	4.566198	C	-3.410912	-7.901314	-2.333541
C	3.691373	-3.161607	-0.828339	F	-4.532187	0.841014	-4.017984	C	-5.094345	-5.533527	-2.370081
H	4.451559	-3.605431	-1.465046	F	-0.561557	2.086666	2.597057	C	-0.679628	-7.085882	-2.912086
C	2.554999	-1.278626	0.059563	C	-8.823298	-0.062512	4.295996	C	-3.402886	-3.246761	-2.911270
H	2.440137	-0.203849	0.142858	H	-9.549845	0.098454	5.083603	C	-0.657676	-4.212365	-3.263007
C	5.804004	-0.421975	-0.490822	C	-3.334991	-3.822961	0.772163	C	-4.723049	-7.890127	-1.737782
C	6.528439	-1.348878	-3.310150	C	-7.472310	-1.402839	-0.790587	C	-2.282348	-8.748718	-2.040880
H	7.227021	-0.778107	-2.704292	H	-7.905367	-2.076511	-0.056152	C	-5.527752	-6.764305	-1.758823
C	2.540246	0.455624	-2.679242	C	-6.679404	-0.893797	3.549287	C	-5.533123	-4.187005	-2.104170
H	1.995063	-0.474811	-2.552171	H	-5.737545	-1.379626	3.791352	C	-0.982331	-8.361795	-2.317244
C	3.842172	0.560022	-2.160267	F	-4.616281	4.910688	0.119574	C	0.543773	-6.324692	-2.854411
C	4.357412	-2.219659	-3.729181	C	-5.096362	-2.175854	1.174478	C	-4.723368	-3.095609	-2.358459
H	3.311141	-2.343157	-3.458992	C	-6.414729	0.260363	-2.716996	C	-2.251362	-2.371809	-2.778706
C	1.945899	1.481949	-3.408921	C	-1.607896	1.553866	3.281079	C	0.557406	-4.954071	-3.022189
C	7.735005	-1.233230	0.782867	C	-7.256237	-1.040101	5.977698	C	-0.941391	-2.849111	-2.954491
F	-0.263717	2.247235	-3.779579	F	-1.674539	2.260936	4.430717	H	-5.058550	-8.753629	-1.168581
C	1.718974	-2.117274	0.800254	F	-6.478397	4.342073	1.045630	H	-2.441731	-9.678209	-1.499497
C	7.111789	1.060899	0.955142	C	-5.160009	4.363967	1.240841	H	-6.460177	-6.780407	-1.203509
F	4.015906	-5.911413	0.652083	B	-5.764452	-0.686614	1.086807	H	-6.478832	-4.023990	-1.596927
C	2.849312	-4.008241	-0.108945	F	-4.914735	5.260907	2.226295	H	-0.167841	-8.999218	-1.982524
C	3.874569	2.841393	-3.051958	C	-9.094428	0.320286	2.990549	H	1.468409	-6.817053	-2.571186
C	6.039775	0.824987	0.093246	F	-6.480424	1.201811	-4.884987	H	-5.056311	-2.116185	-2.034251
H	5.375043	1.653464	-0.120935	C	-1.919025	-4.174971	0.440625	H	1.495062	-4.424814	-2.878608
C	6.682548	-1.446809	-0.102503	F	-5.752160	2.451830	-3.272189	H	-0.127182	-2.169977	-2.721440
H	6.540231	-2.447591	-0.502178	F	-6.618039	-0.031440	6.605168	H	1.914719	6.409889	-6.002675
F	1.940917	-6.174408	0.113524	C	-7.990592	-1.410864	-2.081006	H	4.092992	9.972512	-0.346479
C	6.986448	-1.925600	-4.494553	F	-7.479833	-5.646851	0.716200	H	-5.035111	8.884915	-0.610383
C	4.808074	-2.808809	-4.906196	C	-4.197665	-4.879045	1.048914	H	-2.199922	5.941205	5.074930
C	2.600101	2.696359	-3.581101								

Ru1C·BAr$^{F}{}_{4}$ – v$_{4}$ E = -10551.5617654 a. u

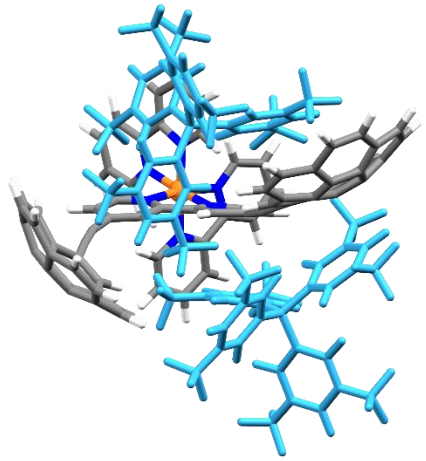

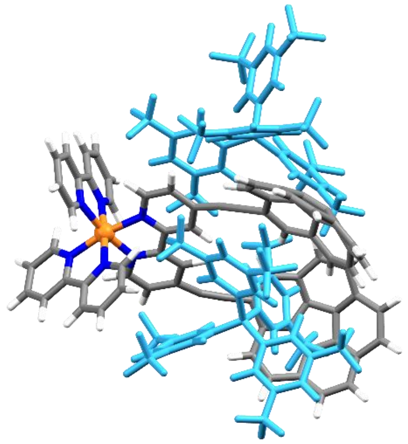

Ru	-2.625477	5.471661	-0.771838	H	-5.301359	2.614431	1.353326	H	9.410223	-1.441340	-1.572862
N	-1.393552	5.018514	0.834165	F	-2.135214	-4.587770	6.183372	C	6.908367	0.871794	-1.559819
N	-2.359894	7.518724	-0.615785	C	-5.395372	-0.524592	0.052710	H	6.782490	1.937200	-1.729958
N	-4.402464	5.346200	0.279997	H	-6.003979	-1.110954	-0.628822	C	8.191423	0.332427	-1.677936
N	-4.020865	5.614663	-2.293960	F	-8.958385	-6.763176	0.187784	F	1.550086	5.527160	0.247651
N	-0.916765	5.759950	-1.914997	C	-3.327898	-4.008362	-3.663529	C	2.964368	-0.656899	3.013824
C	-5.524935	5.268749	-0.476544	H	-3.158239	-4.327108	-4.683898	H	2.675689	-0.992910	4.002798
N	-2.520635	3.399212	-0.928420	F	1.372419	-3.867371	1.645754	F	7.187721	-3.974512	-2.084229
C	-4.508397	5.299373	1.614205	F	-7.065656	-7.610640	0.791890	F	5.819985	4.290378	-4.530915
H	-3.578678	5.350787	2.165814	C	-8.044824	-4.680947	1.726874	C	0.609544	-0.879981	2.302001
C	-0.425196	7.021783	-1.923943	H	-8.983408	-5.139823	2.014060	F	9.708340	1.947831	-0.884242
C	-5.304054	5.377399	-1.924149	C	-2.717710	-3.631621	5.440189	F	8.781458	-3.628635	-0.666485
C	-5.727645	5.154045	2.259411	F	-7.771421	-2.292376	4.327076	F	4.810970	-0.730860	4.991437
H	-5.755364	5.093092	3.341344	C	-1.183014	-4.314003	3.603014	F	6.253358	-1.322947	3.493063
C	-6.882823	5.051867	1.492453	H	-0.495957	-4.714050	4.339641	F	3.392525	6.562597	0.673969
C	0.790562	3.508398	4.089353	C	-3.457770	1.725128	3.079651	C	2.854459	5.333394	0.589850
C	-0.270107	4.801914	-2.593549	F	-1.503632	-6.523603	-2.058234	F	6.731770	-3.794283	0.017549
H	-0.704515	3.813464	-2.556170	F	-2.294963	1.229817	3.512072	F	5.987656	0.766151	3.952120
C	-3.171698	8.357714	0.045143	B	-4.128768	-2.771947	0.580988	F	2.852963	4.805800	1.819863
H	-4.064179	7.910899	0.468959	F	-7.247184	-6.659295	-1.136871	C	9.338010	1.249911	-1.974522
C	-0.968864	5.858962	1.791137	F	-9.129703	-1.733674	2.748284	F	9.020606	2.153999	-2.917941
H	-1.259517	6.897362	1.672052	C	-2.558711	-6.249728	-2.843485	C	5.329536	-0.397385	3.800439
C	-6.778264	5.117270	0.109895	F	-4.298198	1.748664	4.127231	C	-0.515013	-1.504864	-1.931595
H	-7.664351	5.027046	-0.505082	C	-8.489866	-2.784425	3.302170	C	2.534125	-5.930653	-3.156834
C	-1.225878	8.003853	-1.181654	C	-7.626930	-6.571432	0.148084	C	1.663282	-5.004679	-2.542073
C	-3.118581	2.624841	-1.849789	F	-4.070272	-2.299307	-5.678226	C	2.091466	-6.124909	-4.487085
H	-3.892561	3.093538	-2.443332	F	-9.434382	-3.585843	3.820823	C	0.676386	-4.628658	-3.486733
C	-1.037098	3.713831	0.912301	C	-4.012274	-1.737633	-4.465251	C	0.942874	-5.322169	-4.690415
C	-2.891961	9.705929	0.196053	C	0.466537	-4.860644	1.821501	C	3.858868	-6.059196	-2.779828
H	-3.579301	10.340842	0.743663	F	-7.461585	0.625647	-1.342623	C	2.057734	-4.183170	-1.503285
C	0.886063	5.048251	-3.318687	F	-2.172636	-6.430114	-4.117041	C	2.949035	-6.468718	-5.514397
H	1.370819	4.241042	-3.854850	F	-7.621655	2.191146	0.142729	C	0.022781	-3.416297	-3.451966
C	-6.311114	5.236277	-2.877068	F	0.389939	-5.522002	0.659462	C	0.583188	-4.829092	-5.929900
H	-7.322455	5.000266	-2.570561	C	-6.751633	1.481440	-0.608362	C	4.218110	-5.365385	-1.566856
C	-4.688249	5.646539	-4.588936	F	-3.026896	-0.795871	-4.527726	C	4.715230	-6.624918	-3.790509
H	-4.400790	5.758070	-5.628265	F	-6.213678	2.385542	-1.478186	C	3.356966	-4.476184	-0.950893
C	-3.729467	5.758054	-3.594601	F	-3.493822	-7.178311	-2.574005	C	1.220490	-3.043245	-1.299646
H	-2.686532	5.942667	-3.826315	F	-5.149809	-1.040822	-4.280874	C	4.281167	-6.821803	-5.090216
C	-0.884464	9.350451	-1.057157	F	0.998125	-5.694598	2.730401	C	2.460630	-6.153957	-6.834094
H	0.032289	9.721994	-1.498596	C	3.372075	0.044995	-2.342049	C	0.226778	-2.670421	-2.216915
C	0.734264	7.335369	-2.629634	F	4.876846	-2.120238	-5.667797	C	-0.524513	-2.983722	-4.708669
H	1.118002	8.348668	-2.631634	F	4.922792	6.164770	-3.909847	C	1.333744	-5.376487	-7.032411
C	-6.001891	5.368042	-4.222989	C	4.177535	2.254655	-1.217654	C	-0.261454	-3.662343	-5.887465
C	-1.148224	-0.502608	-1.646167	C	3.736913	0.159283	0.392524	H	5.224007	-5.464177	-1.172380
C	-1.587345	2.831049	-0.123822	C	3.779683	-1.022672	-3.150856	H	5.761420	-6.807532	-3.558837
C	-2.760816	1.313819	-2.084036	H	4.673901	-1.582392	-2.896634	H	3.703393	-3.927470	-0.079306
H	-3.231370	0.758155	-2.884397	C	1.544605	0.377253	-3.939582	H	1.378680	-2.406546	-0.438679
C	-1.719851	10.210478	-0.360423	C	3.662725	3.096819	-0.223864	H	5.003447	-7.157408	-5.830429
C	-0.270279	3.239182	1.957850	H	3.347083	2.669950	0.723998	H	3.044222	-6.449924	-7.702407
H	-0.018861	2.192002	2.031425	C	1.985378	-0.676042	-4.734612	H	-1.078253	-2.052664	-4.751953
C	-0.224521	5.450406	2.881812	H	1.472077	-0.944634	-5.649787	H	1.074390	-5.089929	-8.048562
H	0.069312	6.164938	3.641318	C	6.061216	-1.276484	-1.000811	H	-0.629428	-3.235696	-6.817656
C	1.397672	6.340883	-3.335615	H	5.243542	-1.928158	-0.702783	C	1.251937	2.723837	4.897988
C	-1.178512	1.520509	-0.293624	C	2.412987	-0.185349	0.695366	C	4.765496	-0.792783	7.982812
H	-0.408758	1.096976	0.335210	H	1.667714	-0.169557	-0.097528	C	4.679231	0.590529	7.690740
C	-1.716405	0.741415	-1.334002	C	3.105828	-1.376795	-4.319051	C	3.481843	-1.361558	7.801348
C	0.124948	4.096574	2.992145	C	2.217165	0.720638	-2.769124	C	3.341859	0.874752	7.321416
C	-5.577181	-3.471004	0.959844	H	1.857050	1.569954	-2.194416	C	2.603588	-0.331956	7.391858
C	-3.015195	-3.324504	1.654482	C	4.432847	4.245804	-2.620545	C	5.928471	-1.518862	7.802872
C	-3.307791	-3.318226	3.027887	F	-0.090743	-1.296653	1.236566	C	5.751645	1.317651	7.207902
H	-4.274500	-2.957774	3.367717	F	3.871593	-3.592890	-4.458603	C	3.290113	-2.683179	7.438635

C	-1.750242	-3.814441	1.304141	C	3.937910	5.063664	-1.611572	C	3.015048	1.904225	6.461652
H	-1.441819	-3.816714	0.263145	H	3.864120	6.135765	-1.749972	C	1.478215	-0.575990	6.622869
C	-3.756222	-3.167079	-0.961420	C	7.331529	-1.825154	-1.133331	C	7.097652	-0.727115	7.517104
C	-6.028104	-4.667305	0.386521	C	4.644766	0.111546	1.460909	C	5.735553	-2.938354	7.646441
H	-5.441424	-5.150355	-0.388604	H	5.682888	0.377762	1.282153	C	7.013721	0.623940	7.233423
C	-3.679513	-0.364655	1.695128	C	4.537849	2.871044	-2.427426	C	5.397920	2.517807	6.493763
H	-2.879715	-0.802615	2.284487	H	4.899237	2.253249	-3.246038	C	4.479906	-3.493764	7.474107
C	-4.359960	-1.158999	0.764404	F	-0.521749	0.540655	-5.083355	C	2.009019	-2.970691	6.839459
C	-6.421222	-2.901719	1.923845	C	5.802144	0.085575	-1.230173	C	4.092858	2.798091	6.133449
H	-6.141812	-1.962442	2.394161	C	2.035483	-0.583194	1.980967	C	1.734465	1.743971	5.797734
C	-4.033503	0.968282	1.925277	C	3.677921	-2.471919	-5.166625	C	1.138532	-1.969003	6.453404
C	-3.072761	-4.863029	-2.599485	C	4.740887	4.828856	-3.962291	C	1.002928	0.550472	5.881285
F	-3.234345	3.031214	2.763809	F	2.888738	-2.774265	-6.209281	H	8.062069	-1.218724	7.417124
C	-0.865717	-4.330337	2.253205	F	-0.181442	1.910013	-3.440575	H	6.604108	-3.586290	7.558928
C	-3.771194	-2.724655	-3.371671	C	0.430345	1.229004	-4.440333	H	7.914228	1.145390	6.919406
F	-2.257802	-2.448281	5.916463	B	4.266040	0.625783	-1.083330	H	6.186538	3.169381	6.126455
C	-2.407208	-3.776723	3.984411	F	0.866423	2.178784	-5.301181	H	4.407138	-4.556845	7.259505
C	-5.697080	0.822388	0.218341	C	3.536268	4.472737	-0.420453	H	1.751335	-3.999437	6.600325
C	-3.957413	-2.315560	-2.052958	F	0.468666	-1.776060	3.281255	H	3.895071	3.656352	5.496812
H	-4.282092	-1.294936	-1.881330	C	7.513955	-3.301586	-0.962644	H	0.219382	-2.233266	5.940072
C	-3.298092	-4.454654	-1.286767	F	-0.035905	0.241827	2.740231	H	0.110705	0.460926	5.268979
H	-3.098983	-5.166919	-0.490346	F	3.707421	4.631986	-4.823218	H	-7.850828	4.909279	1.960399
F	-4.031986	-3.659962	5.694787	C	4.275794	-0.298710	2.739072	H	-6.774071	5.247839	-4.975435
C	-7.231153	-5.261807	0.760385	F	10.424209	0.586066	-2.402895	H	-1.460942	11.258776	-0.254856
C	-7.625220	-3.490637	2.305389	C	8.416008	-1.021904	-1.475243	H	2.303131	6.564835	-3.888374
C	-5.027652	1.581287	1.174144								

Table S 13. Summary of the interaction energies between cationic complex **Ru1C** and BAr^F_4 anions computed with the counterpoise scheme.[39–42] [a]E_{int} (total) = E_{int} (BAr^F_4) + E_{int} (BAr^F_4').

Complex	E_{int} (BAr^F_4) /kcalmol^{-1}	E_{int} (BAr^F_4') /kcalmol^{-1}	E_{int} (total)[a]/kcalmol^{-1}
**Ru1C·BAr^F_4 - v_1**	-100.34	-93.29	-193.63
**Ru1C·BAr^F_4 - v_2**	-87.71	-81.24	-168.95
**Ru1C·BAr^F_4 - v_3**	-92.09	-98.56	-190.65
**Ru1C·BAr^F_4 - v_4**	-102.75	-91.33	-194.08

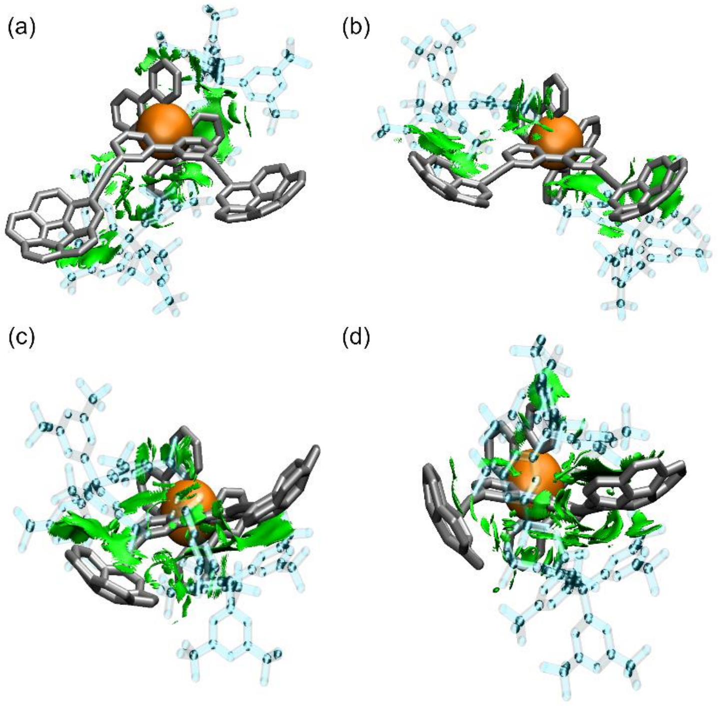

Figure S 170. NCI plot (isovalue = 0.3 a.u.) of the species (a) **Ru1C·BAr^F_4 - v_1**, (b) **Ru1C·BAr^F_4 - v_2**, (c) **Ru1C·BAr^F_4 - v_3** and (d) **Ru1C·BAr^F_4 - v_4**. Hydrogen atoms have been removed for clarity purposes.

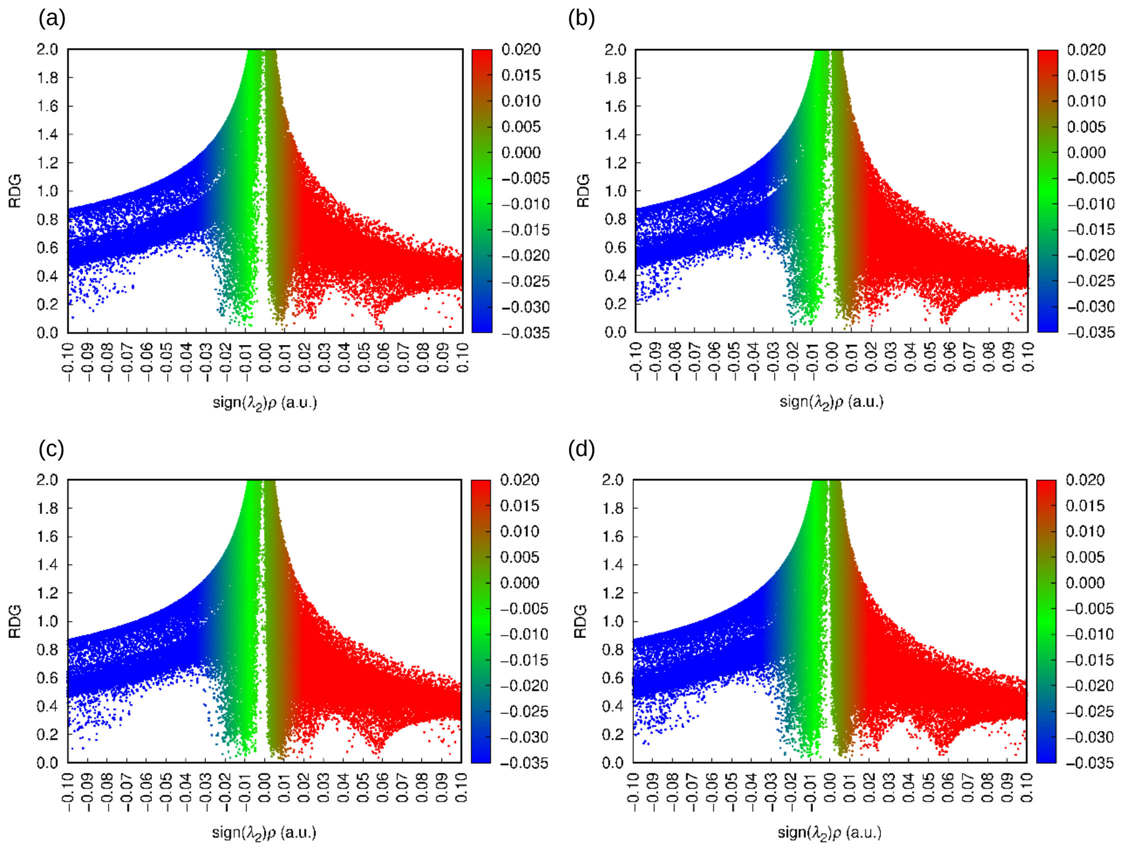

Figure S 171. Plot of the reduced density gradient versus the electron density multiplied by the sign of the second Hessian eigenvalue (λ_2) of species (a) **Ru1C·BAr^F$_4$ - v$_1$**, (b) **Ru1C·BAr^F$_4$ - v$_2$**, (c) **Ru1C·BAr^F$_4$ - v$_3$** and (d) **Ru1C·BAr^F$_4$ - v$_4$**.

References

(1) Williams, D. B. G.; Lawton, M. Drying of Organic Solvents: Quantitative Evaluation of the Efficiency of Several Desiccants. *J. Org. Chem.* **2010**, *75*, 8351–8354.

(2) Armarego, W. L. F.; Chai, C. L. L. *Purification of Laboratory Chemicals*, 7th arg.; Elsevier, 2013.

(3) Doi, T.; Nagamiya, H.; Kokubo, M.; Hirabayashi, K.; Takahashi, T. Synthesis of a tetrabenzyl-substituted 10-membered cyclic diamide. *Tetrahedron* **2002**, *58*, 2957–2963.

(4) Sacristán-Martín, A.; Barbero, H.; Ferrero, S.; Miguel, D.; García-Rodríguez, R.; Álvarez, C. M. ON/OFF metal-triggered molecular tweezers for fullerene recognition. *Chem. Commun.* **2021**, *57*, 11013–11016.

(5) Jones, C. S.; Elliott, E.; Siegel, J. S. Synthesis and Properties of Monosubstituted Ethynylcorannulenes. *Synlett* **2004**, Zenb. 1, 187–191.

(6) Chen, Y.-Y.; Wang, H.; Zhang, D.-W.; Hou, J.-L.; Li, Z.-T. Organogels formed by substituent-free pyrene-appended oligo(m-phenylene ethynylene)s. *Chem. Commun.* **2015**, *51*, 12088–12091.

(7) Norris, M. R.; Concepcion, J. J.; Glasson, C. R. K.; Fang, Z.; Lapides, A. M.; Ashford, D. L.; Templeton, J. L.; Meyer, T. J. Synthesis of Phosphonic Acid Derivatized Bipyridine Ligands and Their Ruthenium Complexes. *Inorg. Chem.* **2013**, *52*, 12492–12501.

(8) Merillas, B.; Cuéllar, E.; Diez-Varga, A.; Asensio-Bartolomé, M.; García-Herbosa, G.; Torroba, T.; Martín-Alvarez, J. M.; Miguel, D.; Villafañe, F. Whole microwave syntheses of pyridylpyrazole and of Re and Ru luminescent pyridylpyrazole complexes. *Inorganica Chim. Acta* **2019**, *484*, 1–7.

(9) Johnson, E. C.; Sullivan, B. P.; Salmon, D. J.; Adeyemi, S. A.; Meyer, T. J. Synthesis and properties of the chloro-bridged dimer [(bpy)2RuCl]22+ and its transient 3+ mixed-valence ion. *Inorg. Chem.* **1978**, *17*, 2211–2215.

(10) Ashford, D. L.; Brennaman, M. K.; Brown, R. J.; Keinan, S.; Concepcion, J. J.; Papanikolas, J. M.; Templeton, J. L.; Meyer, T. J. Varying the Electronic Structure of Surface-Bound Ruthenium(II) Polypyridyl Complexes. *Inorg. Chem.* **2015**, *54*, 460–469.

(11) Nakagawa, A.; Sakuda, E.; Ito, A.; Kitamura, N. Remarkably Intense Emission from Ruthenium(II) Complexes with Multiple Borane Centers. *Inorg. Chem.* **2015**, *54*, 10287–10295.

(12) Boudreau, L. J.; Clarke, T. L.; Murray, A. H.; Robertson, K. N.; Cameron, T. S.; Aquino, M. A. S. Sterically-directed disassembly of diruthenium(II,III) tetraacetate using substituted and unsubstituted 2,2′-bipyridines and 1,10-phenanthrolines. *Inorganica Chim. Acta* **2013**, *394*, 152–158.

(13) Klemm, E.; Prautsch, T.; Gorls, H. *CSD Communication (Private Communication),* **2023**.

(14) Howe, E. N. W.; Bhadbhade, M.; Thordarson, P. Cooperativity and complexity in the binding of anions and cations to a tetratopic ion-pair host. *J. Am. Chem. Soc.* **2014**, *136*, 7505–7516.

(15) Brynn Hibbert, D.; Thordarson, P. The death of the Job plot, transparency, open science and online tools, uncertainty estimation methods and other developments in supramolecular chemistry data analysis. *Chem. Commun.* **2016**, *52*, 12792–12805.

(16) Hunter, C. A.; Anderson, H. L. What is Cooperativity? *Angew. Chemie Int. Ed.* **2009**, *48*, 7488–7499.

(17) Pramanik, S.; Thordarson, P.; Day, V. W.; Bowman-James, K. Oligomeric phosphate clusters in macrocyclic channels. *CrystEngComm* **2022**, *24*, 8047–8051.

(18) Hu, W.; Xie, J.; Chau, H. W.; Si, B. C. Evaluation of parameter uncertainties in nonlinear regression using Microsoft Excel Spreadsheet. *Environ. Syst. Res.* **2015**, *4*.

(19) Genovese, D.; Cingolani, M.; Rampazzo, E.; Prodi, L.; Zaccheroni, N. Static quenching upon adduct formation: a treatment without shortcuts and approximations. *Chem. Soc. Rev.* **2021**, *50*, 8414–8427.

(20) Yanai, T.; Tew, D. P.; Handy, N. C. A new hybrid exchange–correlation functional using the Coulomb-attenuating method (CAM-B3LYP). *Chem. Phys. Lett.* **2004**, *393*, 51–57.

(21) Perdew, J. P.; Burke, K.; Ernzerhof, M. Generalized Gradient Approximation Made Simple. *Phys. Rev. Lett.* **1996**, *77*, 3865–3868.

(22) Perdew, J. P.; Burke, K.; Ernzerhof, M. Generalized Gradient Approximation Made Simple [Phys. Rev. Lett. 77, 3865 (1996)]. *Phys. Rev. Lett.* **1997**, *78*, 1396–1396.

(23) Adamo, C.; Barone, V. Toward reliable density functional methods without adjustable parameters: The PBE0 model. *J. Chem. Phys.* **1999**, *110*, 6158–6170.

(24) Ernzerhof, M.; Scuseria, G. E. Assessment of the Perdew–Burke–Ernzerhof exchange-correlation functional. *J. Chem. Phys.* **1999**, *110*, 5029–5036.

(25) Tao, J.; Perdew, J. P.; Staroverov, V. N.; Scuseria, G. E. Climbing the Density Functional Ladder: Nonempirical Meta–Generalized Gradient Approximation Designed for Molecules and Solids. *Phys. Rev. Lett.* **2003**, *91*,

146401.

(26) Staroverov, V. N.; Scuseria, G. E.; Tao, J.; Perdew, J. P. Comparative assessment of a new nonempirical density functional: Molecules and hydrogen-bonded complexes. *J. Chem. Phys.* **2003**, *119*, 12129–12137.

(27) Chai, J.-D.; Head-Gordon, M. Long-range corrected hybrid density functionals with damped atom–atom dispersion corrections. *Phys. Chem. Chem. Phys.* **2008**, *10*, 6615.

(28) Ditchfield, R.; Hehre, W. J.; Pople, J. A. Self-Consistent Molecular-Orbital Methods. IX. An Extended Gaussian-Type Basis for Molecular-Orbital Studies of Organic Molecules. *J. Chem. Phys.* **1971**, *54*, 724–728.

(29) Hehre, W. J.; Ditchfield, K.; Pople, J. A. Self-consistent molecular orbital methods. XII. Further extensions of gaussian-type basis sets for use in molecular orbital studies of organic molecules. *J. Chem. Phys.* **1972**, *56*, 2257–2261.

(30) Francl, M. M.; Pietro, W. J.; Hehre, W. J.; Binkley, J. S.; Gordon, M. S.; DeFrees, D. J.; Pople, J. A. Self-consistent molecular orbital methods. XXIII. A polarization-type basis set for second-row elements. *J. Chem. Phys.* **1982**, *77*, 3654–3665.

(31) Petersson, G. A.; Al-Laham, M. A. A complete basis set model chemistry. II. Open-shell systems and the total energies of the first-row atoms. *J. Chem. Phys.* **1991**, *94*, 6081–6090.

(32) Hay, P. J.; Wadt, W. R. Ab initio effective core potentials for molecular calculations. Potentials for the transition metal atoms Sc to Hg. *J. Chem. Phys.* **1985**, *82*, 270–283.

(33) Hay, P. J.; Wadt, W. R. Ab initio effective core potentials for molecular calculations. Potentials for K to Au including the outermost core orbitals. *J. Chem. Phys.* **1985**, *82*, 299–310.

(34) Grimme, S.; Ehrlich, S.; Goerigk, L. Effect of the damping function in dispersion corrected density functional theory. *J. Comput. Chem.* **2011**, *32*, 1456–1465.

(35) Bannwarth, C.; Ehlert, S.; Grimme, S. GFN2-xTB—An Accurate and Broadly Parametrized Self-Consistent Tight-Binding Quantum Chemical Method with Multipole Electrostatics and Density-Dependent Dispersion Contributions. *J. Chem. Theory Comput.* **2019**, *15*, 1652–1671.

(36) Bannwarth, C.; Caldeweyher, E.; Ehlert, S.; Hansen, A.; Pracht, P.; Seibert, J.; Spicher, S.; Grimme, S. Extended tight-binding quantum chemistry methods. *WIREs Comput. Mol. Sci.* **2021**, *11*, 1–49.

(37) Tomasi, J.; Mennucci, B.; Cammi, R. Quantum Mechanical Continuum Solvation Models. *Chem. Rev.* **2005**, *105*, 2999–3094.

(38) Marenich, A. V.; Cramer, C. J.; Truhlar, D. G. Universal Solvation Model Based on Solute Electron Density and on a Continuum Model of the Solvent Defined by the Bulk Dielectric Constant and Atomic Surface Tensions. *J. Phys. Chem. B* **2009**, *113*, 6378–6396.

(39) Boys, S. F.; Bernardi, F. The calculation of small molecular interactions by the differences of separate total energies. Some procedures with reduced errors. *Mol. Phys.* **1970**, *19*, 553–566.

(40) Simon, S.; Duran, M.; Dannenberg, J. J. How does basis set superposition error change the potential surfaces for hydrogen-bonded dimers? *J. Chem. Phys.* **1996**, *105*, 11024–11031.

(41) van Duijneveldt, F. B.; van Duijneveldt-van de Rijdt, J. G. C. M.; van Lenthe, J. H. State of the Art in Counterpoise Theory. *Chem. Rev.* **1994**, *94*, 1873–1885.

(42) Jensen, F. An Atomic Counterpoise Method for Estimating Inter- and Intramolecular Basis Set Superposition Errors. *J. Chem. Theory Comput.* **2010**, *6*, 100–106.

(43) Gaussian 16, R. C., M. J. Frisch, G. W. Trucks, H. B. Schlegel, G. E. Scuseria, M. A. Robb, J. R. Cheeseman, G. Scalmani, V. Barone, G. A. Petersson, H. Nakatsuji, X. Li, M. Caricato, A. V. Marenich, J. Bloino, B. G. Janesko, R. Gomperts, B. Mennucci, H. P. Hratchian, J. V. Ortiz, A. F. Izmaylov, J. L. Sonnenberg, D. Williams-Young, F. Ding, F. Lipparini, F. Egidi, J. Goings, B. Peng, A. Petrone, T. Henderson, D. Ranasinghe, V. G. Zakrzewski, J. Gao, N. Rega, G. Zheng, W. Liang, M. Hada, M. Ehara, K. Toyota, R. Fukuda, J. Hasegawa, M. Ishida, T. Nakajima, Y. Honda, O. Kitao, H. Nakai, T. Vreven, K. Throssell, J. A. Montgomery, Jr., J. E. Peralta, F. Ogliaro, M. J. Bearpark, J. J. Heyd, E. N. Brothers, K. N. Kudin, V. N. Staroverov, T. A. Keith, R. Kobayashi, J. Normand, K. Raghavachari, A. P. Rendell, J. C. Burant, S. S. Iyengar, J. Tomasi, M. Cossi, J. M. Millam, M. Klene, C. Adamo, R. Cammi, J. W. Ochterski, R. L. Martin, K. Morokuma, O. Farkas, J. B. Foresman, and D. J. Fox, Gaussian, Inc., Wallingford CT

(44) Johnson, E. R.; Keinan, S.; Mori-Sánchez, P.; Contreras-García, J.; Cohen, A. J.; Yang, W. Revealing Noncovalent Interactions. *J. Am. Chem. Soc.* **2010**, *132*, 6498–6506.

(45) Contreras-García, J.; Johnson, E. R.; Keinan, S.; Chaudret, R.; Piquemal, J.-P.; Beratan, D. N.; Yang, W. NCIPLOT: A Program for Plotting Noncovalent Interaction Regions. *J. Chem. Theory Comput.* **2011**, *7*, 625–632.

(46) Lu, T.; Chen, F. Multiwfn: A multifunctional wavefunction analyzer. *J. Comput. Chem.* **2012**, *33*, 580–592.

(47) Humphrey, W.; Dalke, A.; Schulten, K. VMD: Visual molecular dynamics. *J. Mol. Graph.* **1996**, *14*, 33–38.

(48) Williams and Kelley. Gnuplot 4.5: an interactive plotting program. **2011**. URL http://gnuplot.info.
